# Beyond natural flavonoids: exploring bioisosterism in design and synthesis of influenza endonuclease inhibitors†

**DOI:** 10.1039/d5md00071h

**Published:** 2025-03-11

**Authors:** Róbert Reiberger, Michal Kráľ, Kateřina Radilová, Tomáš Kotačka, Martin Dračínský, Artem Tsalyy, Jiří Brynda, Pavel Majer, Jan Konvalinka, Milan Kožíšek, Aleš Machara

**Affiliations:** a Institute of Organic Chemistry and Biochemistry of the Czech Academy of Sciences Flemingovo n. 2 166 10 Prague 6 Czech Republic milan.kozisek@uochb.cas.cz machara@uochb.cas.cz; b Department of Organic Chemistry, Faculty of Science, Charles University Hlavova 8 128 00 Prague 2 Czech Republic; c First Faculty of Medicine, Charles University Kateřinská 1660 121 08 Prague 2 Czech Republic; d Department of Biochemistry, Faculty of Science, Charles University Hlavova 8 128 00 Prague 2 Czech Republic; e Institute of Molecular Genetics of the Czech Academy of Sciences Vídeňská 1083 140 00 Prague 4 Czech Republic

## Abstract

Influenza virus, an RNA virus of the *Orthomyxoviridae* family, is responsible for widespread seasonal epidemics that result in 3 to 5 million severe illnesses and more than half a million deaths annually. Given the persistent circulation of pandemic influenza variants and increasing resistance to available inhibitors, there is an urgent need for new antiviral drugs effective against various viral subtypes. Viral RNA-dependent RNA polymerase, essential for viral replication, has emerged as a promising drug target. The PA subunit with endonuclease function is especially interesting, as development of the highly potent baloxavir marboxil (Xofluza) validated its importance as a novel drug target. Flavonoids have long been studied for their anti-influenza activity but have only recently been recognized as endonuclease inhibitors. We previously identified luteolin and its glucoside derivate, orientin, as potent endonuclease inhibitors, with their binding illustrated by X-ray crystallography structures. Building on this, we employed a scaffold-hopping approach based on the luteolin structure to design structurally distinct compounds that resemble the flavonoid scaffold. Using an AlphaScreen binding assay, we identified 33 as a submicromolar PA inhibitor with low toxicity. We solved the crystal structure of the PA endonuclease-binding pseudoflavonoid 36, which has similar structure and inhibitory potency to 33. Furthermore, we identified 24, 33, 34 and 36 as inhibitors of influenza polymerase in a minireplicon luciferase reporter assay as well as inhibitors of live H1N1 virus infection in A549 human lung cells.

## Abstract 2

Scaffold-hopping from luteolin led to a new class of submicromolar pseudoflavonoid-based influenza endonuclease inhibitors. A novel binding mode of compound 36 was observed in the PA-Nter active site.

## Graphical abstract

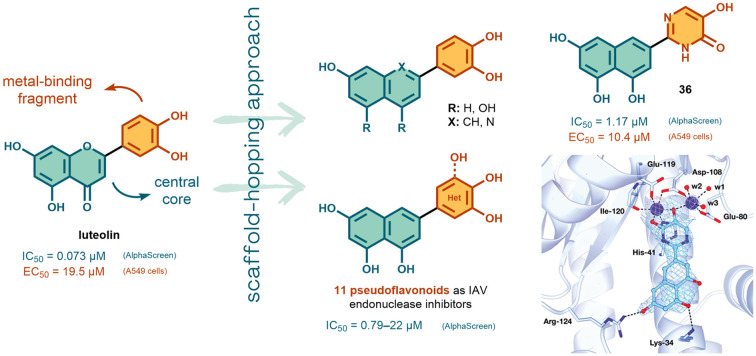

## 1 Introduction

Influenza A virus (IAV) remains a major public health concern due to its high virulence and rapid mutation rate, which have contributed to several human influenza pandemics over the past century. Despite substantial efforts to develop and disseminate vaccines, seasonal influenza epidemics result in millions of severe cases and hundreds of thousands of deaths worldwide annually.^1^ Antiviral drugs are the primary treatment for influenza infections. In recent years, antiviral compounds targeting RNA-dependent RNA polymerase^2–5^ (RdRp) have demonstrated acceptable efficacy, while viral mutations have diminished the effectiveness of neuraminidase inhibitors and M2 protein blockers. Influenza viruses contain a single-stranded, negative-sense RNA genome complexed with RdRp, which comprises PA, PB1, and PB2 subunits.^2,6^ The virus cannot synthesize the 5′-mRNA cap necessary for eukaryotic translation, representing a significant vulnerability and a target for drug development.^4,7^ The virus acquires host primers required for transcription initiation through a “cap-snatching” mechanism.^8^ This begins when the PB2 subunit binds to the 5′-cap (m7GTP) of host pre-mRNA. Next, the PA subunit cleaves the RNA strand approximately 10–13 nucleotides downstream from the 5′-cap to obtain the primer.^9,10^ The PB1 subunit subsequently uses this primer as a template for viral mRNA synthesis. Notably, RdRp, particularly the PA domain, is highly conserved across influenza strains, making it an attractive target for drug development.^4,11–13^ Research on inhibitors targeting all three subunits involved in the cap-snatching mechanism has been prolific.^3,7^

**Fig. 1:**
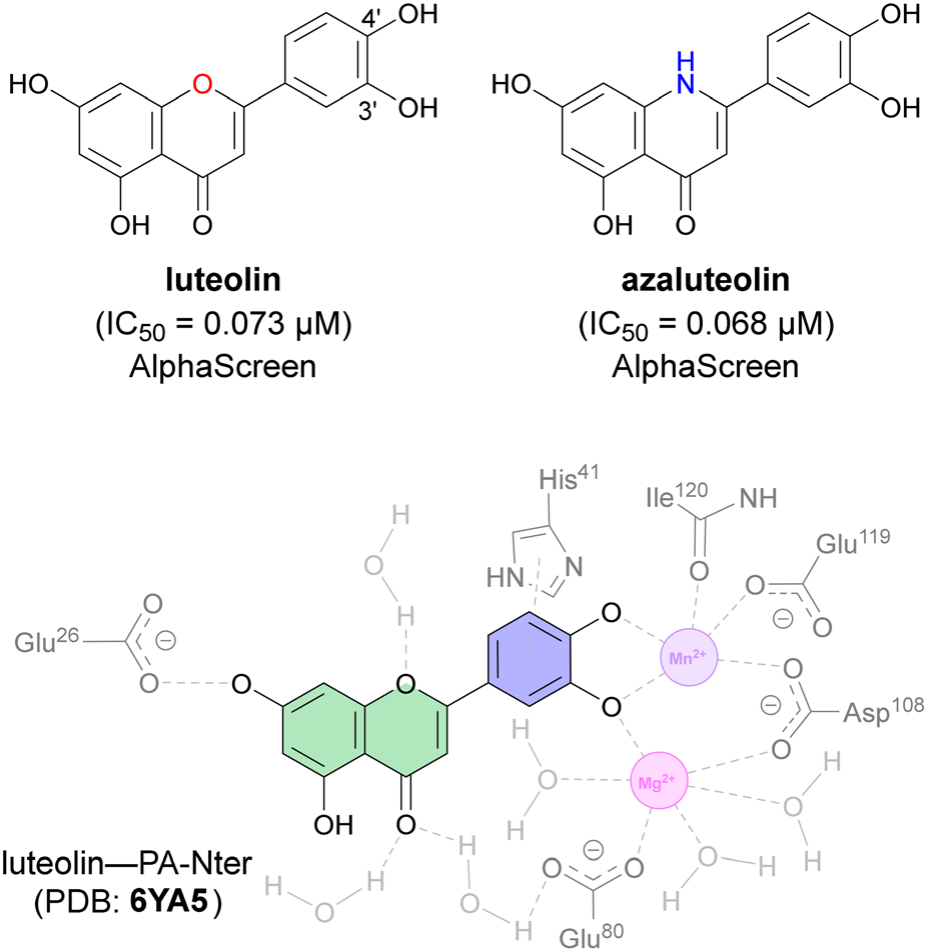
Flavonoid inhibitors of influenza endonuclease and graphical illustration of the binding mode of luteolin in the PA-Nter active site.

The PA subunit of influenza RdRp is a bridged binuclear metalloenzyme,^14^ with its N-terminal domain (PA-Nter) housing the endonuclease active site responsible for cleaving RNA segments.^10^ This active site is a negatively charged pocket that binds two Mg^2+^ or Mn^2+^ ions, with a stronger affinity for Mn^2+^.^15^ These ions are crucial for endonuclease function, and an inhibitor designed to target this site must include a metal-binding pharmacophore^14,16^ capable of binding Mg^2+^/Mn^2+^ ions efficiently.^17,18^ The most successful PA inhibitor to date is baloxavir marboxil (BMX),^19,20^ which was developed by Roche and Shionogi and has received regulatory approval in the USA and Japan. Although baloxavir marboxil showed favorable efficacy in clinical studies, recent studies indicate that influenza virus can develop resistance against baloxavir through I38T/M/F mutations in PA.^21–23^ This highlights the need for the development of new anti-influenza drugs that target endonuclease using a novel metal-binding pharmacophore. Approximately a dozen classes of PA-Nter endonuclease inhibitors have been documented, including diketo acids,^24^ dopamine derivatives,^25,26^ hydroxylated heterocycles,^27–32^ green tea catechins,^33,34^ flutamide derivatives,^35^ catechol congeners,^36^ 2,3-dihydroisoindole derivatives,^37^ carbamoyl pyridone derivatives,^29,38,39^ hydroxylated *N*-acylhydrazones^40^ and others.^41^ In 2020, we elucidated the molecular mode of action of flavonoids in influenza-infected cells.^42^ Using an AlphaScreen-based assay, we determined the inhibitory potencies of more than 30 flavonoids, identifying luteolin (Fig. 1, IC_50_ = 73 ± 3 nM) and its 8-*C*-glucoside orientin (IC_50_ = 42 ± 2 nM) as the most potent PA inhibitors. These results were corroborated by a gel-based endonuclease inhibitory assay. Furthermore, we performed structural analyses of PA-Nter complexes with luteolin and orientin, detailing their binding poses within the PA-Nter active site.^42,43^ The crystal structure of the PA-Nter complex with luteolin (PDB entry 6YA5, 2.0 Å resolution) revealed that the phenolic group at the C-7 position forms a hydrogen bond with the Glu-26 residue of PA-Nter (Fig. 1). The Mn^2+^ cation is coordinated by the atoms of the protein residues His-41, Asp-108, Glu-119, O Ile-120, and the catechol moiety of luteolin (3′,4′-dihydroxyphenyl moiety; see blue residue in Fig. 1). The Mg^2+^ cation is coordinated by Glu-80, Asp-108, the C-3′ phenolic group of luteolin, and three water molecules. The high affinity of luteolin can be attributed to surface complementarity and a strong hydrogen bonding network, evidenced by the well-defined electron density map of luteolin in the crystal structure.

Our structure–activity relationship (SAR) study indicated that the 3′,4′-dihydroxyflavone motif is crucial for inhibitory activity against PA.^42^ The presence of phenolic groups at the C-5 and C-7 positions significantly enhanced anti-influenza activity. Additionally, we observed that the corresponding aza-analogues, such as azaluteolin (Fig. 1), exhibited similar half-maximal inhibitory concentrations.

Over the past decades, it has become evident that there are significant differences between the biological properties of flavonoids observed *in vitro* and their bioactivity *in vivo*. Flavonoids exhibit two primary drawbacks for drug development. First, they are classified as pan-assay interference compounds (PAINS),^44,45^ meaning their polyphenolic structure frequently leads to false positives across a variety of biochemical assays. This chemical promiscuity must always be considered. Nonetheless, in our prior research, two different biochemical assays confirmed that flavonoids can inhibit PA.^46,47^ Moreover, X-ray crystallography of the luteolin–PA-Nter complex clearly revealed that it binds to the endonuclease active site. The second drawback is that flavonoids have limited bioavailability.^48–50^ The *in vivo* biological activity of flavonoids is clearly compromised by their poor bioavailability due to facile modifications like oxidation, glucuronylation or sulfation. This study outlines a rational design approach for influenza endonuclease inhibitors derived from the luteolin structure, aiming to pave the way for more effective *in cellulo* inhibitors. Specifically, we detail our medicinal chemistry efforts to develop a series of pseudoflavonoids using a scaffold-hopping strategy.

## 2 Results and discussion

### 2.1 Scaffold-hopping approach generates a series of pseudoflavonoids

**Scheme 1:**
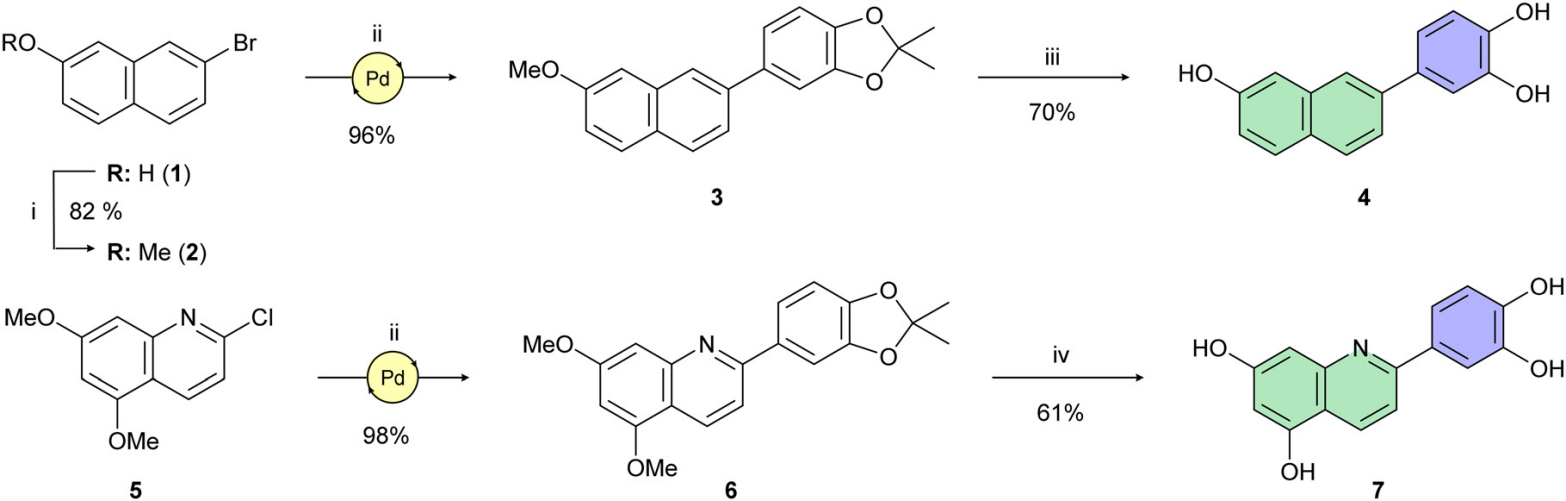
Reagents and reaction conditions: (i) MeI, K_2_CO_3_, DMF, r.t., 24 h; (ii) (2,2-dimethylbenzo[*d*][1,3]dioxol-5-yl)boronic acid, SPhos Pd G2 (10 mol%), K_2_CO_3_, THF/H_2_O (25 : 1), 100 °C, 16 h; (iii) BBr_3_, DCM, 0 °C → r.t., 1 h; (iv) BBr_3_, DCM, −78 °C → r.t.

To optimize the central core of luteolin using a scaffold-hopping approach,^51^ we had to examine the significance of individual hydroxy groups within the polyphenol structure. Initially, our attention was drawn to the simplest derivative 4 (Scheme 1), whose structure retains only the most essential OH group on the central naphthalene core. In luteolin, this C-7 phenolic group interacts with Glu-26 of the PA subunit.^46^ To synthesize 4 (depicted in Scheme 1), bromonaphthol 1 was methylated according to Kawara's protocol^52^ in high yield. Subsequent C–C cross-coupling using a Buchwald precatalyst gave benzodioxole 3 in 96% yield. A similarly high yield of Pd-catalyzed coupling was achieved in the synthesis of quinoline derivative 6 starting with dimethoxychloroquinoline 5. Treatment of 3 and 6 under standard *O*-demethylation conditions^53^ using boron tribromide afforded derivatives 4 and 7, respectively, in yields of around 65%.

**Scheme 2:**
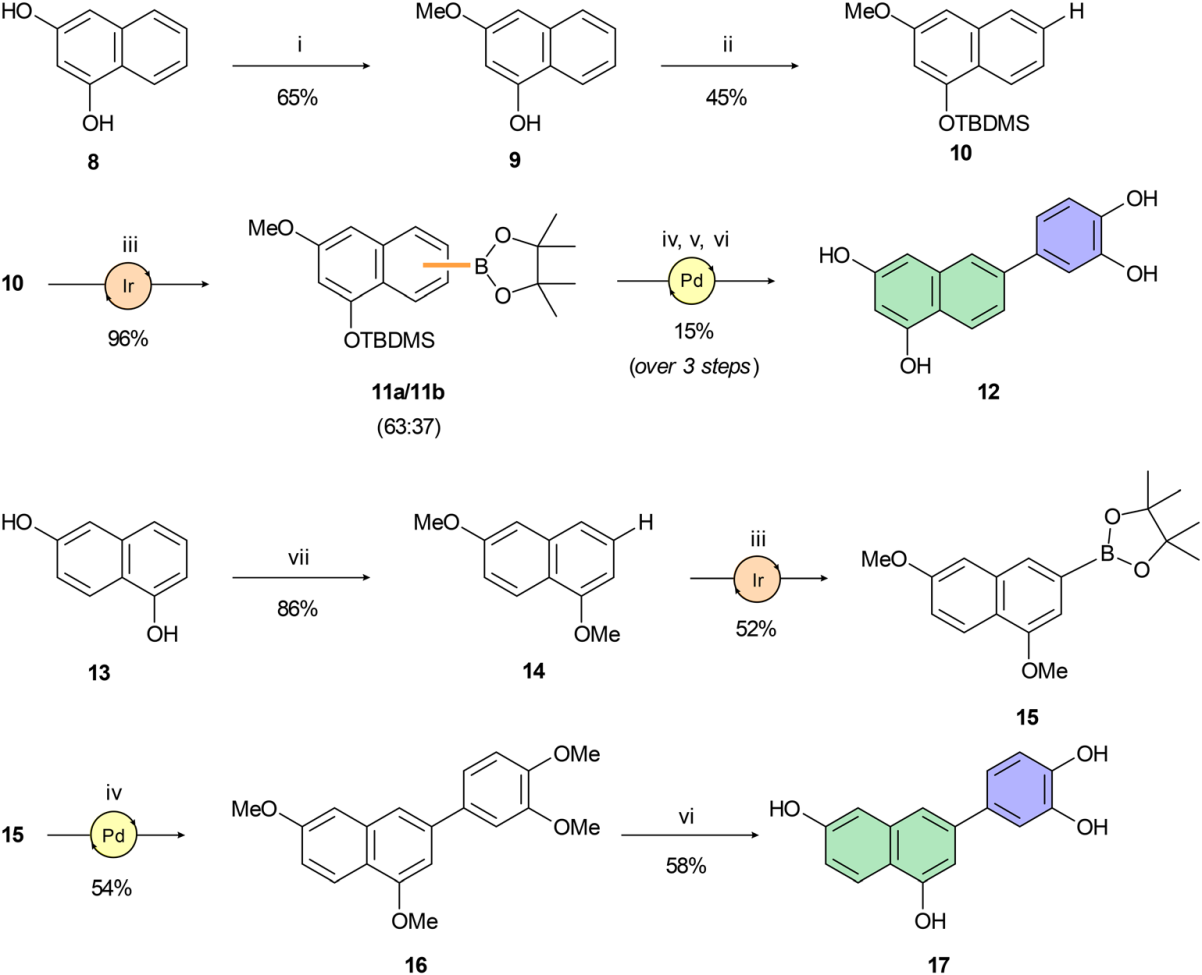
Reagents and reaction conditions: (i) 4 M HCl (1,4-dioxane), MeOH, 0 °C → r.t., 24 h; (ii) TBDMSCl, imidazole, THF, 0 °C → r.t., 16 h; (iii) B_2_(pin)_2_, [Ir(COD)OMe]_2_ (4 mol%), bbbpy (6 mol%), *n*-hexane, 100 °C, 16–72 h; (iv) 4-bromoveratrole, SPhos Pd G2 (10 mol%), K_2_CO_3_, THF/H_2_O (25 : 1), 100 °C, 16 h; (v) TBAF, THF, 0 °C, 2 h; (vi) BBr_3_, DCM, 0 °C → r.t., 2 h; (vii) MeI, K_2_CO_3_, DMF, r.t., 18 h.

Compounds 10 and 14, key intermediates for synthesis of isomeric tetraols 12 and 17, were prepared following protocols described in the literature^54–56^ (Scheme 2). Miyaura–Ishiyama–Hartwig borylations^57^ of 10 and 14 using C–H bond activation provided boronates 11 and 15 in acceptable yields ranging from 52% to 96%. However, high regioselectivity of borylation was achieved only for boronate 15. Despite the introduction of a bulky TBDMS group on the C-1 hydroxy group of 10, we were able to isolate only an inseparable mixture of regioisomers 11a and 11b in a roughly 2 : 1 ratio. Therefore, we used a pre-purified mixture of boronates 11a,b in a subsequent Suzuki–Miyaura cross-coupling reaction, again yielding an inseparable mixture of isomers. This mixture was deprotected in a two-step sequence using TBAF for desilylation and BBr_3_ for double *O*-demethylation. The pure product 12 was isolated after repeated preparative HPLC in an overall 15% yield (over 3 steps). Preparation of 17 was accomplished analogously (Scheme 2). Both the cross-coupling leading to 16 and the subsequent *O*-demethylation affording C-3 arylated 1,6-dihydroxynaphthalene 17 provided corresponding products in moderate yields.

**Scheme 3:**
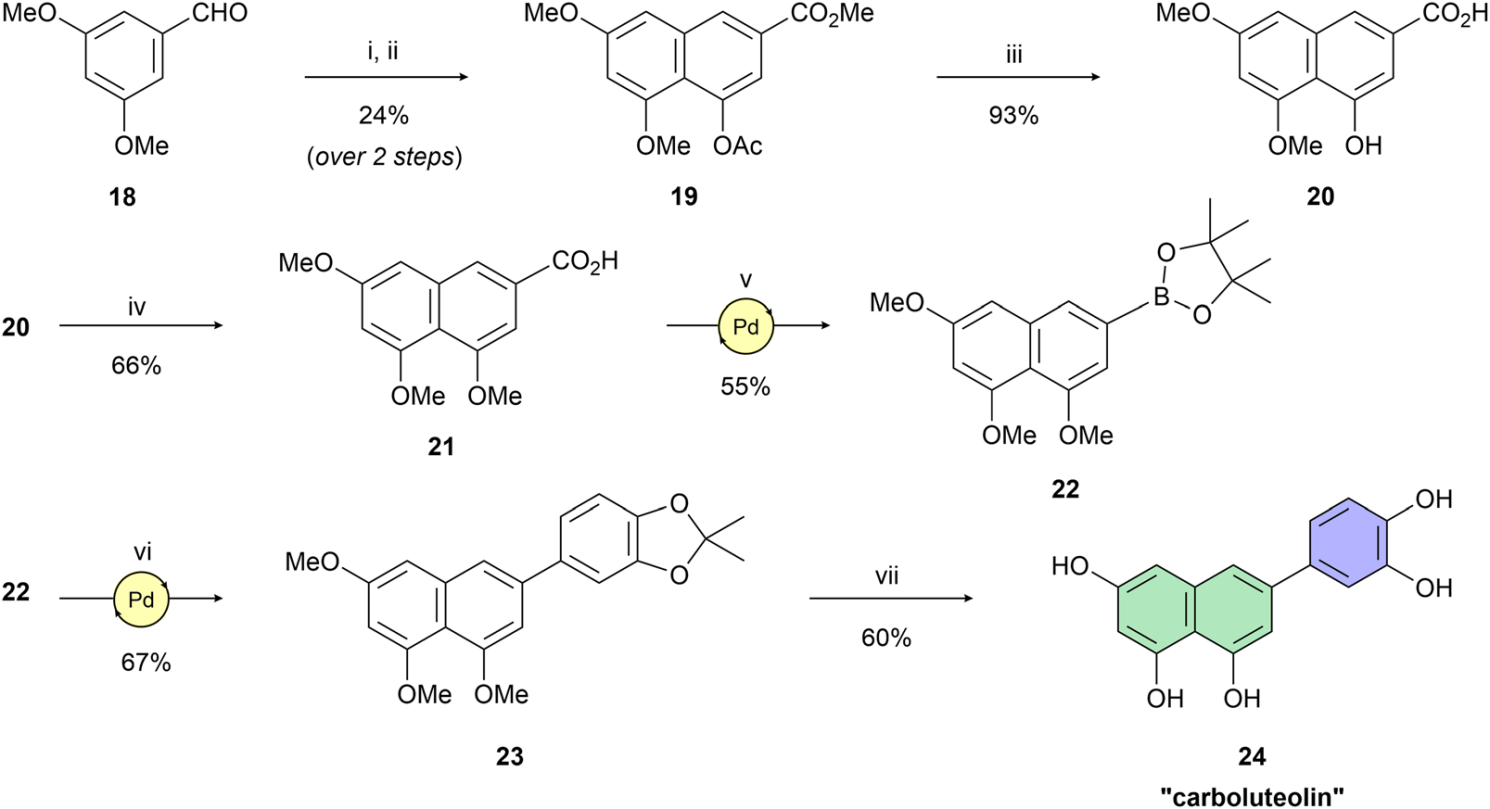
Reagents and reaction conditions: (i) dimethyl succinate, *t*-BuOK, *t*-BuOH, 90 °C, 3 h; (ii) Ac_2_O, KOAc, 145 °C, 6 h; (iii) 2.5 M NaOH (aq.), H_2_O, 100 °C, 1 h; (iv) MeI, NaH, benzyltriethylammonium chloride, DMF, 25 °C; 3 d; (v) B_2_(pin)_2_, Pd(OAc)_2_ (5 mol%), dppb (10 mol%), Et_3_N, Piv_2_O, 1,4-dioxane, 160 °C, 15 h; (vi) 5-bromo-2,2-dimethylbenzo[*d*][1,3]dioxole, SPhos Pd G2 (10 mol%), K_2_CO_3_, THF/H_2_O (25 : 1), 100 °C, 2 h; (vii) BBr_3_, DCM, 0 °C → r.t., 2 h.

Having naphthol 4, quinolone derivative 7, and dihydroxynaphthalene derivatives 12 and 17 on hand, our interest shifted to preparation of a trihydroxynapthalene derivative with three hydroxy groups aligned topologically as in luteolin. In theory, we expected 6-(3,4-dihydroxyphenyl)naphthalene-1,3,8-triol (24) to form interactions within the PA-Nter active site comparable to those of luteolin. We thus considered 24 as a prime candidate for the scaffold-hopping approach. Following a modified Cameron's approach,^58^ linear synthesis of 24 began with Stobbe condensation of 3,5-dimethoxybenzaldehyde and dimethyl succinate under strongly basic conditions (Scheme 3). 6-*Exo-trig* cyclization of the crude intermediate with acetic anhydride and potassium acetate resulted in formation of ester 19. Next, acid 20 was obtained through saponification. A subsequent domino methylation/saponification reaction in DMF containing a trace amount of water provided acid 21 in 66% yield. Catalytic decarbonylative borylation of 21 by the Szostak protocol^59^ afforded boronate 22 in 55% yield. The synthesis of carboluteolin (24) was subsequently accomplished using a similar reaction sequence to that of the dihydroxynaphthalene derivative 17.

**Scheme 4:**
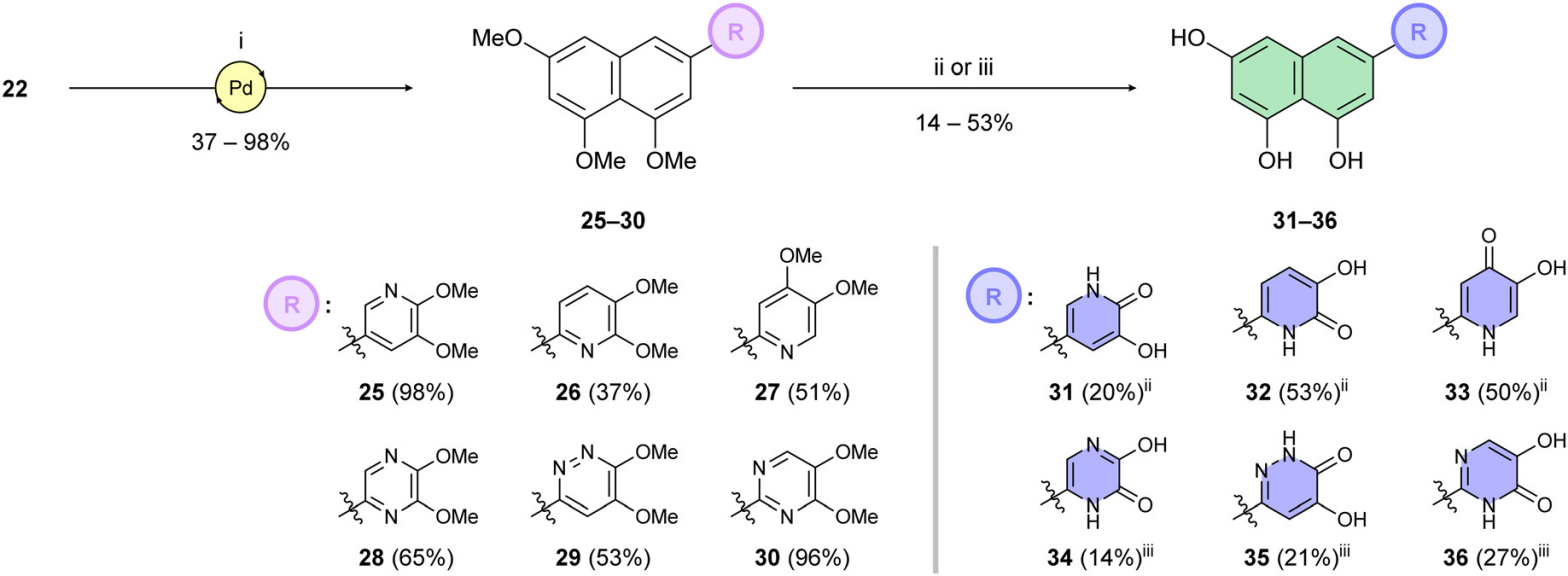
Reagents and reaction conditions: (i) heteroarylhalide (39/40/41/43/44/45), Pd(PPh_3_)_4_ (10 mol%), K_2_CO_3_, 1,4-dioxane/H_2_O (3 : 1), 100 °C, 16 h; (ii) BBr_3_, DCM, 0 °C → r.t., 48 h; (iii) (a) BBr_3_, DCM, 0 °C → r.t., 6–24 h; (b) 4 M HCl (1,4-dioxane), 1,4-dioxane, 110 °C, 16 h, 5–24 h.

Building on this foundation, we designed and synthesized a series of compounds in which azacyclic metal-binding pharmacophores are attached to the 1,3,8-trihydroxynaphthalene core, thus replacing the metabolically labile 3′,4′-dihydroxyphenyl (catechol) moiety of the parent luteolin structure. We hypothesize that these azacyclic derivatives, which are likely metabolized *via* distinct pathways compared to the catechol moiety, could demonstrate enhanced antiviral activity in tissue culture. Boronate 22 was subjected to Suzuki–Miyaura cross-coupling with the corresponding heteroaryl halides (Scheme 4). Optimization of the catalytic systems indicated that Pd(PPh_3_)_4_ was a more efficient option than Buchwald precatalysts. Surprisingly, these palladacycle-based catalysts initially provided 25 in yields up to only 25%. On the other hand, the Pd(PPh_3_)_4_ catalyst led to the preparation of C-6 substituted per-*O*-methylated naphthalentriols 25–30 in yields of 37–98%. A standard one-step *O*-demethylation using BBr_3_ was successful only for pyridine derivatives 25–27. Due to the lower reactivity of 28–30 towards BBr_3_, a procedure described by Sagong^60^ was employed. Although BBr_3_ was used in the first step, after evaporation of the reaction mixture, the crude product was further combined with 4 M hydrogen chloride in 1,4-dioxane at elevated temperature. This approach yielded pseudoflavonoids 31–36 containing azacycle moieties with yields varying from 14% to 53%.

### 2.2 AlphaScreen highlights the importance of the trihydroxynaphthalene core and enables selection of lead compounds

We first assessed the inhibitory potencies of the synthesized compounds using an AlphaScreen binding assay.^46,61^ Our initial SAR study aimed to identify the hydroxy groups on the naphthalene and quinoline scaffolds that are essential for optimal inhibitory potency (Table 1). The parent compound luteolin inhibited endonuclease with an IC_50_ of 0.073 μM. Compound 4, which bears a single phenolic group on the central core, exhibited an approximately two-orders-of-magnitude weaker inhibitory potency (IC_50_ = 22 μM). Structurally similar derivatives 7 and 12 had greater potency than 4 (IC_50_ = 8.1 and 7.7 μM, respectively), highlighting the positive impact of two hydroxy groups at C-5 and C-7 (flavonoid numbering) on inhibitory potency. Compound 17, a structural isomer of 12 that retains the phenolic group at C-7 but has a second phenolic group at C-4, proved to be a superior inhibitor compared to 12. Compound 24 exhibited an IC_50_ of 0.38 μM, indicating that a specific trihydroxynaphthalene core is necessary for submicromolar inhibitory potency. Furthermore, its fivefold higher IC_50_ value compared to that of luteolin suggests that, despite having similar footprints, luteolin and 24 slightly differ in their binding interactions with the endonuclease active site.

**Table 1:** Inhibition assay of influenza endonuclease. Structure–activity relationship of polyphenol derivatives

Compound	Structure	IC_50_ ± SD (μM) (AlphaScreen)
Luteolin	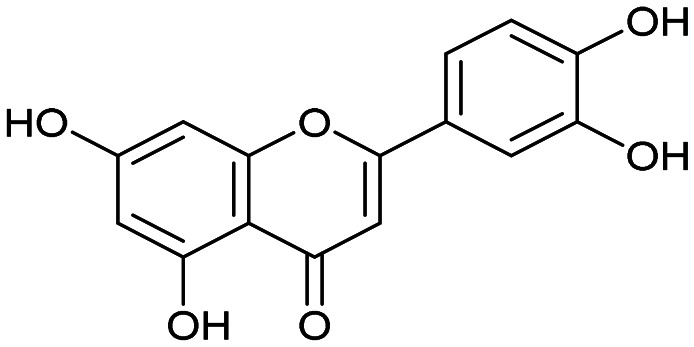	0.073 ± 0.003
4	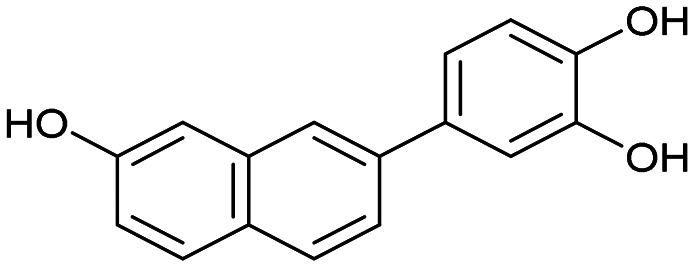	22 ± 4
7	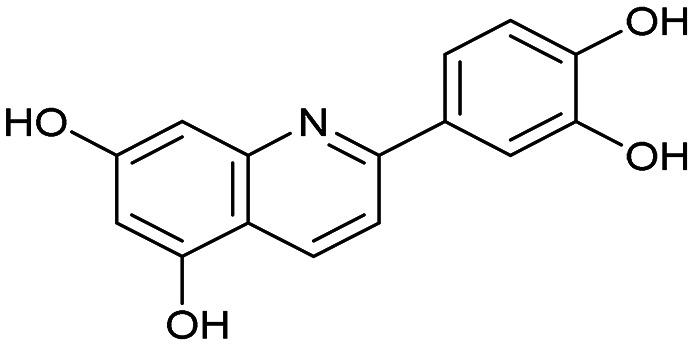	8.1 ± 0.8
12	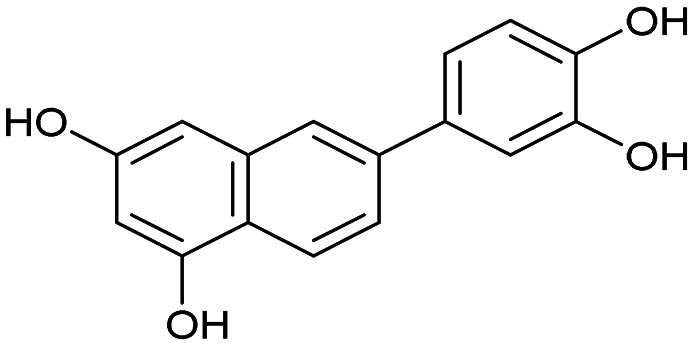	7.7 ± 0.4
17	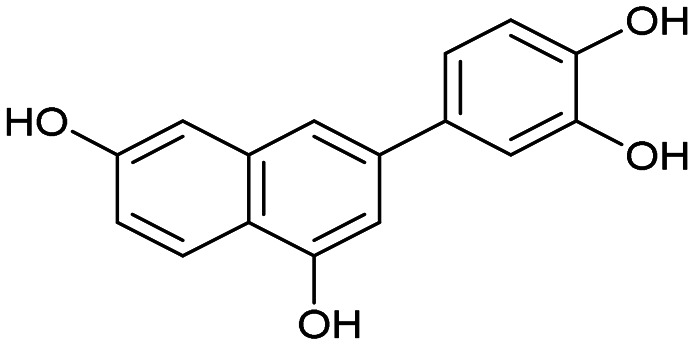	3.9 ± 1.2
24	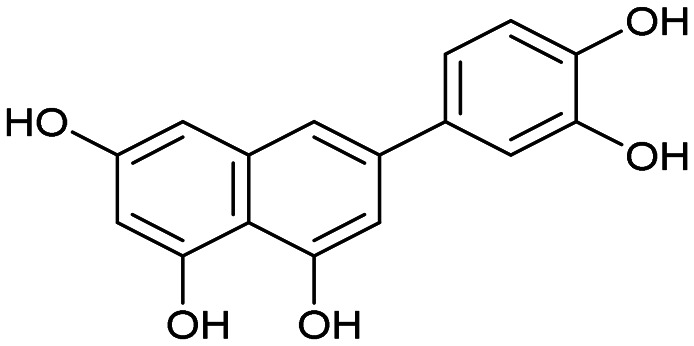	0.38 ± 0.06

Having identified the minimal number of phenolic groups on the naphthalene core required for submicromolar inhibitory potency, we proceeded to investigate carboluteolin analogues with hydroxylated azacycles replacing the metabolically vulnerable 3′,4′-dihydroxyphenyl motif (Table 2). Isomeric compounds 31 and 32, containing 3-hydroxypyridin-2(1*H*)-one-5-yl^62^ and 3-hydroxypyridin-2(1*H*)-one-6-yl^62^ moieties, had lower inhibitory potencies than 24, with IC_50_ values greater than 4 μM. Conversely, 33, which features a 5-hydroxypyridine-4(1*H*)-one-2-yl metal-binding pharmacophore,^18^ demonstrated submicromolar inhibitory potency. Derivatives 34 and 35, with 3-hydroxypyrazin-2(1*H*)-one-6-yl^60^ and 4-hydroxypyridazin-3(2*H*)-6-yl^60^ moieties, respectively, displayed reduced inhibitory potencies, with IC_50_ values of 3.3 and 12 μM. The IC_50_ value determined for 36 was between those of 33 and 32, reflecting the structural similarities between hydroxypyridinone and hydroxypyrimidinone^60^ moieties. Based on the results of this SAR analysis, we selected compounds 24, 33, 34 and 36 for further evaluation.

**Table 2:** Inhibition assay of influenza endonuclease. Structure–activity relationship of pseudoflavonoids

Compound	Structure	IC_50_ ± SD (μM) (AlphaScreen)
Luteolin	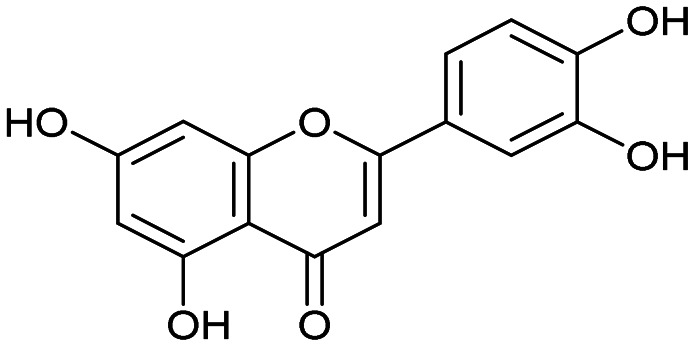	0.073 ± 0.003
Azaluteolin	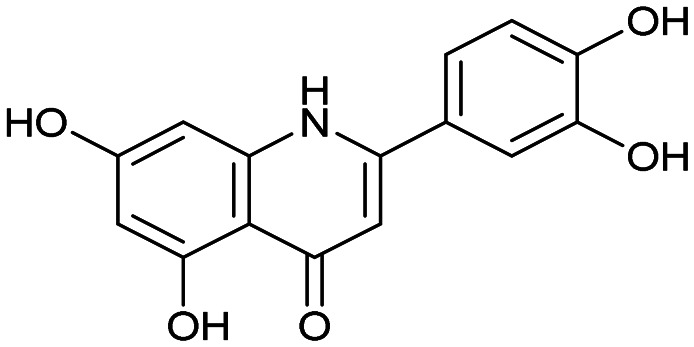	0.068 ± 0.002
24 (carboluteolin)	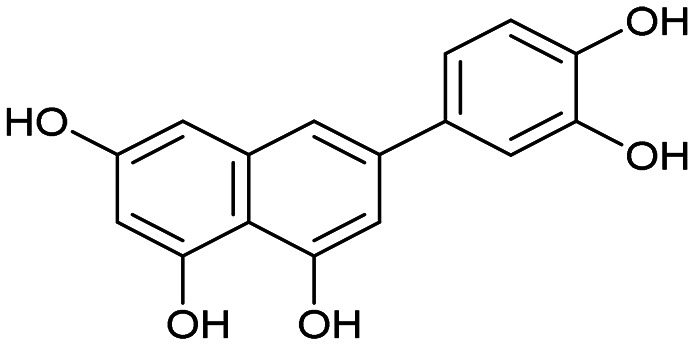	0.38 ± 0.06
31	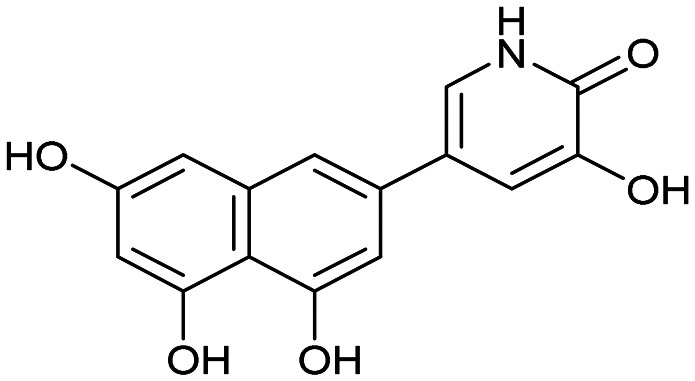	8.8 ± 0.6
32	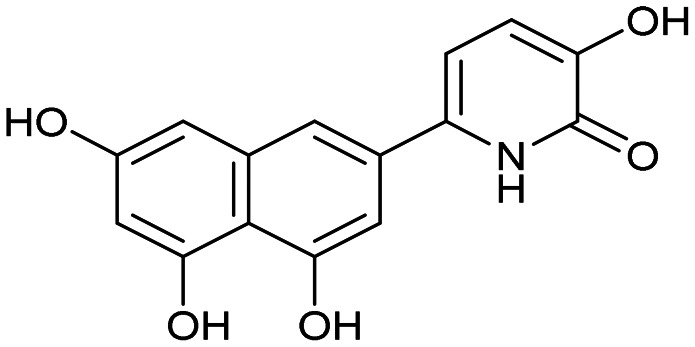	4.3 ± 0.4
33	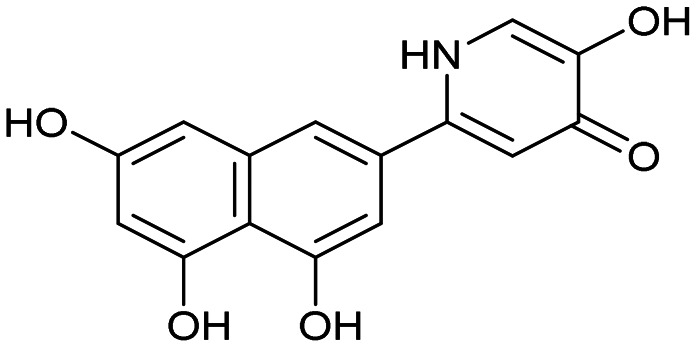	0.79 ± 0.1
34	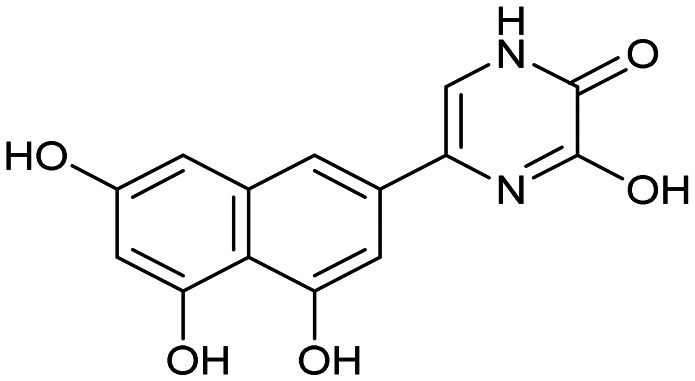	3.3 ± 0.1
35	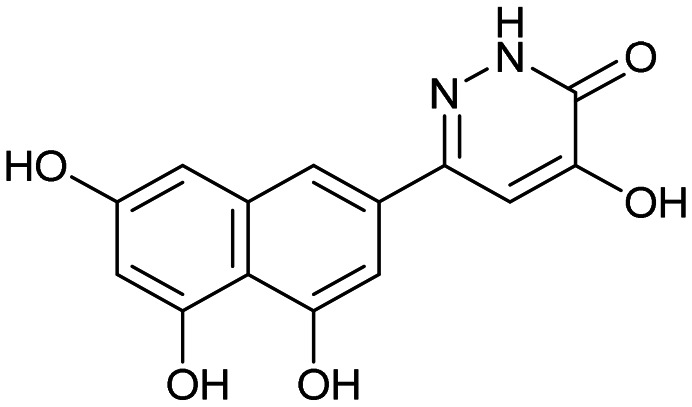	12 ± 2
36	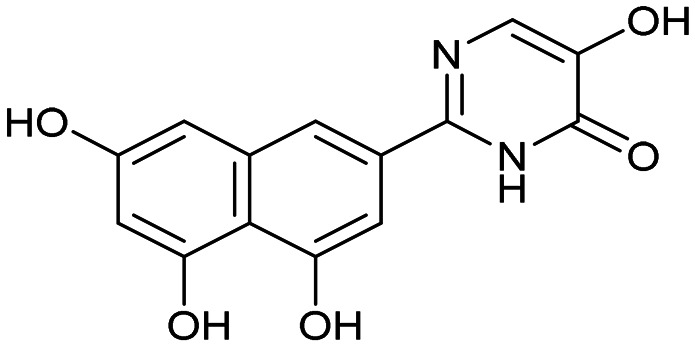	1.7 ± 0.3

### 2.3 Pseudoflavonoids inhibit endonuclease and exhibit antiviral activity in cell-based assays

Based on the good performance of 24, 33, 34 and 36 in the AlphaScreen binding assay, we further characterized their inhibitory potency using two approaches: (i) a minireplicon (or minigenome) luciferase reporter assay based on transfection of plasmids encoding active influenza A polymerase into cells and (ii) a cytopathic effect reduction assay using infectious influenza H1N1 virus. First, we employed the minigenome strategy; all four selected compounds showed promising inhibition in transfected HEK 293T cells, with IC_50_ values in the low micromolar range (1.1–2.1 μM, Table 3). The parent compound luteolin did not show any inhibition of influenza A polymerase.

**Table 3:** Antiviral activity in cell-based assays

Compound	EC_50_ minireplicon (μM)		EC_50_ antiviral testing (μM)		CC_50_ cytotoxicity (μM)		
	HEK 293T	A549	MDCK	A549	HEK 293T	A549	MDCK
24	1.1 ± 0.1	0.8 ± 0.2	>100	12 ± 1	>50	>50	>50
33	2.1 ± 0.2	2.8 ± 0.6	41 ± 13	2.4 ± 0.4	>50	>50	>50
34	1.8 ± 0.1	1.7 ± 0.4	>100	>100	>50	>50	>50
36	1.4 ± 0.2	1.8 ± 0.5	>100	10 ± 1	>50	>50	>50
Luteolin	>100	9.2 ± 4.1	>100	20 ± 2	>50	>50	>50
BMX	0.002 ± 0.0006	0.004 ± 0.001	0.030 ± 0.008	1.8 ± 0.4	>50	>50	>50
BXA	n.d.	n.d.	0.070 ± 0.017	0.093 ± 0.013	n.d.	14 ± 3	5 ± 1

We next tested the compounds in a cytopathic effect reduction assay using Madin–Darby canine kidney (MDCK) cells, which are generally used for anti-influenza inhibitor testing. However, luteolin and all the selected compounds except 33 (EC_50_ = 41 ± 13 μM) exhibited very poor or no activity (EC_50_ >100 μM). This effect is likely due to the poor permeability of MDCK cells to the tested compounds, as our control compound, baloxavir marboxil, was able to stop the infection in its usual concentration range (EC_50_ = 0.030 ± 0.008 μM). To overcome this, we switched from MDCK cells to A549 cells, human lung adenocarcinoma cells that are susceptible to influenza virus infection. Using A549 cells, we were able to determine the inhibitory potencies of 24 (EC_50_ = 12 ± 1 μM), 33 (EC_50_ = 2.4 ± 0.4 μM) and 36 (EC_50_ = 10 ± 1 μM). Interestingly, although luteolin did not exhibit any antiviral activity in MDCK cells, it had a weak effect in A549 cells (EC_50_ = 20 ± 2 μM). The control compound, baloxavir marboxil, exhibited markedly decreased inhibitory potency in A549 cells (EC_50_ = 1.8 ± 0.4 μM) compared to MDCK cells. As an additional control, we used baloxavir acid, the active form of baloxavir marboxil. Interestingly, it retained its inhibitory potency in nanomolar range in both A549 and MDCK cells. This active form exhibited moderate cytotoxicity, which was, however, well outside of the inhibitory range.

### 2.4 X-ray crystallography reveals details of pseudoflavonoid binding to influenza endonuclease

**Fig. 2:**
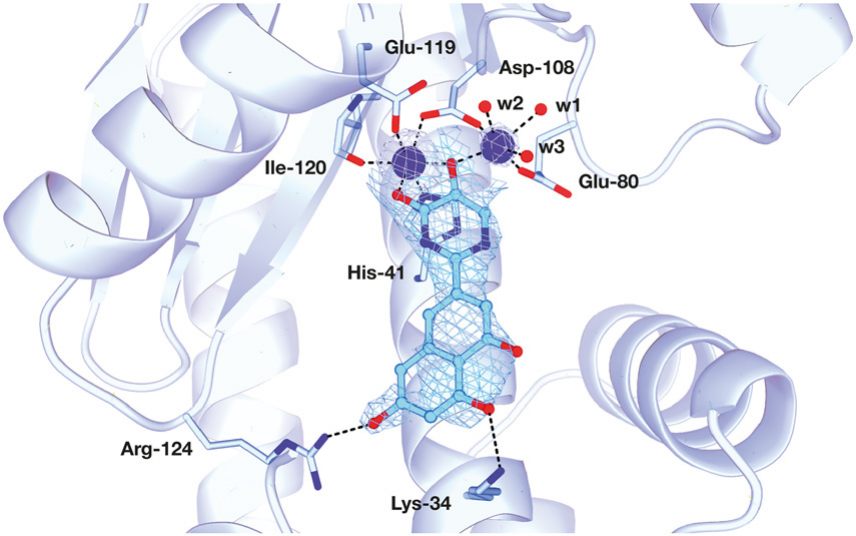
Close-up view of PA-Nter with bound ligand 36 (PDB entry 8PPX). Metal ions (left – manganese, right – magnesium) are shown as purple-blue spheres. Compound 36 (in cyan stick representation) coordinates the manganese ion with its C-5′ hydroxy and C-4′ keto moieties. The hydroxy group at C-7 (flavonoid numbering) of 36 forms a hydrogen bond with N^η2^ Arg-124, and the C-5 hydroxy group with N^ζ^ Lys-34. The ligand electron density map (blue mesh) is contoured at 1.5*σ*, and the electron density for anomalous scattering (white mesh) is contoured at 5*σ*. Interacting side chains of PA-Nter are shown as blue sticks. Other color coding: oxygen – red, nitrogen – dark blue.

To confirm the binding of pseudoflavonoids in the active site of PA-Nter, we selected 36 (IC_50_ = 1.7 ± 0.3 μM) to be soaked into empty protein crystals, as the most active compound, 33, had not yet been synthesized at the time. The structure of PA-Nter in complex with 36 (PDB ligand 8PPX) was refined to 2.5 Å. Each asymmetric unit consisted of one PA-Nter molecule. In alignment with earlier PA-Nter structures,^33^ two octahedrally coordinated metal ions were present in the active site (Fig. 2). The distal magnesium ion with a weaker anomalous signal was coordinated by O^ε2^ Glu-80, O^δ^ Asp-108, three water molecules (w1, w2, w3), and the C-5′ hydroxy group of 36. The proximal site was fully occupied by the manganese ion with a strong anomalous signal (up to 2.75 Å). The manganese ion lies in the center of an octahedral coordination sphere including four protein atoms (N^ε2^ His-41, O^ε2^ Asp-108, O^ε2^ Glu 119, O Ile-120) and the C-5′ hydroxy and C-4′ keto groups of 36.

**Fig. 3:**
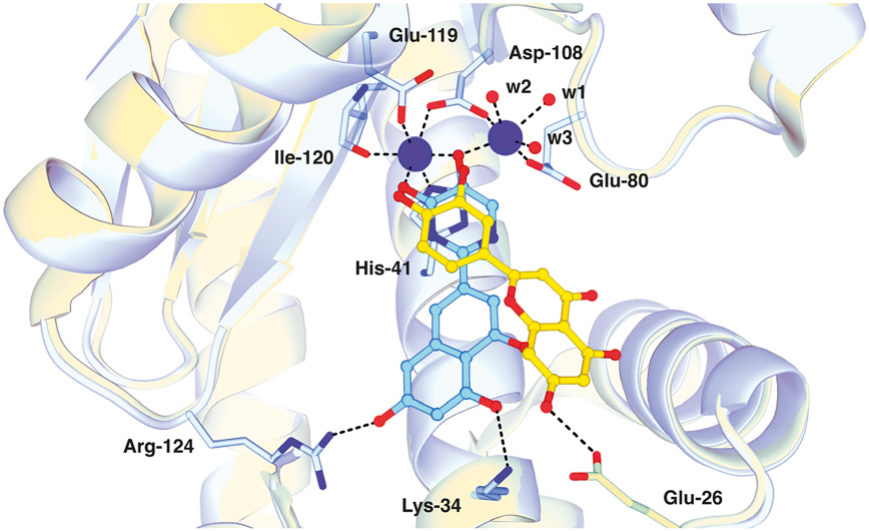
Alignment of PA-Nter in complex with 36 (cyan, PDB entry 8PPX) and luteolin (yellow, PDB entry 6YA5). Both 36 and luteolin coordinate metal ions in the protein active site *via* hydroxylated (hetero)aryl moieties (hydroxypyrimidone and 3′,4′-dihydroxyphenyl moieties, respectively). In the case of luteolin, the C-7 hydroxy group forms a hydrogen bond with O^ε2^ Glu-26. This represents a distinct binding mode compared to that of 36, in which the 3′,4′-dihydroxyphenyl moiety is rotated by approximately 180 degrees, resulting in the C-7 hydroxy group interacting with the side chain of Arg-124. PA-Nter is in cartoon representation with interacting side chains shown as sticks. Color coding: oxygen – red, nitrogen – dark blue, carbon atoms of protein – blue (PA-Nter/36) and yellow (PA-Nter/luteolin).

Compound 36 binds in the PA-Nter domain active site^46^ with a similar coordination pattern to that of luteolin. Like luteolin, one hydroxy and one keto group of the hydroxypyrimidinone moiety of 36 chelate the manganese ion. However, the positions of the C-5′ hydroxy and C-4′ keto groups of 36 are rotated by 180 degrees relative to luteolin's hydroxy groups, resulting in a distinct orientation of the central naphthalene core of 36 within the PA-Nter active site. Structural superposition of 36 and luteolin gave an RMSD of 4.2 Å for nineteen corresponding atoms. Additionally, the C-7 hydroxy group (luteolin numbering) of 36 formed a hydrogen bond with Arg-134; in the PA-Nter–luteolin complex; this interaction occurs with O^ε2^ Glu-26 (Fig. 3). The C-5 hydroxy group of 36 also formed an interaction with N^ζ^ Lys-34.

## 3 Conclusions

We report the synthesis and biochemical evaluation of a series of luteolin bioisosteres. These unprecedented compounds, named “pseudoflavonoids”, were designed as inhibitors of influenza endonuclease. Applying a scaffold-hopping approach involving replacement of 3′,4′-dihydroxyphenyl with hydroxylated azacycles, we obtained a few compounds with moderate in-cell activity. Optimizing the topology of the hydroxypyridinone moiety serving as a metal-binding pharmacophore led to discovery of 33, which exhibited submicromolar inhibitory potency. The scaffold-hopping approach furnished compounds that exhibited increased inhibitory potencies in cells (minireplicon and cytopathic effect reduction assays) compared to luteolin. Interestingly, commonly used MDCK cells proved to be unsuitable for testing of these compounds, possibly due to their poor cellular permeability. However, we were able to establish the inhibitory effect of our compounds (24, 33, 34 and 36) in human lung A549 cells and confirm it with a luciferase reporter assay. In a cytopathic effect reduction assay using the infectious influenza H1N1 virus and human lung adenocarcinoma cells, compound 33 demonstrated a comparable inhibitory potency to baloxavir marboxil, which is currently used in clinical practice (EC_50_ = 2.4 ± 0.4 μM *versus* EC_50_ = 1.8 ± 0.4 μM). Through X-ray crystallography structural analysis, we elucidated similarities and differences in the binding motif of 36 compared to luteolin. The “pseudoflavonoids” described here share a relatively high structural similarity with flavonoids, particularly due to the presence of phenolic groups. Nevertheless, these compounds represent important stepping stones toward the design of flavonoid bioisosteres with significantly improved properties enabling them to be used for the treatment of important human diseases.

## 4 Experimental section

### 4.1 Chemistry

Unless otherwise noted, all reactions were carried out under argon in oven-dried glassware. Anhydrous solvents were distilled from solvents using the indicated agents and transferred under nitrogen: THF (Na/benzophenone), toluene (Na/benzophenone), MeCN (CaH_2_), and DCM (CaH_2_). Chromatography was performed using a Teledyne ISCO CombiFlash NextGen 300+ flash chromatography system with RediSep Rf Gold Silica or RediSep (marked as SiO_2_) Rf Gold Reversed-phase C18 columns (marked SiO_2_-C_18_ or SiO_2_-C_18_-Aq). All starting materials were used as purchased (Merck, Alfa Aesar, TCI, Fluorochem, Combi-Blocks) unless otherwise indicated. All inhibitors were purified using an ECOM compact preparative system TOY18DAD800 (flow rate 15 mL min^−1^; gradient 0 → 60% or 0 → 80% H_2_O (0.1% trifluoroacetic acid)/MeCN in 60 min) with a ProntoSIL 120-10-C18 ace-EPS column, 10 μm, 20 × 250 mm. The purity of compounds and composition of the reaction mixtures were tested on a Waters UPLC-MS ACQUITY system with a QDa Mass Detector (flow rate 0.5 mL min^−1^, gradient 0 → 100% H_2_O (0.1% formic acid)/MeCN in 7 min) and an ACQUITY UPLC BEH C18 Column, 130 Å, 1.7 μm, 2.1 mm × 100 mm with a 2.1 mm × 5 mm pre-column. The final inhibitors were of at least 95% purity. ^1^H NMR spectra were recorded on Bruker instruments at 401, 500 or 600 MHz; ^13^C NMR spectra were recorded at 101, 126 or 151 MHz. Chemical shifts are provided in *δ*-scale in ppm; coupling constants (*J*) are given in Hz. Signals marked with an asterisk (*) were visible in two-dimensional NMR spectra (HMBC). FT-IR spectra were recorded using a Nicolet iS50 spectrophotometer. Wavenumbers are provided in *ν̃*-scale in cm^−1^. ESI or APCI high-resolution mass spectra were recorded using a Thermo Scientific LTQ Orbitrap XL spectrometer (Thermo Fisher Scientific) and EI or CI high-resolution mass spectra were recorded using an Agilent 7250 GC/Q-TOF system, both controlled by MassLynx software.

#### 4.1.1 Inhibitor synthesis

##### 2-Bromo-7-methoxynaphthalene (2)

7-Bromonaphthalen-2-ol (1) (500 mg, 2.24 mmol, 1.0 eq.) was dissolved in DMF (10 mL), followed by addition of K_2_CO_3_ (928 mg, 6.72 mmol, 3.0 eq.) and iodomethane (1.05 mL, 16.8 mmol, 7.5 eq.). The reaction mixture was allowed to stir at room temperature overnight until the starting material was consumed (TLC, UPLC-MS). The mixture was quenched with saturated NH_4_Cl (2.0 mL) followed by addition of EtOAc (15 mL). The organic phase was washed with water (3 × 20 mL) and brine (1 × 10 mL) and dried over anhydrous MgSO_4_. The solvents were evaporated, and the residue was purified by flash chromatography (SiO_2_, *n*-hexane/EtOAc = 100 → 90 : 10) to afford the desired compound 2 (435 mg, 82%). ^1^H NMR (401 MHz, CDCl_3_) *δ* = 7.90 (dd, *J* = 2.0, 0.7 Hz, 1H), 7.69 (dd, *J* = 8.9, 0.7 Hz, 1H), 7.66–7.57 (m, 1H), 7.41 (dd, *J* = 8.7, 2.0 Hz, 1H), 7.15 (dd, *J* = 9.0, 2.5 Hz, 1H), 7.02 (d, *J* = 2.5 Hz, 1H), 3.91 (s, 3H) ppm. ^13^C NMR (101 MHz, CDCl_3_) *δ* = 158.5, 135.9, 129.5, 129.4, 128.8, 127.4, 127.0, 120.7, 119.3, 105.0, 55.5 ppm. LRMS (APCI) *m*/*z* calcd for C_11_H_10_BrO [M + H^+^]^+^ 236.99, found 236.46.

##### 5-(7-Methoxynaphthalen-2-yl)-2,2-dimethylbenzo[*d*][1,3]dioxole (3)

A tube with 2-bromo-7-methoxynaphthalene (2) (100 mg, 0.42 mmol, 1.0 eq.), (2,2-dimethylbenzo[*d*][1,3]dioxol-5-yl)boronic acid (123 mg, 0.63 mmol, 1.5 eq.), K_2_CO_3_ (232 mg, 1.68 mmol, 4.0 eq.) and SPhos Pd G2 (30 mg, 0.042 mmol, 0.1 eq.) was sealed and THF/water (25 : 1, 5.3 mL) was added *via* septum. The mixture was degassed by a stream of argon for 15 minutes, followed by heating to 100 °C for 16 hours until the starting material was consumed (TLC, UPLC-MS). The reaction mixture was cooled to room temperature, filtered through Celite, and washed with EtOAc. The solvents were evaporated, and the residue was purified by flash chromatography (SiO_2_, *n*-hexane/EtOAc = 100 : 0 → 85 : 15) to afford the desired product 3 (123 mg, 96%). ^1^H NMR (401 MHz, CDCl_3_) *δ* = 7.86 (d, *J* = 1.8 Hz, 1H), 7.80 (d, *J* = 8.5 Hz, 1H), 7.74 (d, *J* = 8.8 Hz, 1H), 7.53 (dd, *J* = 8.5, 1.8 Hz, 1H), 7.21–7.09 (m, 4H), 6.85 (d, *J* = 8.0 Hz, 1H), 3.95 (s, 3H), 1.74 (s, 6H) ppm. ^13^C NMR (101 MHz, CDCl_3_) *δ* = 158.1, 148.1, 147.1, 139.2, 135.1, 135.0, 129.2, 128.2, 128.0, 124.3, 123.4, 120.4, 118.6, 118.3, 108.6, 107.8, 106.0, 55.4, 53.5, 26.0 ppm. HRMS (APCI) *m*/*z* calcd for C_20_H_19_O_3_ [M + H^+^]^+^ 307.1329, found 307.1329.

##### 4-(7-Hydroxynaphthalen-2-yl)benzene-1,2-diol (4)

Compound 3 (123 mg, 0.4 mmol, 1.0 eq.) was dissolved in anhydrous CH_2_Cl_2_ (1.4 mL). The solution was cooled to 0 °C in an ice water bath, and a 1 M solution of BBr_3_ in CH_2_Cl_2_ (3 mL, 3.0 mmol, 7.5 eq.) was added dropwise under a nitrogen atmosphere. Then, the reaction mixture was allowed to stir for 15 minutes at 0 °C, followed by 1 hour at room temperature until the starting material was consumed (UPLC-MS). The reaction mixture was poured into a mixture of ice/water (20 mL). After 30 minutes of stirring, the resulting white suspension was extracted with EtOAc (3 × 20 mL). The combined organic phases were dried over anhydrous MgSO_4_. The solvents were evaporated, and the residue was purified by preparative HPLC to afford the desired final product 4 (71 mg, 70%). ^1^H NMR (401 MHz, DMSO-*d*_6_) *δ* = 9.70 (s, 1H), 9.11–9.05 (m, 1H), 9.05–9.01 (m, 1H), 7.88–7.70 (m, 3H), 7.46 (dd, *J* = 8.5, 1.8 Hz, 1H), 7.16 (d, *J* = 2.2 Hz, 2H), 7.04 (ddd, *J* = 8.8, 7.5, 2.3 Hz, 2H), 6.85 (d, *J* = 8.2 Hz, 1H) ppm. ^13^C NMR (101 MHz, DMSO-*d*_6_) *δ* = 155.6, 145.6, 145.2, 138.0, 135.0, 131.7, 129.0, 128.0, 126.5, 122.3, 121.7, 118.2, 118.0, 116.1, 114.2, 108.9 ppm. HRMS (ESI) *m*/*z* calcd for C_16_H_11_O_3_ [M–H^+^]^−^ 251.0714, found 251.0713.

##### 2-(2,2-Dimethylbenzo[*d*][1,3]dioxol-5-yl)-5,7-dimethoxyquinoline (6)

A tube with 2-chloro-5,7-dimethoxyquinoline (5) (100 mg, 0.44 mmol, 1.0 eq.), (2,2-dimethylbenzo[*d*][1,3]dioxol-5-yl)boronic acid (133 mg, 0.67 mmol, 1.5 eq.), K_2_CO_3_ (243 mg, 1.76 mmol, 4.0 eq.) and SPhos Pd G2 (32 mg, 0.044 mmol, 0.1 eq.) was sealed and THF/water (25 : 1, 5.5 mL) was added *via* septum. The mixture was degassed by a stream of argon for 15 minutes, followed by heating to 100 °C for 3 hours until the starting material was consumed (TLC, UPLC-MS). Then, the reaction mixture was cooled to room temperature, filtered through Celite, and washed with EtOAc. The solvents were evaporated, and the residue was purified by flash chromatography (SiO_2_, *n*-hexane/EtOAc = 100 : 0 → 80 : 20) to afford the desired product 6 (149 mg, 98%). ^1^H NMR (401 MHz, CDCl_3_) *δ* = 8.39 (dd, *J* = 8.7, 0.7 Hz, 1H), 7.60 (d, *J* = 1.8 Hz, 1H), 7.58–7.53 (m, 2H), 7.05 (dd, *J* = 2.2, 0.7 Hz, 1H), 6.84 (d, *J* = 8.1 Hz, 1H), 6.45 (d, *J* = 2.2 Hz, 1H), 3.93 (s, 3H), 3.92 (s, 3H), 1.71 (s, 6H) ppm. ^13^C NMR (101 MHz, CDCl_3_) *δ* = 161.4, 157.8, 156.0, 150.3, 148.6, 148.2, 133.7, 131.3, 121.2, 118.4, 115.6, 115.2, 108.3, 107.8, 99.9, 97.6, 55.7, 55.6, 25.9 ppm. HRMS (ESI) *m*/*z* calcd for C_20_H_20_O_4_N [M + H^+^]^+^ 338.1387, found 338.1386.

##### 2-(3,4-Dihydroxyphenyl)quinoline-5,7-diol (7)

Quinoline 6 (140 mg, 0.41 mmol, 1.0 eq.) was dissolved in anhydrous CH_2_Cl_2_ (1.4 mL). The solution was cooled to −78 °C in an acetone/anhydrous ice bath, and a 1 M solution of BBr_3_ in CH_2_Cl_2_ (5.1 mL, 5.10 mmol, 12.5 eq.) was added dropwise under a nitrogen atmosphere. The reaction mixture was allowed to stir for 15 minutes at −78 °C, followed by 16 hours at room temperature until the starting material was consumed (UPLC-MS). The reaction mixture was poured into a mixture of ice/water (20 mL). The solvents were evaporated, and the residue was purified by preparative HPLC to afford the desired final product 7 (67 mg, 61%). ^1^H NMR (401 MHz, DMSO-*d*_6_) *δ* = 11.61 (s, 1H), 11.33 (s, 1H), 10.24 (s, 1H), 9.61 (s, 1H), 8.89–8.64 (m, 1H), 7.79 (d, *J* = 8.7 Hz, 1H), 7.53 (d, *J* = 2.3 Hz, 1H), 7.47 (dd, *J* = 8.4, 2.4 Hz, 1H), 7.08–7.00 (m, 2H), 6.70 (d, *J* = 2.1 Hz, 1H) ppm. ^13^C NMR (101 MHz, DMSO-*d*_6_) *δ* = 164.6, 158.9, 158.5, 156.5, 154.4, 150.6, 146.6, 139.6, 121.6, 116.7, 116.1, 115.3, 114.8, 102.6, 95.2 ppm. HRMS (ESI) *m*/*z* calcd for C_15_H_12_O_4_N [M + H^+^]^+^ 270.0761, found 270.0760.

##### 3-Methoxynaphthalen-1-ol (9)

Naphthalene-1,3-diol (8) (1.0 g, 6.25 mmol, 1.0 eq.) was dissolved in anhydrous MeOH (20 mL). The solution was cooled to 0 °C and a 4 M solution of hydrogen chloride in 1,4-dioxane (7 mL, 28.1 mmol, 4.5 eq.) was added to the solution under a nitrogen atmosphere. The reaction mixture was allowed to stir for 16 hours at room temperature until the starting material was consumed (TLC, UPLC-MS). The solvents were evaporated, and oily residues were dissolved in EtOAc (30 mL) and washed with water (3 × 20 mL) and brine (1 × 20 mL). The organic phases were dried over anhydrous MgSO_4_. The solvents were evaporated and crude product 9 was used in the next step without further purification (705 mg, 65%). ^1^H NMR (401 MHz, CDCl_3_) *δ* = 8.15–8.07 (m, 1H), 7.72–7.68 (m, 1H), 7.46 (ddd, *J* = 8.2, 6.8, 1.3 Hz, 1H), 7.34 (ddd, *J* = 8.2, 6.8, 1.2 Hz, 1H), 6.78 (d, *J* = 2.3 Hz, 1H), 6.55 (d, *J* = 2.3 Hz, 1H), 3.89 (s, 3H) ppm. ^13^C NMR (101 MHz, CDCl_3_) *δ* = 157.9, 152.9, 135.6, 127.2, 126.8, 123.1, 121.8, 120.7, 101.5, 99.0, 55.5 ppm. LRMS (ESI) *m*/*z* calcd for C_11_H_11_O_2_ [M + H^+^]^+^ 175.08, found 175.12.

##### *tert*-Butyl((3-methoxynaphthalen-1-yl)oxy)dimethylsilane (10)

3-Methoxynaphthalen-1-ol (9) (705 mg, 4.05 mmol, 1.0 eq.) was dissolved in THF (14 mL), and imidazole (1.38 g, 20.2 mmolm 5.0 eq.) was added. The mixture was cooled to 0 °C, followed by addition of *tert*-butyldimethylsilyl chloride (1.52 g, 10.1 mmol, 2.5 eq.). The reaction mixture was allowed to stir for 2 days at room temperature under a nitrogen atmosphere until the starting material was consumed (TLC, UPLC-MS). A mixture of cyclohexane/EtOAc (1 : 1, 30 mL) was added, and the organic phase was washed with water (1 × 20 mL) and brine (1 × 20 mL). The aqueous phase was extracted with EtOAc (3 × 30 mL). The combined organic phases were dried over anhydrous MgSO_4_. The solvents were evaporated, and the residue was purified by flash chromatography (SiO_2_, *n*-hexane/EtOAc = 100 : 0 → 80 : 20) to afford the desired compound 10 (529 mg, 45%). ^1^H NMR (401 MHz, CDCl_3_) *δ* = 8.08 (dt, *J* = 8.4, 0.7 Hz, 1H), 7.69 (dt, *J* = 8.2, 0.6 Hz, 1H), 7.43 (ddd, *J* = 8.2, 6.8, 1.3 Hz, 1H), 7.31 (ddd, *J* = 8.2, 6.8, 1.2 Hz, 1H), 6.79 (d, *J* = 2.3 Hz, 1H), 6.56 (d, *J* = 2.3 Hz, 1H), 3.90 (s, 3H), 1.10 (s, 9H), 0.30 (s, 6H) ppm. ^13^C NMR (101 MHz, CDCl_3_) *δ* = 157.9, 152.8, 135.4, 126.8, 126.6, 124.0, 122.9, 122.6, 105.5, 99.1, 55.3, 25.9, 18.5, −4.3 ppm. LRMS (ESI) *m*/*z* calcd for C_17_H_25_O_2_Si [M + H^+^]^+^ 289.16, found 289.05.

##### Miyaura–Ishiyama–Hartwig borylation of 9

A tube with 9 (250 mg, 0.870 mmol, 1.0 eq.), bis(pinacolato)diboron (440 mg, 1.73 mmol, 2.0 eq.), 4,4′-di-*tert*-butyl-2,2′-dipyridyl (14 mg, 0.052 mmol, 0.06 eq.), and bis(1,5-cyclooctadiene)di-μ-methoxydiiridium(i) (23 mg, 0.035 mmol, 0.04 eq.) was sealed, and *n*-hexane (4.8 mL) was added *via* septum. The mixture was degassed by a stream of argon for 15 minutes and heated to 100 °C for 16 hours. Then, the reaction mixture was cooled to room temperature, quenched by the addition of water (2.0 mL) and extracted with EtOAc (3 × 20 mL). The combined organic phases were dried over anhydrous MgSO_4_. The solvents were evaporated and the residue was purified by flash chromatography (SiO_2_, *n*-hexane/EtOAc = 100 : 0 → 90 : 10) to afford an inseparable mixture of *tert*-butyl((3-methoxy-6-(4,4,5,5-tetramethyl-1,3,2-dioxaborolan-2-yl)naphthalen-1-yl)oxy)dimethylsilane (11a) and *tert*-butyl((3-methoxy-7-(4,4,5,5-tetramethyl-1,3,2-dioxaborolan-2-yl)naphthalen-1-yl)oxy)dimethylsilane (11b) (345 mg, 11a/11b = 63 : 37, 96%). Characterization was performed for the mixture of isomers 11a and 11b. ^1^H NMR (401 MHz, CDCl_3_) *δ* = 8.64 (dd, *J* = 1.3, 0.7 Hz, 0.47H), 8.22 (t, *J* = 0.9 Hz, 1.00H), 8.10–8.07 (m, 0.43H), 8.05 (d, *J* = 8.3 Hz, 1.00H), 7.77 (dd, *J* = 8.2, 1.2 Hz, 0.41H), 7.68 (dd, *J* = 8.3, 1.2 Hz, 1.13H), 7.67–7.63 (m, 0.61H), 7.42 (ddd, *J* = 8.2, 6.8, 1.3 Hz, 0.41H), 7.31 (ddd, *J* = 8.2, 6.8, 1.2 Hz, 0.41H), 6.87–6.83 (m, 1.00H), 6.80–6.76 (m, 0.92H), 6.59 (d, *J* = 2.3 Hz, 0.99H), 6.55 (dd, *J* = 5.1, 2.3 Hz, 0.94H), 3.90 (s, 2.69H), 3.88 (s, 3.10H), 1.38 (s, 12.00H), 1.36 (s, 6.58H), 1.12 (s, 4.75H), 1.08 (s, 9.35H), 0.30 (s, 2.84H), 0.28 (s, 6.06H) ppm. ^13^C NMR (101 MHz, CDCl_3_) *δ* = 171.3, 159.0, 158.0, 157.8, 153.6, 152.9, 152.8, 137.3, 135.5, 135.0, 134.8, 131.5, 131.4, 127.8, 126.9, 126.7, 125.8 (2C), 124.1, 123.6, 123.0, 122.7, 121.8, 106.9, 105.9, 105.6, 99.9, 99.3, 99.2, 84.0, 83.7, 60.5, 55.4, 26.0 (4C), 25.1 (4C), 21.2, 18.6 (2C), 14.3, −4.2 (2C) ppm.

##### 6-(3,4-Dihydroxyphenyl)naphthalene-1,3-diol (12)

A tube with a mixture of boronates 11a/11b (ratio: 63 : 37, 345 mg, 0.83 mmol, 1.5 eq.), 4-bromoveratrole (120 mg, 0.55 mmol, 1.0 eq.), K_2_CO_3_ (307 mg, 2.22 mmol, 4.0 eq.) and SPhos Pd G2 (40 mg, 0.055 mmol, 0.1 eq.) was sealed, and THF/water (25 : 1, 5.8 mL) was added *via* septum. The mixture was degassed by a stream of argon for 15 minutes, followed by heating to 100 °C for 16 hours until the starting material was consumed (TLC, UPLC-MS). Then, the reaction mixture was cooled to room temperature, filtered through Celite, and washed with EtOAc. The solvents were evaporated and the residue was purified by flash chromatography (SiO_2_, *n*-hexane/EtOAc = 100 : 0 → 80 : 20) to afford a mixture of *tert*-butyl((6-(3,4-dimethoxyphenyl)-3-methoxynaphthalen-1-yl)oxy)dimethylsilane and *tert*-butyl((7-(3,4-dimethoxyphenyl)-3-methoxynaphthalen-1-yl)oxy)dimethylsilane (194 mg, 82%). The mixture of regioisomers (194 mg, 0.45 mmol, 1.0 eq.) was dissolved in anhydrous THF (1.6 mL). The solution was cooled to 0 °C in an ice water bath, followed by addition of a 1 M solution of tetrabutylammonium fluoride in THF (0.68 mL, 0.68 mmol, 1.5 eq.). The reaction mixture was allowed to stir for 2 hours at 0 °C until the starting material was consumed (UPLC-MS). Then, the solvents were evaporated and the crude product was used in the next step without further purification. The residue was dissolved in anhydrous CH_2_Cl_2_ (0.7 mL). The solution was cooled to 0 °C in an ice water bath, and a 1 M solution of BBr_3_ in CH_2_Cl_2_ (2.1 mL, 2.1 mmol, 4.7 eq.) was added dropwise under a nitrogen atmosphere. Then, the reaction mixture was allowed to stir for 15 minutes at 0 °C, followed by 2 hours at room temperature until the starting material was consumed (UPLC-MS). The reaction mixture was poured into a mixture of ice/water (20 mL). After 30 minutes of stirring, the mixture was lyophilized, and the residue was twice purified by preparative HPLC to afford the desired final product 12 (8 mg, 15%). ^1^H NMR (500 MHz, DMSO-*d*_6_) *δ* = 10.03 (s, 1H), 9.45 (s, 1H), 9.05 (s, 1H), 9.00 (s, 1H), 7.95 (d, *J* = 8.7 Hz, 1H), 7.66 (d, *J* = 1.8 Hz, 1H), 7.35 (dd, *J* = 8.7, 1.9 Hz, 1H), 7.12 (d, *J* = 2.2 Hz, 1H), 7.02 (dd, *J* = 8.2, 2.3 Hz, 1H), 6.82 (d, *J* = 8.2 Hz, 1H), 6.62 (d, *J* = 2.1 Hz, 1H), 6.45 (d, *J* = 2.1 Hz, 1H) ppm. ^13^C NMR (126 MHz, DMSO-*d*_6_) *δ* = 156.2, 154.3, 145.6, 145.2, 138.2, 135.9, 131.7, 122.5, 122.1, 120.1, 118.2, 117.8, 116.0, 114.1, 100.6, 100.4 ppm. HRMS (ESI) *m*/*z* calcd for C_16_H_11_O_4_ [M–H^+^]^−^ 267.0663, found 267.0663.

##### 1,6-Dimethoxynaphthalene (14)

Naphthalene-1,6-diol (13) (2.00 g, 12.5 mmol, 1.0 eq.) was dissolved in anhydrous DMF (20 mL), followed by addition of K_2_CO_3_ (17.3 g, 125 mmol, 10 eq.) and iodomethane (7.8 mL, 125 mmol, 10 eq.). The reaction mixture was allowed to stir at room temperature overnight until the starting material was consumed (TLC, UPLC-MS). Then, EtOAc (30 mL) was added, and the organic phase was washed with water (3 × 50 mL) and brine (1 × 10 mL) and dried over anhydrous MgSO_4_. The solvents were evaporated, and the residue was purified by flash chromatography (SiO_2_, *n*-hexane/EtOAc = 100 : 0 → 80 : 20) to afford the desired compound 14 (2.03 g, 86%). ^1^H NMR (401 MHz, CDCl_3_) *δ* = 8.22 (d, *J* = 9.1 Hz, 1H), 7.43–7.32 (m, 2H), 7.20–7.12 (m, 2H), 6.71 (dd, *J* = 6.8, 1.9 Hz, 1H), 4.00 (s, 3H), 3.94 (s, 3H) ppm. ^13^C NMR (101 MHz, CDCl_3_) *δ* = 158.2, 155.8, 136.0, 126.8, 123.8, 120.9, 119.3, 117.6, 105.8, 102.1, 55.5, 55.3 ppm. HRMS (ESI) *m*/*z* calcd for C_20_H_21_O_4_ [M + H^+^]^+^ 325.1434, found 325.1433.

##### 2-(4,7-Dimethoxynaphthalen-2-yl)-4,4,5,5-tetramethyl-1,3,2-dioxaborolane (15)

A tube with 1,6-dimethoxynaphthalene (14) (376 mg, 2.00 mmol, 1.0 eq.), bis(pinacolato)diboron (1.02 g, 4.00 mmol, 2.0 eq.), 4,4′-di-*tert*-butyl-2,2′-dipyridyl (32 mg, 0.12 mmol, 0.06 eq.), and bis(1,5-cyclooctadiene)di-μ-methoxydiiridium(i) (53 mg, 0.08 mmol, 0.04 eq.) was sealed, and *n*-hexane (6.0 mL) was added *via* septum. The mixture was degassed by a stream of argon for 15 minutes and heated to 100 °C for 72 hours. Then, the reaction mixture was cooled to room temperature, quenched by the addition of water (2.0 mL) and extracted with EtOAc (3 × 20 mL). The combined organic phases were dried over anhydrous MgSO_4_. The solvents were evaporated, and the residue was purified by flash chromatography (SiO_2_, *n*-hexane/EtOAc = 100 : 0 → 90 : 10) to afford the desired boronate 15 (329 mg, 52%). ^1^H NMR (401 MHz, CDCl_3_) *δ* = 8.16 (dd, *J* = 9.9, 0.8 Hz, 1H), 7.88 (s, 1H), 7.20–7.11 (m, 2H), 7.03 (d, *J* = 0.9 Hz, 1H), 4.03 (s, 3H), 3.90 (s, 3H), 1.39 (s, 12H) ppm. ^13^C NMR (101 MHz, CDCl_3_) *δ* = 158.1, 155.1, 135.5, 128.0, 123.8, 122.7, 118.8, 106.5, 105.9, 84.0, 55.7, 55.4, 25.1 ppm. HRMS (ESI) *m*/*z* calcd for C_18_H_23_O_4_BNa [M + Na^+^]^+^ 337.1582, found 337.1580.

##### 3-(3,4-Dimethoxyphenyl)-1,6-dimethoxynaphthalene (16)

A tube with boronate 15 (217 mg, 0.69 mmol, 1.5 eq.), 4-bromoveratrole (100 mg, 0.46 mmol, 1.0 eq.), K_2_CO_3_ (254 mg, 1.84 mmol, 4.0 eq.) and SPhos Pd G2 (33 mg, 0.046 mmol, 0.1 eq.) was sealed, and THF/water (25 : 1) (5.8 mL) was added *via* septum. The mixture was degassed by a stream of argon for 15 minutes, followed by heating to 100 °C for 16 hours until the starting material was consumed (TLC, UPLC-MS). Then, the reaction mixture was cooled to room temperature, filtered through Celite, and washed with EtOAc. The solvents were evaporated, and the residue was purified by flash chromatography (SiO_2_, *n*-hexane/EtOAc = 100 : 0 → 80 : 20) to afford the desired product 16 (81 mg, 54%). ^1^H NMR (401 MHz, CDCl_3_) *δ* = 8.18 (d, *J* = 9.0 Hz, 1H), 7.51–7.46 (m, 1H), 7.28–7.23 (m, 2H), 7.17–7.10 (m, 2H), 6.98 (d, *J* = 8.2 Hz, 1H), 6.91 (d, *J* = 1.5 Hz, 1H), 4.05 (s, 3H), 4.00 (s, 3H), 3.95 (s, 3H), 3.93 (s, 3H) ppm. ^13^C NMR (101 MHz, CDCl_3_) *δ* = 158.6, 156.0, 149.2, 148.8, 139.6, 136.1, 134.8, 123.7, 119.8 (2C), 117.4, 117.0, 111.5, 110.8, 106.0, 102.0, 56.1, 56.0, 55.6, 55.3 ppm. HRMS (APCI) *m*/*z* calcd for C_20_H_21_O_4_ [M + H^+^]^+^ 325.1434, found 325.1433.

##### 3-(3,4-Dihydroxyphenyl)naphthalene-1,6-diol (17)

3-(3,4-Dimethoxyphenyl)-1,6-dimethoxynaphthalene (16) (81 mg, 0.25 mmol, 1.0 eq.) was dissolved in anhydrous CH_2_Cl_2_ (10 mL). The solution was cooled to 0 °C in an ice water bath, and a 1 M solution of BBr_3_ in CH_2_Cl_2_ (2.5 mL, 2.5 mmol, 10 eq.) was added dropwise under a nitrogen atmosphere. Then the reaction mixture was allowed to stir for 15 minutes at 0 °C and then for 2 hours at room temperature until the starting material was consumed (UPLC-MS). The reaction mixture was poured into a mixture of ice/water (20 mL). After 30 minutes of stirring the aqueous phase was extracted with EtOAc (3 × 25 mL) and the combined organic phases were dried over anhydrous MgSO_4_. The solvents were evaporated, and the residue was purified by preparative HPLC to afford the desired final product 17 (39 mg, 58%). ^1^H NMR (401 MHz, DMSO-*d*_6_) *δ* = 9.96 (br s, 1H), 9.62 (br s, 1H), 9.02 (br s, 2H), 7.94 (d, *J* = 9.0 Hz, 1H), 7.25 (br s, 1H), 7.07 (dd, *J* = 7.0, 2.3 Hz, 2H), 6.96 (td, *J* = 9.2, 8.7, 2.3 Hz, 2H), 6.86–6.78 (m, 2H) ppm. ^13^C NMR (101 MHz, DMSO-*d*_6_) *δ* = 155.9, 153.7, 145.5, 145.1, 138.9, 136.5, 132.1, 123.6, 118.0, 117.8, 116.6, 116.1, 114.1, 113.6, 108.8, 104.3 ppm. HRMS (APCI) *m*/*z* calcd for C_16_H_13_O_4_ [M + H^+^]^+^ 269.0808, found 269.0808.

##### Methyl 4-acetoxy-5,7-dimethoxy-2-naphthoate (19)

A solution of 3,5-dimethoxybenzaldehyde (18) (4.00 g, 24.1 mmol, 1.0 eq.) and dimethyl succinate (3.8 mL, 28.9 mmol, 1.2 eq.) in anhydrous *tert*-butanol (12 mL) was added dropwise to a refluxing solution of potassium *tert*-butoxide (4.05 g, 36.1 mmol, 1.5 eq.) in anhydrous *tert*-butanol (24 mL) for 1 hour. Then, the reaction mixture was allowed to stir for 2 hours at 90 °C until the starting material was consumed (UPLC-MS). The solvents were evaporated, the residue was dissolved in 5% HCl (aq., 25 mL), and the aqueous solution was extracted with EtOAc (3 × 12 mL). The combined organic phases were washed with brine (1 × 10 mL) and dried over anhydrous MgSO_4_. The solvents were evaporated, a mixture of *n*-hexane/Et_2_O (9 : 1, 25 mL) was added into the oily residue, and the emulsion was vigorously stirred overnight. The crude product containing 4-(3,5-dimethoxyphenyl)-3-(methoxycarbonyl)but-3-enoic acid [(UPLC-ESI): *t*_R_ = 4.01 min, *m*/*z* calcd for C_14_H_15_O_6_ [M–H^+^]^−^ 279, found 279] was isolated by decantation of the resulting suspension and used in the next reaction without additional purification. The crude product was suspended in acetic anhydride (35 mL), followed by addition of potassium acetate (3.28 g, 33.5 mmol, 1.5 eq.). The reaction mixture was heated to 145 °C for 6 hours until the starting material was consumed (UPLC-MS, TLC). Then, the reaction mixture was cooled to room temperature, poured into ice (100 mL), and allowed to stir for 30 minutes. The resulting emulsion was extracted with Et_2_O (3 × 150 mL), and the combined organic phases were washed with brine (1 × 20 mL) and dried over anhydrous MgSO_4_. The solvents were evaporated, and the residue was pre-purified by flash chromatography (SiO_2_, cyclohexane/EtOAc = 100 : 0 → 50 : 50). The combined fractions containing product were evaporated and purified by recrystallization in EtOH (20 mL) to afford the desired product 19 (1.73 g, 24% over two steps). ^1^H NMR (401 MHz, CDCl_3_) *δ* = 8.31 (d, *J* = 1.7 Hz, 1H), 7.49 (d, *J* = 1.7 Hz, 1H), 6.85 (d, *J* = 2.2 Hz, 1H), 6.59 (d, *J* = 2.2 Hz, 1H), 3.94 (s, 3H), 3.90 (s, 6H), 2.36 (s, 3H) ppm. ^13^C NMR (101 MHz, CDCl_3_) *δ* = 170.0, 166.3, 158.7, 156.1, 146.8, 137.0, 128.3, 127.9, 117.2, 116.6, 101.6, 100.1, 56.1, 55.4, 52.3, 20.9 ppm. LRMS (ESI) *m*/*z* calcd for C_16_H_17_O_6_ [M + H^+^]^+^ 305.10, found 305.07.

##### 4-Hydroxy-5,7-dimethoxy-2-naphthoic acid (20)

Methyl 4-acetoxy-5,7-dimethoxy-2-naphthoate (19) (1.73 g, 5.69 mmol, 1.0 eq.) was suspended in 2.5 M NaOH (aq., 34 mL, 85.4 mmol, 15 eq.). The reaction mixture was heated to 100 °C under a nitrogen atmosphere for 2 hours until the starting material was consumed (UPLC-MS). Then, the reaction mixture was cooled to room temperature followed by addition of a concentrated aqueous solution of HCl until pH = 1. The solids were filtered (S3) and purified by recrystallization in EtOH (10 mL) to afford the desired acid 20 (1.31 g, 93%). ^1^H NMR (401 MHz, DMSO-*d*_6_) *δ* = 12.90 (br s, 1H), 9.37 (s, 1H), 7.87 (d, *J* = 1.6 Hz, 1H), 7.07–7.02 (m, 2H), 6.69 (d, *J* = 2.3 Hz, 1H), 3.99 (s, 3H), 3.86 (s, 3H) ppm. ^13^C NMR (101 MHz, DMSO-*d*_6_) *δ* = 67.3, 158.2, 157.0, 154.4, 136.6, 129.9, 120.0, 112.5, 107.2, 100.5, 99.7, 56.4, 55.4 ppm. HRMS (ESI) *m*/*z* calcd for C_13_H_11_O_5_ [M–H^+^]^−^ 247.0612, found 247.0612.

##### 4,5,7-Trimethoxy-2-naphthoic acid (21)

4-Hydroxy-5,7-dimethoxy-2-naphthoic acid (20) (1.37 g, 5.50 mmol, 1.0 eq.) was dissolved in DMF (35 mL), followed by addition of benzyltriethylammonium chloride (125 mg, 0.35 mmol, 0.1 eq.), sodium hydride (60 wt% in mineral oil, 1.65 g, 41.3 mmol, 7.5 eq.) and iodomethane (1.7 mL, 27.5 mmol, 5.0 eq.). The open reaction mixture was allowed to stir at room temperature for 3 days until the starting material was consumed (UPLC-MS). Concentrated hydrochloric acid was slowly added until pH = 1–2, followed by addition of EtOAc (25 mL). The organic phase was washed with water (3 × 75 mL) and brine (1 × 10 mL) and dried over anhydrous MgSO_4_. The solvents were evaporated and the crude product was purified by recrystallization in EtOH to afford the desired acid 21 (954 mg, 66%). ^1^H NMR (401 MHz, C_5_D_5_N) *δ* = 8.61 (d, *J* = 1.5 Hz, 1H), 7.81 (d, *J* = 1.5 Hz, 1H), 7.07 (d, *J* = 2.4 Hz, 1H), 6.82 (d, *J* = 2.3 Hz, 1H), 3.93 (s, 3H), 3.86 (s, 3H), 3.84 (s, 3H) ppm. ^13^C NMR (101 MHz, C_5_D_5_N) *δ* = 169.8, 159.7, 159.5, 158.8, 138.7, 131.5, 123.8, 116.2, 104.6, 101.7, 101.0, 56.6, 56.5, 55.8 ppm. HRMS (ESI) *m*/*z* calcd for C_14_H_13_O_5_ [M–H^+^]^−^ 261.0769, found 261.0768.

##### 4,4,5,5-Tetramethyl-2-(4,5,7-trimethoxynaphthalen-2-yl)-1,3,2-dioxaborolane (22)

A tube with 4,5,7-trimethoxy-2-naphthoic acid (21) (262 mg, 1.00 mmol, 1.00 eq.), bis(pinacolato)diboron (381 mg, 1.50 mmol, 1.50 eq.), palladium acetate (12 mg, 0.05 mmol, 0.05 eq.), 1,4-bis(diphenylphosphino)butane (dppb, 43 mg, 0.10 mmol, 0.10 eq.), triethylamine (210 μL, 1.50 mmol, 1.50 eq.), and trimethylacetic anhydride (305 μL, 1.50 mmol, 1.50 eq.) was placed under a positive pressure of argon and subjected to three evacuation/backfilling cycles under high vacuum. Anhydrous 1,4-dioxane (5.0 mL) was added *via* septum, followed by heating to 160 °C for 15 hours until the starting material was consumed (TLC, UPLC-MS). Then, the reaction mixture was cooled to room temperature, diluted with CH_2_Cl_2_ (10 mL), and filtered through a syringe filter. The solvents were evaporated, and the residue was purified by flash chromatography (SiO_2_, cyclohexane/EtOAc = 100 : 0 → 70 : 30) to afford the desired product 22 (188 mg, 55%). ^1^H NMR (401 MHz, CDCl_3_) *δ* = 7.81 (d, *J* = 1.0 Hz, 1H), 7.04 (d, *J* = 1.0 Hz, 1H), 6.77 (d, *J* = 2.4 Hz, 1H), 6.54 (d, *J* = 2.4 Hz, 1H), 4.00 (s, 3H), 3.94 (s, 3H), 3.87 (s, 3H), 1.38 (s, 12H) ppm. ^13^C NMR (101 MHz, CDCl_3_) *δ* = 185.1, 158.3, 158.0, 156.7, 137.7, 128.5, 115.0, 108.3, 100.0, 99.6, 56.5 (2C), 55.3, 38.7, 27.1 ppm. HRMS (EI) *m*/*z* calcd for C_19_H_25_BO_5_ [M]˙^+^ 344.1790, found 344.1802.

##### 2,2-Dimethyl-5-(4,5,7-trimethoxynaphthalen-2-yl)benzo[*d*][1,3]dioxole (23)

A tube with boronate 22 (100 mg, 0.29 mmol, 1.0 eq.), 5-bromo-2,2-dimethylbenzo[*d*][1,3]dioxole (80 mg, 0.35 mmol, 1.2 eq.), K_2_CO_3_ (120 mg, 0.87 mmol, 3.0 eq.) and SPhos Pd G2 (21 mg, 0.029 mmol, 0.1 eq.) was sealed, and THF/water (25 : 1, 3.0 mL) was added *via* septum. The mixture was degassed by a stream of argon for 15 minutes, followed by heating to 100 °C for 2 hours until the starting material was consumed (TLC, UPLC-MS). Then, the reaction mixture was cooled to room temperature, filtered through Celite, and washed with EtOAc. The solvents were evaporated, and the residue was purified by flash chromatography (SiO_2_, cyclohexane/EtOAc = 100 : 0 → 80 : 20) to afford the desired product 23 (56 mg, 67%). ^1^H NMR (401 MHz, CDCl_3_) *δ* = 7.40 (d, *J* = 1.6 Hz, 1H), 7.14 (dd, *J* = 8.0, 1.8 Hz, 1H), 7.10 (d, *J* = 1.8 Hz, 1H), 6.88 (d, *J* = 1.6 Hz, 1H), 6.83 (d, *J* = 8.0 Hz, 1H), 6.74 (d, *J* = 2.3 Hz, 1H), 6.49 (d, *J* = 2.3 Hz, 1H), 4.01 (s, 3H), 3.96 (s, 3H), 3.91 (s, 3H), 1.73 (s, 6H) ppm. ^13^C NMR (101 MHz, CDCl_3_) *δ* = 158.6, 158.4, 157.7, 148.1, 147.3, 139.9, 138.5, 135.0, 120.3, 118.3, 117.7, 108.5, 107.7, 104.1, 99.2, 98.8, 60.5, 56.5, 56.4, 55.4, 26.0 ppm. HRMS (ESI) *m*/*z* calcd for C_22_H_23_O_5_ [M + H^+^]^+^ 367.1540, found 367.1538.

##### 6-(3,4-Dihydroxyphenyl)naphthalene-1,3,8-triol (carboluteolin) (24)

Compound 23 (56 mg, 0.15 mmol, 1.0 eq.) was dissolved in anhydrous CH_2_Cl_2_ (0.5 mL). The solution was cooled to 0 °C in an ice bath, and a 1 M solution of BBr_3_ in CH_2_Cl_2_ (2.4 mL, 2.40 mmol, 15.6 eq.) was added dropwise under a nitrogen atmosphere. The reaction mixture was allowed to stir for 15 minutes at 0 °C, followed by 1 hour at room temperature until the starting material was consumed (UPLC-MS). The reaction mixture was poured into a mixture of ice/water (10 mL). The solvents were evaporated, and the residue was purified by preparative HPLC to afford the desired final product 24 (25 mg, 60%). ^1^H NMR (500 MHz, DMSO-*d*_6_) *δ* = 10.69 (s, 1H), 10.67 (s, 1H), 9.54 (br s, 1H), 9.03 (br s, 2H), 7.16 (d, *J* = 1.7 Hz, 1H), 7.05 (d, *J* = 2.2 Hz, 1H), 6.96 (dd, *J* = 8.2, 2.2 Hz, 1H), 6.80 (d, *J* = 8.2 Hz, 1H), 6.65 (d, *J* = 1.7 Hz, 1H), 6.57 (d, *J* = 2.2 Hz, 1H), 6.28 (d, *J* = 2.2 Hz, 1H) ppm. ^13^C NMR (126 MHz, DMSO-*d*_6_) *δ* = 156.5, 155.2, 154.4, 145.5, 145.3, 139.1, 138.0, 131.4, 117.7, 116.0, 114.0, 113.9, 108.0, 104.1, 101.2, 100.5 ppm. IR (KBr): *ν̃* = 3414(s), 3247(m), 1639(s), 1607(s), 1522(m), 1487(m), 1380(m), 1283(m), 1192(m) cm^−1^. HRMS (ESI) *m*/*z* calcd for C_16_H_11_O_5_ [M–H^+^]^−^ 283.0612, found 283.0609.

##### 2,3-Dimethoxy-5-(4,5,7-trimethoxynaphthalen-2-yl)pyridine (25)

Boronate 22 (150 mg, 0.44 mmol, 1.5 eq.), 5-bromo-2,3-dimethoxypyridine (63 mg, 0.29 mmol, 1.0 eq.) and K_2_CO_3_ (80 mg, 0.58 mmol, 2.0 eq.) were dissolved in 1,4-dioxane/water (3 : 1, 2.7 mL) in a tube. The mixture was degassed by a stream of argon for 15 minutes, followed by addition of Pd(PPh_3_)_4_ (35 mg, 0.03 mmol, 0.1 eq.). The tube was sealed and heated to 100 °C for 16 hours until the starting material was consumed (TLC, UPLC-MS). Then, the reaction mixture was cooled to room temperature, filtered through Celite, and washed with EtOAc. The solvents were evaporated, and the residue was purified by flash chromatography (SiO_2_, cyclohexane/EtOAc = 100 : 0 → 70 : 30) to afford the desired product 25 (101 mg, 98%). ^1^H NMR (401 MHz, CDCl_3_) *δ* = 8.05 (d, *J* = 2.0 Hz, 1H), 7.40 (d, *J* = 1.7 Hz, 1H), 7.34 (d, *J* = 2.0 Hz, 1H), 6.85 (d, *J* = 1.6 Hz, 1H), 6.75 (d, *J* = 2.4 Hz, 1H), 6.51 (d, *J* = 2.3 Hz, 1H), 4.08 (s, 3H), 4.01 (s, 3H), 3.97 (s, 3H), 3.95 (s, 3H), 3.91 (s, 3H) ppm. ^13^C NMR (101 MHz, CDCl_3_) *δ* = 158.7, 158.4, 158.0, 154.1, 144.1, 138.5, 136.8, 135.4, 130.9, 117.9, 116.6, 112.5, 103.7, 99.1 (2C), 56.6, 56.4, 55.9, 55.4, 54.0 ppm. HRMS (ESI) *m*/*z* calcd for C_20_H_21_O_5_NNa [M + Na^+^]^+^ 378.1312, found 378.1310.

##### 2,3-Dimethoxy-6-(4,5,7-trimethoxynaphthalen-2-yl)pyridine (26)

Boronate 22 (285 mg, 0.83 mmol, 1.3 eq.), 6-bromo-2,3-dimethoxypyridine (139 mg, 0.64 mmol, 1.0 eq.) and K_2_CO_3_ (177 mg, 1.28 mmol, 2.0 eq.) were dissolved in 1,4-dioxane/water (3 : 1, 6.0 mL) in a tube. The mixture was degassed by a stream of argon for 15 minutes, followed by addition of Pd(PPh_3_)_4_ (74 mg, 0.064 mmol, 0.1 eq.). The tube was sealed and heated to 100 °C for 16 hours until the starting material was consumed (TLC, UPLC-MS). Then, the reaction mixture was cooled to room temperature, filtered through Celite, and washed with EtOAc. The solvents were evaporated, and the residue was purified by flash chromatography (SiO_2_, cyclohexane/EtOAc = 100 : 0 → 70 : 30) to afford the desired product 26 (83 mg, 37%). ^1^H NMR (401 MHz, CDCl_3_) *δ* = 7.86 (d, *J* = 1.6 Hz, 1H), 7.43 (d, *J* = 1.6 Hz, 1H), 7.41 (d, *J* = 8.0 Hz, 1H), 7.09 (d, *J* = 8.1 Hz, 1H), 6.79 (d, *J* = 2.3 Hz, 1H), 6.50 (d, *J* = 2.3 Hz, 1H), 4.17 (s, 3H), 4.04 (s, 3H), 3.95 (s, 3H), 3.90 (s, 3H), 3.89 (s, 3H) ppm. ^13^C NMR (101 MHz, CDCl_3_) *δ* = 158.4, 158.3, 157.6, 153.6, 144.9, 143.5, 138.4, 137.3, 117.8, 117.3, 113.3, 112.9, 102.7, 99.5, 99.0, 56.4, 56.3, 55.9, 55.3, 53.6 ppm. HRMS (ESI) *m*/*z* calcd for C_20_H_22_O_5_N [M + H^+^]^+^ 356.1493, found 356.1496.

##### 4,5-Dimethoxy-2-(4,5,7-trimethoxynaphthalen-2-yl)pyridine (27)

Boronate 22 (100 mg, 0.29 mmol, 1.5 eq.), 2-bromo-4,5-dimethoxypyridine (42 mg, 0.19 mmol, 1.0 eq.) and K_2_CO_3_ (53 mg, 0.38 mmol, 2.0 eq.) were dissolved in 1,4-dioxane/water (3 : 1, 2.0 mL) in a tube. The mixture was degassed by a stream of argon for 15 minutes, followed by addition of Pd(PPh_3_)_4_ (34 mg, 0.029 mmol, 0.1 eq.). The tube was sealed and heated to 100 °C for 16 hours until the starting material was consumed (TLC, UPLC-MS). Then, the reaction mixture was cooled to room temperature, filtered through Celite, and washed with EtOAc. The solvents were evaporated, and the residue was purified by flash chromatography (SiO_2_, cyclohexane/EtOAc = 100 : 0 → 40 : 60) to afford the desired product 27 (53 mg, 51%). ^1^H NMR (401 MHz, CDCl_3_) *δ* = 8.26 (s, 1H), 7.72 (d, *J* = 1.5 Hz, 1H), 7.40 (d, *J* = 1.5 Hz, 1H), 7.34 (s, 1H), 6.80 (d, *J* = 2.3 Hz, 1H), 6.52 (d, *J* = 2.3 Hz, 1H), 4.06 (s, 3H), 4.03 (s, 3H), 4.00 (s, 3H), 3.95 (s, 3H), 3.90 (s, 3H) ppm. ^13^C NMR (101 MHz, CDCl_3_) *δ* = 158.5, 158.4, 157.9, 155.7, 152.2, 145.2, 138.3, 138.1, 133.0, 117.8, 113.2, 103.9, 103.0, 99.5, 99.3, 56.8, 56.6, 56.4, 55.9, 55.4 ppm. HRMS (ESI) *m*/*z* calcd for C_20_H_22_O_5_N [M + H^+^]^+^ 356.1493, found 356.1489.

##### 2,3-Dimethoxy-5-(4,5,7-trimethoxynaphthalen-2-yl)pyrazine (28)

Boronate 22 (100 mg, 0.29 mmol, 1.5 eq.), 5-bromo-2,3-dimethoxypyrazine (48 mg, 0.19 mmol, 1.0 eq.) and K_2_CO_3_ (61 mg, 0.44 mmol, 2.0 eq.) were dissolved in 1,4-dioxane/water (3 : 1, 2.0 mL) in a tube. The mixture was degassed by a stream of argon for 15 minutes, followed by addition of Pd(PPh_3_)_4_ (26 mg, 0.022 mmol, 0.1 eq.). The tube was sealed and heated to 100 °C for 16 hours until the starting material was consumed (TLC, UPLC-MS). Then, the reaction mixture was cooled to room temperature, filtered through Celite, and washed with EtOAc. The solvents were evaporated, and the residue was purified by flash chromatography (SiO_2_, cyclohexane/EtOAc = 100 : 0 → 60 : 40) to afford the desired product 28 (51 mg, 65%). ^1^H NMR (401 MHz, CDCl_3_) *δ* = 8.19 (s, 1H), 7.83 (d, *J* = 1.6 Hz, 1H), 7.30 (d, *J* = 1.6 Hz, 1H), 6.79 (d, *J* = 2.3 Hz, 1H), 6.51 (d, *J* = 2.3 Hz, 1H), 4.17 (s, 3H), 4.08 (s, 3H), 4.03 (s, 3H), 3.96 (s, 3H), 3.92 (s, 3H) ppm. ^13^C NMR (101 MHz, CDCl_3_) *δ* = 158.6, 158.4, 157.9, 149.7, 149.5, 141.1, 138.4, 135.1, 128.6, 117.4, 114.7, 113.2, 102.3, 99.6, 99.4, 56.5, 56.4, 55.4, 54.3, 53.9 ppm. HRMS (ESI) *m*/*z* calcd for C_19_H_20_O_5_N_2_Na [M + Na^+^]^+^ 379.1264, found 379.1267.

##### 3,4-Dimethoxy-6-(4,5,7-trimethoxynaphthalen-2-yl)pyridazine (29)

Boronate 22 (100 mg, 0.29 mmol, 1.3 eq.), 6-chloro-3,4-dimethoxypyridazine (39 mg, 0.22 mmol, 1.0 eq.) and K_2_CO_3_ (61 mg, 0.44 mmol, 2.0 eq.) were dissolved in 1,4-dioxane/water (3 : 1, 2.0 mL) in a tube. The mixture was degassed by a stream of argon for 15 minutes, followed by addition of Pd(PPh_3_)_4_ (26 mg, 0.022 mmol, 0.1 eq.). The tube was sealed and heated to 100 °C for 16 hours until the starting material was consumed (TLC, UPLC-MS). Then, the reaction mixture was cooled to room temperature, filtered through Celite, and washed with EtOAc. The solvents were evaporated, and the residue was purified by flash chromatography (SiO_2_-C_18_, H_2_O (0.1% TFA)/MeCN = 90 : 10 → 40 : 60) to afford the desired product 29 (41 mg, 53%). ^1^H NMR (401 MHz, C_5_D_5_N) *δ* = 8.27 (s, 1H), 8.10 (s, 1H), 7.74 (s, 1H), 6.93 (s, 1H), 6.79 (s, 1H), 4.25 (s, 3H), 4.00 (s, 3H), 3.93 (s, 3H), 3.88 (s, 3H), 3.81 (s, 3H) ppm. ^13^C NMR (101 MHz, C_5_D_5_N) *δ* = 159.7 (2C), 159.1, 157.9, 157.0, 149.8, 139.4, 136.8, 119.2, 114.6, 106.4, 103.5, 100.5 (2C), 56.6 (2C), 56.2, 55.8, 55.2 ppm. HRMS (ESI) *m*/*z* calcd for C_19_H_21_O_5_N_2_ [M + H^+^]^+^ 357.1445, found 357.1448.

##### 4,5-Dimethoxy-2-(4,5,7-trimethoxynaphthalen-2-yl)pyrimidine (30)

Boronate 22 (200 mg, 0.58 mmol, 1.3 eq.), 2-chloro-4,5-dimethoxypyrimidine (77 mg, 0.44 mmol, 1.0 eq.) and K_2_CO_3_ (122 mg, 0.88 mmol, 2.0 eq.) were dissolved in 1,4-dioxane/water (3 : 1, 4.0 mL) in a tube. The mixture was degassed by a stream of argon for 15 minutes, followed by addition of Pd(PPh_3_)_4_ (67 mg, 0.058 mmol, 0.1 eq.). The tube was sealed and heated to 100 °C for 16 hours until the starting material was consumed (TLC, UPLC-MS). Then, the reaction mixture was cooled to room temperature, filtered through Celite, and washed with EtOAc. The solvents were evaporated, and the residue was purified by flash chromatography (SiO_2_, cyclohexane/EtOAc = 100 : 0 → 40 : 60) to afford the desired product 30 (151 mg, 96%). ^1^H NMR (401 MHz, CDCl_3_) *δ* = 8.30 (d, *J* = 1.5 Hz, 1H), 8.16 (s, 1H), 7.73 (d, *J* = 1.6 Hz, 1H), 6.85 (d, *J* = 2.3 Hz, 1H), 6.54 (d, *J* = 2.3 Hz, 1H), 4.21 (s, 3H), 4.07 (s, 3H), 3.97 (s, 3H), 3.96 (s, 3H), 3.91 (s, 3H) ppm. ^13^C NMR (101 MHz, CDCl_3_) *δ* = 159.7, 158.4, 158.3, 157.6, 156.0, 141.3, 138.2, 137.0, 135.9, 119.8, 114.0, 102.9, 100.1, 99.8, 56.5, 56.4 (2C), 55.4, 54.2 ppm. HRMS (ESI) *m*/*z* calcd for C_19_H_21_O_5_N_2_ [M + H^+^]^+^ 357.1445, found 357.1444.

##### 3-Hydroxy-5-(4,5,7-trihydroxynaphthalen-2-yl)pyridin-2(1*H*)-one (31)

2,3-Dimethoxy-5-(4,5,7-trimethoxynaphthalen-2-yl)pyridine (25) (101 mg, 0.28 mmol, 1.0 eq.) was dissolved in anhydrous CH_2_Cl_2_ (1.0 mL) under a nitrogen atmosphere. The solution was cooled to 0 °C in an ice bath, and a 1 M solution of BBr_3_ in CH_2_Cl_2_ (5.1 mL, 5.10 mmol, 18 eq.) was added dropwise. Then, the reaction mixture was allowed to stir for 15 minutes at 0 °C, followed by 2 hours at room temperature until the starting material was consumed (UPLC-MS). The reaction mixture was poured into a mixture of ice/water (20 mL). The mixture was lyophilized, and the residue was purified by preparative HPLC to afford the desired final product 31 (16 mg, 20%). ^1^H NMR (500 MHz, DMSO-*d*_6_, 333 K) *δ* = 0.66 (br s, 1H), 10.52 (br s, 1H), 7.18–7.16 (m, 2H), 7.11 (d, *J* = 2.4 Hz, 1H), 6.60 (d, *J* = 1.7 Hz, 1H), 6.59 (d, *J* = 2.3 Hz, 1H), 6.32 (d, *J* = 2.2 Hz, 1H) ppm. ^13^C NMR (126 MHz, DMSO-*d*_6_, 333 K) *δ* = 157.5, 156.3, 154.9, 154.4, 146.9, 137.8, 135.1, 121.0, 118.3, 114.4, 113.3, 108.0, 103.0, 101.2, 100.6 ppm. IR (KBr): *ν̃* = 3422(s), 3093(m), 1652(sh), 1616(s), 1496(m), 1456(m), 1383(m), 1313(m), 1237(m), 1195(m) cm^−1^. HRMS (ESI) *m*/*z* calcd for C_15_H_10_O_5_N [M–H^+^]^−^ 284.0565, found 284.0562.

##### 3-Hydroxy-6-(4,5,7-trihydroxynaphthalen-2-yl)pyridin-2(1*H*)-one (32)

2,3-Dimethoxy-6-(4,5,7-trimethoxynaphthalen-2-yl)pyridine (26) (83 mg, 0.23 mmol, 1.0 eq.) was dissolved in anhydrous CH_2_Cl_2_ (4.2 mL) under a nitrogen atmosphere. The solution was cooled to 0 °C in an ice bath, and a 1 M solution of BBr_3_ in CH_2_Cl_2_ (2.1 mL, 2.10 mmol, 9.0 eq.) was added dropwise. Then, the reaction mixture was allowed to stir for 15 minutes at 0 °C, followed by 48 hours at room temperature until the starting material was consumed (UPLC-MS). The reaction mixture was poured into a mixture of ice/water (20 mL). The mixture was lyophilized, and the residue was purified by preparative HPLC to afford the desired final product 32 (36 mg, 53%). ^1^H NMR (401 MHz, DMSO-*d*_6_) *δ* = 11.81 (br s, 1H), 10.90 (s, 1H), 10.69 (s, 1H), 9.67 (br s, 1H), 9.19 (br s, 1H), 7.39 (d, *J* = 1.7 Hz, 1H), 6.80 (d, *J* = 7.6 Hz, 1H), 6.70 (d, *J* = 1.7 Hz, 1H), 6.59 (d, *J* = 2.2 Hz, 1H), 6.45 (d, *J* = 7.5 Hz, 1H), 6.36 (d, *J* = 2.2 Hz, 1H) ppm. ^13^C NMR (101 MHz, DMSO-*d*_6_) *δ* = 158.7, 156.7, 155.2, 154.6, 146.5, 137.5, 135.5, 132.1, 116.1, 114.7, 108.9, 104.1, 103.1, 101.8, 101.4 ppm. IR (KBr): *ν̃* = 3418(s), 3051(m), 1646(s), 1609(s), 1496(m), 1383(m), 1275(m), 1194(m), 1007(m) cm^−1^. HRMS (ESI) *m*/*z* calcd for C_15_H_11_O_5_NNa [M + Na^+^]^+^ 308.0529, found 308.0531.

##### 5-Hydroxy-2-(4,5,7-trihydroxynaphthalen-2-yl)pyridin-4(1*H*)-one (33)

4,5-Dimethoxy-2-(4,5,7-trimethoxynaphthalen-2-yl)pyridine (27) (53 mg, 0.15 mmol, 1.0 eq.) was dissolved in anhydrous CH_2_Cl_2_ (2.7 mL) under a nitrogen atmosphere. The solution was cooled to 0 °C in an ice bath, and a 1 M solution of BBr_3_ in CH_2_Cl_2_ (1.4 mL, 1.40 mmol, 9.0 eq.) was added dropwise. Then, the reaction mixture was allowed to stir for 15 minutes at 0 °C, followed by 72 hours at room temperature until the starting material was consumed (UPLC-MS). The reaction mixture was poured into a mixture of ice/water (20 mL). The mixture was lyophilized, and the residue was purified by preparative HPLC to afford the desired final product 33 (21 mg, 50%). ^1^H NMR (401 MHz, DMSO-*d*_6_) *δ* = 7.98 (s, 1H), 7.49 (s, 1H), 7.36 (s, 1H), 6.80 (s, 1H), 6.69 (s, 1H), 6.47 (s, 1H) ppm. ^13^C NMR (101 MHz, DMSO-*d*_6_) *δ* = 162.6, 157.7, 155.9, 155.8, 145.9, 145.2, 137.8, 131.2, 127.0, 116.9, 110.8, 110.1, 103.4, 102.8, 102.5 ppm. IR (KBr): *ν̃* = 3414(s), 3266(m), 1623(s), 1595(s), 1539(m), 1487(m), 1384(m), 1207(s) cm^−1^. HRMS (ESI) *m*/*z* calcd for C_15_H_10_O_5_N [M–H^+^]^−^ 284.0565, found 284.0560.

##### 3-Hydroxy-6-(4,5,7-trihydroxynaphthalen-2-yl)pyrazin-2(1*H*)-one (34)

2,3-Dimethoxy-5-(4,5,7-trimethoxynaphthalen-2-yl)pyrazine (28) (91 mg, 0.26 mmol, 1.0 eq.) was dissolved in anhydrous CH_2_Cl_2_ (5.4 mL) under a nitrogen atmosphere. The solution was cooled to 0 °C in an ice bath, and a 1 M solution of BBr_3_ in CH_2_Cl_2_ (2.7 mL, 2.70 mmol, 10.5 eq.) was added dropwise. Then, the reaction mixture was allowed to stir for 15 minutes at 0 °C, followed by 48 hours at room temperature. The reaction mixture was poured into a mixture of ice/water (20 mL). The mixture was lyophilized and dissolved in anhydrous 1,4-dioxane (5.4 mL) followed by addition of a 4 M solution of hydrogen chloride in 1,4-dioxane (5.4 mL, 21.6 mmol, 83.1 eq.). The reaction mixture was heated to 110 °C for 5 hours until the starting material was consumed (UPLC-MS). Then, the solvents were evaporated, and the residue was purified by preparative HPLC to afford the desired final product 34 (10 mg, 14%). ^1^H NMR (600 MHz, DMSO-*d*_6_) *δ* = 11.43 (br s, 2H), 9.64 (s, 1H), 7.28 (d, *J* = 1.7 Hz, 1H), 6.62 (br s, 1H), 6.59 (d, *J* = 1.8 Hz, 1H), 6.54 (d, *J* = 2.2 Hz, 1H), 6.33 (d, *J* = 2.2 Hz, 1H) ppm. ^13^C NMR (151 MHz, DMSO-*d*_6_) *δ* = 156.9, 156.7, 155.8, 155.2, 154.7, 137.4, 130.0, 121.8, 113.6, 108.7, 107.1, 102.3, 101.5, 101.1 ppm. IR (KBr): *ν̃* = 3442(s), 3211(m), 1641(sh), 1629(s), 1543(m), 1384(m), 1368(m), 1114(m) cm^−1^. HRMS (ESI) *m*/*z* calcd for C_14_H_10_O_5_N_2_Na [M + Na^+^]^+^ 309.0482, found 309.0481.

##### 4-Hydroxy-6-(4,5,7-trihydroxynaphthalen-2-yl)pyridazin-3(2*H*)-one (35)

3,4-Dimethoxy-6-(4,5,7-trimethoxynaphthalen-2-yl)pyridazine (28) (41 mg, 0.12 mmol, 1.0 eq.) was dissolved in anhydrous CH_2_Cl_2_ (8.0 mL) under a nitrogen atmosphere. The solution was cooled to 0 °C in an ice bath, and a 1 M solution of BBr_3_ in CH_2_Cl_2_ (3.1 mL, 3.1 mmol, 25.8 eq.) was added dropwise. Then, the reaction mixture was allowed to stir for 15 minutes at 0 °C, followed by 48 hours at room temperature. The reaction mixture was poured into a mixture of ice/water (20 mL). The mixture was lyophilized and dissolved in anhydrous 1,4-dioxane (0.58 mL), followed by addition of a 4 M solution of hydrogen chloride in 1,4-dioxane (0.6 mL, 2.4 mmol, 20 eq.). Then, the reaction mixture was heated to 110 °C for 5 hours until the starting material was consumed (UPLC-MS). The solvents were evaporated, and the residue was purified by preparative HPLC to afford the desired final product 35 (7.0 mg, 21%). ^1^H NMR (600 MHz, DMSO-*d*_6_) *δ* = 13.09 (s, 1H), 10.94 (br s, 1H), 10.83 (s, 1H), 10.69 (s, 1H), 9.65 (s, 1H), 7.51 (d, *J* = 1.7 Hz, 1H), 7.27 (s, 1H), 7.00 (d, *J* = 1.7 Hz, 1H), 6.65 (d, *J* = 2.2 Hz, 1H), 6.36 (d, *J* = 2.2 Hz, 1H) ppm. ^13^C NMR (151 MHz, DMSO-*d*_6_) *δ* = 157.8, 156.7, 155.3, 154.5, 154.2, 145.8, 137.6, 133.9, 115.3, 109.2, 106.0, 102.3, 101.9, 101.6 ppm. IR (KBr): *ν̃* = 3433(s), 3237(s), 3078(m), 1641(sh), 1635(s), 1534(m), 1468(m), 1392(m), 1368(m), 1238(m) cm^−1^. HRMS (ESI) *m*/*z* calcd for C_14_H_9_O_5_N_2_ [M–H^+^]^−^ 285.0517, found 285.0515.

##### 5-Hydroxy-2-(4,5,7-trihydroxynaphthalen-2-yl)pyrimidin-4(3*H*)-one (36)

4,5-Dimethoxy-2-(4,5,7-trimethoxynaphthalen-2-yl)pyrimidine (30) (151 mg, 0.42 mmol, 1.0 eq.) was dissolved in anhydrous CH_2_Cl_2_ (9.0 mL) under a nitrogen atmosphere. The solution was cooled to 0 °C in an ice bath, and a 1 M solution of BBr_3_ in CH_2_Cl_2_ (4.5 mL, 4.5 mmol, 10.7 eq.) was added dropwise. The reaction mixture was allowed to stir for 15 minutes at 0 °C, followed by 24 hours at room temperature. The reaction mixture was poured into a mixture of ice/water (30 mL). The mixture was lyophilized and dissolved in anhydrous 1,4-dioxane (2.1 mL), followed by addition of a 4 M solution of hydrogen chloride in 1,4-dioxane (4.2 mL, 16,8 mmol, 40 eq.). Then, the reaction mixture was heated to 110 °C for 24 hours until the starting material was consumed (UPLC-MS). The solvents were evaporated, and the residue was purified by preparative HPLC to afford the desired final product 36 (32 mg, 27%). ^1^H NMR (600 MHz, DMSO-*d*_6_) *δ* = 12.68 (v b s, 1H), 10.83 (s, 1H), 10.80 (s, 1H), 9.72 (br s, 1H), 9.62 (br s, 1H), 7.79 (d, *J* = 1.7 Hz, 1H), 7.58 (s, 1H), 7.11 (d, *J* = 1.6 Hz, 1H), 6.63 (d, *J* = 2.2 Hz, 1H), 6.40 (d, *J* = 2.2 Hz, 1H) ppm. ^13^C NMR (151 MHz, DMSO-*d*_6_) *δ* = 159.0, 156.8, 155.3, 154.4, 148.1*, 143.6, 137.1, 131.5*, 130.8, 116.5, 109.8, 103.3, 102.2, 102.1 ppm. IR (KBr): *ν̃* = 3439(s), 3250(s), 3088(m), 1641(sh), 1627(s), 1596(m), 1538(m), 1483(m), 1366(m), 1270(m) cm^−1^. HRMS (ESI) *m*/*z* calcd for C_14_H_11_O_5_N_2_ [M + H^+^]^+^ 287.0663, found 287.0665.

### 4.2 Cloning, expression, and purification of recombinant proteins

DNA encoding the first 196 amino acids of PA-Nter from the A/California/07/2009 (H1N1) strain (GenBank accession no. CY121685.1) with the flexible loop (residues 51–72) replaced by a GGS linker was prepared by GenScript USA Inc.^63^ The GST–PA-Nter construct was prepared by inserting DNA coding PA-Nter into pGEX-1λT. The His_6_-SUMO–PA-Nter construct was prepared by cloning DNA encoding PA-Nter with a (GS)_4_ linker extension at the N-terminal part into the plasmid pETM11-SUMO3 using *Bam*HI and *Xho*I sites. Both constructs were expressed in *E. coli* BL21 (DE3) RIL cells. Cells were harvested by centrifugation (6000*g*) and resuspended in lysis buffer [25 mM Tris-HCl, pH 7.5, 150 mM NaCl, 1 mM EDTA (GST) or 50 mM Tris-HCl, pH 8.0, 200 mM NaCl, 10 mM imidazole (His_6_-SUMO)] with cOmplete™, EDTA-free protease inhibitor cocktail (Roche). Resuspended cells were lysed with a CF1 cell disruptor (Constant Systems Limited) at a pressure of 15–20 ksi. The soluble proteins expressed from the GST construct were further purified *via* glutathione agarose and subsequently eluted with a mixture of 50 mM Tris-HCl, pH 7.5, 150 mM NaCl, 10 mM reduced l-glutathione, and 1 mM EDTA. Analogously, His_6_-SUMO–PA-Nter was purified by Ni-NTA agarose (Roche) and eluted with a mixture of 50 mM Tris-HCl, pH 8.0, 200 mM NaCl, and 250 mM imidazole. The His_6_-SUMO tag was cleaved with ULP1 protease and further eliminated from PA-Nter by Ni-NTA agarose. Both proteins were additionally purified by size exclusion chromatography on a HiLoad Superdex 75 pg column.

### 4.3 Crystallization and diffraction data collection

To obtain unoccupied crystals of PA-Nter, protein solution (10 mg mL^−1^) was mixed with crystallization reservoir solution (12.5% (w/v) MDP, 12.5% w/v PEG 1000, 12.5% (w/v) PEG 3350, 0.1 M imidazole/MES monohydrate, pH 6.5, 0.03 M magnesium chloride hexahydrate, 0.03 M calcium chloride dihydrate) and PA-Nter seed in a 1 : 1 : 0.2 ratio. Crystals grew in approximately 2 days at 291.14 K and were soaked overnight with 36 in reservoir solution supplemented with 1 mM ligand solution (final 5% DMSO concentration). Crystals were flash-cooled by plunging into liquid nitrogen and were stored in liquid nitrogen.

Diffraction data were collected at 100 K on the MX14.1 beamline at the BESSY II synchrotron, operated by the Helmholtz-Zentrum Berlin, Germany.^64^ Crystals of PA-Nter soaked with 36 (8PPX) diffracted to a resolution of up to 2.5 Å. Diffraction data were processed, integrated, and reduced using XDS^65^ and scaled using XSCALE from the XDS suite.^66^ Crystals belonged to the *P*6_4_22 space group and contained one molecule in the asymmetric unit, with a solvent content of approximately 47%. Crystal parameters and data collection statistics are given in Table S1.†

### 4.4 Structure determination and analyses

The structure of PA-Nter in complex with 36 was determined by molecular replacement with MOLREP^67^ from the CCP4 package^68^ using a previously determined PA-Nter structure as a template (PDB entry 6YA5).^46^ The protein structure with ligand was manually built and adjusted in Coot.^69^ Refinement was carried out using REFMAC 8.0.012.^69^ The MolProbity server^70^ was used to evaluate the final model quality. All protein structure figures were prepared with PyMOL (The PyMOL Molecular Graphics System, version 3.0.3 accessed on 1st May 2024; Schrödinger, LLC., New York, NY, USA). Atomic coordinates and structural factors were deposited in the PDB under accession code 8PPX.

### 4.5 AlphaScreen assay

AlphaScreen experiments were performed using a Perkin Elmer Enspire plate reader in 96-well ProxiPlates. Biotinylated L-742.001 derivative was captured on streptavidin-coated donor beads (Perkin Elmer). Separately, GST–PA-Nter fusion protein was bound to GSH-coated acceptor beads (Perkin Elmer). These solutions were incubated for 60 min at room temperature in the dark and subsequently mixed and incubated for an additional 120 min. In experiments screening for endonuclease inhibitors, compounds were mixed with both types of beads prior to the 120 min incubation. The optimal concentrations of biotinylated L-742.001 derivative and GST–PA-Nter were 15 nM and 50 nM, respectively. The concentrations of donor and acceptor beads were 5 μg mL^−1^ each in a 50 μL reaction volume. All experiments were performed in AlphaScreen reaction buffer (25 mM Tris-HCl, pH 7.4, 150 mM NaCl, 0.05% Tween 20, 1 mM MnCl_2_, 10 mM MgCl_2_, and 1 mM 2-mercaptoethanol).

### 4.6 Anti-influenza A H1N1 California effect of pseudoflavonoid compounds, cytopathic effect (CPE) detection

Anti-influenza A (A/California/07/2009 (H1N1)) activity of selected compounds was tested in MDCK cells (20 000 cells per 100 μL) and A549 cells (25 000 cells per 100 μL) in 96-well plates. Compounds were added to the cells, and after one hour, cells were infected with influenza A (H1N1) (MOI 0.002 for MDCK cells and MOI 0.02 for A549 cells). Infection was carried out in influenza growth medium (DMEM high glucose, no serum, 10 mM HEPES, 0.125% BSA fraction, 1 μg mL^−1^ TPCK-trypsin, penicillin/streptomycin). Cells were incubated for 3 days (MDCK cells) or 4 days (A549) at 37 °C, 5% CO_2_. After incubation, CPE was analysed by XTT colorimetric assay. Then, 50 μL of a 50 : 1 mixture of XTT labelling reagent (1 mg mL^−1^) and PMS electron-coupling reagent (0.383 mg mL^−1^) was added to the wells and incubated for 4 hours at 37 °C in 5% CO_2_. Formation of orange formazan dye was measured with an EnVision plate reader. Experiments were performed in biological triplicates, and the resulting data were analysed using GraphPad Prism 9.0 software (GraphPad Software, San Diego, CA, USA).

### 4.7 Minireplicon assay

All plasmids for the minireplicon assay were kindly provided by Prof. Yoshihiro Kawaoka, University of Wisconsin, Madison, USA. HEK293T cells were seeded in a 96-well plate at a concentration of 3 × 10^5^ cells per well in 100 μL of DMEM complete (10% FBS, penicillin/streptomycin) medium. Alternatively, A549 cells were seeded in a 96-well plate at a concentration of 3 × 10^5^ cells per well in 100 μL of DMEM complete medium. The cells were co-transfected with a set of plasmids encoding the three polymerase subunits and the viral nucleoprotein (pCAGGS-PB1, pCAGGS-PB2, pCAGGS-PA, pCAGGS-NP, all sequences originating from influenza A/WSN/33 H1N1 strain), and with an influenza virus-specific RNA polymerase I-driven firefly luciferase reporter plasmid (pPolI-Flu-ffLuc), using Lipofectamine 2000 (Thermo Fisher Scientific, Waltham, MA, USA). To minimize transfection variability, the plasmid pGL4.74 (Promega, Madison, WI, USA) encoding the sequence for *Renilla* luciferase was used as an internal control. Cells were harvested two days after transfection and incubation with peptides, and luciferase expression was determined using Dual-Glo® Luciferase Assay System (Promega, Madison, USA) according to the supplied protocol. The experiments were performed in biological triplicates, and the resulting data were analysed using GraphPad Prism 9.0 software (GraphPad Software, San Diego, CA, USA).

### 4.8 Cytotoxicity assay

The cytotoxic concentrations that reduced target cell viability by 50% (CC_50_) were determined by incubating serial dilutions of each test compound and control compounds with the selected cell cultures. HEK 293T, A549 and MDCK cells were seeded at 3 × 10^3^ cells per well in 100 μL DMEM complete medium. The following day, compounds were added to the corresponding wells, and the cells were incubated for 48 hours at 37 °C in 5% CO_2_. After incubation, cell viability was analyzed by XTT colorimetric assay. Then, 50 μL of a 50 : 1 mixture of XTT labelling reagent (1 mg mL^−1^) and PMS electron-coupling reagent (0.383 mg mL^−1^) were added to the wells and incubated for 4 hours at 37 °C in 5% CO_2_. The formation of orange formazan dye was measured on a Tecan Spark plate reader (Tecan Life Sciences, USA). The experiments were performed in biological triplicates, and the resulting data were analyzed using GraphPad Prism 10.0 software (GraphPad Software, San Diego, CA, USA).

## Declaration of generative AI and AI-assisted technologies in the writing process

During the preparation of this work the authors used ChatGPT in order to improve the readability and language of the manuscript.

## Author contributions

Róbert Reiberger: investigation. Michal Kráľ: investigation, validation, methodology. Kateřina Radilová: methodology, supervision. Tomáš Kotačka: investigation. Artem Tsalyy: investigation. Jiří Brynda: validation, supervision. Pavel Majer: supervision. Jan Konvalinka: writing – review & editing, funding acquisition. Milan Kožíšek: validation, methodology, data curation, writing – original draft, funding acquisition. Aleš Machara: writing – original draft, supervision. Correspondence respective to chemical synthesis should be directed to Dr. Machara. Correspondence respective to evaluations of inhibitory potency of tested compounds should be directed to Dr. Kožíšek.

## Conflicts of interest

There are no conflicts to declare.

## Supplementary Material

MD-OLF-D5MD00071H-s001

## Data Availability

The data supporting this article have been included as part of the ESI.†
