# Supplementary material for: Beyond natural flavonoids: exploring bioisosterism in design and synthesis of influenza endonuclease inhibitors
Source: RSC Med Chem. 2025 Mar 11;16(7):3030–48. doi: 10.1039/d5md00071h (PMC12154555; doi:10.1039/d5md00071h)
Supplement: MD-016-D5MD00071H-s001 [file MD-016-D5MD00071H-s001.pdf]

Supplementary Information (SI) for RSC Medicinal Chemistry.
This journal is © The Royal Society of Chemistry 2025

RSC
Medicinal Chemistry

*Supplemental Material to:*

# Beyond Natural Flavonoids: Exploring Bioisosterism in Design and Synthesis of Influenza Endonuclease Inhibitors

Róbert Reiberger,[a, b] Michal Kráľ,[a, c] Kateřina Radilová,[a] Tomáš Kotačka,[a, c] Martin Dračínský,[a] Artem Tsalyy,[a] Jiří Brynda,[a, e] Pavel Majer,[a] Jan Konvalinka,[a,d] Milan Kožíšek,[a,*] Aleš Machara[a,*]

[a.] *Institute of Organic Chemistry and Biochemistry of the Czech Academy of Sciences, Gilead Sciences And IOCB Research Center, Flemingovo n. 2, 166 10, Prague 6, Czech Republic*

[b.] *Department of Organic Chemistry, Faculty of Science, Charles University, Hlavova 8, 128 00, Prague 2, Czech Republic*

[c.] *First Faculty of Medicine, Charles University, Kateřinská 1660, 121 08, Prague 2, Czech Republic*

[d.] *Department of Biochemistry, Faculty of Science, Charles University, Hlavova 8, 128 00, Prague 2, Czech Republic*

[e.] *Institute of Molecular Genetics of the Czech Academy of Sciences, Vídeňská 1083, 140 00, Prague 4, Czech Republic*

* **Corresponding authors**

Milan Kožíšek and Aleš Machara, Institute of Organic Chemistry and Biochemistry, Academy of Sciences of the Czech Republic, Flemingovo n. 2, 166 10 Prague 6, Czech Republic

*Tel: +420 220 183 518; e-mails: milan.kozisek@uochb.cas.cz, ales.machara@uochb.cas.cz*

# Table of Contents

General information ........ 3

Experimental procedures ........ 4

Synthesis of heterocyclic intermediates ........ 4

Anti-Influenza A H1N1 California effect of compounds, cytopathic effect (CPE) detection.... 8

Minireplicon assay ........ 9

Cytotoxicity assay ........ 11

AlphaScreen Assay ........ 13

Crystallographic data collection and refinement statistics ........ 14

Literature ........ 16

$^{1}H$, $^{13}C$ and APT NMR spectra ........ 17

IR spectra of pseudoflavonoids ........ 97

HRMS spectra ........ 104

## General information

Unless noted otherwise, all reactions were carried out under argon in oven-dried glassware. Anhydrous solvents were distilled from solvents using agents as indicated and transferred under nitrogen: THF (Na/benzophenone), toluene (Na/benzophenone), MeCN ($CaH_2$), and DCM ($CaH_2$). Chromatography was performed using flash chromatography Teledyne ISCO CombiFlash NextGen 300+ system with RediSep Rf Gold Silica or RediSep (marked as $SiO_2$) Rf Gold Reversed-phase C18 columns (marked as $SiO_2$-$C_{18}$ or $SiO_2$-$C_{18}$-Aq). All starting materials were used as purchased (Merck, Alfa Aesar, TCI, Fluorochem, Combi-Blocks), unless otherwise indicated. All inhibitors were purified using an ECOM compact preparative system TOY18DAD800 (flow rate 15 mL/min; gradient 0 → 60% or 0 → 80% $H_2O$ (0.1% trifluoroacetic acid)/MeCN in 60 min), with a ProntoSIL 120-10-C18 ace-EPS column, 10 µm, 20 × 250 mm. The purity of compounds and composition of the reaction mixtures were tested on a Waters UPLC-MS Acquity with QDa Mass Detector (flow rate 0.5 mL/min, gradient 0 → 100% $H_2O$ (0.1% formic acid)/MeCN in 7 min) with an ACQUITY UPLC BEH C18 Column, 130 Å, 1.7 µm, 2.1 mm × 100 mm with 2.1 mm × 5 mm pre-column. The final inhibitors were of at least 95% purity. $^{1}$H-NMR spectra were recorded on Bruker instruments at 401, 500 or 600 MHz; $^{13}$C-NMR spectra were recorded at 101, 126 or 151 MHz. Chemical shifts are provided in $\delta$-scale in ppm; coupling constants $J$ are given in Hz. Chemical shifts marked with an asterisk (*) are visible in two-dimensional NMR spectra (HMBC). FT-IR spectra were recorded using Nicolet iS50 spectrophotometer. Wavenumbers $\tilde{\nu}$ are provided in cm$^{-1}$. ESI or APCI high resolution mass spectra were recorded using a Thermo Scientific LTQ Orbitrap XL (Termo Fisher Scientific) and EI or CI high resolution mass spectra were recorded using an Agilent 7250 GC/Q-TOF, both controlled by MassLynx software.

# Experimental procedures

## Synthesis of heterocyclic intermediates

*5-Bromo-2,2-dimethylbenzo[d][1,3]dioxole (**37**):*

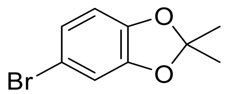

Compound was prepared according to according to the published procedure[1]. 2,2-Dimethylbenzo[*d*][1,3]dioxole (1.00 g, 6.66 mmol, 1.0 eq.) was dissolved in DMF (20 mL) followed by an addition of *N*-bromosuccinimide (1.18 g, 6.66 mmol, 1.0 eq.). The reaction mixture was allowed to stir for 16 hours until the starting material was consumed (GC-MS). Then EtOAc (50 mL) was added, and the organic phase was washed with water (3 × 80 mL) and brine (1 × 20 mL). Then the organic phase was dried over anhydrous $MgSO_4$, and the solvents were evaporated, and the residue was purified by flash chromatography ($SiO_2$, *n*-hexane/EtOAc = 100:0 → 95:5) to afford the desired acetonide **37** (1.28 g, 84%). $^1$H NMR (401 MHz, $CDCl_3$) $\delta$ = 6.89 (dd, $J$ = 8.1, 2.0 Hz, 1H), 6.86 (d, $J$ = 2.0 Hz, 1H), 6.60 (d, $J$ = 8.1 Hz, 1H), 1.67 (s, 6H) ppm. $^{13}$C NMR (101 MHz, $CDCl_3$) $\delta$ = 148.4, 146.8, 123.7, 119.0, 112.4, 112.1, 109.4, 25.8 ppm.

*(2,2-Dimethylbenzo[d][1,3]dioxol-5-yl)boronic acid (**38**):*

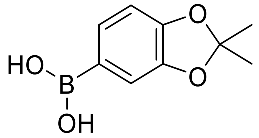

Compound was prepared according to according to the published procedure[2].

Compound **37** (2.0 g, 8.73 mmol, 1.0 eq.) was dissolved in anhydrous THF (44 mL) in flame-dried flask under argon atmosphere. The solution was cooled to −78 °C followed by an addition of 2.5M solution of *n*-butyllithium in hexanes (4.2 mL, 10.5 mmol, 1.2 eq.) dropwise at −78 °C under argon atmosphere. Then the reaction mixture was allowed to stir for 1 hour followed by an addition of triisopropyl borate (2.4 mL, 10.5 mmol, 1.2 eq.) dropwise. The reaction mixture was allowed to stir for 1 hour −78 °C and then 16 hours at room temperature. Then the reaction mixture was quenched by water (2.0 mL) followed by an addition of 5% HCl (aq.) until pH = 4 and slurry was dissolved. The aqueous phase was extracted with EtOAc (3 × 50 mL) and combined organic phases was washed with brine (1 × 10 mL) and dried over anhydrous $MgSO_4$. The solvents were evaporated, and the residue was purified by flash chromatography ($SiO_2$, *n*-hexane/EtOAc = 100:0 → 0:100) to afford the desired boronic acid **38** (1.21 g, 71%). $^1$H NMR (401 MHz, $CDCl_3$) $\delta$ = 7.75 (dd, $J$ = 7.7, 1.2 Hz, 1H), 7.53 (d, $J$ = 1.2 Hz,

1H), 6.86 (d, $J$ = 7.8 Hz, 1H), 1.73 (s, 6H) ppm. $^{13}$C NMR (101 MHz, $CDCl_3$) $\delta$ = 151.4, 147.5, 130.7, 118.2, 114.4, 108.5, 26.1 ppm.

**Bromination of 2,3-dimethoxypyridine:**

2,3-Dimethoxypyridine (1.00 g, 7.2 mmol, 1.0 eq.) was dissolved in anhydrous DMF (2.0 mL) followed by an addition of solution of *N*-bromosuccinimide (1.28 g, 7.2 mmol, 1.0 eq.) in anhydrous DMF (1.0 mL). Then the reaction mixture was allowed to stir for 24 hours at room temperature until the starting material was consumed (TLC, UPLC-MS). Then EtOAc (40 mL) was added, and organic phase was washed with water (3 × 100 mL) and brine (1 × 10 mL). The organic phase was dried over anhydrous $MgSO_4$. The solvents were evaporated, and the residue was purified by flash chromatography ($SiO_2$-$C_{18}$, $H_2O$ (0.1% TFA)/MeCN= 100:0 → 30:70) to obtain two fractions. Both were separately concentrated to half of volume and extracted with dichloromethane (3 × 30 mL), washed with water (1 × 20 mL) and dried over anhydrous $MgSO_4$. The solvents were evaporated to afford more polar 5-bromo-2,3-dimethoxypyridine (**39**) (628 mg, 40%) and less polar 6-bromo-2,3-dimethoxypyridine (**40**) (707 mg, 45%).

*5-Bromo-2,3-dimethoxypyridine (**39**):*

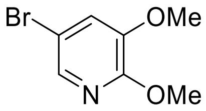


$^1$H NMR (401 MHz, $CDCl_3$) $\delta$ = 7.76 (d, $J$ = 2.1 Hz, 1H), 7.12 (d, $J$ = 2.1 Hz, 1H), 3.97 (s, 3H), 3.85 (s, 3H) ppm. $^{13}$C NMR (101 MHz, $CDCl_3$) $\delta$ = 153.5, 144.6, 137.4, 120.3, 111.2, 56.1, 54.0 ppm. MS (ESI) *m/z* calcd for $C_7H_9BrNO_2$ $[M + H^+]^+$ 217.98, found 217.75.

*6-Bromo-2,3-dimethoxypyridine (**40**):*

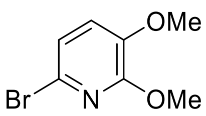


$^1$H NMR (401 MHz, $CDCl_3$) $\delta$ = 6.96 (d, $J$ = 8.0 Hz, 1H), 6.89 (d, $J$ = 8.0 Hz, 1H), 3.98 (s, 3H), 3.82 (s, 3H) ppm. $^{13}$C NMR (101 MHz, $CDCl_3$) $\delta$ = 153.9, 143.6, 126.7, 119.9, 119.8, 56.1, 54.5 ppm. MS (ESI) *m/z* calcd for $C_7H_9BrNO_2$ $[M + H^+]^+$ 217.98, found 217.78.

*2-Bromo-4,5-dimethoxypyridine (**41**):*

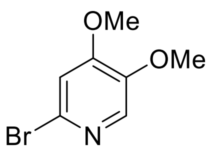


2-Bromo-5-fluoro-4-iodopyridine (1.21 g, 4.0 mmol, 1.0 eq.) was dissolved in anhydrous DMF (2.5 mL). Then the flask was put into a cold-water bath (approx. 15 °C) and freshly prepared sodium methoxide (540 mg, 10 mmol, 2.5 eq.) was added to the mixture. The reaction mixture was allowed to stir for 2 hours at room temperature under nitrogen atmosphere until starting material was consumed (according to

UPLC-MS ($\lambda$ = 254 nm): ratio (2-bromo-4,5-dimethoxypyridine/2-bromo-4-iodo-5-methoxypyridine) = 27.6:72.4). Then the reaction mixture was quenched by an addition of saturated aqueous solution of $NH_4Cl$ (30 mL) and water (30 mL). Aqueous phase was extracted with EtOAc (1 × 20 mL). Then the organic phase was washed with water (3 × 50 mL), brine (1 × 10 mL) and dried over anhydrous $MgSO_4$. The solvents were evaporated, and the residue was purified by flash chromatography ($SiO_2$, cyclohexane/hexane/EtOAc = 100:0 → 0:100) to afford the desired 2-bromo-4,5-dimethoxypyridine (**41**) (242 mg, 28%). $^1$H NMR (401 MHz, $CDCl_3$) $\delta$ = 7.80 (s, 1H), 6.88 (s, 1H), 3.87 (s, 6H) ppm. $^{13}$C NMR (101 MHz, $CDCl_3$) $\delta$ = 156.5, 145.9, 133.8, 132.6, 110.5, 56.7, 56.3 ppm. HRMS (EI) *m/z* calcd for $C_7H_8BrNO_2$ $[M]^{+\bullet}$ 216.9733, found 216.9731.

*2,3-Dimethoxypyrazine (**42**):*

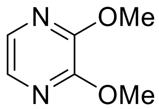


2,3-Dichloropyrazine (1.50 g, 10.1 mmol, 1.0 eq.) was dissolved in anhydrous methanol (10 mL). Then the solution was cooled to 0°C by ice/water bath and freshly prepared sodium methoxide (4.91 g, 90.1 mmol, 9.0 eq.) was added in one portion. Then the reaction mixture was allowed to stir for 30 minutes at 0 °C and then for 2 hours at room temperature under nitrogen atmosphere until the starting material was consumed (TLC, UPLC-MS). Solvent was evaporated and residual solids were suspended in water (50 mL). Aqueous phase was extracted with dichloromethane (3 × 40 mL) and combined organic phases were washed with brine (1 × 10 mL) dried over anhydrous $MgSO_4$. The solvents were evaporated to afford the desired 2,3-dimethoxypyrazine (**42**) (1.12 g, 84%). $^1$H NMR (401 MHz, $CDCl_3$) $\delta$ = 7.61 (s, 2H), 4.00 (s, 6H) ppm. $^{13}$C NMR (101 MHz, $CDCl_3$) $\delta$ = 150.7 (2C), 132.1 (2C), 54.0 (2C) ppm. MS (ESI) *m/z* calcd for $C_6H_9N_2O_2$ $[M + H^+]^+$ 141.06, found 141.07.

*5-Bromo-2,3-dimethoxypyrazine (**43**):*

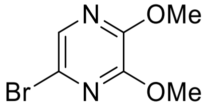


Compound **42** (280 mg, 2.0 mmol, 1.0 eq.) was dissolved in anhydrous DMF (2.8 mL) followed by an addition of *N*-bromosuccinimide (498 mg, 2.8 mmol, 1.4 eq.). Then the reaction mixture was allowed to stir for 4 days at room temperature until the starting material was consumed (TLC, UPLC-MS). Then EtOAc (20 mL) was added, and organic phase was washed with water (3 × 50 mL). The organic phase was dried over anhydrous $MgSO_4$. The solvents were evaporated to afford the desired 5-bromo-2,3-dimethoxypyrazine (**43**) (107 mg, 24%). $^1$H NMR (401 MHz, $CDCl_3$) $\delta$ = 7.69 (s, 1H), 4.01 (s,

3H), 3.98 (s, 3H) ppm. $^{13}$C NMR (101 MHz, $CDCl_3$) $\delta$ = 149.7, 149.6, 133.0, 124.9, 54.9, 54.5 ppm. MS (ESI) *m/z* calcd for $C_6H_8BrN_2O_2$ [M + $H^+$]$^+$ 218.98, found 218.76.

*6-Chloro-3,4-dimethoxypyridazine (**44**):*

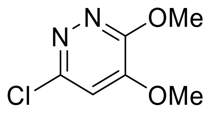

Compound was prepared according to according to the published procedure[3]. 3,4,6-Trichloropyridazine (2.00 g, 10.9 mmol, 1.0 eq.) was dissolved in anhydrous methanol (150 mL). Then the solution was cooled to 0°C by ice/water bath and freshly prepared sodium methoxide (1.76 g, 32.7 mmol, 2.0 eq.) was added. Then the reaction mixture was allowed to stir for 48 hours at room temperature under nitrogen atmosphere until conversion of starting material stayed unchanged (according to UPLC-MS ($\lambda$ = 254 nm): ratio (product/starting material) = 30.1:69.9). Solvent was evaporated and residual solids were dissolved in EtOAc (40 mL). Organic phase was washed with saturated aqueous solution of $NH_4Cl$ (3 × 40 mL) and brine (1 × 20 mL) dried over anhydrous $MgSO_4$. The solvents were evaporated, and the residue was purified by flash chromatography ($SiO_2$, cyclohexane/EtOAc = 100:0 → 70:30) to afford the desired 6-chloro-3,4-dimethoxypyridazine (**44**) (286 mg, 15%). $^1$H NMR (401 MHz, $CDCl_3$) $\delta$ = 6.76 (s, 1H), 4.14 (s, 3H), 3.94 (s, 3H) ppm. $^{13}$C NMR (101 MHz, $CDCl_3$) $\delta$ = 157.1, 151.2, 149.9, 56.4, 55.5 ppm. MS (ESI) *m/z* calcd for $C_6H_8ClN_2O_2$ [M + $H^+$]$^+$ 175.03, found 175.15.

*2-Chloro-4,5-dimethoxypyrimidine (**45**):*

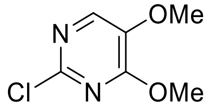

Compound was prepared according to according to the published procedure[3]. 2,4-Dichloro-5-methoxypyrimidine (1.00 g, 5.59 mmol, 1.0 eq.) was dissolved in methanol (21 mL) followed by an addition of potassium carbonate (772 mg, 5.59 mmol, 1.0 eq.) Then the reaction mixture was allowed to stir for 16 hours at room temperature under nitrogen atmosphere until the starting material was consumed (TLC, UPLC-MS). Solvent was evaporated and residual solids were suspended in EtOAc (50 mL). Organic phase was washed with water (2 × 50 mL), brine (1 × 10 mL) and dried over anhydrous $MgSO_4$. The solvents were evaporated to afford the desired 2-chloro-4,5-dimethoxypyrimidine (**45**) (801 mg, 82%). $^1$H NMR (401 MHz, $CDCl_3$) $\delta$ = 7.87 (s, 1H), 4.06 (s, 3H), 3.91 (s, 3H) ppm. $^{13}$C NMR (101 MHz, $CDCl_3$) $\delta$ = 161.3, 150.1, 141.8, 138.2, 56.7, 55.2 ppm. MS (ESI) *m/z* calcd for $C_6H_8ClN_2O_2$ [M + $H^+$]$^+$ 175.03, found 175.09.

## Anti-Influenza A H1N1 California effect of compounds, cytopathic effect (CPE) detection

Anti-Influenza A H1N1 California activity of selected compounds was tested in MDCK cells (20,000 cells/100 µL), and A549 cells (25,000 cells/100 µL) in 96-well plate. Compounds were added to the cells, and after one hour, cells were infected with Influenza A H1N1 California virus (MOI 0.002 for MDCK cells and MOI 0.02 for A549 cells). Infection was carried out in the Influenza growth medium (DMEM high glucose, no serum, 10 mM HEPES, 0.125% BSA Fraction, 1 µg/mL TPCK-Trypsin, Penicillin/Streptomycin)

Cells were incubated for 3 days (MDCK cells) or 4 days (A549) in 37°C, 5% CO2. After incubation, cytopathic effect (CPE) was analysed by XTT colorimetric assay. 50 µl of 50:1 mixture of XTT labelling reagent (1 mg/mL) and PMS electron-coupling reagent (0.383 mg/mL) was added to the wells and incubated for 4 hours at 37 °C in 5% $CO_2$. Formation of orange formazan dye was measured in EnVision plate reader. The experiments were performed in biological triplicates, and the resulting data were analysed using GraphPad Prism 9.0 software (GraphPad Software, San Diego, CA, USA).

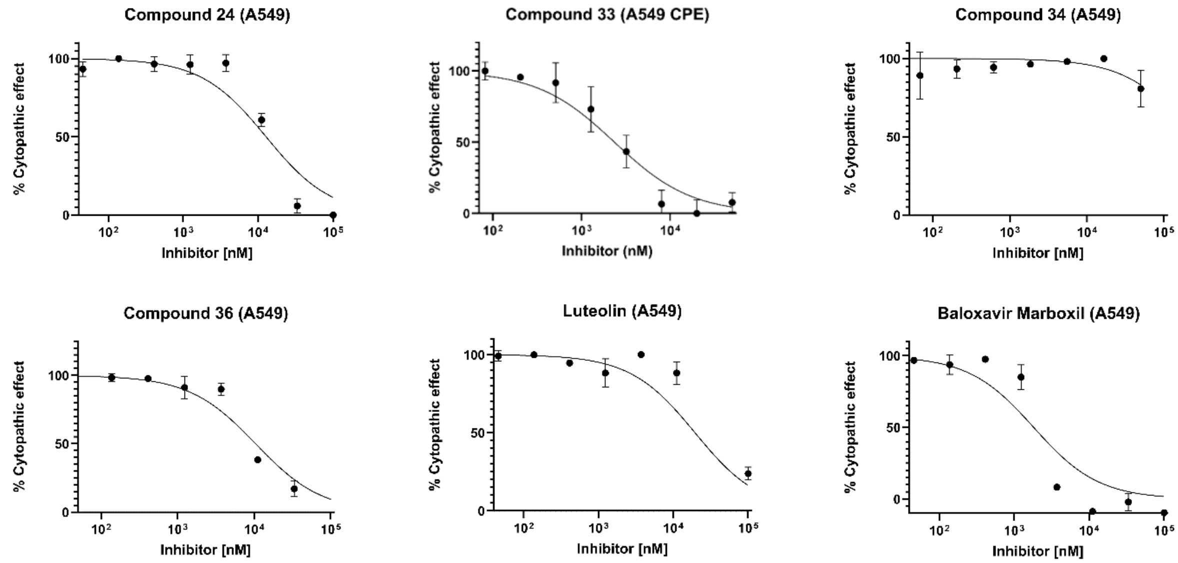

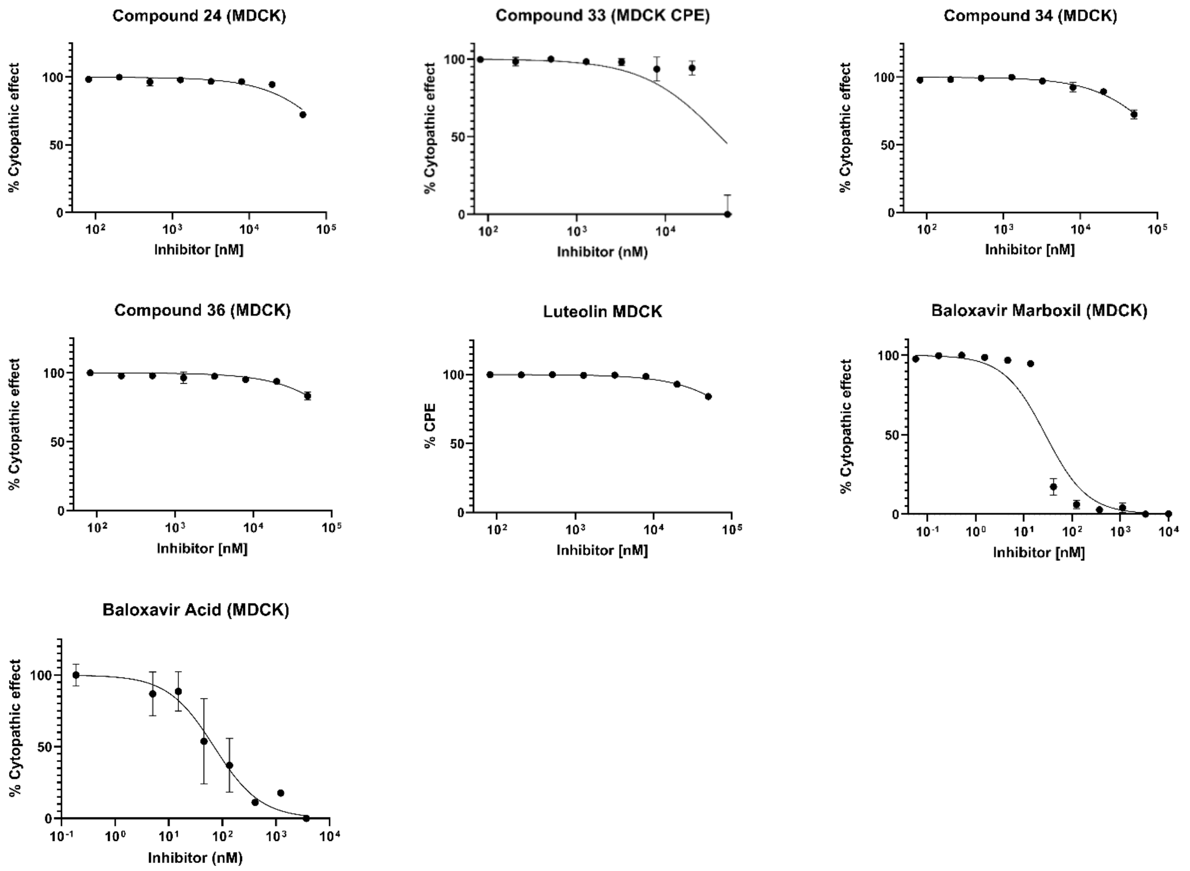


## Minireplicon assay

To determine the effect of the tested compounds on polymerase activity in cell, we used a minireplicon assay adapted as described. All plasmids for the minireplicon assay were kindly provided by Prof. Yoshihiro Kawaoka, University of Wisconsin, Madison, USA. HEK293T cells were seeded in a 96-well plate at a concentration of $3x10^5$ cells per well in 100 µL of DMEM complete (10% FBS, Penicillin/Streptomycin) medium. Alternatively, A549 cells were seeded in a 96-well plate at a concentration of $3x10^5$ cells per well in 100 µL of DMEM complete (10% FBS, Penicillin/Streptomycin) medium. The cells were co-transfected with a set of plasmids encoding the three polymerase subunits and the viral nucleoprotein (pcDNA-PB1, pcDNA-PB2, pcDNA-PA, pcDNA-NP, all sequences originating from influenza A/WSN/33 H1N1 strain), and with an influenza virus-specific RNA polymerase I-driven firefly luciferase reporter plasmid (pPolI-Flu-ffLuc), using Lipofectamine 2000 (Thermo Fisher Scientific, Waltham, USA). To minimize transfection variability, the plasmid pGL4.74 (Promega, Madison, USA) encoding the sequence for *Renilla* luciferase was used as an internal control. Cells were harvested two days after

transfection and incubation with peptides, and luciferase expression was determined using Dual-Glo® Luciferase Assay System (Promega, Madison, USA) as per the supplied protocol. The experiments were performed in biological triplicates, and the resulting data were analysed using GraphPad Prism 9.0 software (GraphPad Software, San Diego, CA, USA).

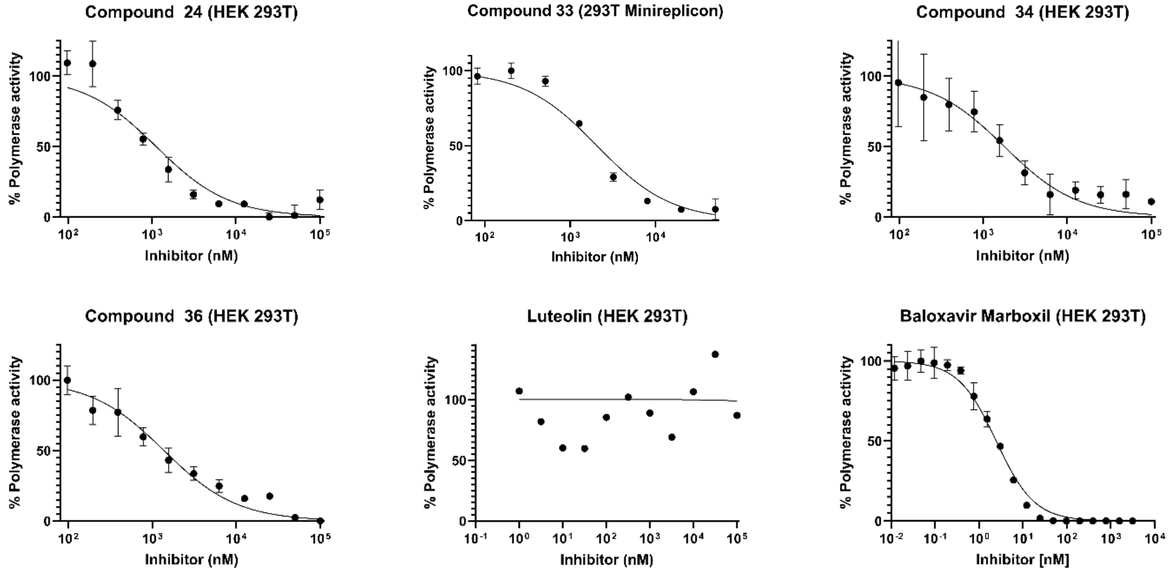


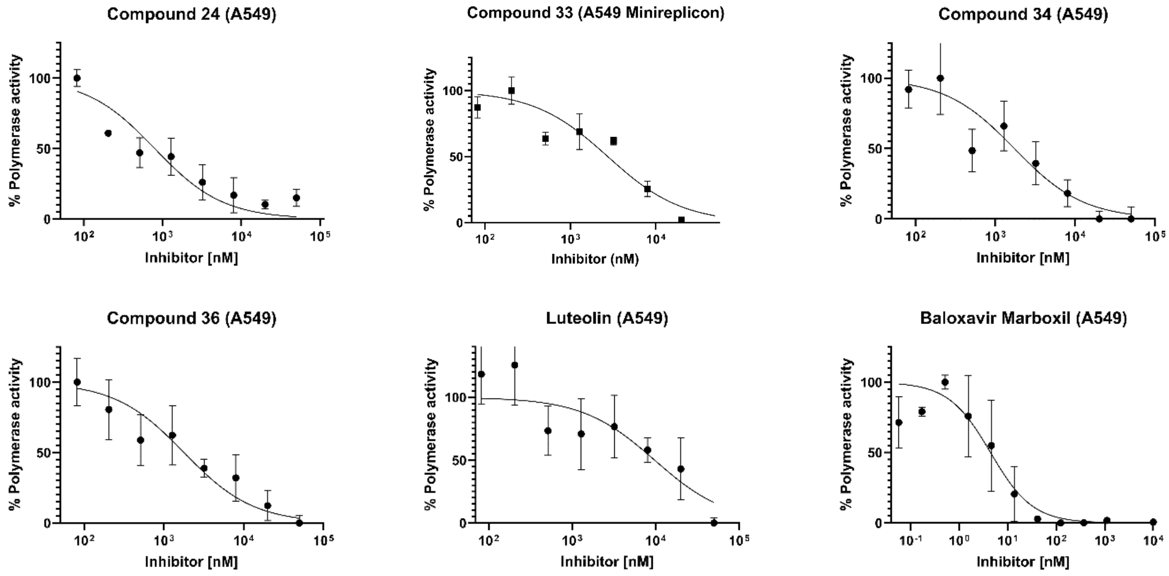

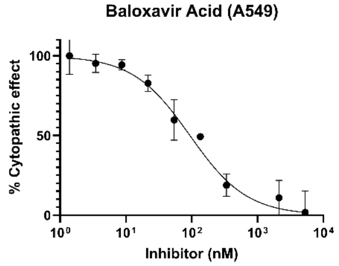


## Cytotoxicity assay

The cytotoxic concentrations that reduced target cell viability by 50% ($CC_{50}$) were determined by incubating serial dilutions of each tested compound and control compounds with the selected cell cultures. HEK 293T cells were seeded at $3x10^3$ cells per well in 100 μl of DMEM complete medium. The following day the tested compounds were added to the corresponding well and the cells were incubated for 48 hours at 37 °C in 5% $CO_2$. After incubation, cell viability was analysed by XTT colorimetric assay. 50 μl of 50:1 mixture of XTT labelling reagent (1 mg/mL) and PMS electron-coupling reagent (0.383 mg/mL) were added to the wells and incubated for 4 hours at 37 °C in 5% $CO_2$. The formation of orange formazan dye was measured on a Tecan Spark plate reader (Tecan Life Sciences, USA). The experiments were performed in biological triplicates, and the resulting data were analyzed using GraphPad Prism 10.0 software (GraphPad Software, San Diego, CA, USA).

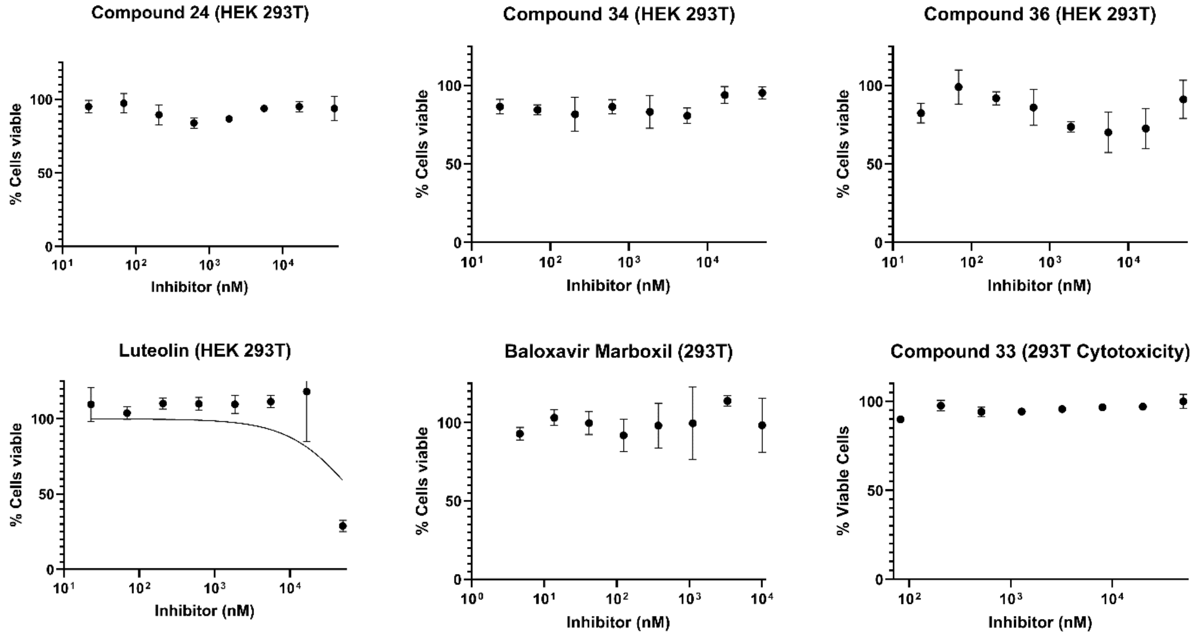

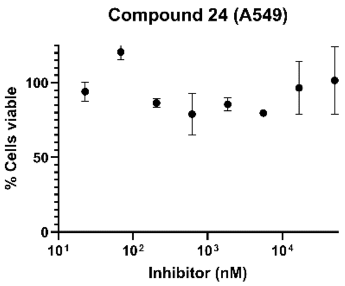
Compound 24 (A549)
% Cells viable
Inhibitor (nM)

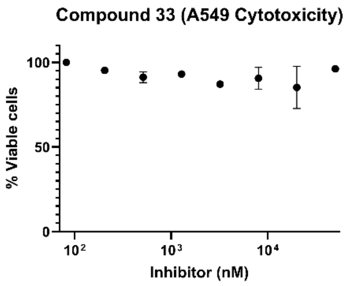
Compound 33 (A549 Cytotoxicity)
% Viable cells
Inhibitor (nM)

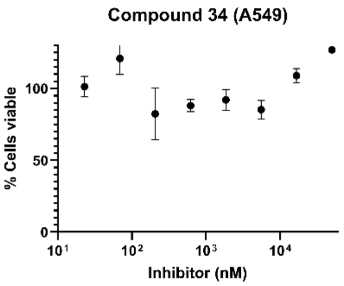
Compound 34 (A549)
% Cells viable
Inhibitor (nM)

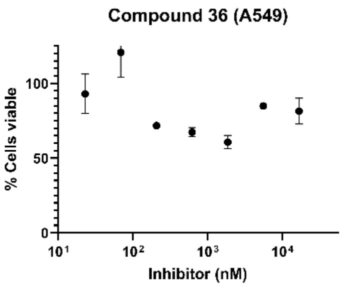
Compound 36 (A549)
% Cells viable
Inhibitor (nM)

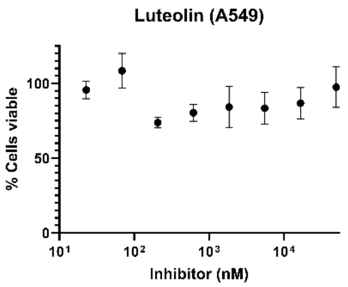
Luteolin (A549)
% Cells viable
Inhibitor (nM)

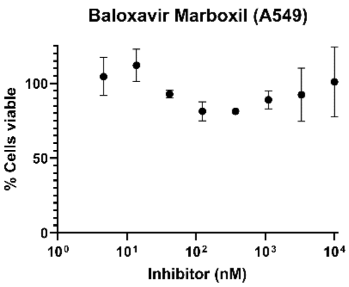
Baloxavir Marboxil (A549)
% Cells viable
Inhibitor (nM)

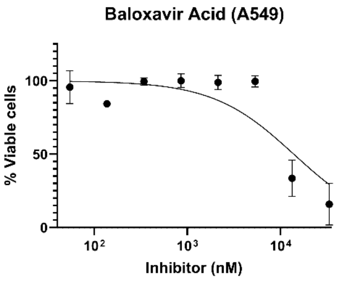
Baloxavir Acid (A549)
% Viable cells
Inhibitor (nM)

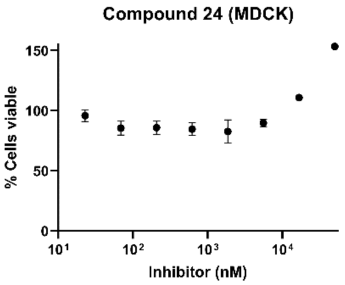
Compound 24 (MDCK)
% Cells viable
Inhibitor (nM)

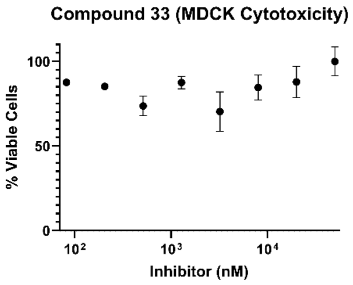
Compound 33 (MDCK Cytotoxicity)
% Viable Cells
Inhibitor (nM)

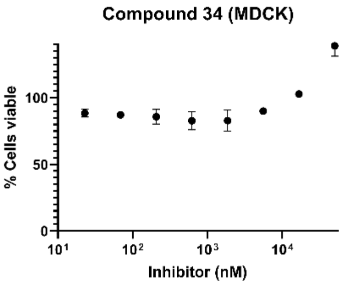
Compound 34 (MDCK)
% Cells viable
Inhibitor (nM)

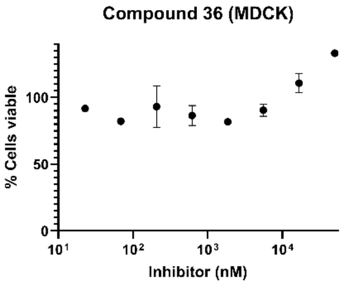
Compound 36 (MDCK)
% Cells viable
Inhibitor (nM)

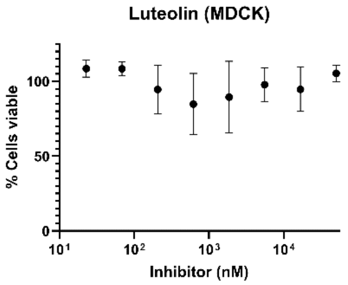
Luteolin (MDCK)
% Cells viable
Inhibitor (nM)

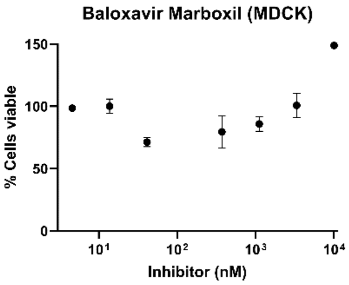
Baloxavir Marboxil (MDCK)
% Cells viable
Inhibitor (nM)

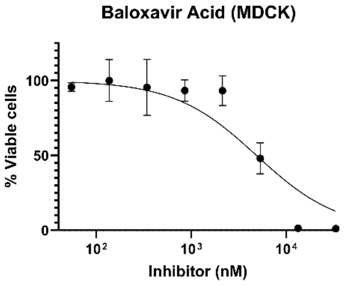


## AlphaScreen Assay

AlphaScreen experiments were performed using a Perkin Elmer Enspire plate reader in 96-well ProxiPlates. Biotinylated L-742.001 derivative was captured on Streptavidin-coated donor beads (Perkin Elmer). Separately, GST-PA-Nter fusion protein was bound to GSH-coated acceptor beads (Perkin Elmer). These solutions were incubated for 60 min at room temperature in the dark and subsequently mixed and incubated for an additional 120 min. In experiments screening for endonuclease inhibitors, compounds were mixed with both types of beads prior to the 120-min incubation. The optimal concentrations of biotinylated L-742.001 derivative and GST-PA-Nter were 15 nM and 50 nM, respectively. The concentrations of donor and acceptor beads were 5 µg/mL each in a 50 µL reaction volume. All experiments were performed in AlphaScreen reaction buffer (25 mM Tris-HCl, pH 7.4, 150 mM NaCl, 0.05% Tween20, 1 mM $MnCl_2$, 10 mM $MgCl_2$, and 1 mM 2-mercaptoethanol).

## Crystallographic data collection and refinement statistics

Supplementary **Table S1** – Diffraction data collection and refinement statistics. The data in parentheses refer to the highest-resolution shell for data collection statistic.

| NPA in complex with compound **36** | |
|---|---|
| Wavelength (Å) | 0.9184 |
| Space group | $P6_422$ |
| Cell parameters (Å, °) | 73.93, 73.93, 128.16,<br>90.00, 90.00, 120.00 |
| Resolution range (Å) | 50.00–2.46 (2.61–2.46) |
| Number of unique reflections | 14236 (2289) |
| Multiplicity | 20.9 (20.7) |
| Completeness (%) | 99.9 (99.9) |
| $R_{merge}$[a] | 0.27 (5.16) |
| $CC_{(1/2)}$ (%) | 99.8 (32.1) |
| Average I/σ(I) | 11.93 (0.64) |
| Wilson B (Å$^2$) | 66.35 |
| Refinement statistics | |
| Resolution range (Å) | 45.3–2.5 (2.57–2.5) |
| No. of reflection in working set | 7306 (527) |
| No. of reflection in the test set | 385 (28) |
| $R_{work}$ value (%)[b] | 0.227 (0.345) |
| $R_{free}$ value (%)[c] | 0.258 (0.370) |
| RMSD bond length (Å) | 0.004 |
| RMSD angle (°) | 1.024 |
| Number of atoms in AU | 1486 |
| Number of protein atoms in AU | 1437 |
| Number of water molecules in AU | 25 |
| Mean ADP value (Å$^2$) | 58.05 |
| Ramachandran plot statistics[d] | |
| Residues in favored regions (%) | 93.89 |
| Residues in allowed regions (%) | 6.11 |
| PDB code | **8PPX** |

[a] $R_{merge} = (|I_{hkl} - \langle I \rangle|)/I_{hkl}$, where the average intensity ⟨I⟩ is taken over all symmetry equivalent measurements and $I_{hkl}$ is the measured intensity for any given reflection

[b] R-value = $||F_o| - |F_c||/|F_o|$, where $F_o$ and $F_c$ are the observed and calculated structure factors, respectively.

[c] $R_{free}$ is equivalent to R-value but is calculated for 5% of the reflections chosen at random and omitted from the refinement process.[4]

[d] As determined by Molprobity.[5]

## Literature


[1] J.-F. Delhomel, E. Perspicace, Z. Majd, P. Parroche, R. Walczak, *N-{[2-(Piperidin-1-Yl)Phenyl](Phenyl)Methyl}-2-(3-Oxo-3,4-Dihydro-2h-1,4-Benzoxa Zin-7-Yl)Acetamide Derivatives and Related Compounds as Ror-Gamma Modulators for Treating Autoimmune Diseases*, **2018**, WO2018138362A1.

[2] P. C. Meltzer, M. McPhee, B. K. Madras, *Bioorg. Med. Chem. Lett.* **2003**, *13*, 4133–4137.

[3] E. J. Lavoie, J. D. Bauman, A. Parhi, H. Y. Sagong, D. Patel, E. Arnold, K. Das, S. K. Vijayan, *Therapeutic Hydroxypyridinones, Hydroxypyrimidinones and Hydroxypyridazinones*, **2014**, WO2014043252A2.

[4] G. N. Murshudov, P. Skubák, A. A. Lebedev, N. S. Pannu, R. A. Steiner, R. A. Nicholls, M. D. Winn, F. Long, A. A. Vagin, *Acta Crystallogr. D Biol. Crystallogr.* **2011**, *67*, 355–367.

[5] V. B. Chen, W. B. Arendall, J. J. Headd, D. A. Keedy, R. M. Immormino, G. J. Kapral, L. W. Murray, J. S. Richardson, D. C. Richardson, *Acta Crystallogr. D Biol. Crystallogr.* **2010**, *66*, 12–21.

## $^{1}$H, $^{13}$C and APT NMR spectra

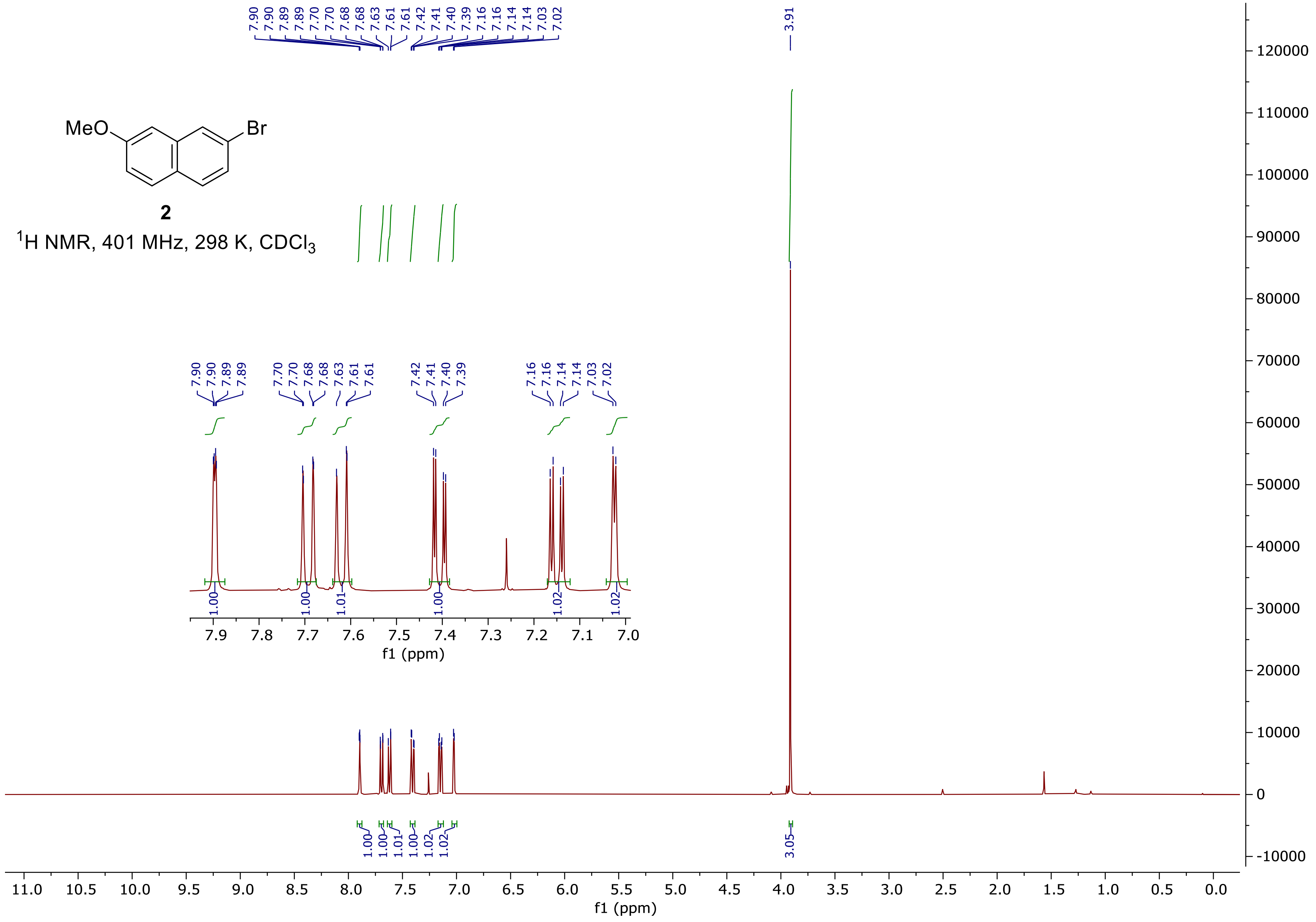

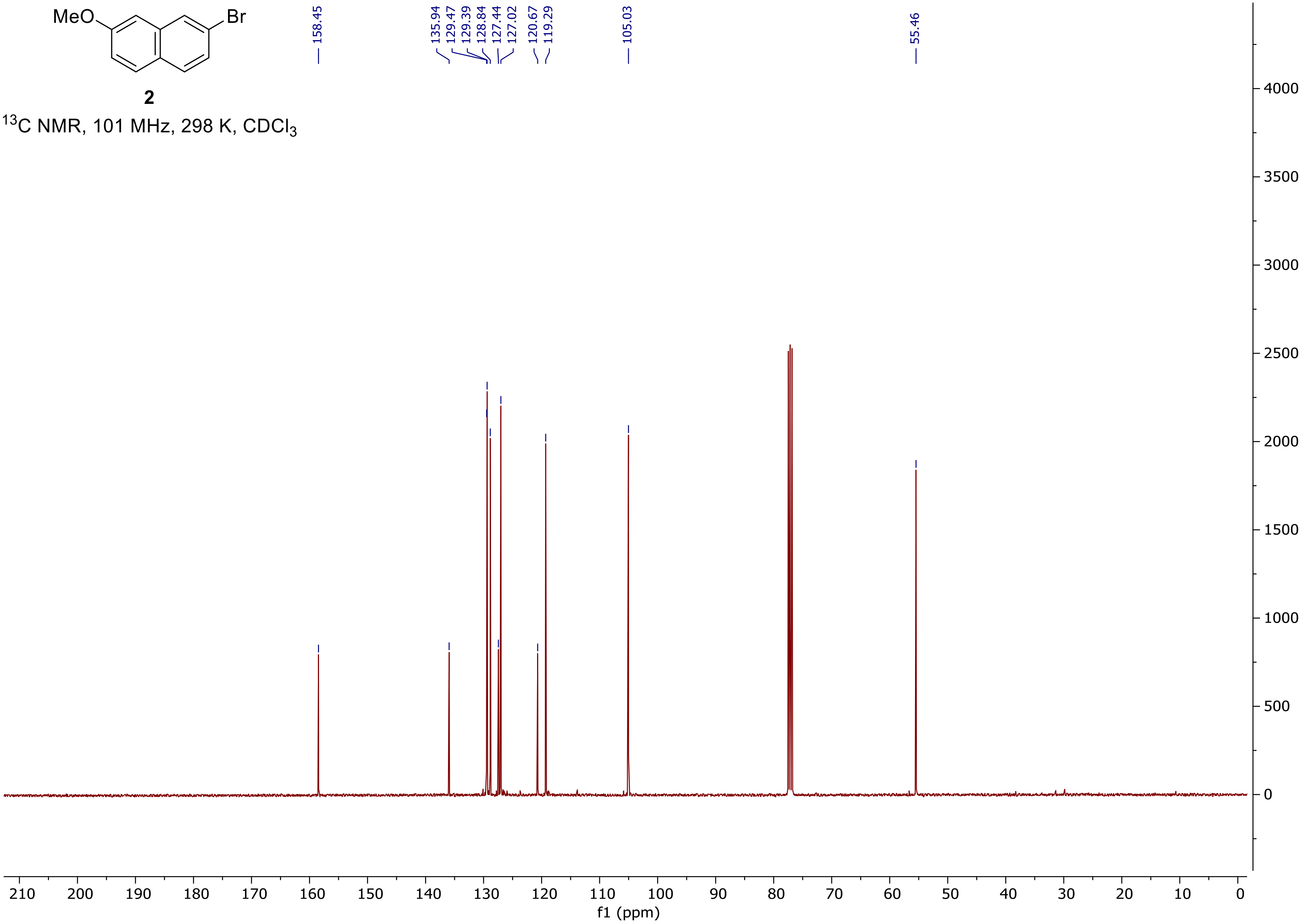


**2**

$^{13}$C NMR, 101 MHz, 298 K, $CDCl_3$

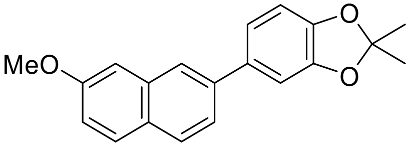

**3**

$^{1}$H NMR, 401 MHz, 298 K, $CDCl_3$

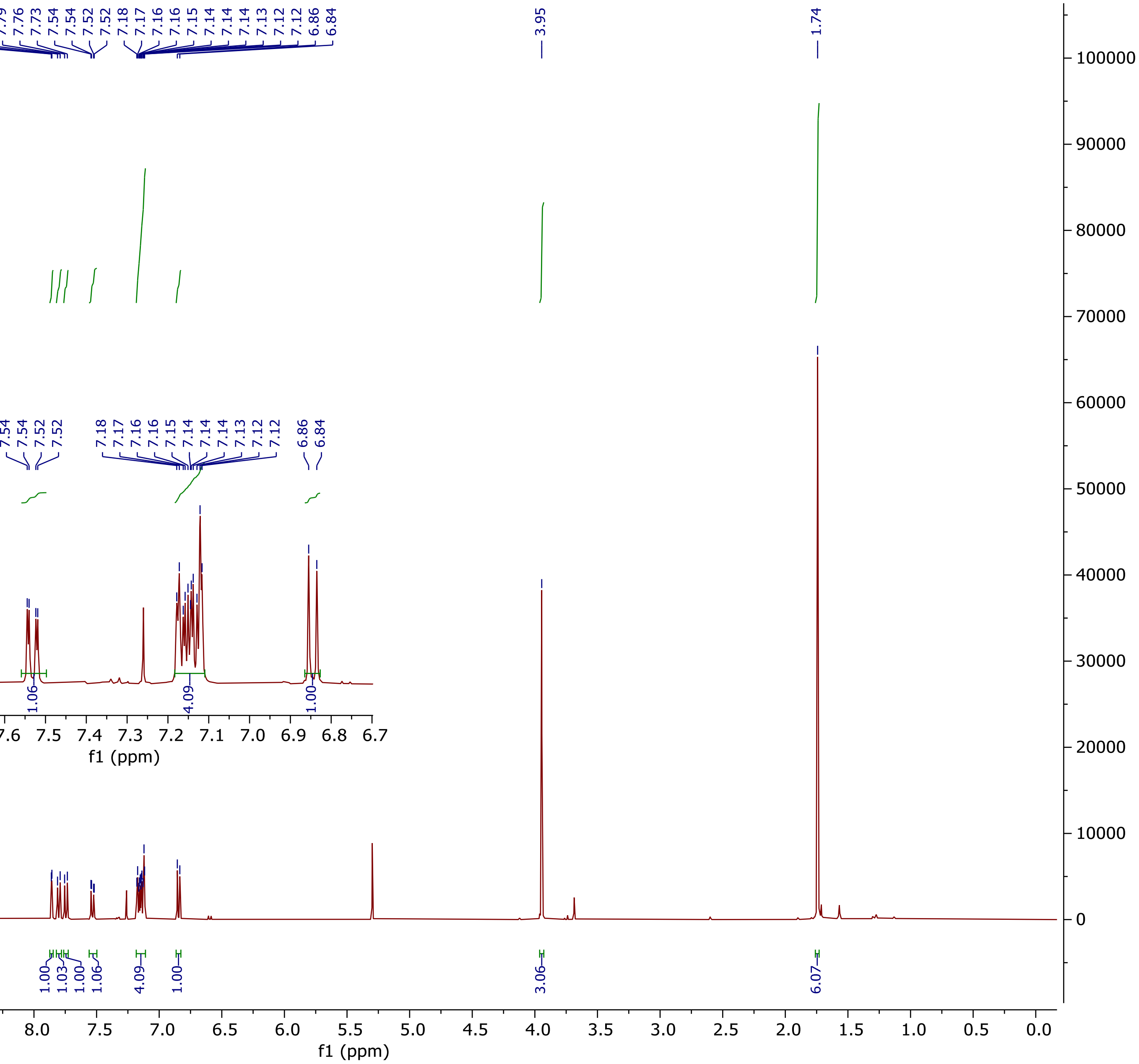

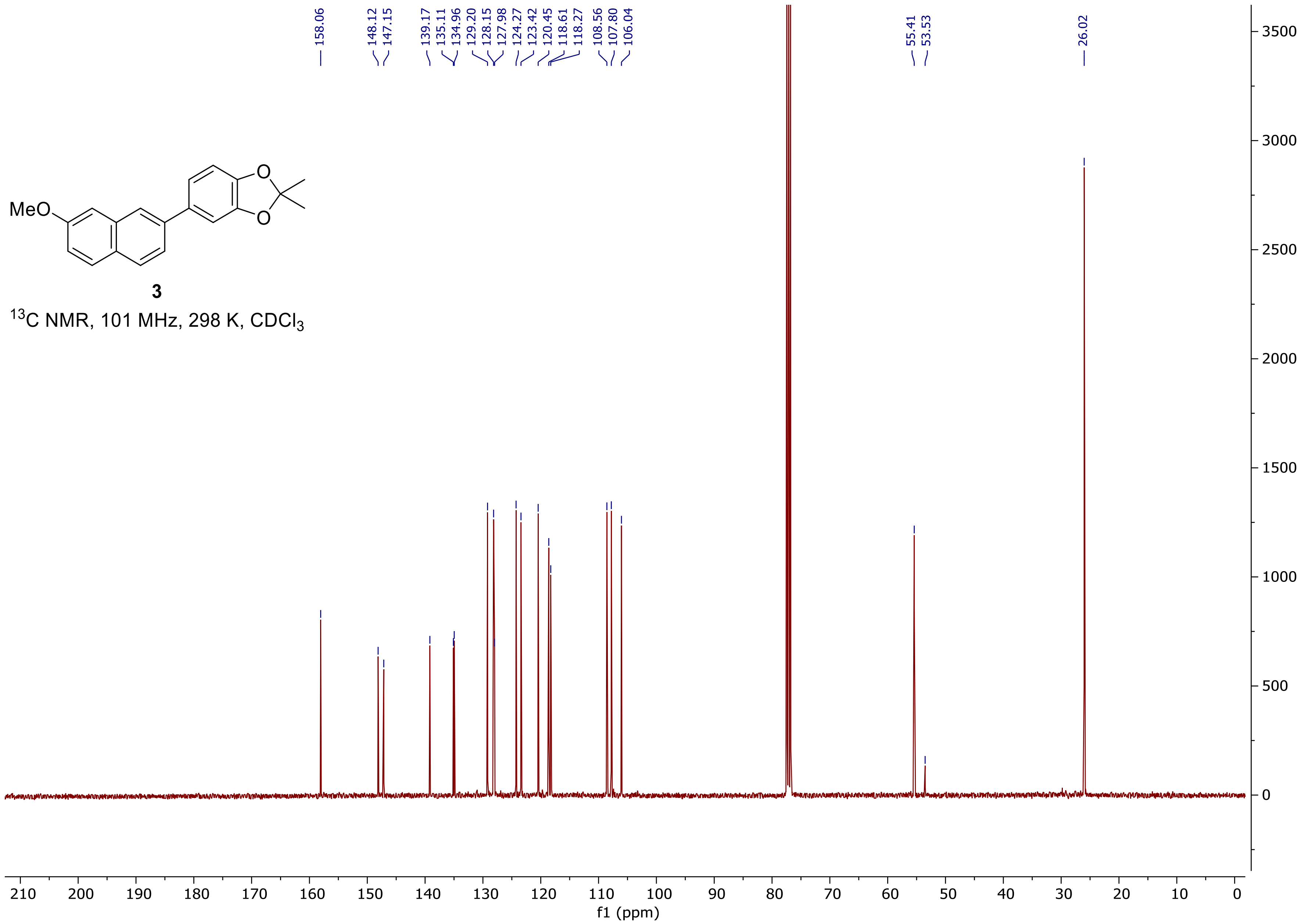

158.06
148.12
147.15
139.17
135.11
134.96
129.20
128.15
127.98
124.27
123.42
120.45
118.61
118.27
108.56
107.80
106.04
55.41
53.53
26.02
MeO
O
O
3
$^{13}$C NMR, 101 MHz, 298 K, CDCl$_3$
f1 (ppm)

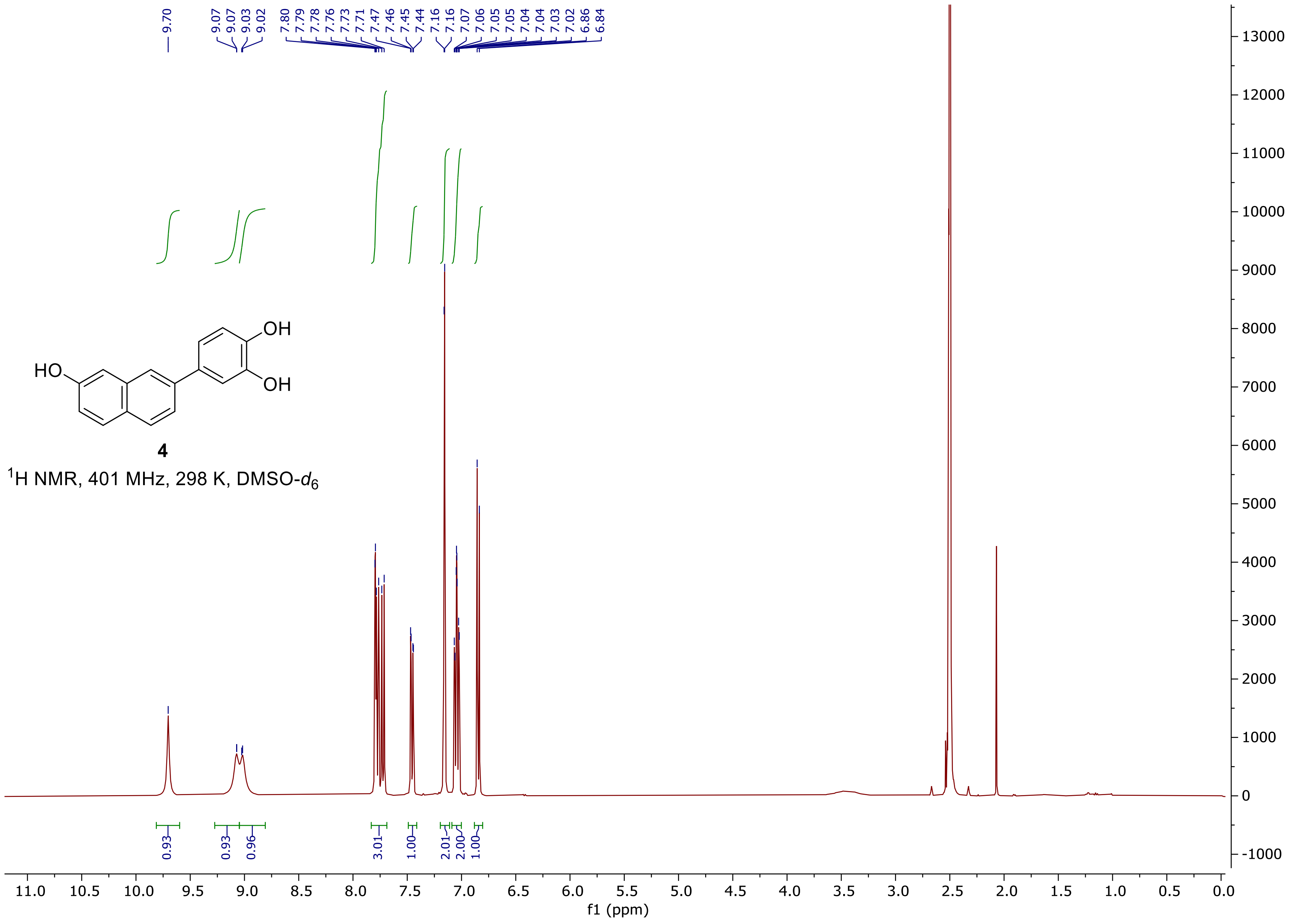

4
$^1$H NMR, 401 MHz, 298 K, DMSO-$d_6$
f1 (ppm)

**4**

$^{13}C$ NMR, 101 MHz, 298 K, DMSO-$d_6$

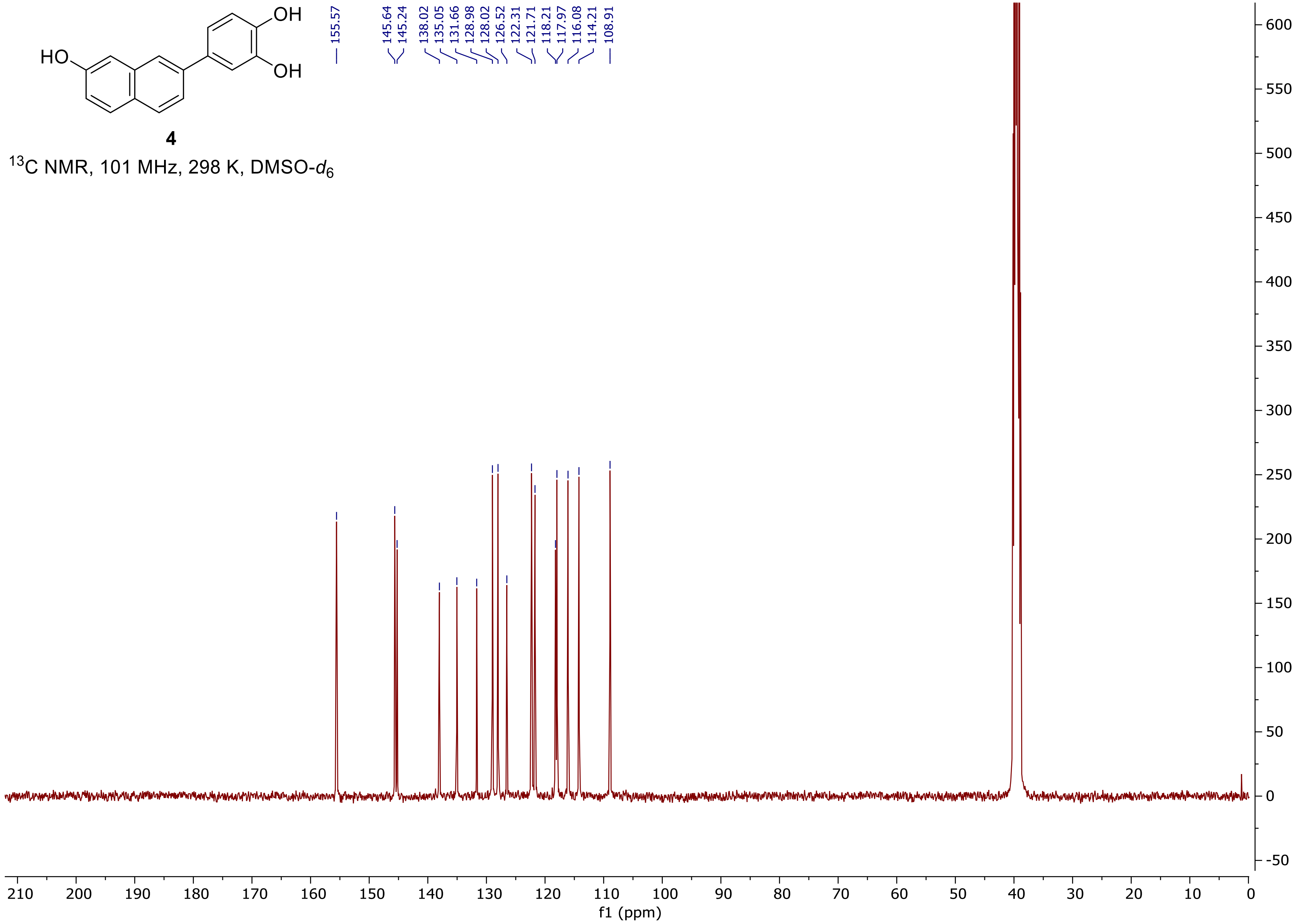

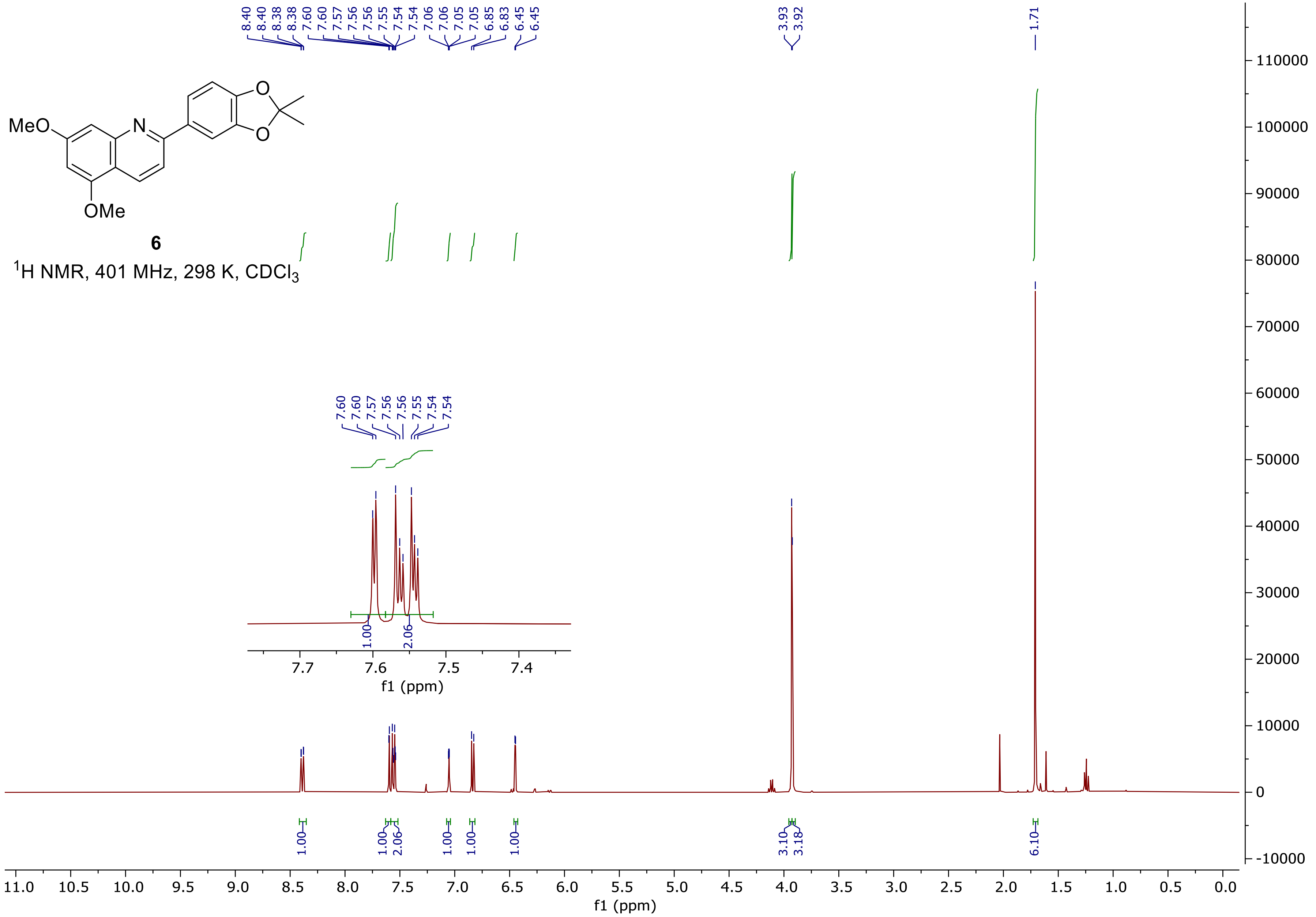


**6**

$^{1}$H NMR, 401 MHz, 298 K, $CDCl_3$

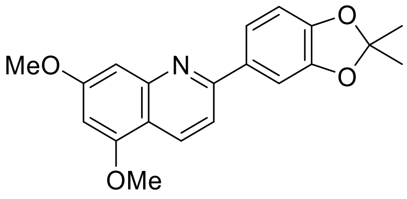

**6**

$^{13}C$ NMR, 101 MHz, 298 K, $CDCl_3$

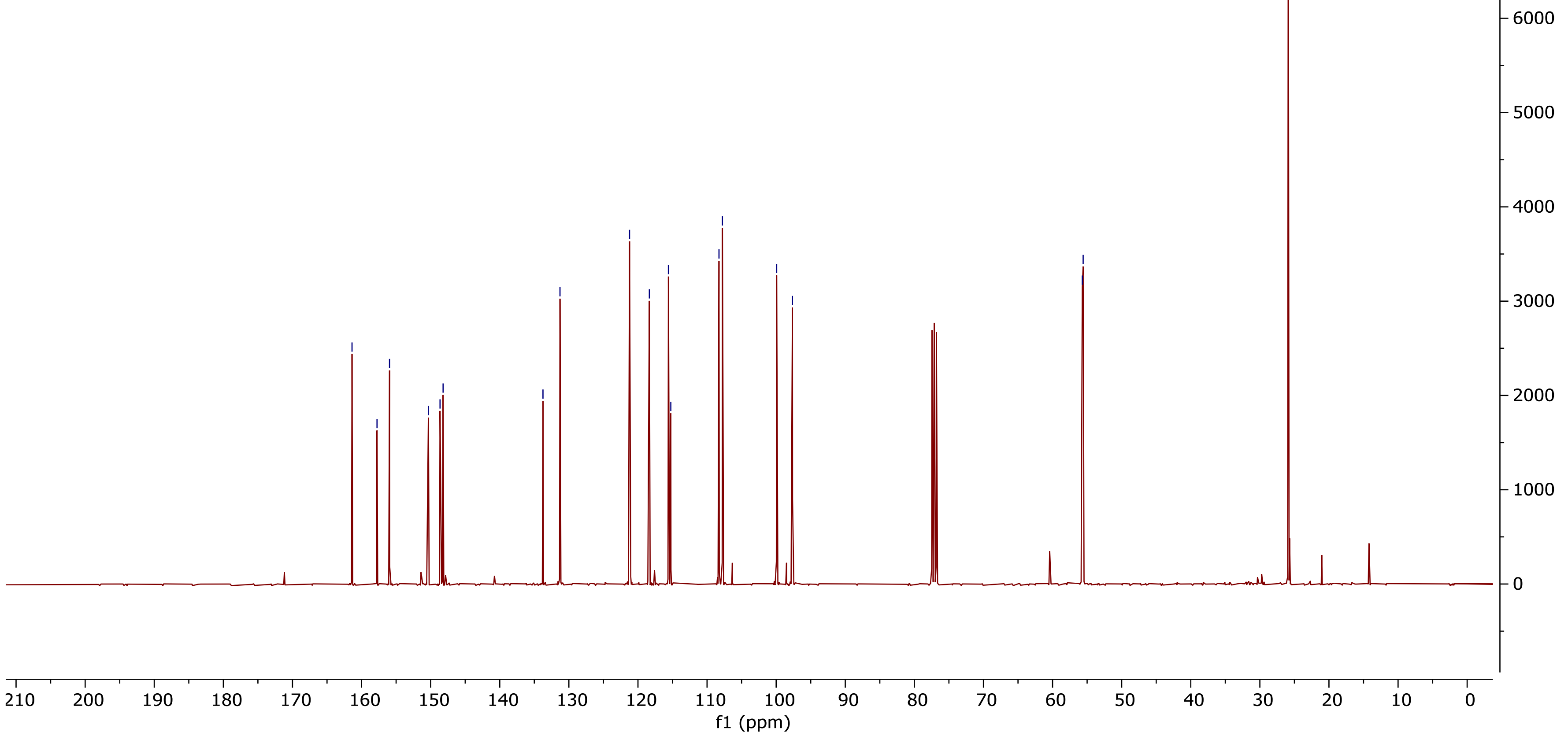

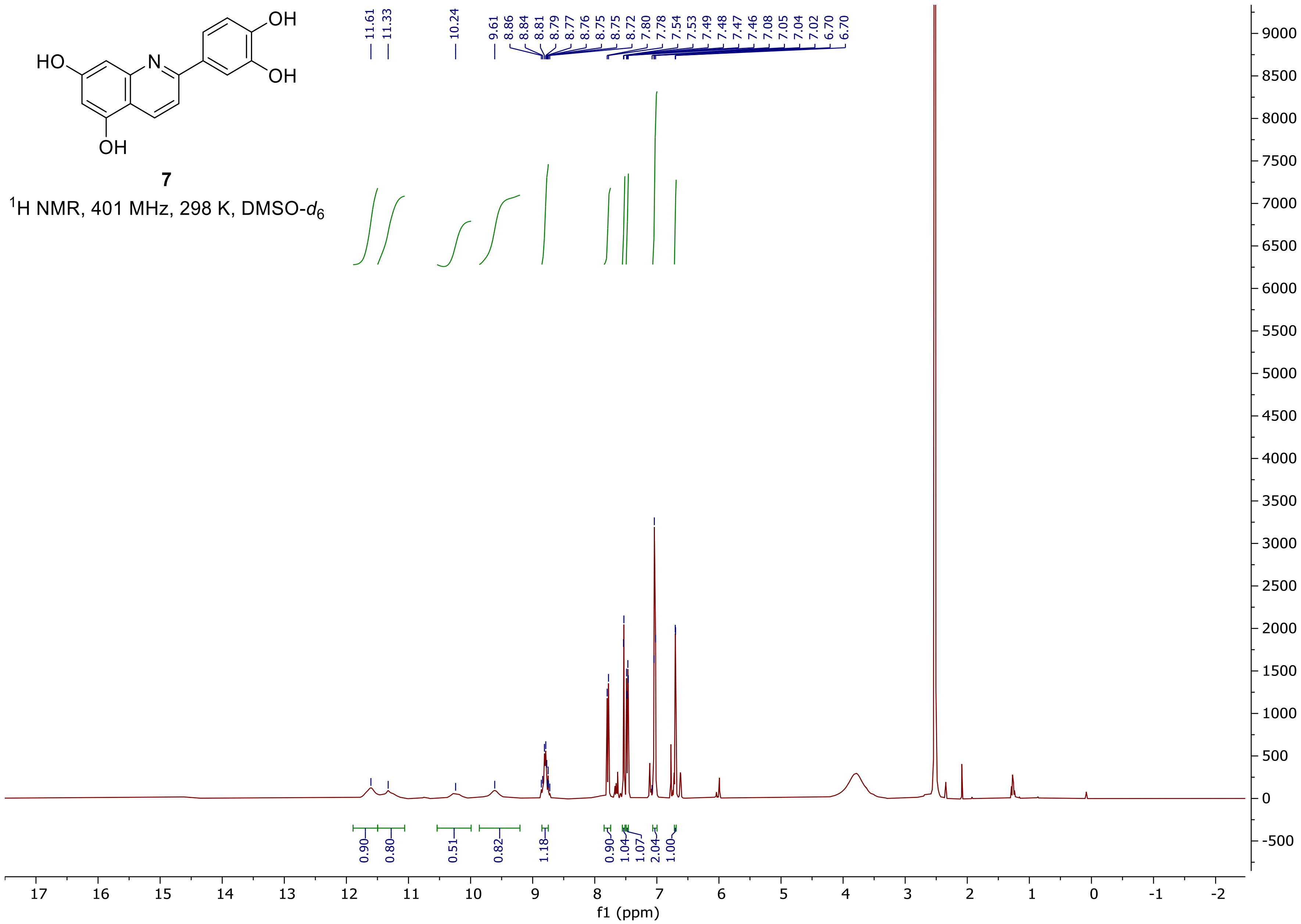
HO
N
OH
OH
OH
7
¹H NMR, 401 MHz, 298 K, DMSO-d6
11.61
11.33
10.24
9.61
8.86
8.84
8.81
8.79
8.77
8.76
8.75
8.75
8.72
7.80
7.78
7.54
7.53
7.49
7.48
7.47
7.46
7.08
7.05
7.04
7.02
6.70
6.70
0.90
0.80
0.51
0.82
1.18
0.90
1.04
1.07
2.04
1.00
f1 (ppm)

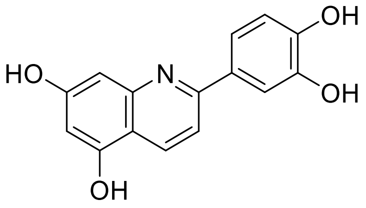


**7**

$^{13}$C NMR, 101 MHz, 298 K, DMSO-$d_6$

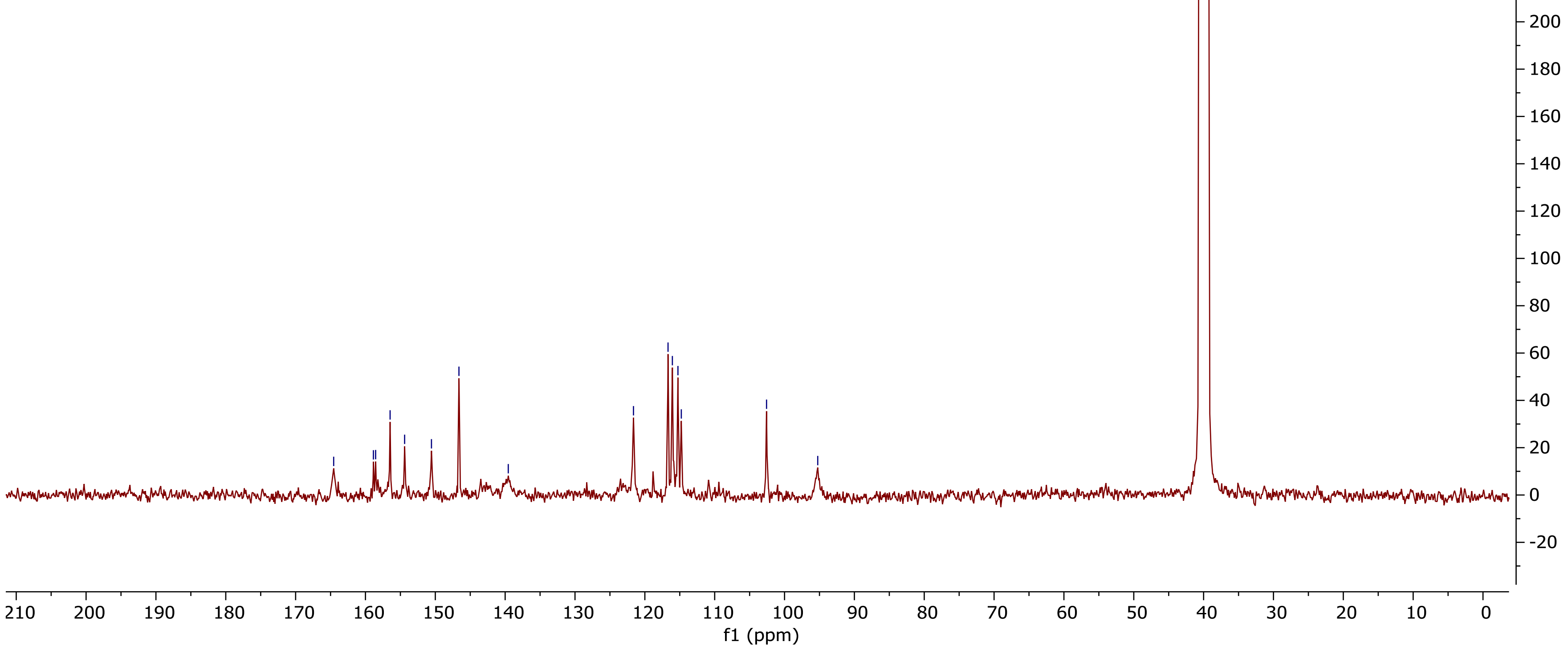

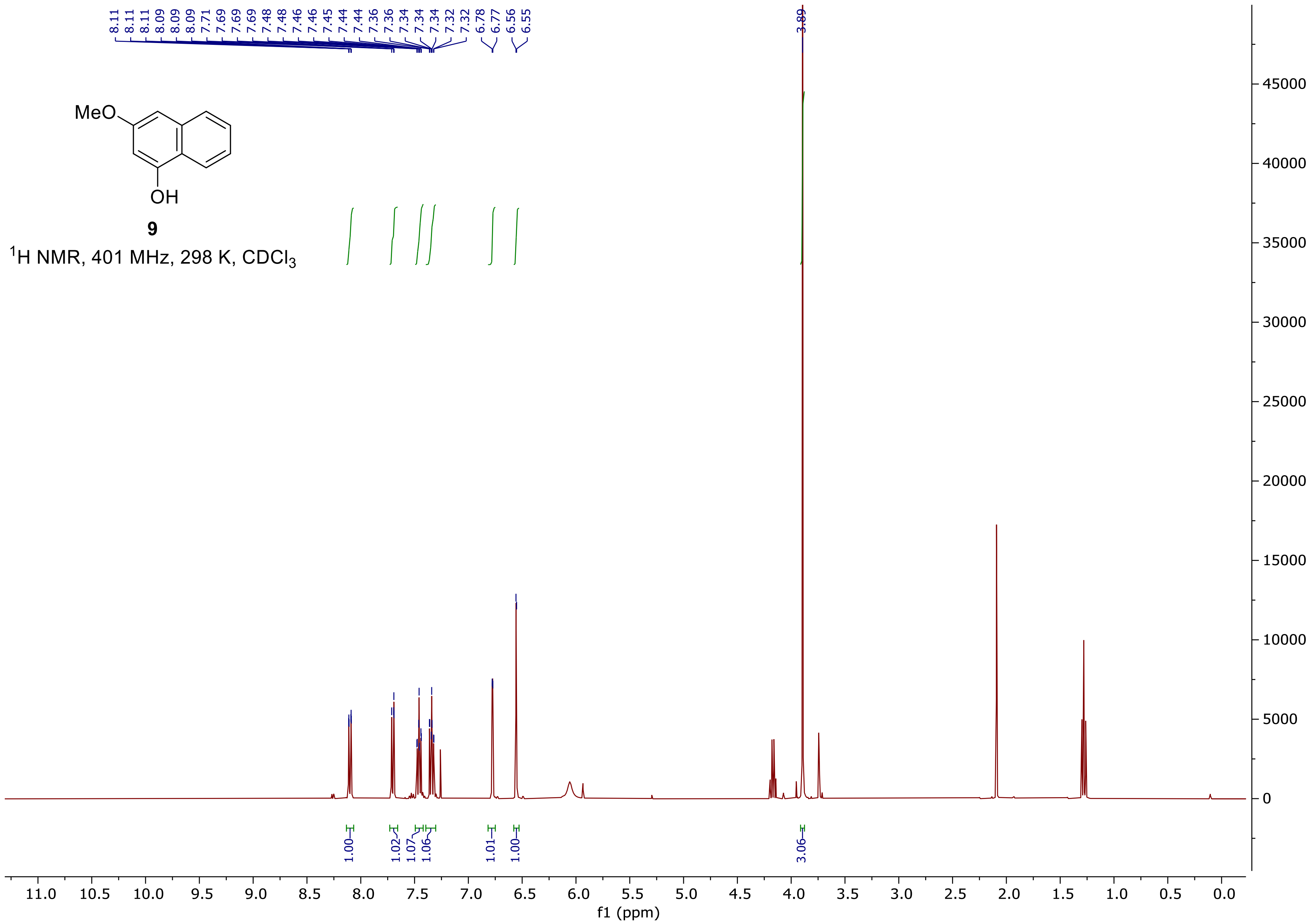

8.11
8.11
8.11
8.09
8.09
8.09
7.71
7.69
7.69
7.69
7.48
7.48
7.46
7.46
7.45
7.44
7.44
7.36
7.36
7.34
7.34
7.34
7.32
7.32
6.78
6.77
6.56
6.55
3.89
MeO
OH
9
1H NMR, 401 MHz, 298 K, CDCl3
1.00
1.02
1.07
1.06
1.01
1.00
3.06
f1 (ppm)

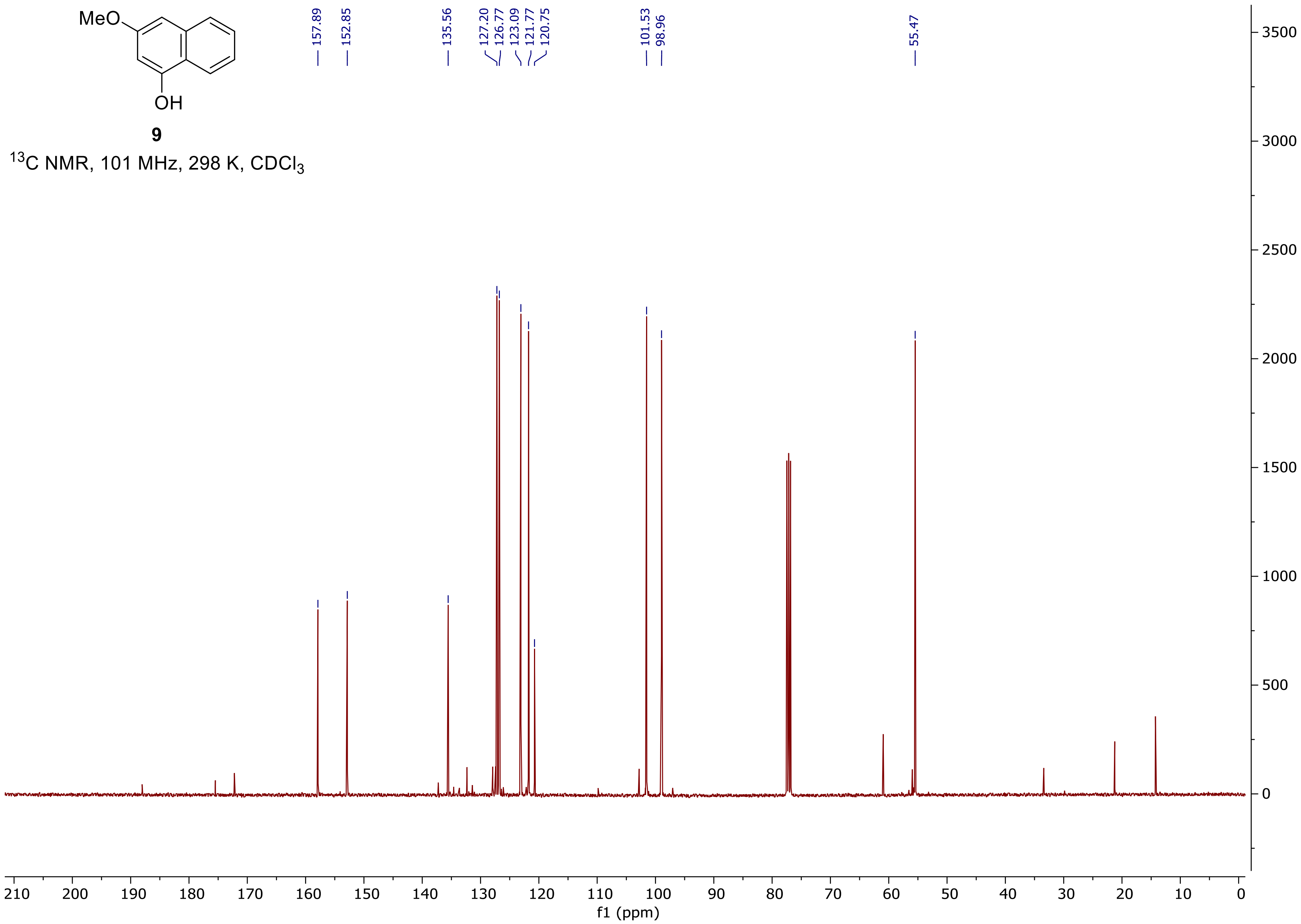
MeO
OH
9
$^{13}C$ NMR, 101 MHz, 298 K, $CDCl_3$
157.89
152.85
135.56
127.20
126.77
123.09
121.77
120.75
101.53
98.96
55.47
f1 (ppm)

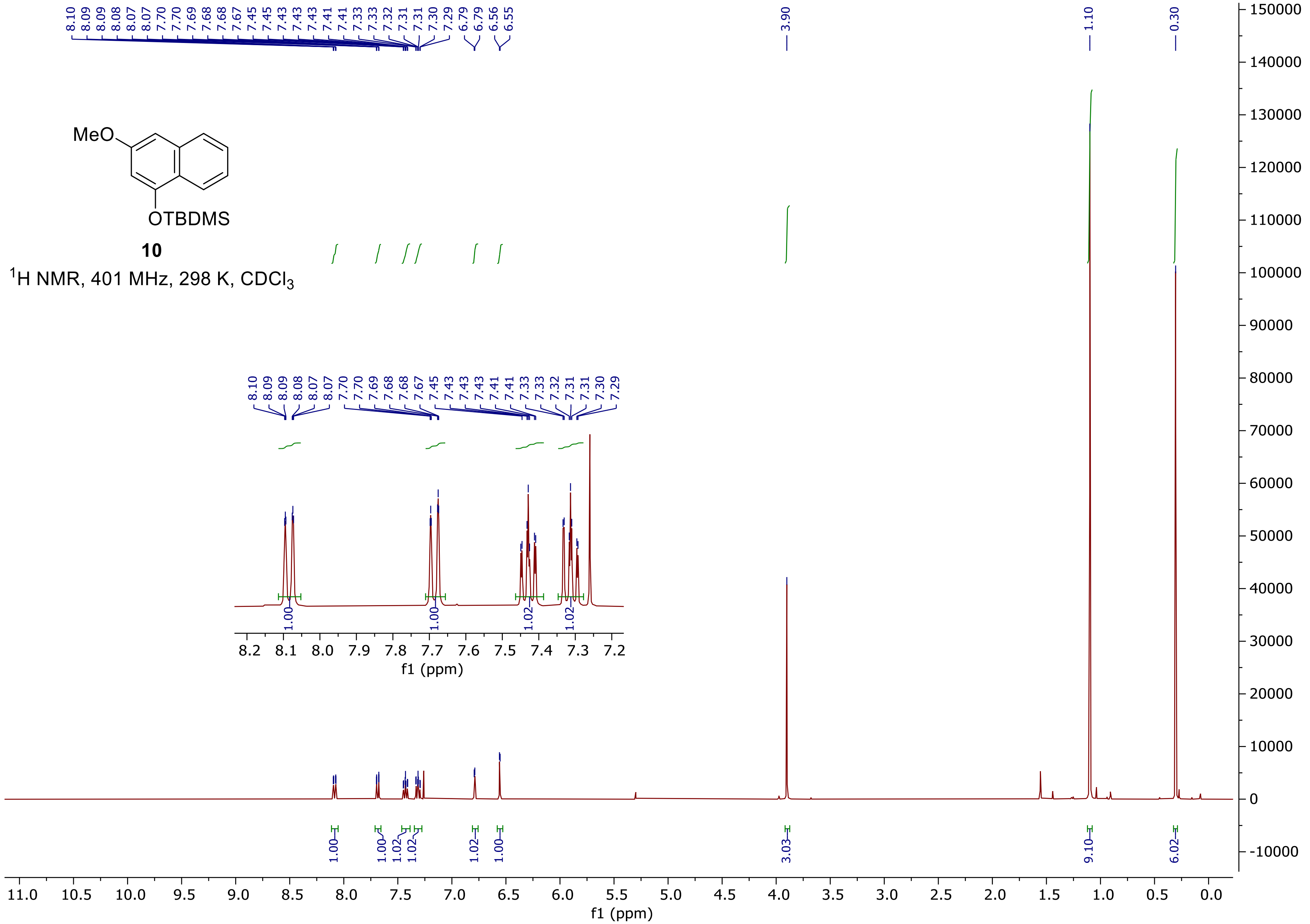
MeO
OTBDMS
10
$^1$H NMR, 401 MHz, 298 K, $CDCl_3$
f1 (ppm)

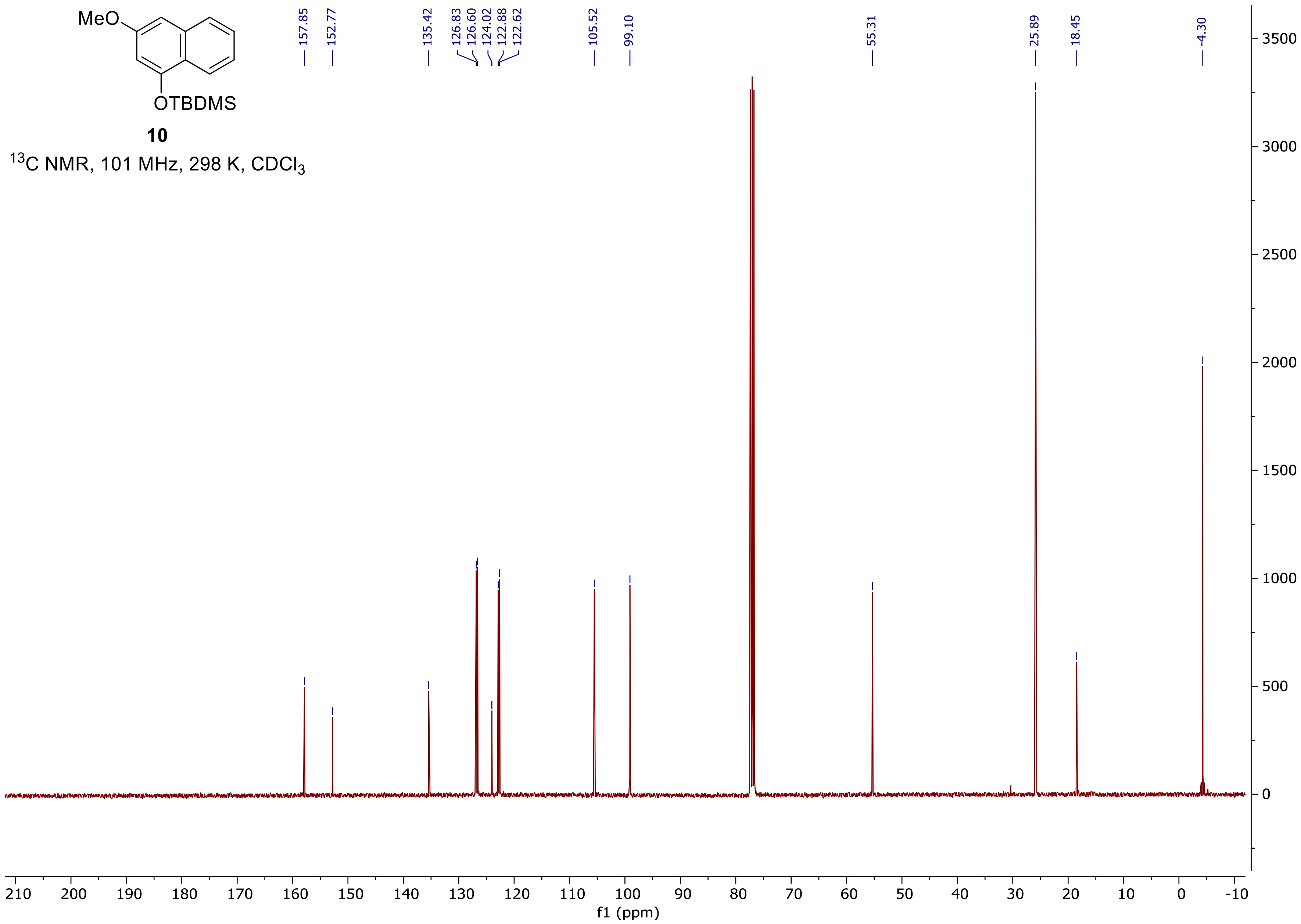

MeO
OTBDMS
10
$^{13}$C NMR, 101 MHz, 298 K, $CDCl_3$
157.85
152.77
135.42
126.83
126.60
124.02
122.88
122.62
105.52
99.10
55.31
25.89
18.45
-4.30
f1 (ppm)

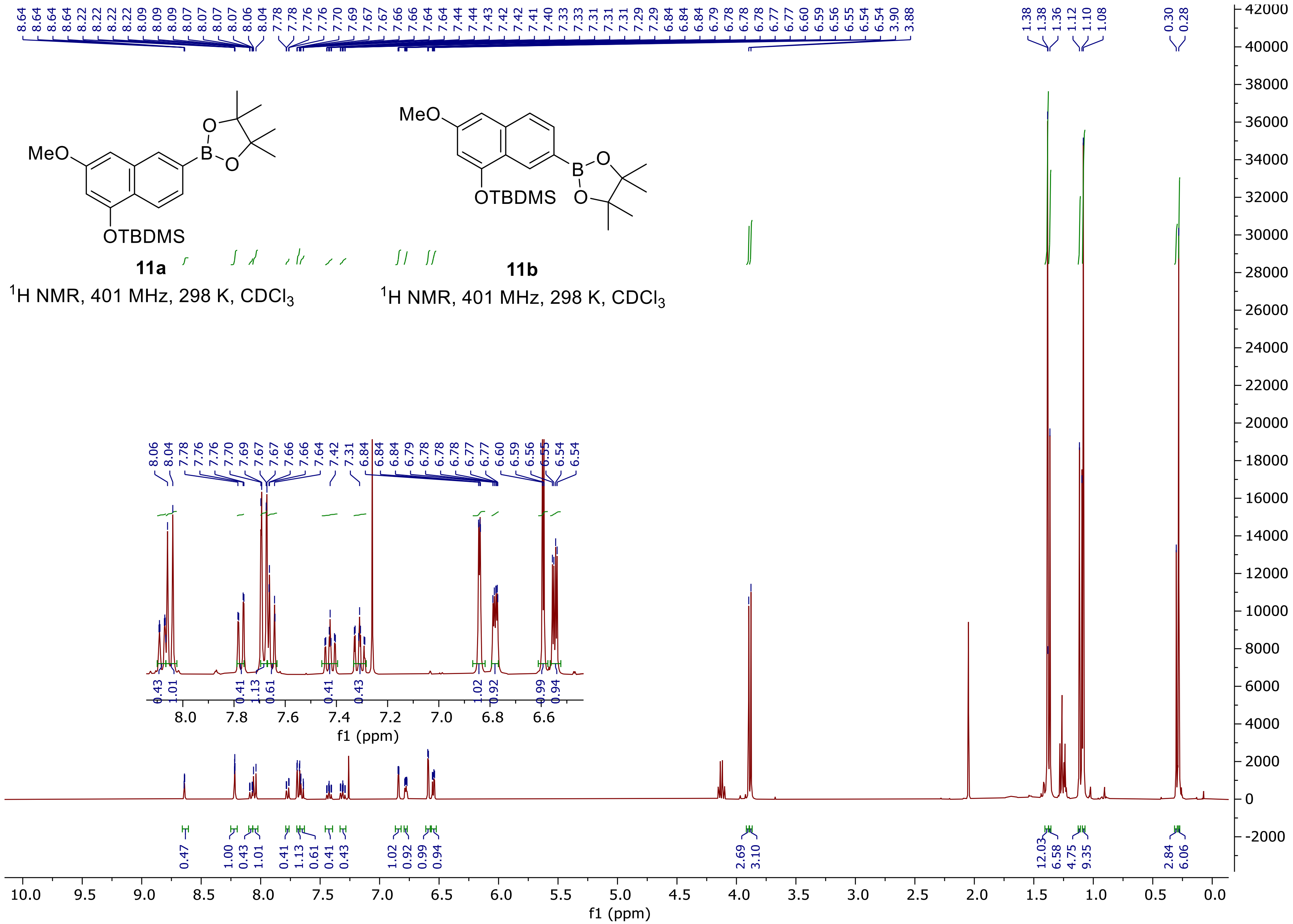
11a
1H NMR, 401 MHz, 298 K, CDCl3
11b
1H NMR, 401 MHz, 298 K, CDCl3
f1 (ppm)

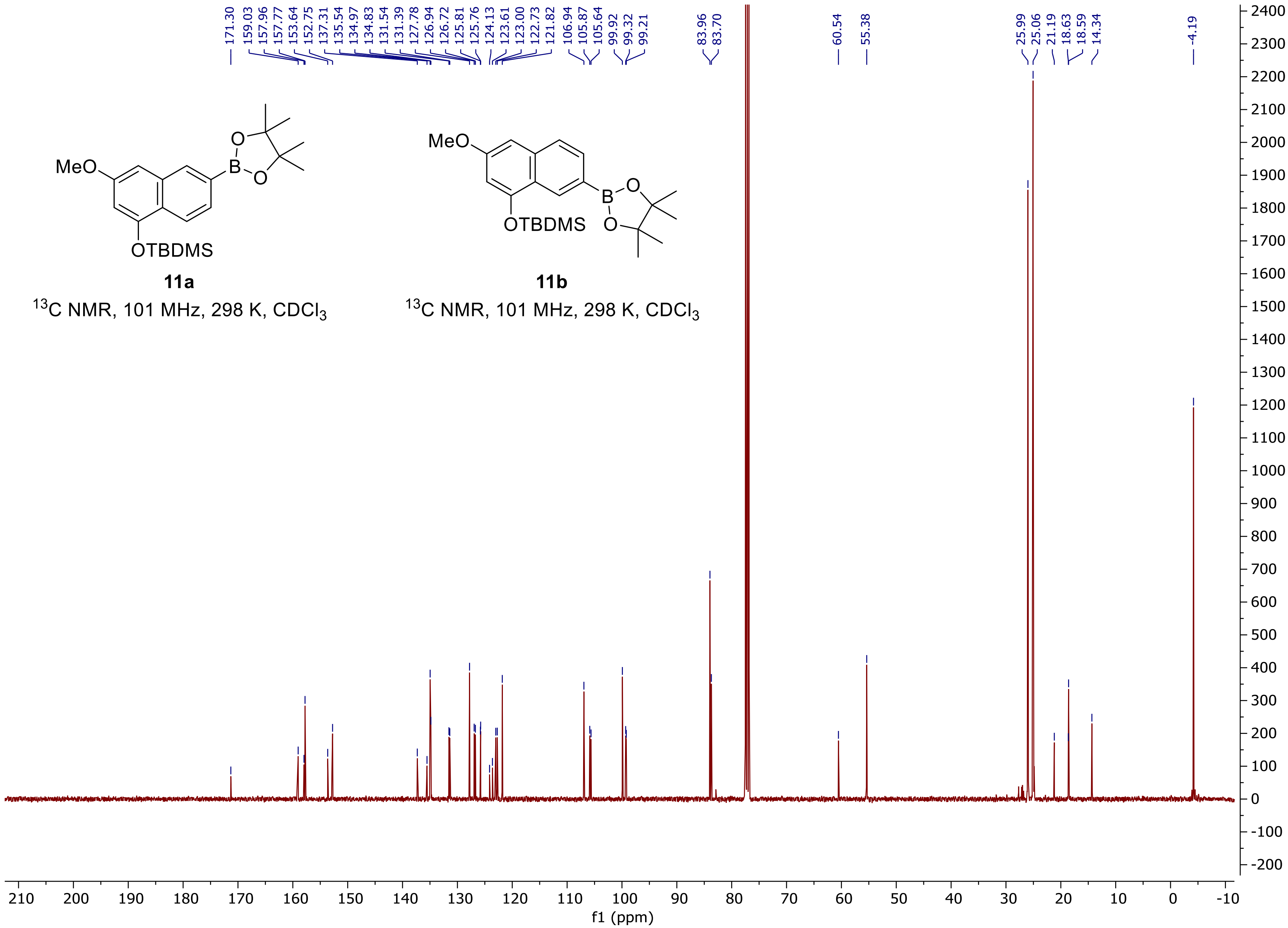

171.30
159.03
157.96
157.77
153.64
152.75
137.31
135.54
134.97
134.83
131.54
131.39
127.78
126.94
126.72
125.81
125.76
124.13
123.61
123.00
122.73
121.82
106.94
105.87
105.64
99.92
99.32
99.21
83.96
83.70
60.54
55.38
25.99
25.06
21.19
18.63
18.59
14.34
-4.19
MeO
OTBDMS
11a
$^{13}$C NMR, 101 MHz, 298 K, CDCl$_3$
MeO
OTBDMS
11b
$^{13}$C NMR, 101 MHz, 298 K, CDCl$_3$
f1 (ppm)

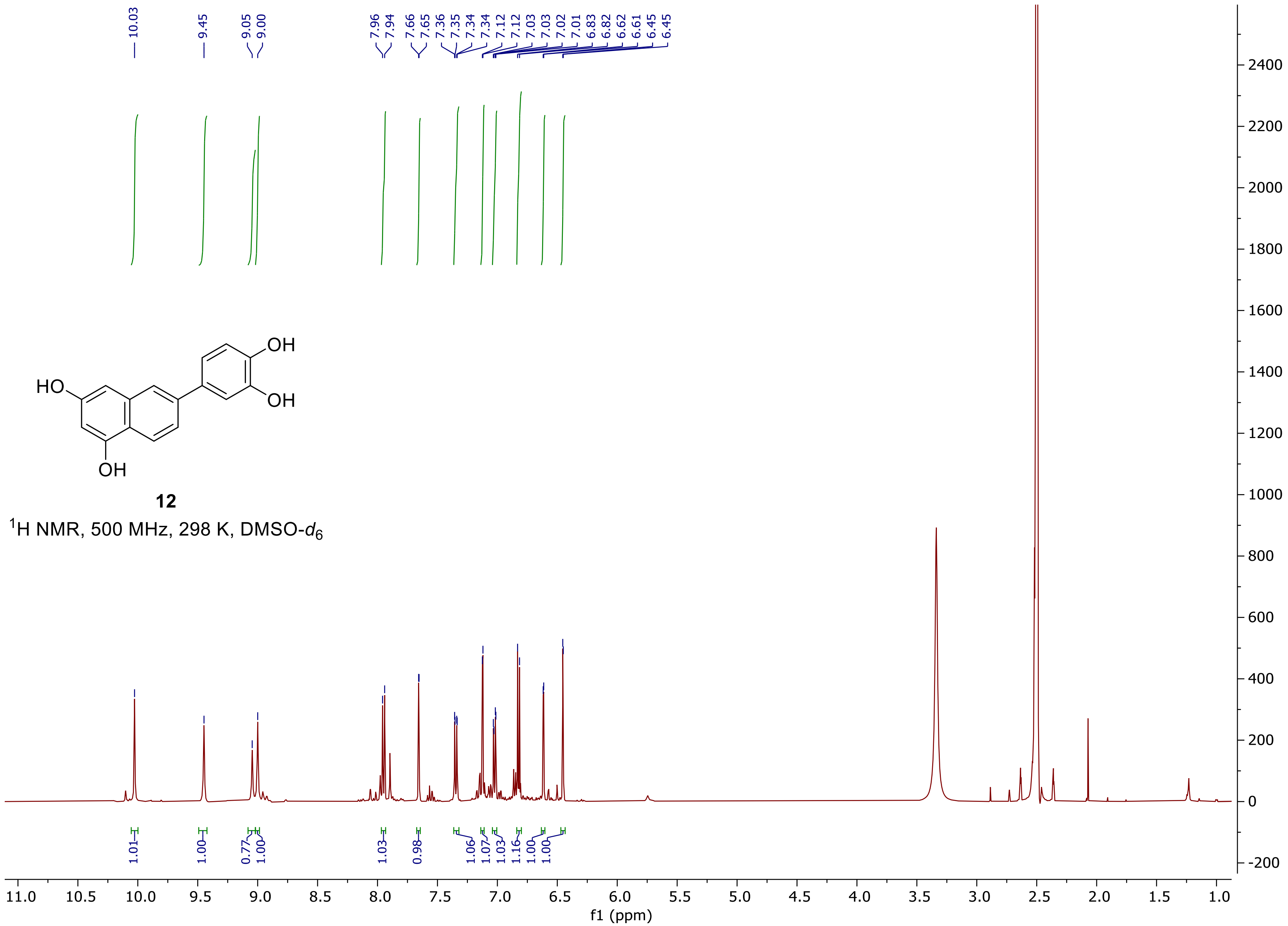


**12**

$^{1}$H NMR, 500 MHz, 298 K, DMSO-$d_6$

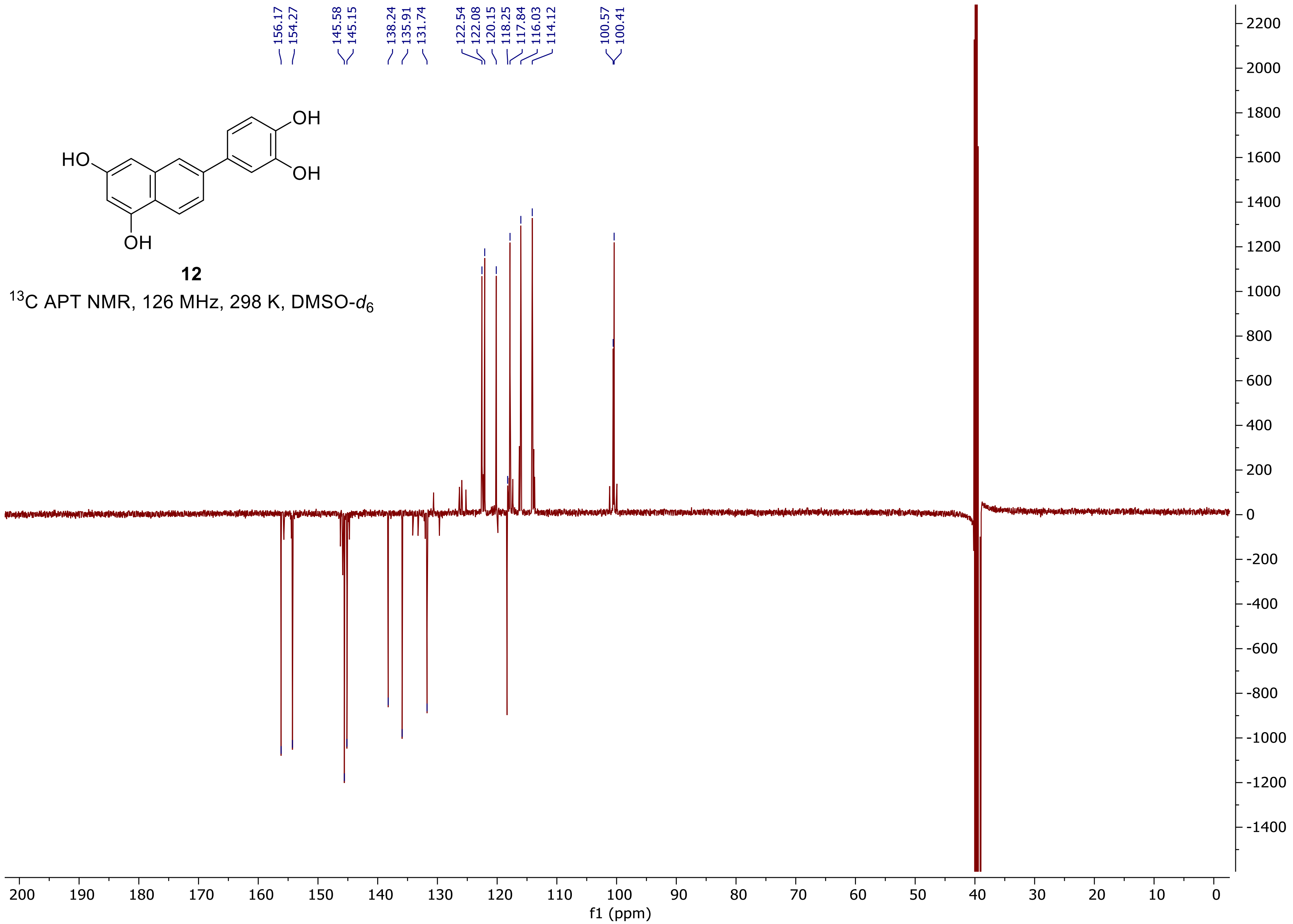


**12**

$^{13}$C APT NMR, 126 MHz, 298 K, DMSO-$d_6$

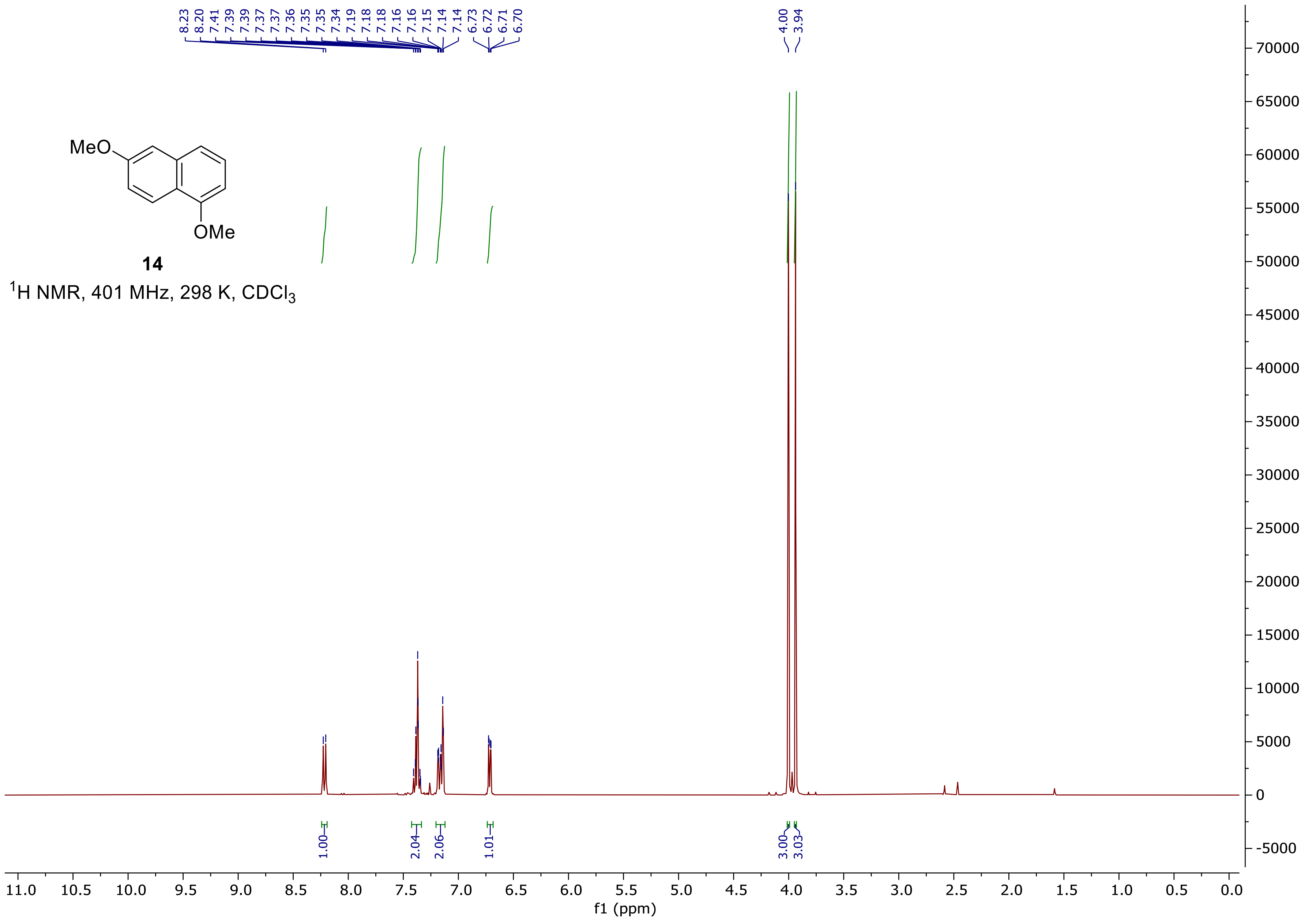
8.23 8.20 7.41 7.39 7.39 7.37 7.37 7.36 7.35 7.35 7.34 7.19 7.18 7.18 7.16 7.16 7.15 7.14 7.14 6.73 6.72 6.71 6.70
4.00 3.94
MeO
OMe
14
$^{1}$H NMR, 401 MHz, 298 K, $CDCl_3$
1.00 2.04 2.06 1.01 3.00 3.03
f1 (ppm)

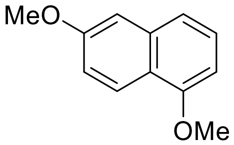

**14**

$^{13}C$ NMR, 101 MHz, 298 K, $CDCl_3$

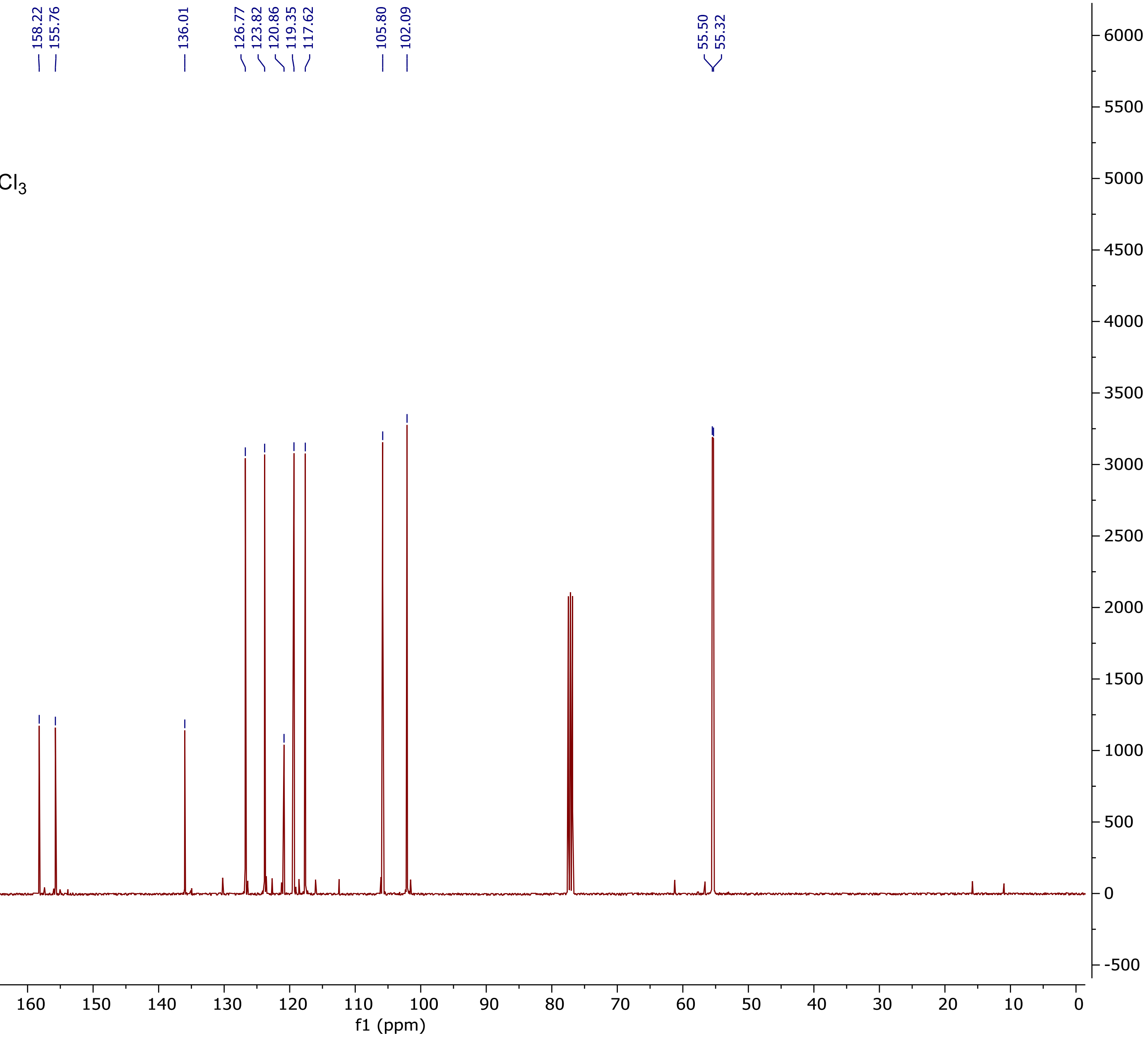

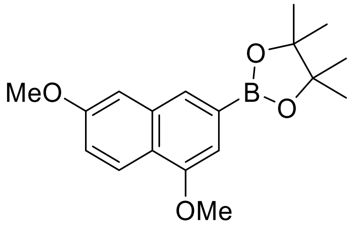


**15**

$^{1}$H NMR, 401 MHz, 298 K, $CDCl_3$

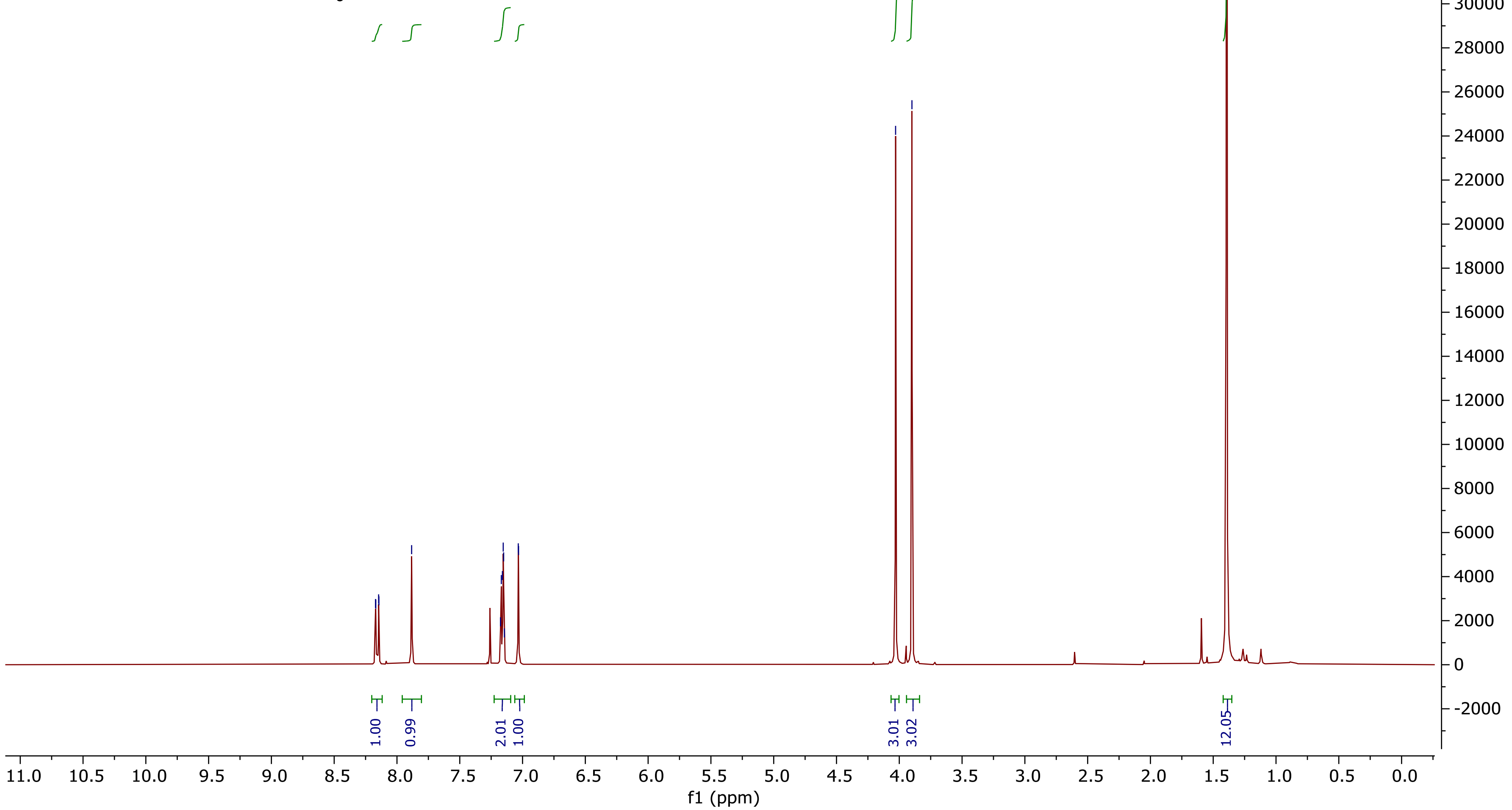

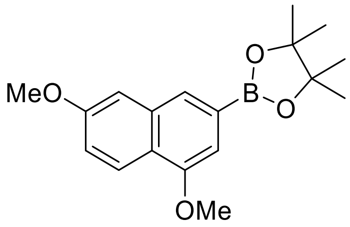

**15**

$^{13}C$ NMR, 101 MHz, 298 K, $CDCl_3$

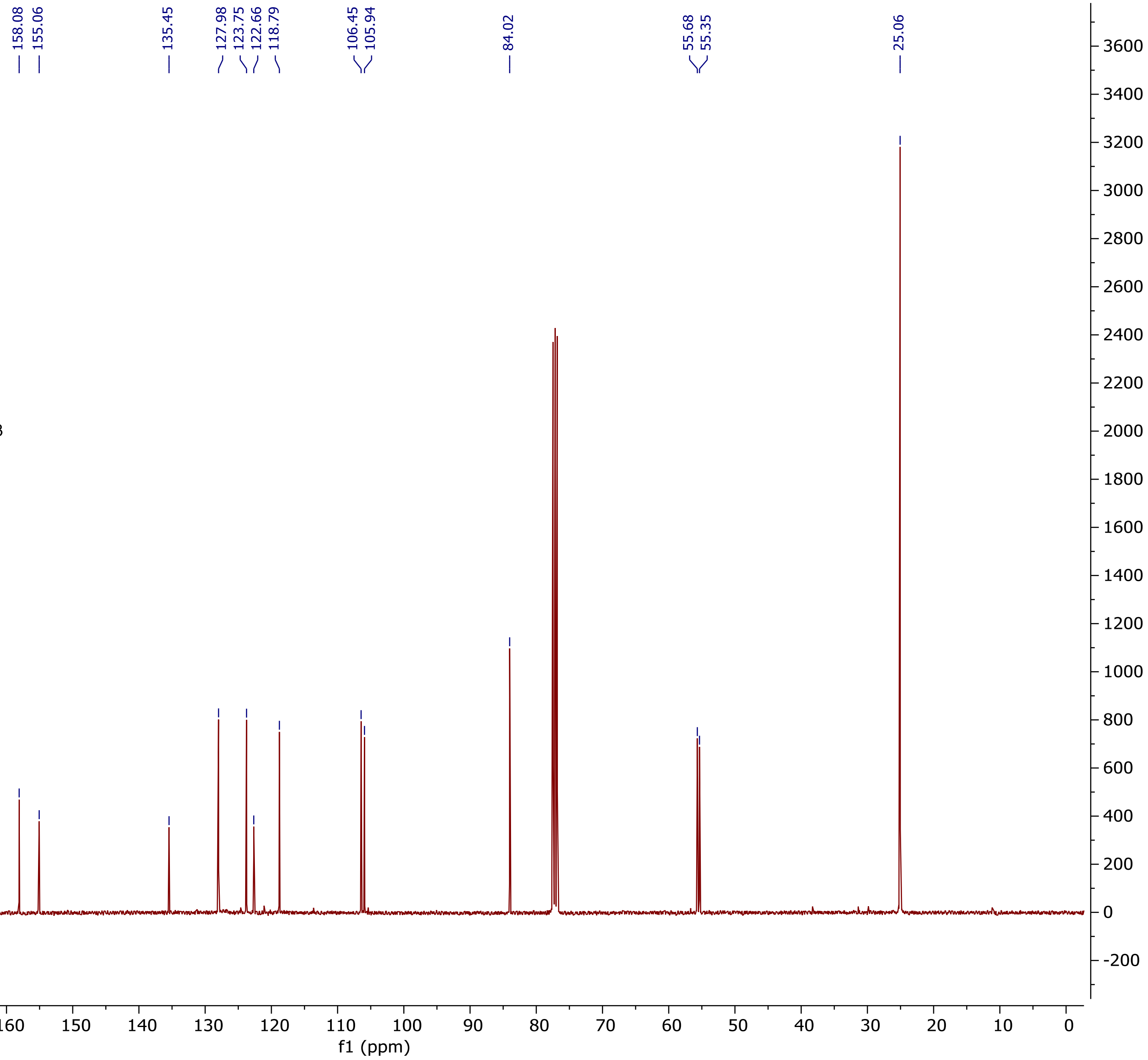

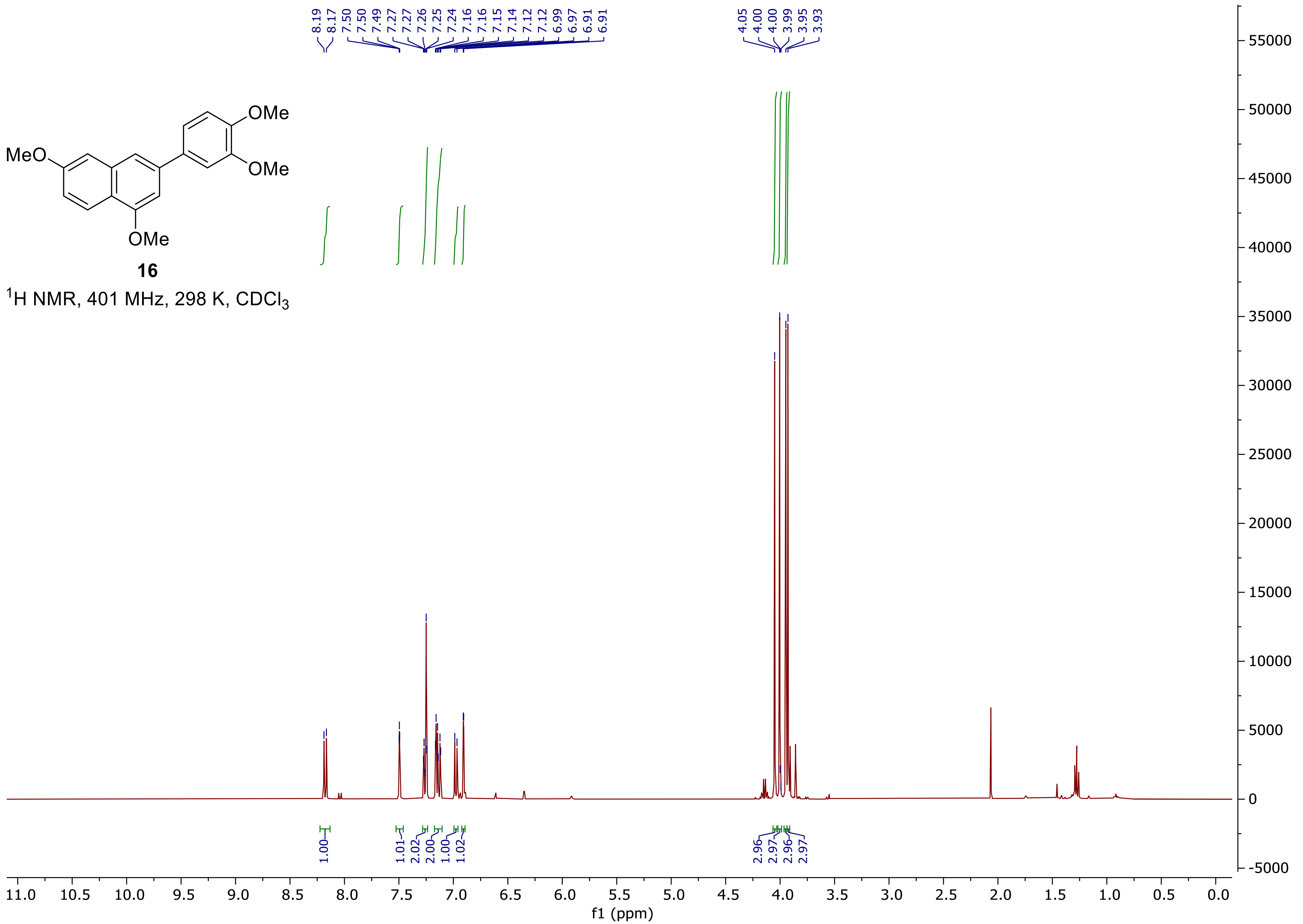
MeO
OMe
OMe
OMe
16
$^1$H NMR, 401 MHz, 298 K, CDCl$_3$
8.19
8.17
7.50
7.49
7.27
7.27
7.26
7.25
7.24
7.16
7.16
7.15
7.14
7.12
7.12
6.99
6.97
6.91
6.91
4.05
4.00
4.00
3.99
3.95
3.93
1.00
1.01
2.02
2.00
1.00
1.02
2.96
2.97
2.96
2.97
f1 (ppm)

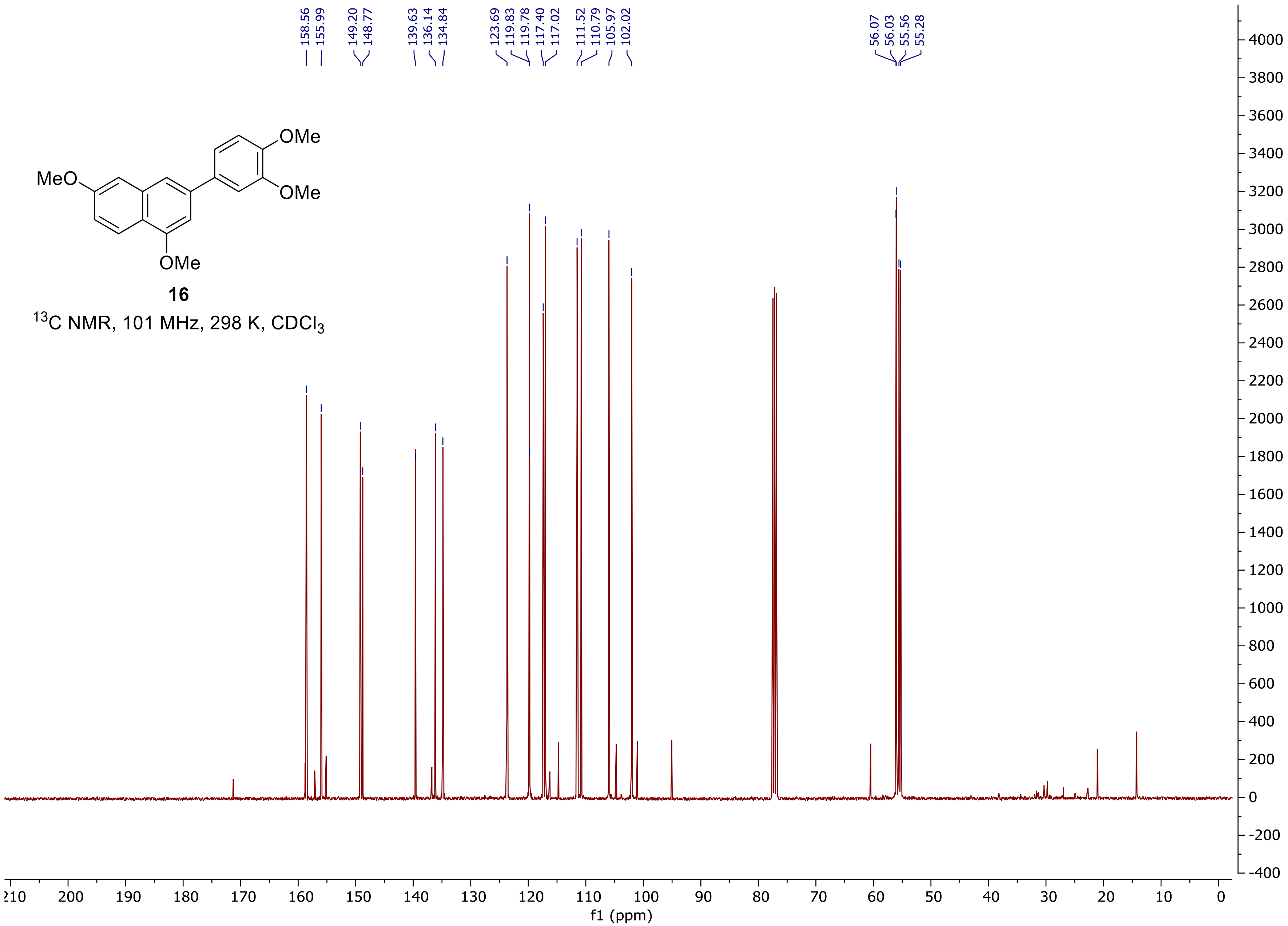
158.56
155.99
149.20
148.77
139.63
136.14
134.84
123.69
119.83
119.78
117.40
117.02
111.52
110.79
105.97
102.02
56.07
56.03
55.56
55.28
MeO
OMe
OMe
OMe
16
$^{13}$C NMR, 101 MHz, 298 K, $CDCl_3$
f1 (ppm)

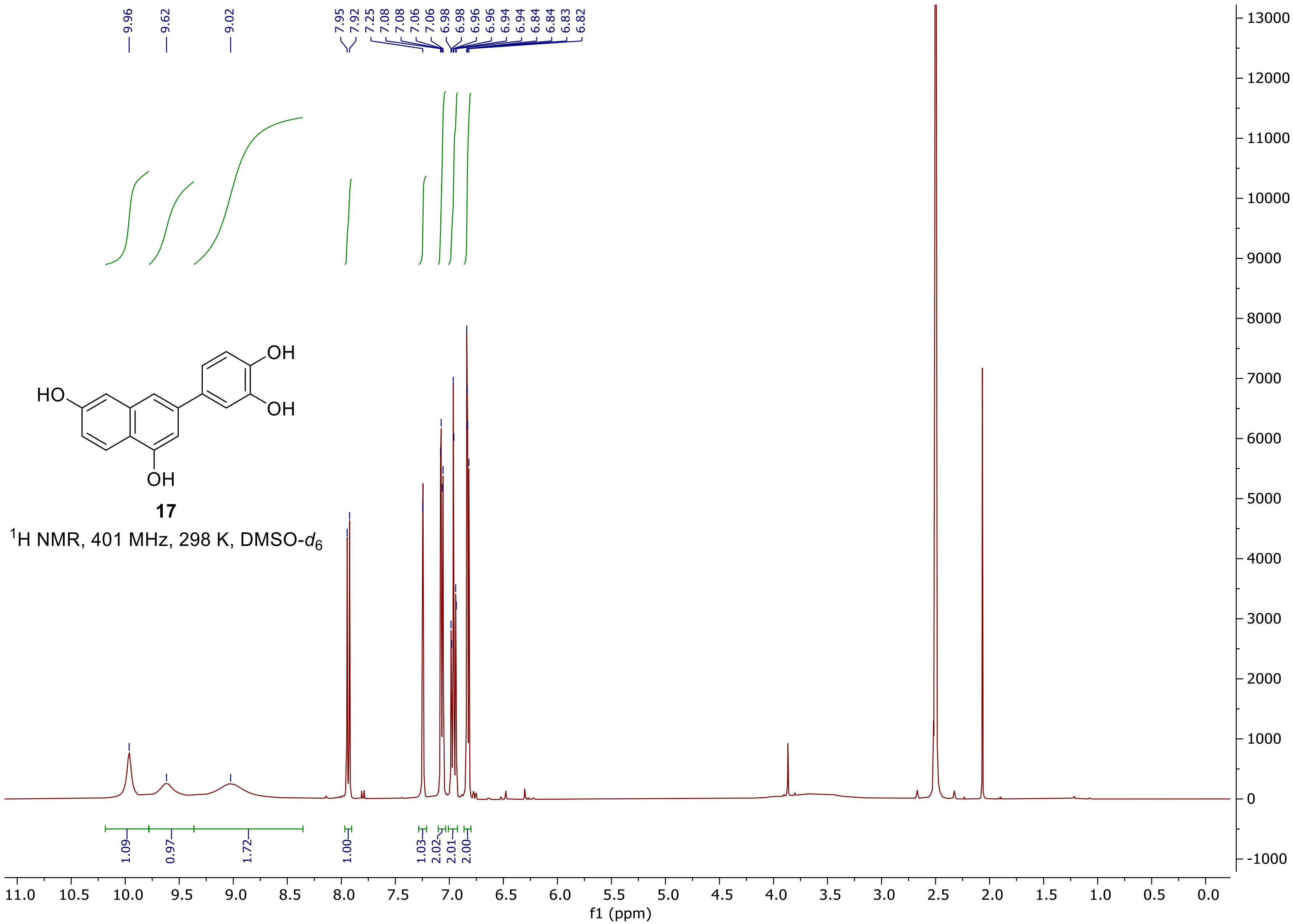
9.96
9.62
9.02
7.95
7.92
7.25
7.08
7.08
7.06
7.06
6.98
6.98
6.96
6.96
6.94
6.94
6.84
6.84
6.83
6.82
HO
OH
OH
OH
17
1H NMR, 401 MHz, 298 K, DMSO-d6
1.09
0.97
1.72
1.00
1.03
2.02
2.01
2.00
f1 (ppm)

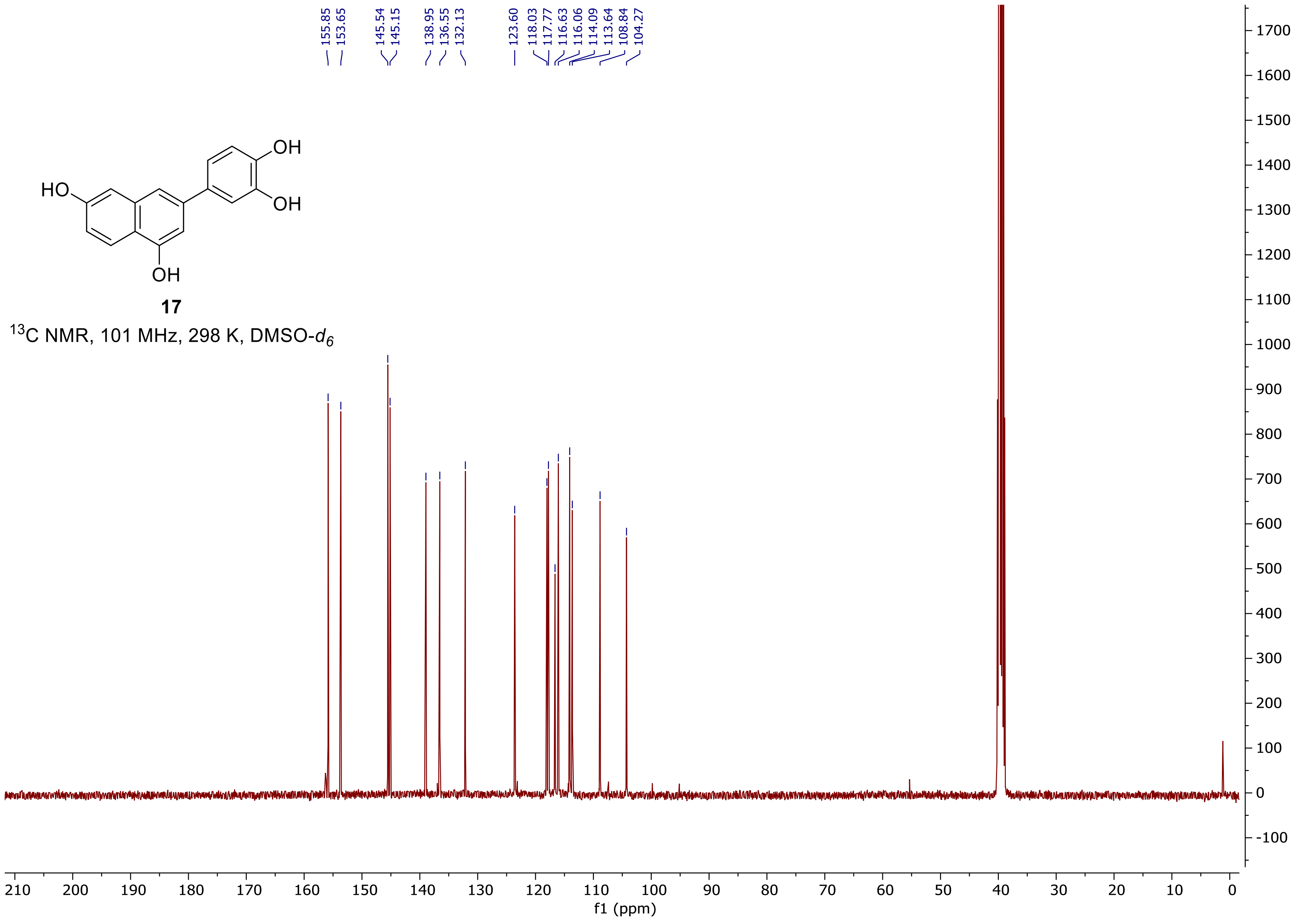


**17**

$^{13}$C NMR, 101 MHz, 298 K, DMSO-$d_6$

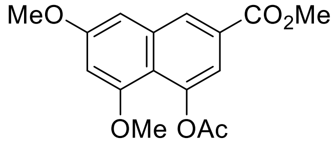

**19**

$^1H$ NMR, 401 MHz, 298 K, $CDCl_3$

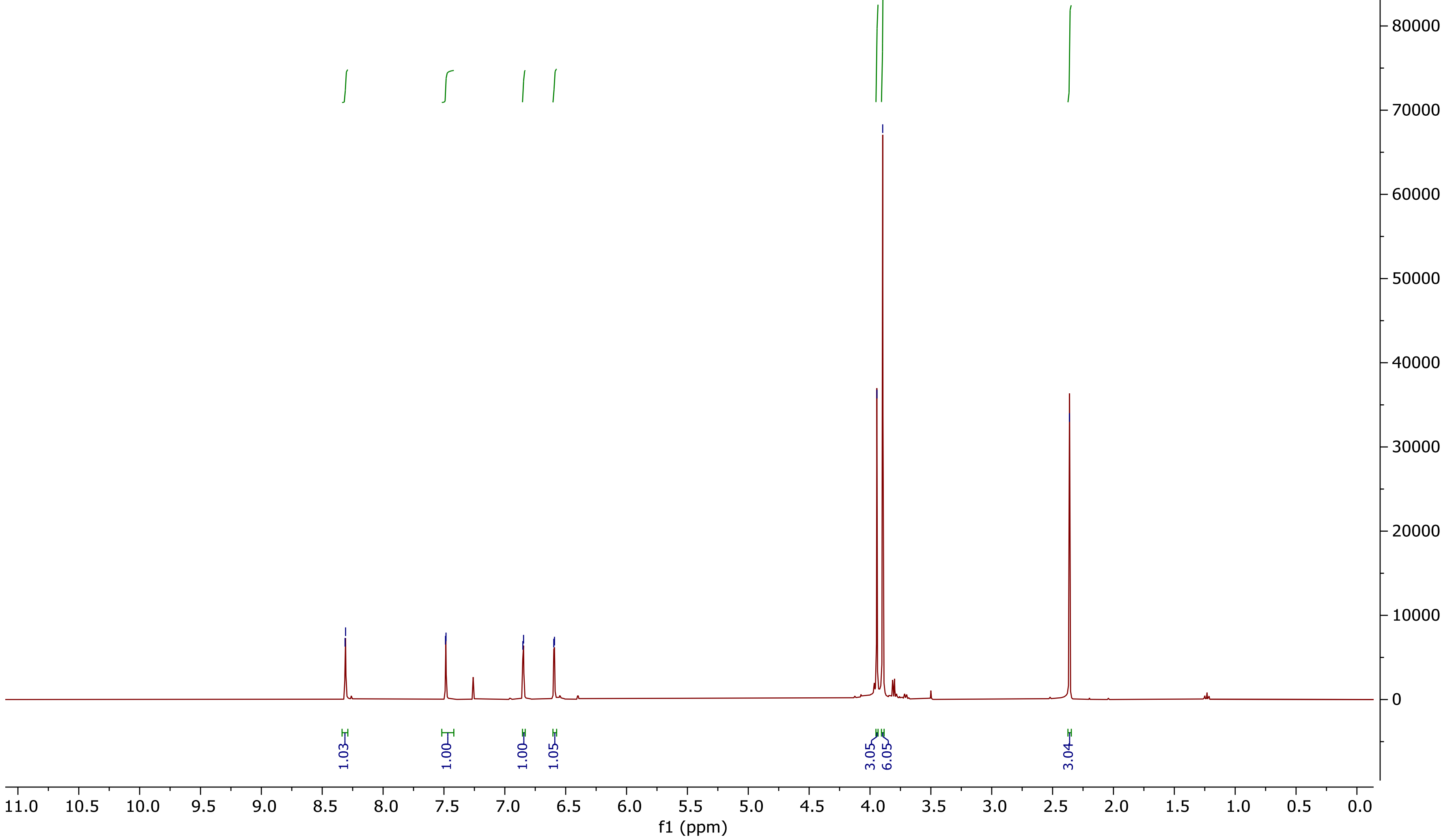

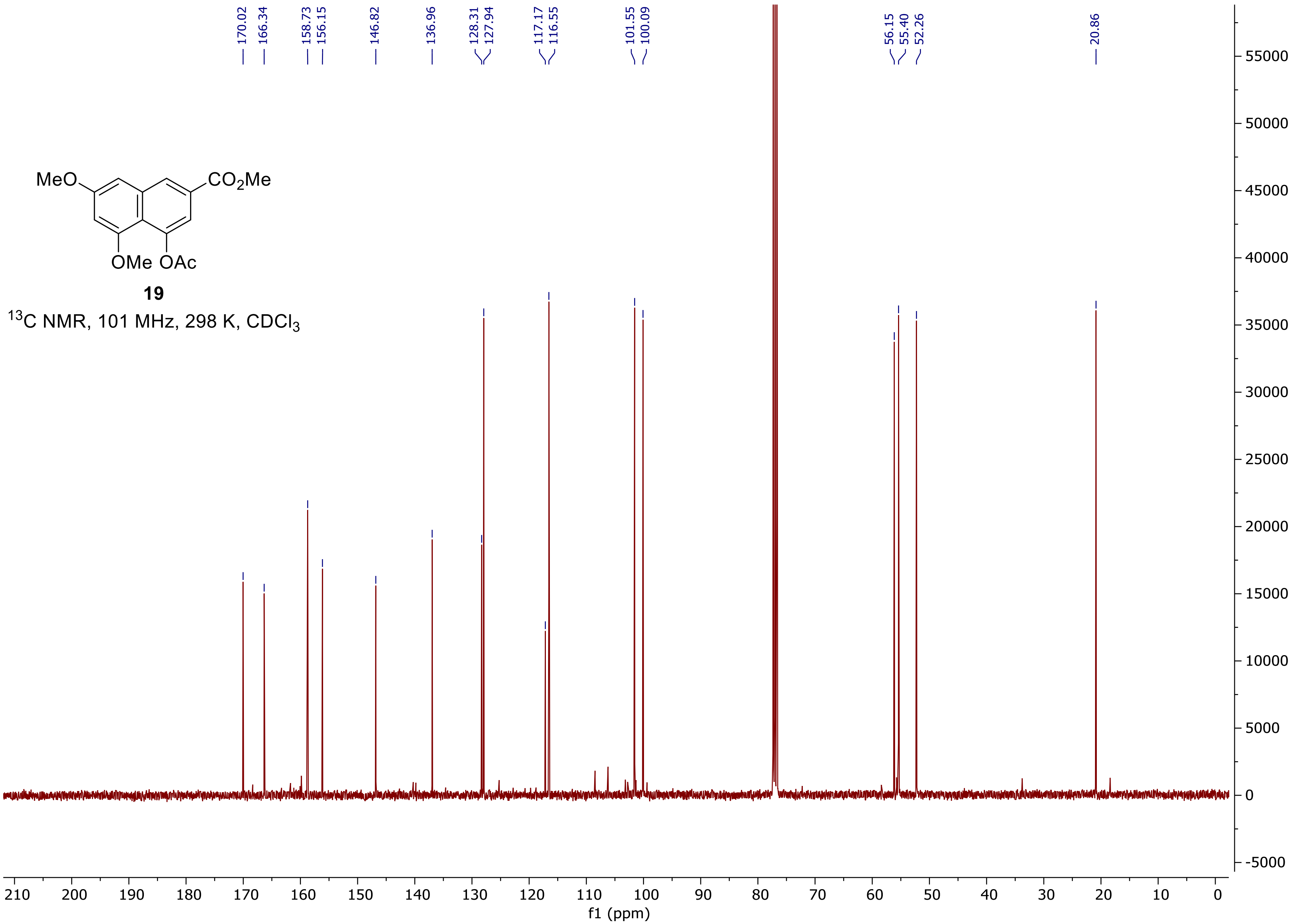

MeO
$CO_2Me$
OMe OAc
19
$^{13}C$ NMR, 101 MHz, 298 K, $CDCl_3$
170.02
166.34
158.73
156.15
146.82
136.96
128.31
127.94
117.17
116.55
101.55
100.09
56.15
55.40
52.26
20.86
55000
50000
45000
40000
35000
30000
25000
20000
15000
10000
5000
0
-5000
210
200
190
180
170
160
150
140
130
120
110
100
90
80
70
60
50
40
30
20
10
0
f1 (ppm)

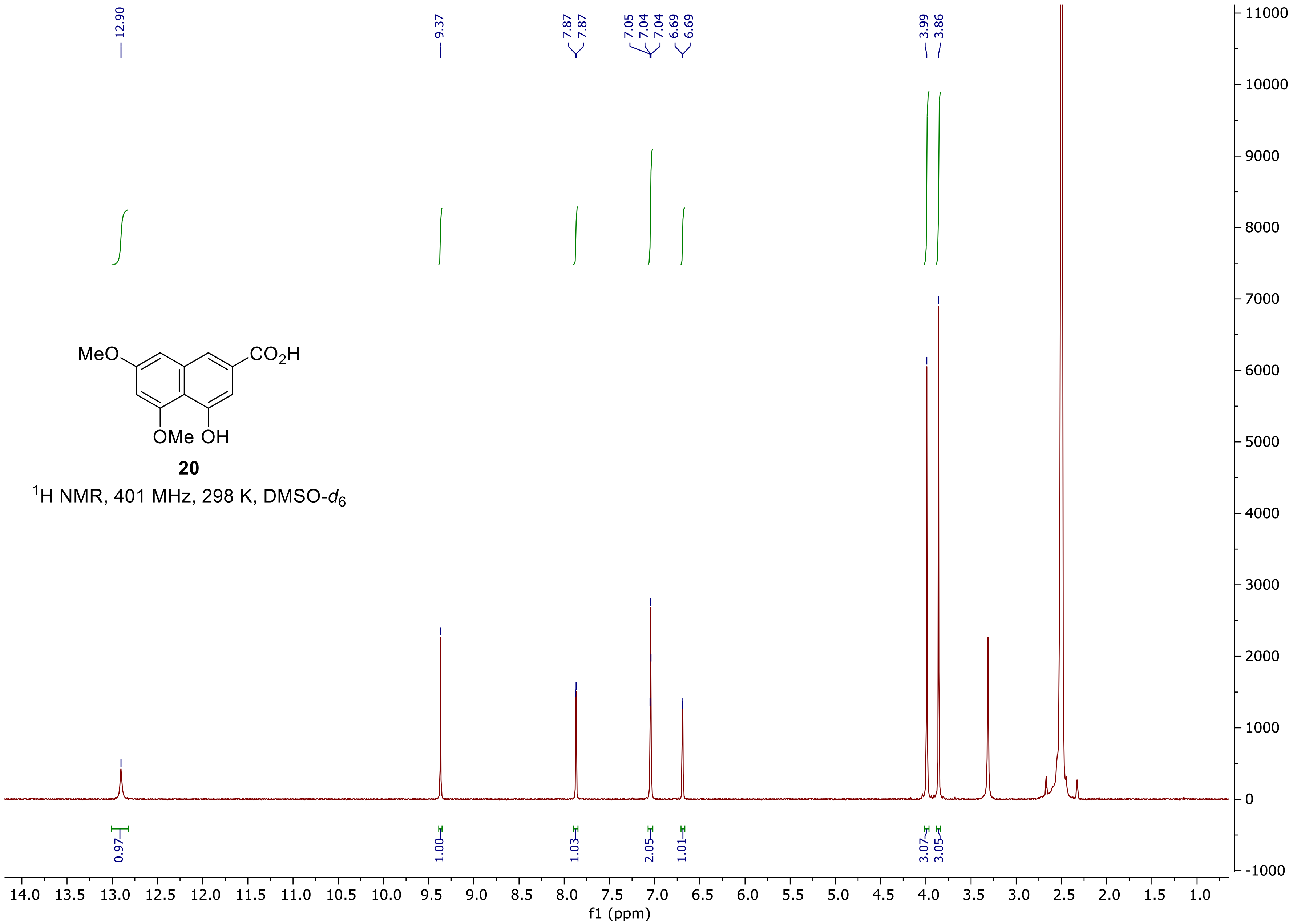

12.90
9.37
7.87
7.87
7.05
7.04
7.04
6.69
6.69
3.99
3.86
MeO
$CO_2H$
OMe OH
20
$^1$H NMR, 401 MHz, 298 K, DMSO-$d_6$
0.97
1.00
1.03
2.05
1.01
3.07
3.05
14.0 13.5 13.0 12.5 12.0 11.5 11.0 10.5 10.0 9.5 9.0 8.5 8.0 7.5 7.0 6.5 6.0 5.5 5.0 4.5 4.0 3.5 3.0 2.5 2.0 1.5 1.0
f1 (ppm)
11000 10000 9000 8000 7000 6000 5000 4000 3000 2000 1000 0 -1000

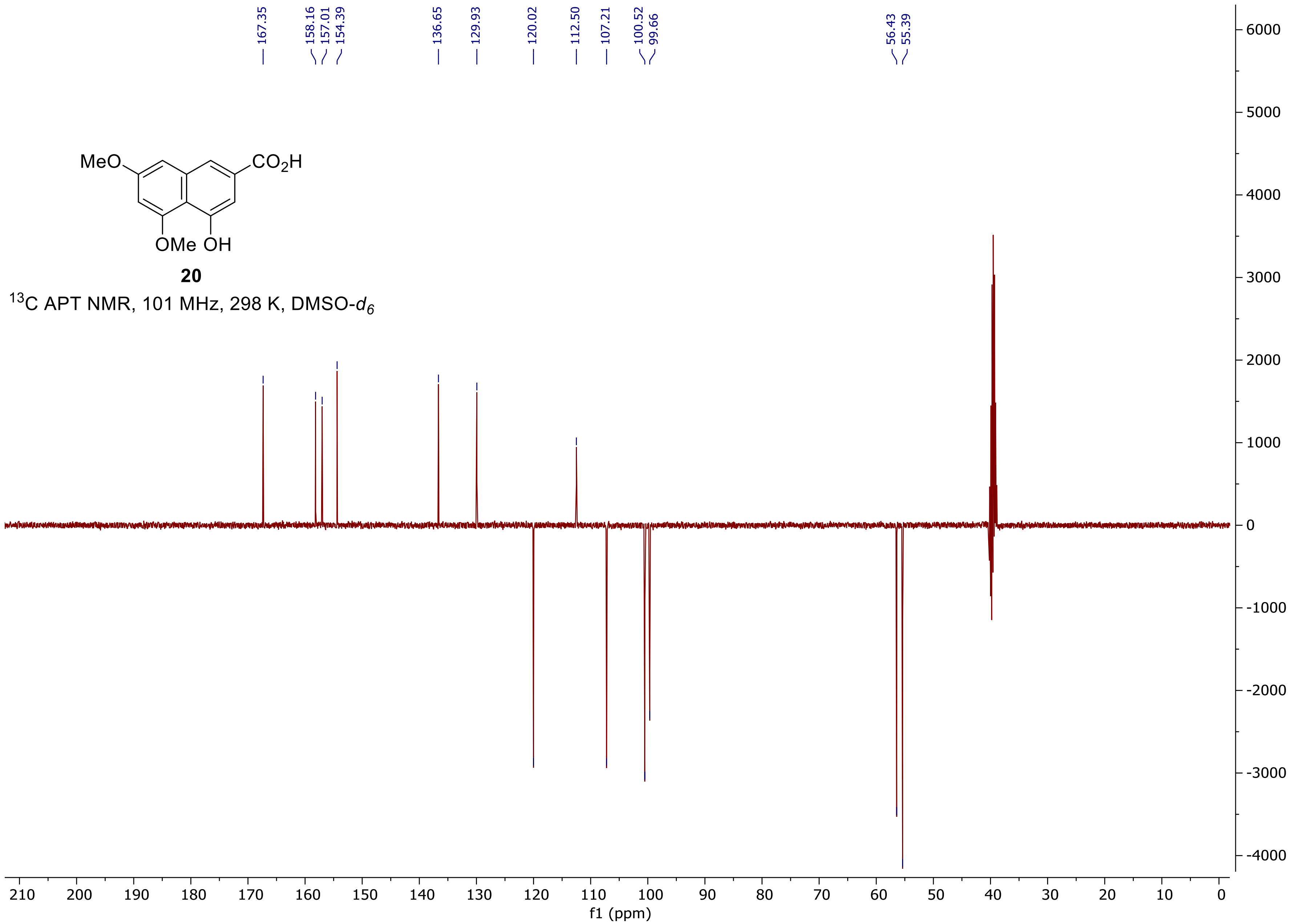

167.35
158.16
157.01
154.39
136.65
129.93
120.02
112.50
107.21
100.52
99.66
56.43
55.39
MeO
$CO_2H$
OMe OH
20
$^{13}C$ APT NMR, 101 MHz, 298 K, DMSO-$d_6$
f1 (ppm)

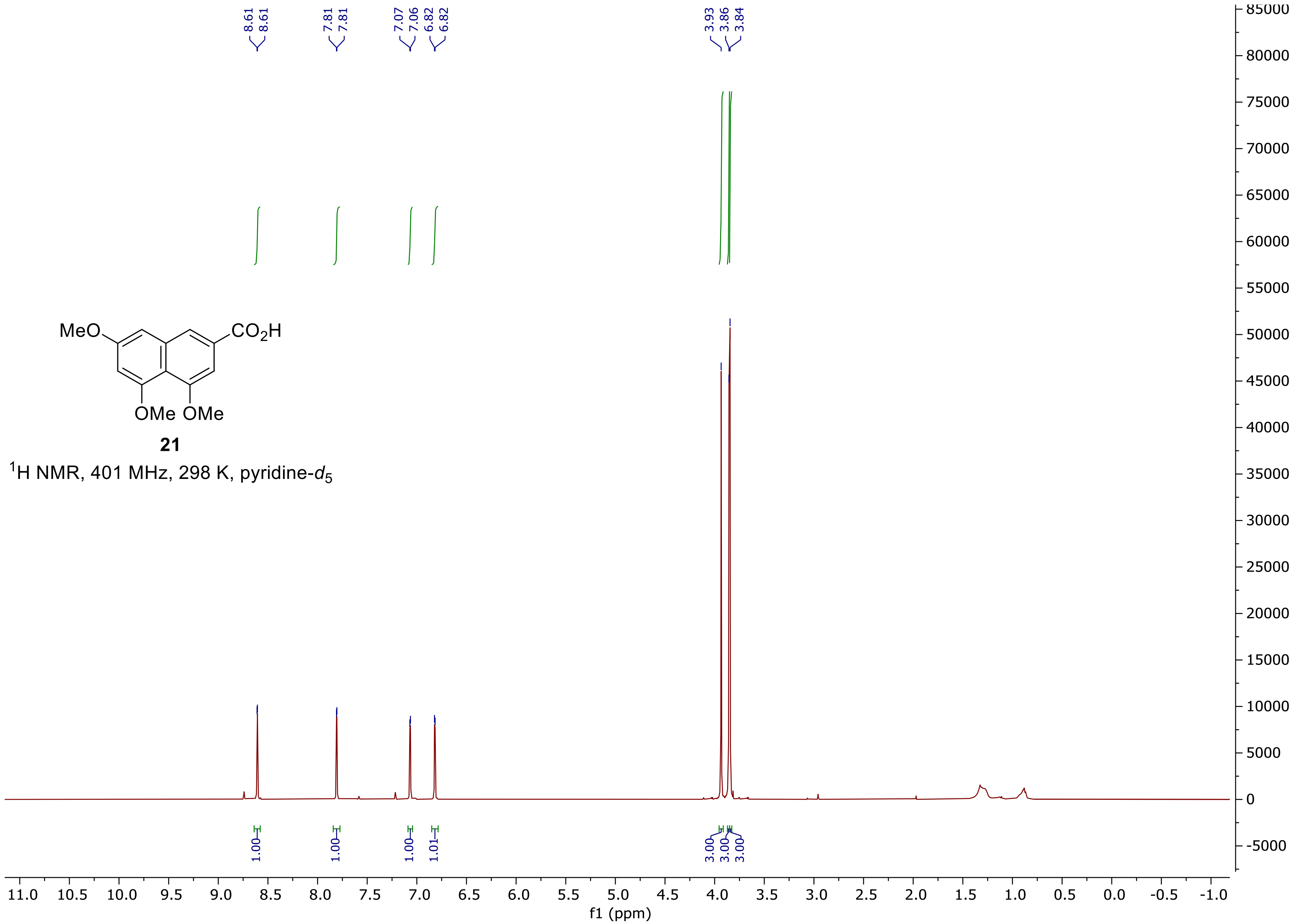


**21**

$^1$H NMR, 401 MHz, 298 K, pyridine-$d_5$

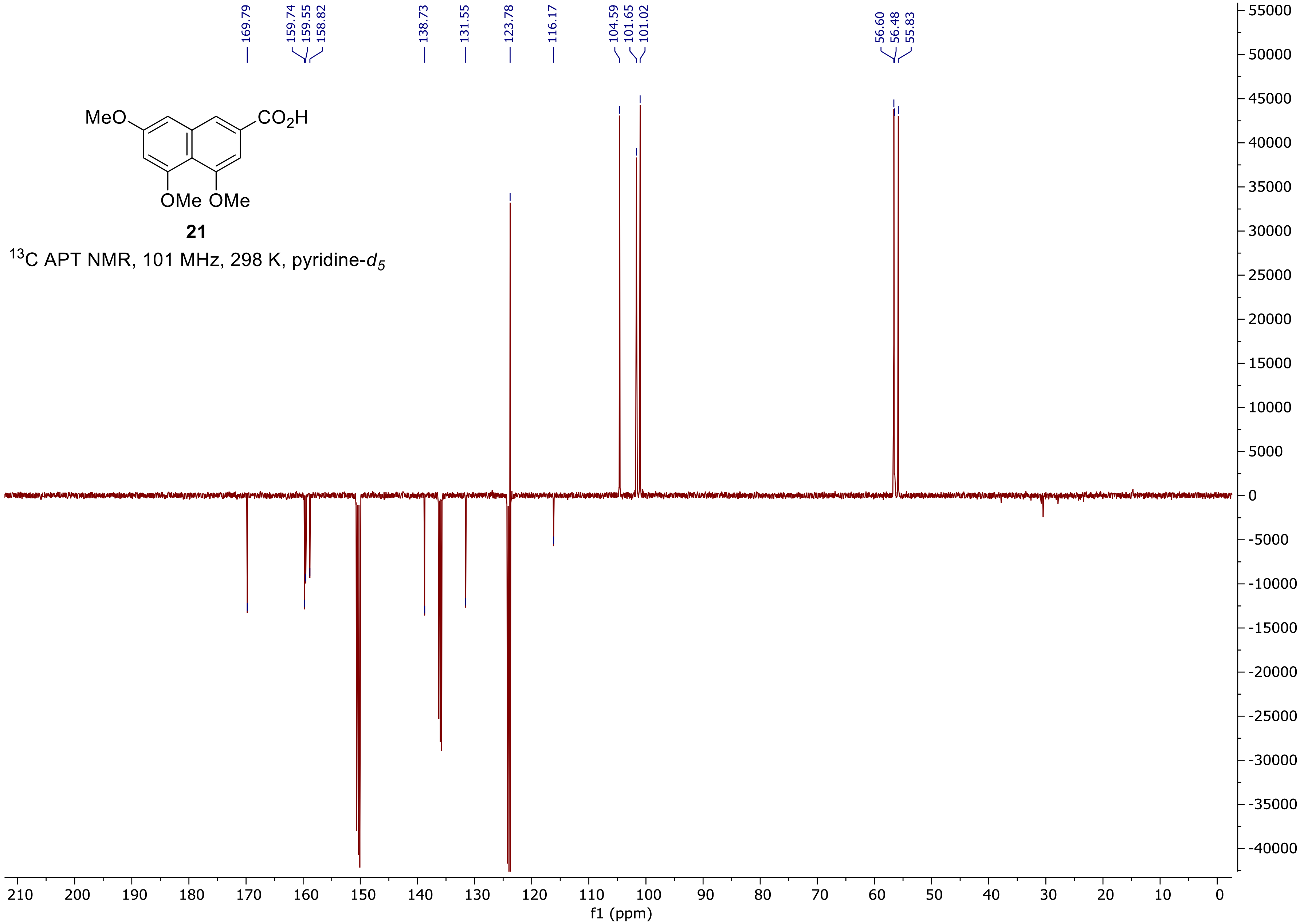
169.79
159.74
159.55
158.82
138.73
131.55
123.78
116.17
104.59
101.65
101.02
56.60
56.48
55.83
MeO
CO2H
OMe OMe
21
13C APT NMR, 101 MHz, 298 K, pyridine-d5
55000
50000
45000
40000
35000
30000
25000
20000
15000
10000
5000
0
-5000
-10000
-15000
-20000
-25000
-30000
-35000
-40000
210 200 190 180 170 160 150 140 130 120 110 100 90 80 70 60 50 40 30 20 10 0
f1 (ppm)

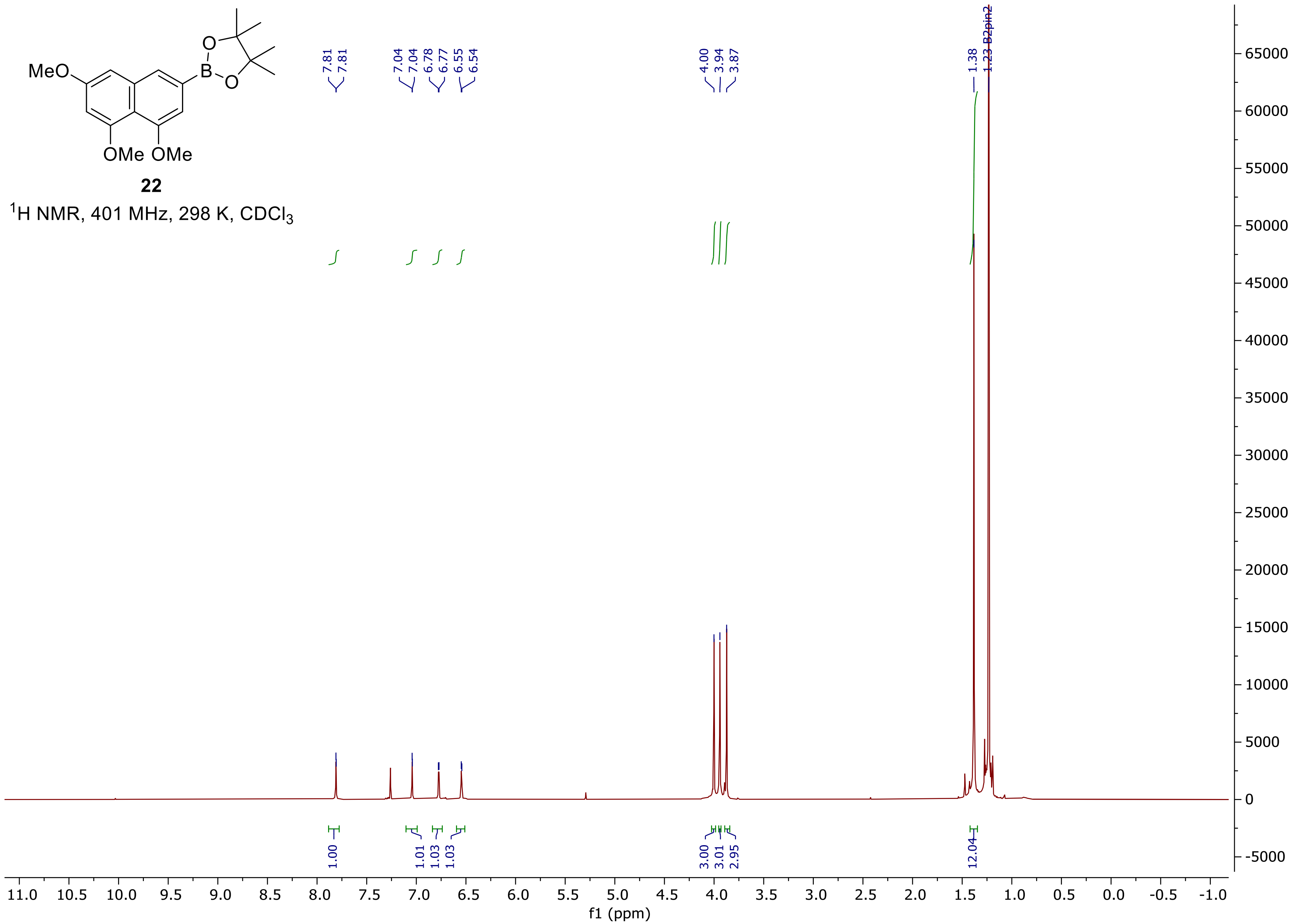
MeO
OMe OMe
B
O
O
22
$^1$H NMR, 401 MHz, 298 K, $CDCl_3$
7.81
7.81
7.04
7.04
6.78
6.77
6.55
6.54
4.00
3.94
3.87
1.38
1.23 B2pin2
1.00
1.01
1.03
1.03
3.00
3.01
2.95
12.04
f1 (ppm)

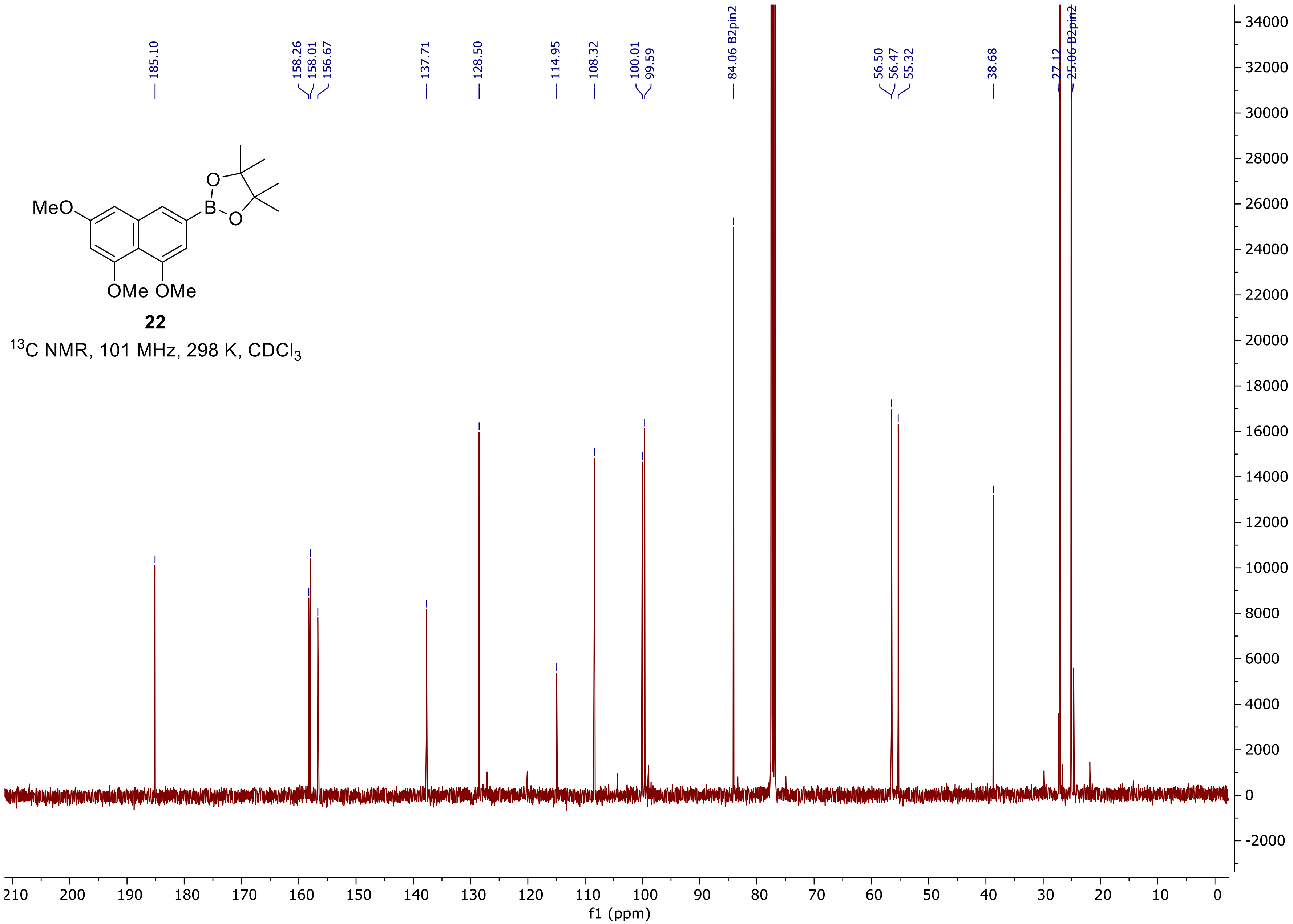
185.10
158.26
158.01
156.67
137.71
128.50
114.95
108.32
100.01
99.59
84.06 B2pin2
56.50
56.47
55.32
38.68
27.12
25.06 B2pin2
MeO
O
B
O
OMe OMe
22
$^{13}$C NMR, 101 MHz, 298 K, $CDCl_3$
f1 (ppm)

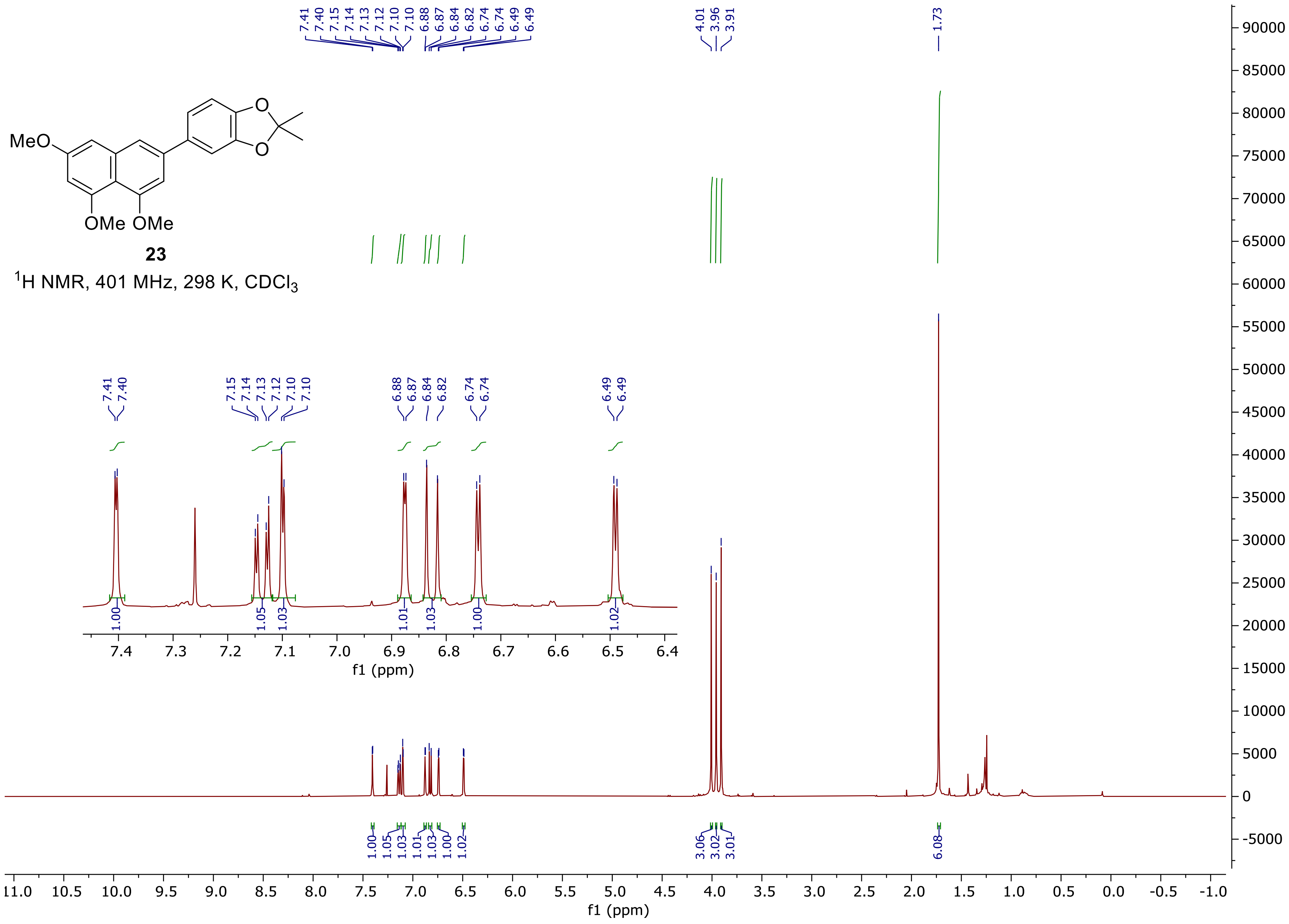
MeO
OMe OMe
23
$^1$H NMR, 401 MHz, 298 K, $CDCl_3$
f1 (ppm)

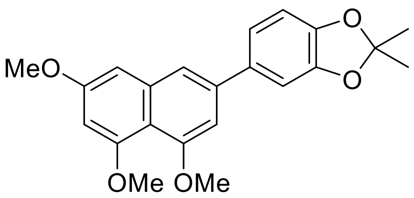


**23**

$^{13}C$ NMR, 101 MHz, 298 K, $CDCl_3$

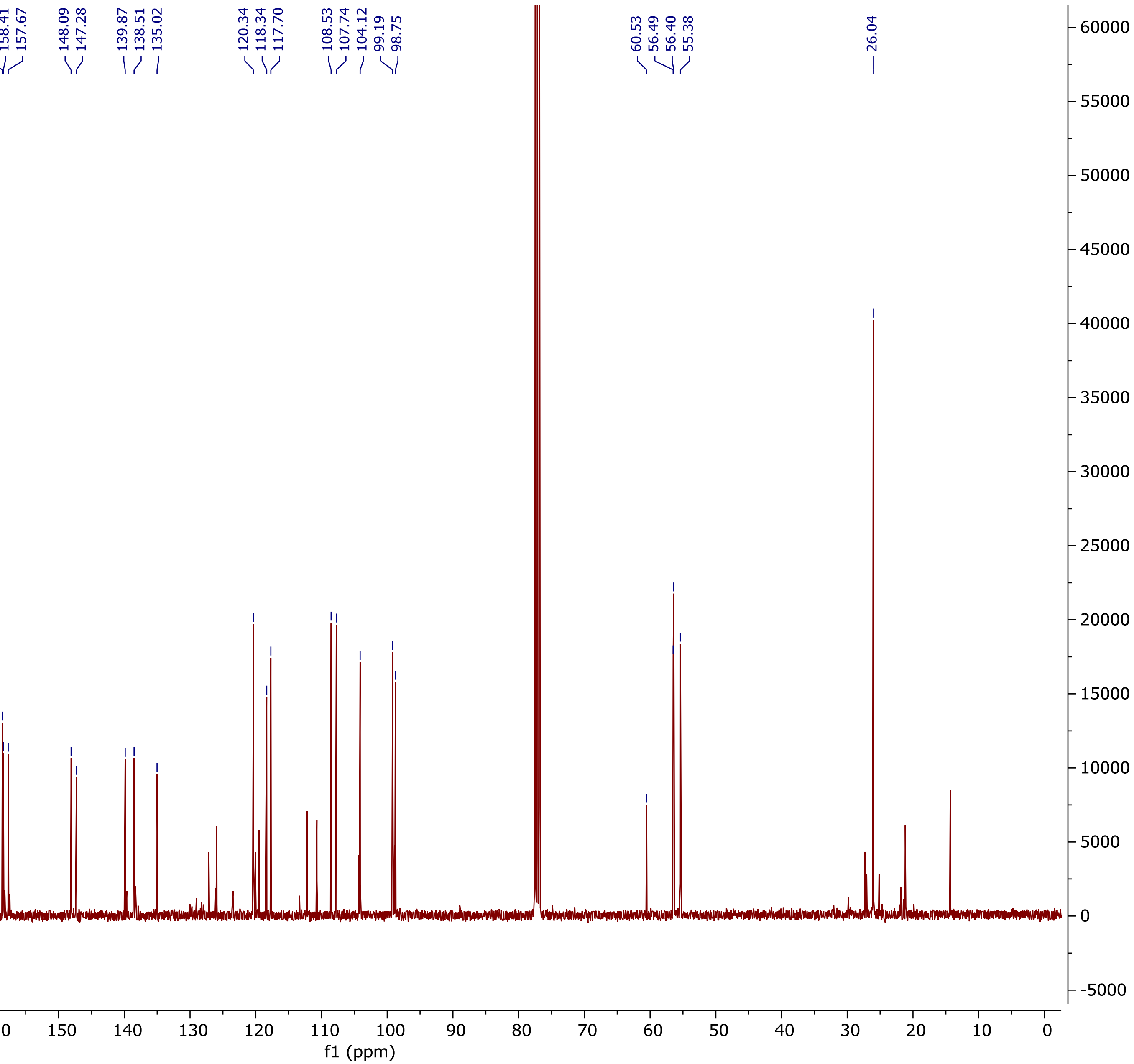

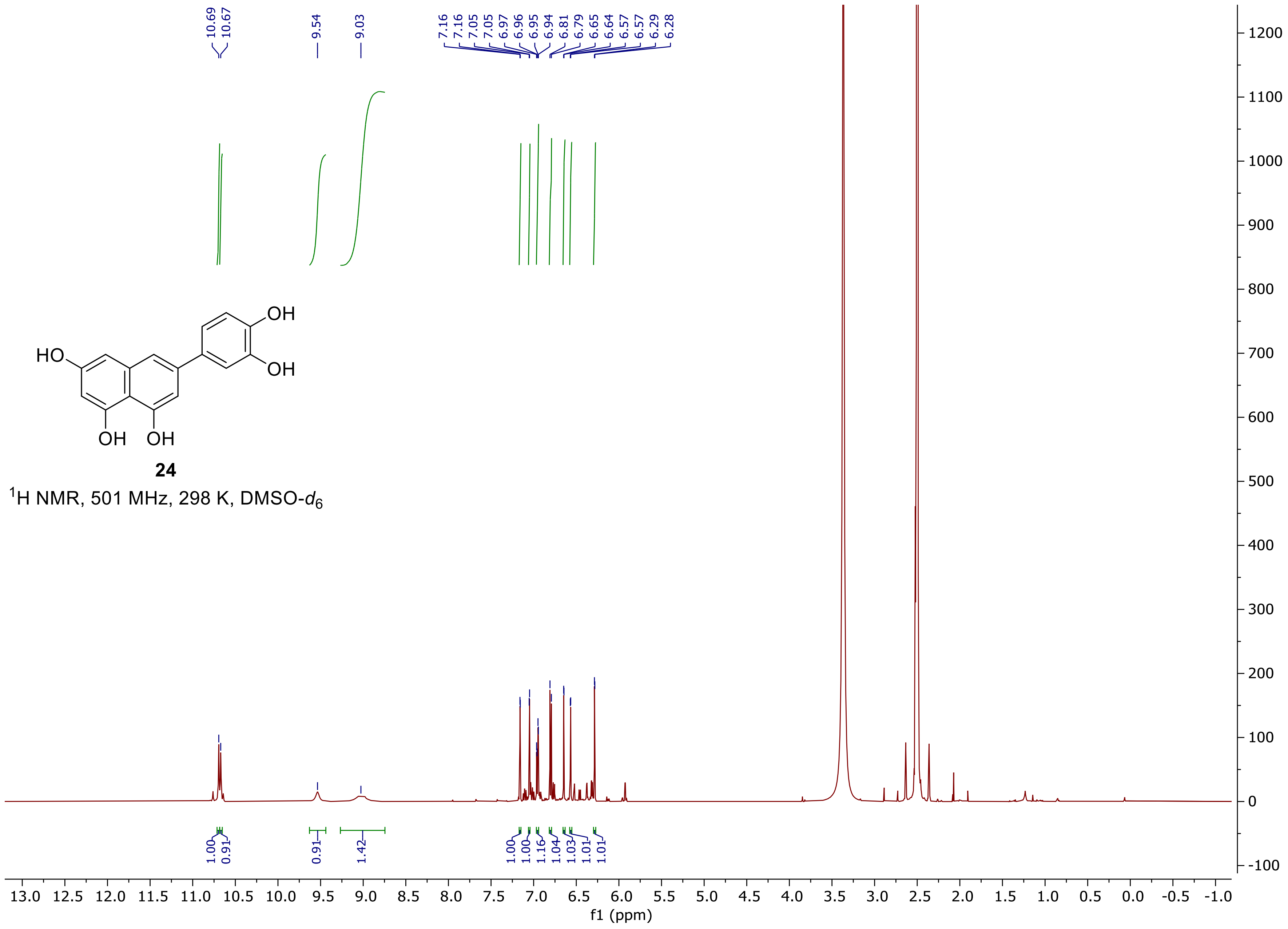

**24**

$^{1}$H NMR, 501 MHz, 298 K, DMSO-$d_6$

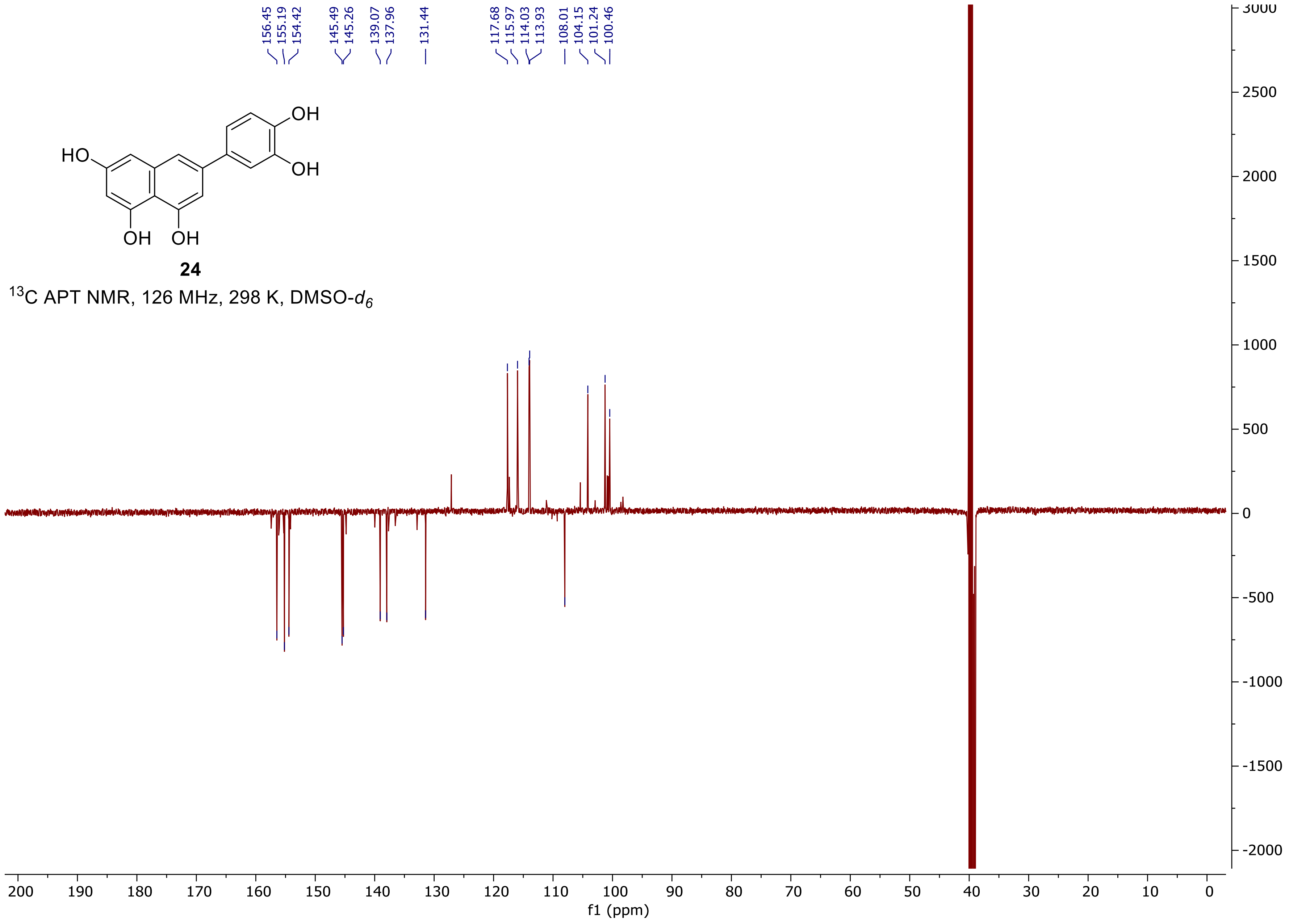
156.45
155.19
154.42
145.49
145.26
139.07
137.96
131.44
117.68
115.97
114.03
113.93
108.01
104.15
101.24
100.46
HO
OH
OH
OH
OH
24
$^{13}$C APT NMR, 126 MHz, 298 K, DMSO-$d_6$
3000
2500
2000
1500
1000
500
0
-500
-1000
-1500
-2000
200 190 180 170 160 150 140 130 120 110 100 90 80 70 60 50 40 30 20 10 0
f1 (ppm)

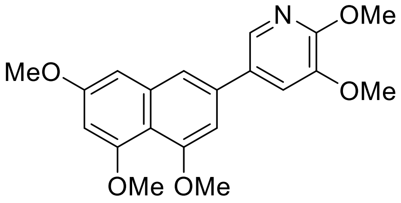


**25**

$^{1}$H NMR, 401 MHz, 298 K, $CDCl_3$

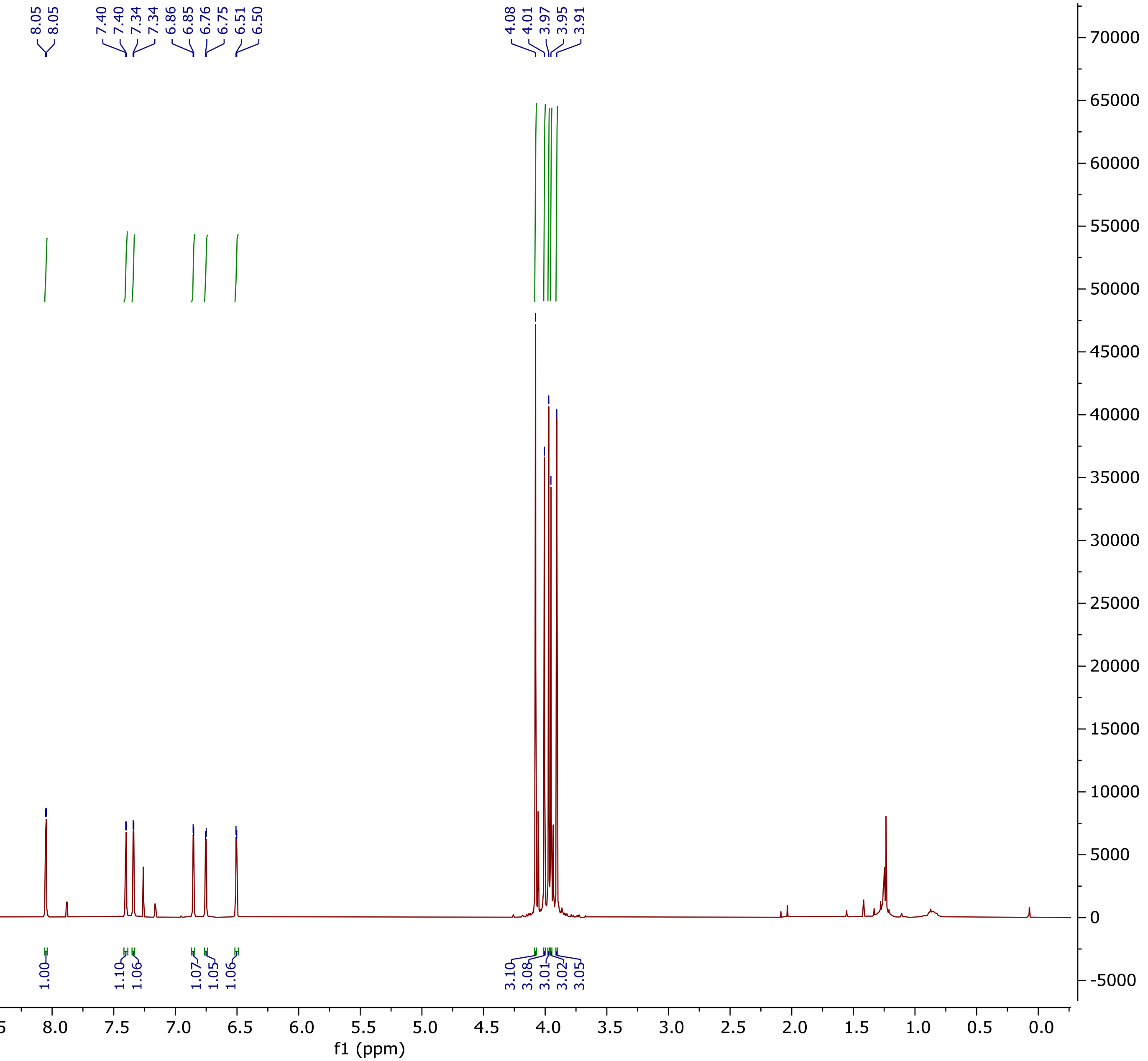

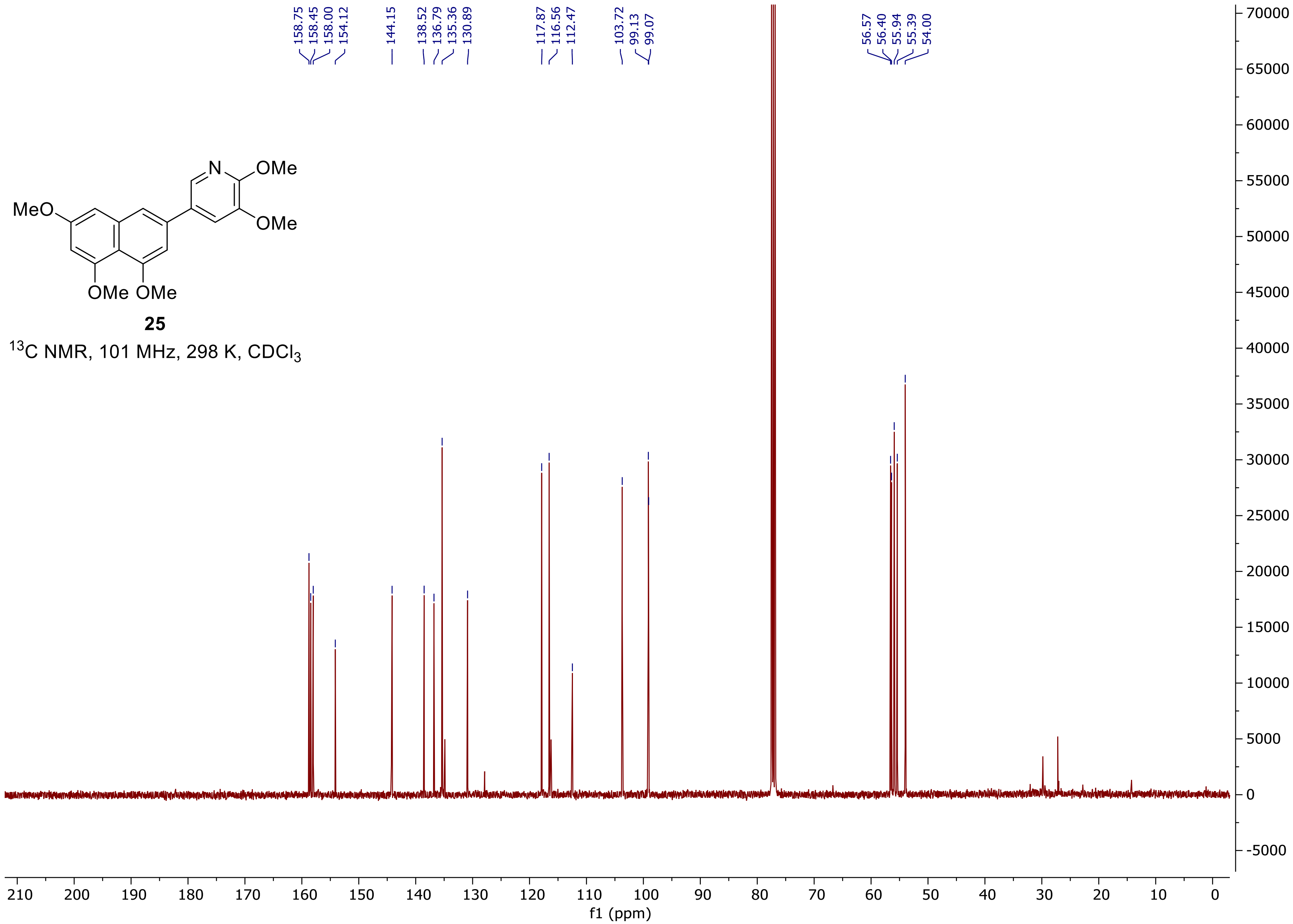


**25**

$^{13}C$ NMR, 101 MHz, 298 K, $CDCl_3$

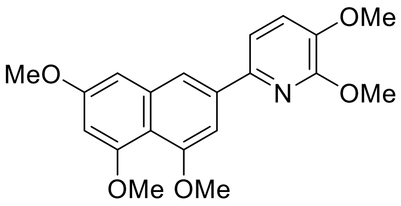

**26**

$^{1}H$ NMR, 401 MHz, 298 K, $CDCl_3$

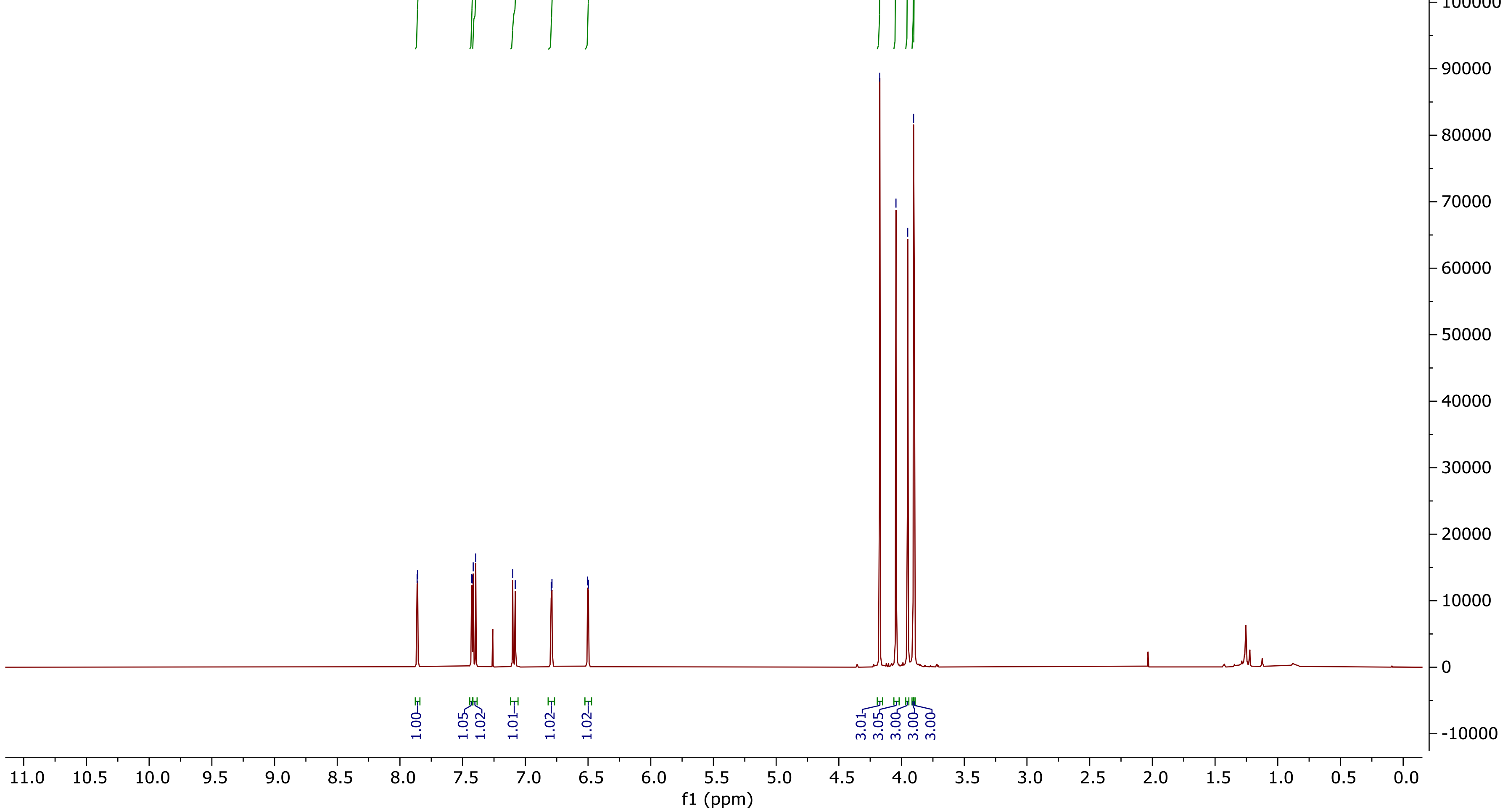

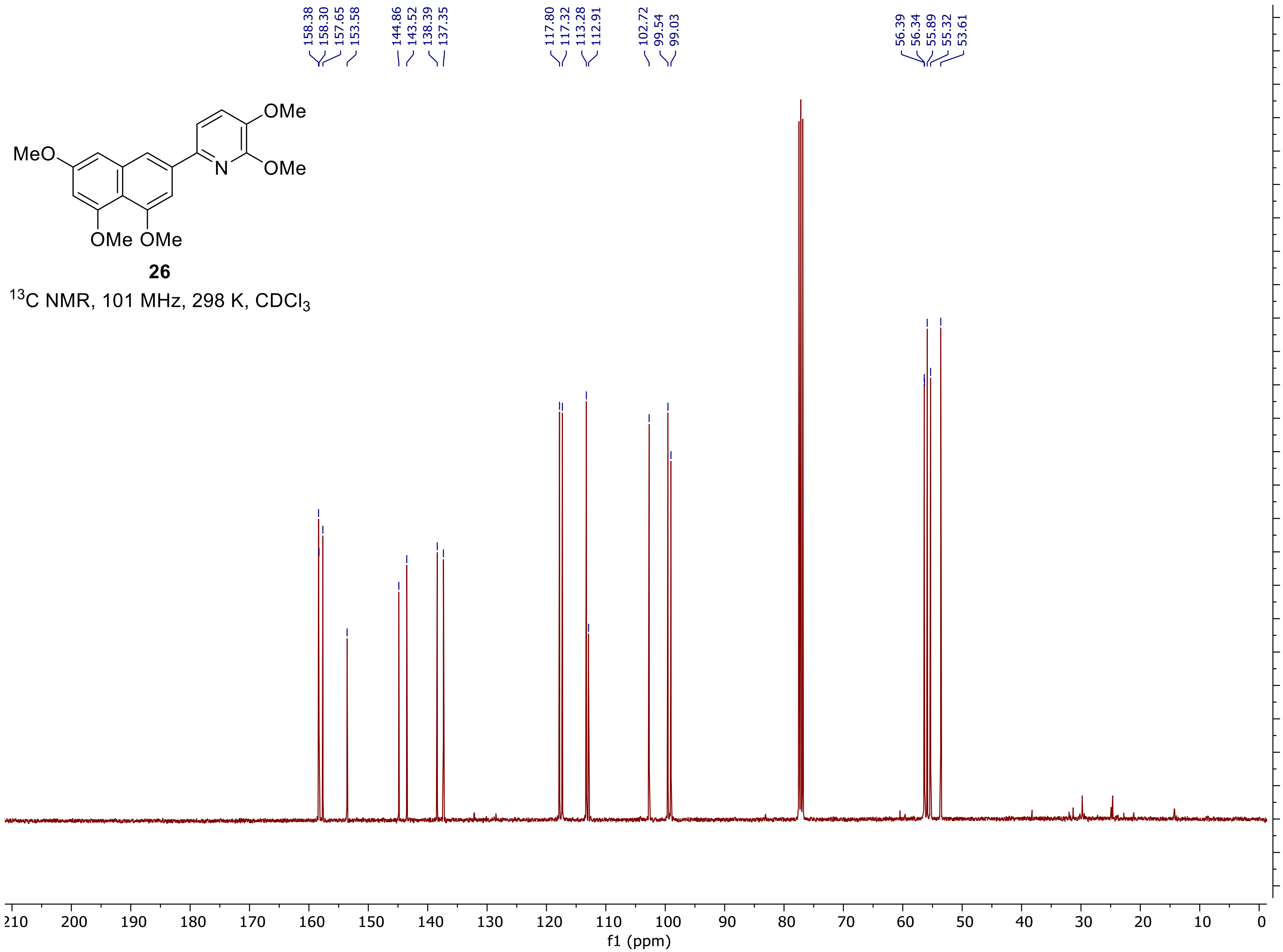


**26**

$^{13}C$ NMR, 101 MHz, 298 K, $CDCl_3$

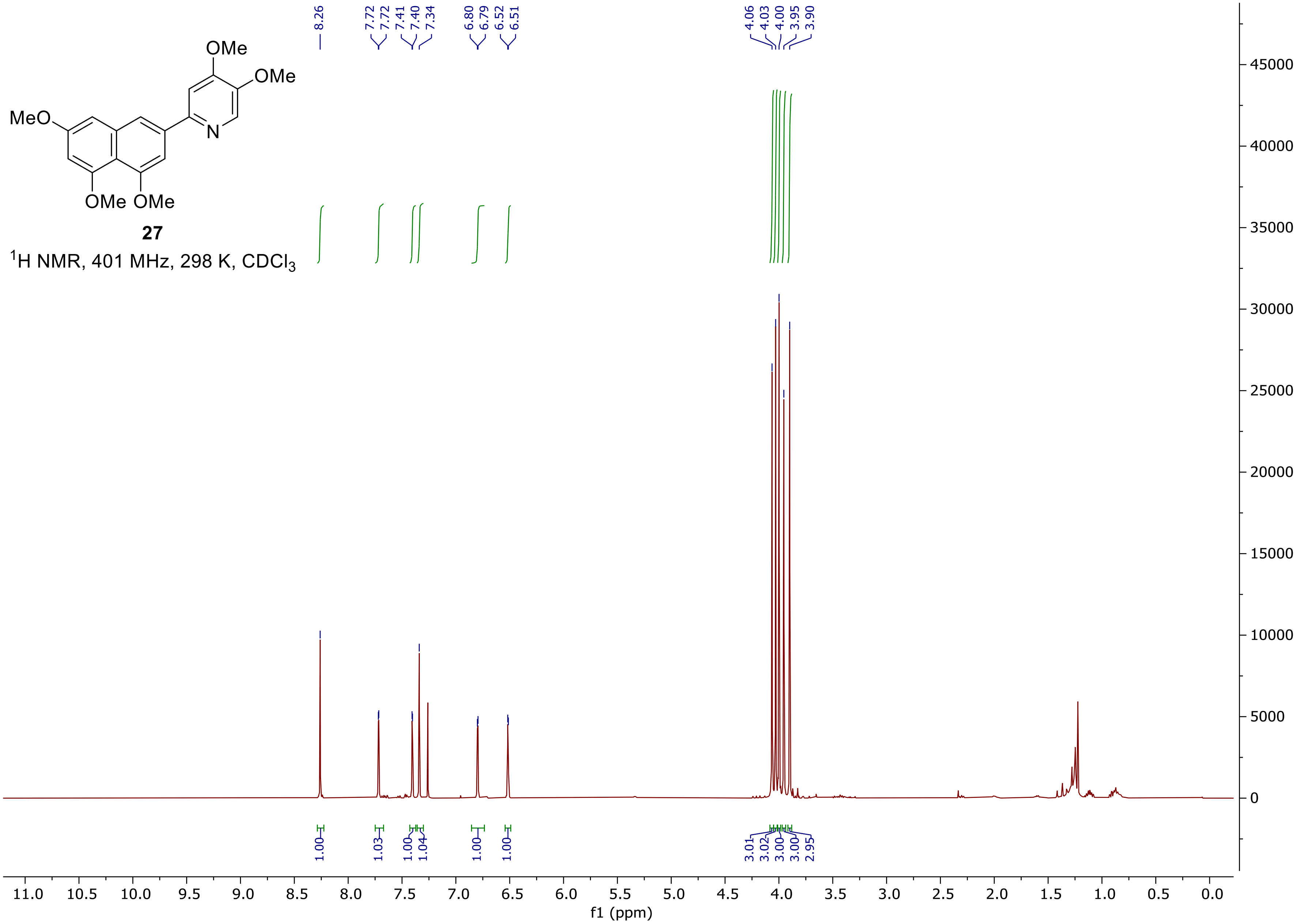
OMe
OMe
MeO
N
OMe OMe
27
$^1$H NMR, 401 MHz, 298 K, CDCl$_3$
8.26
7.72
7.72
7.41
7.40
7.34
6.80
6.79
6.52
6.51
4.06
4.03
4.00
3.95
3.90
1.00
1.03
1.00
1.04
1.00
1.00
3.01
3.02
3.00
3.00
2.95
f1 (ppm)

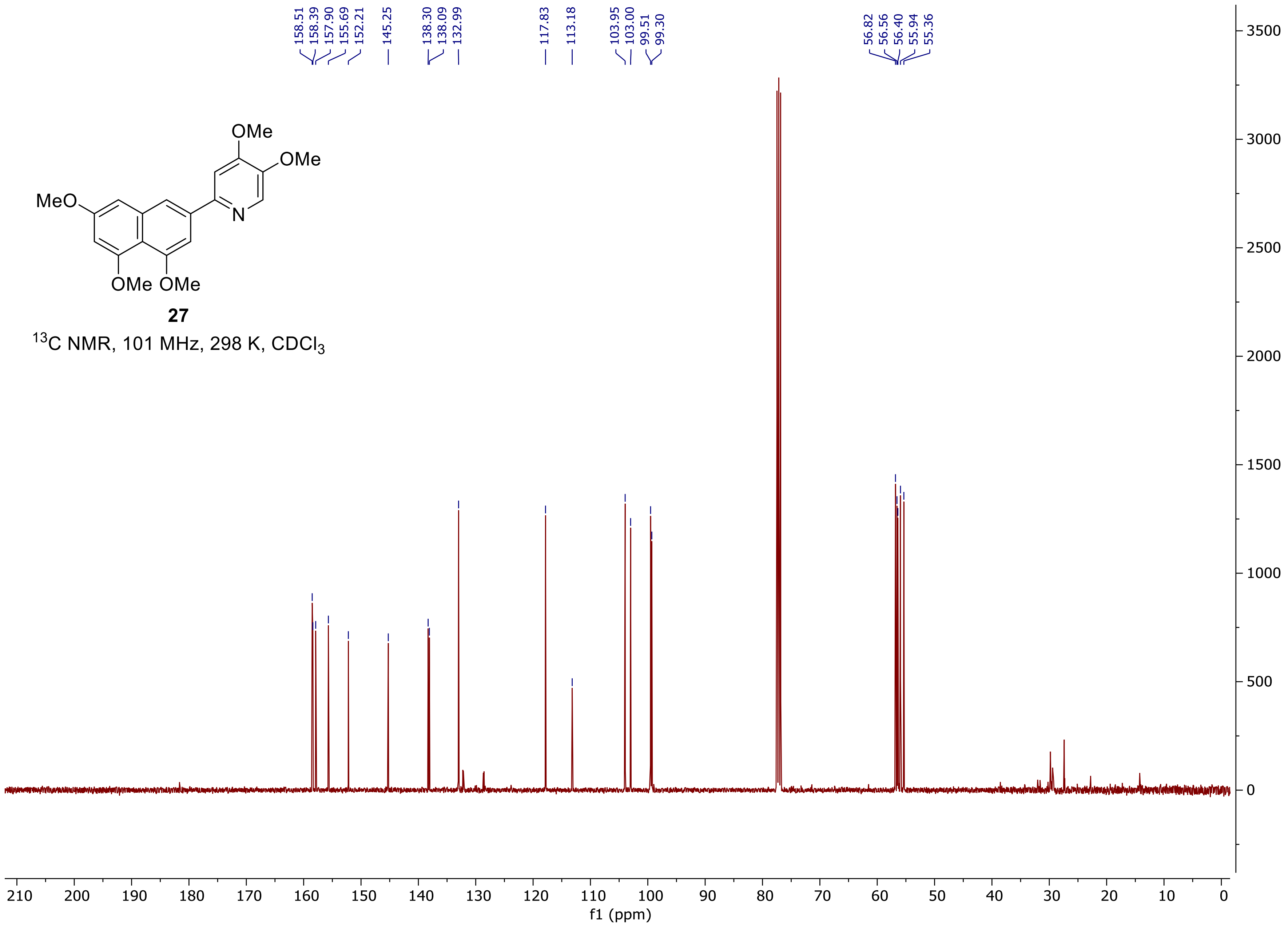


**27**

$^{13}C$ NMR, 101 MHz, 298 K, $CDCl_3$

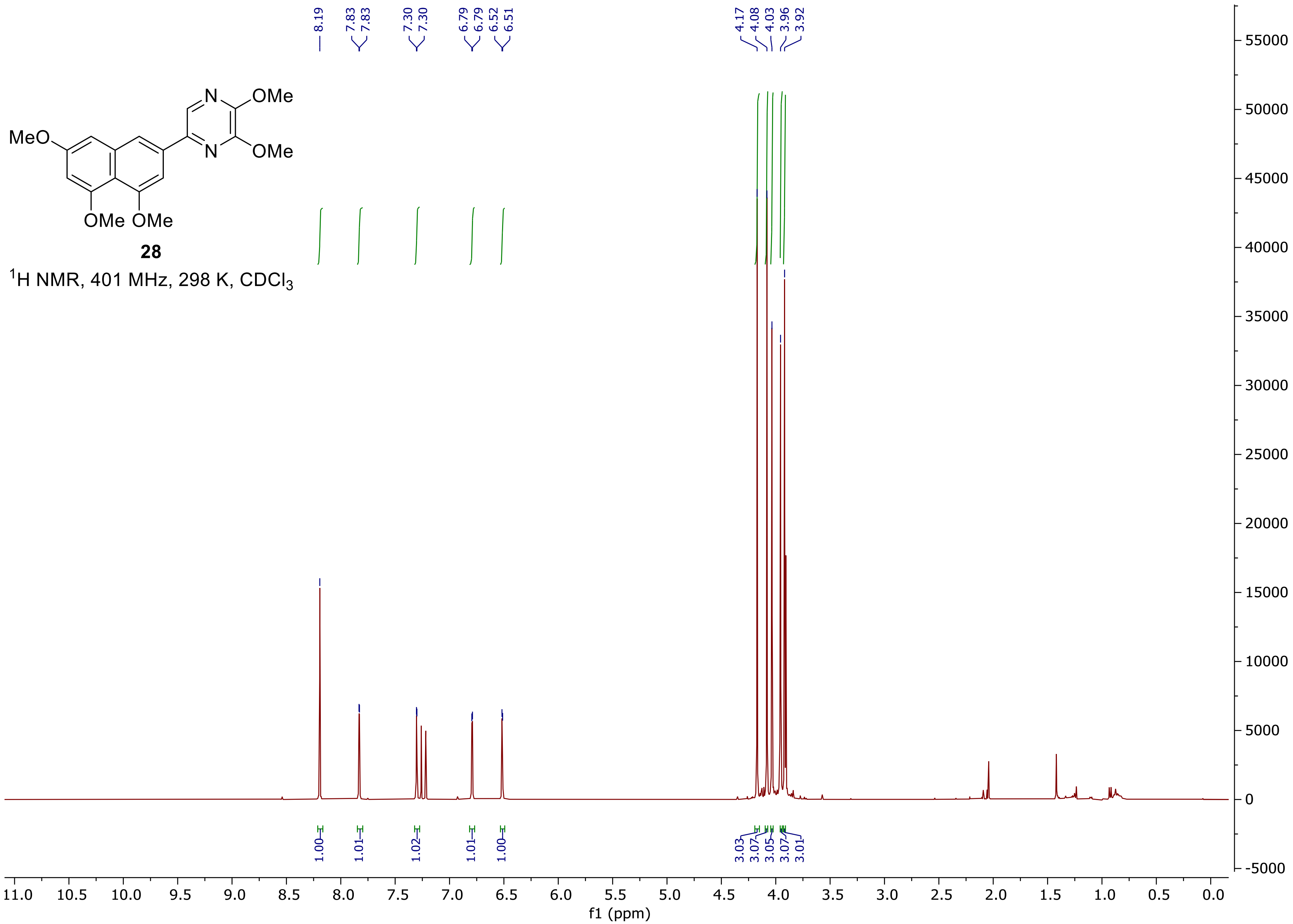

MeO
N
OMe
N
OMe
OMe OMe
28
¹H NMR, 401 MHz, 298 K, CDCl₃
8.19
7.83
7.83
7.30
7.30
6.79
6.79
6.52
6.51
4.17
4.08
4.03
3.96
3.92
1.00
1.01
1.02
1.01
1.00
3.03
3.07
3.05
3.07
3.01
f1 (ppm)

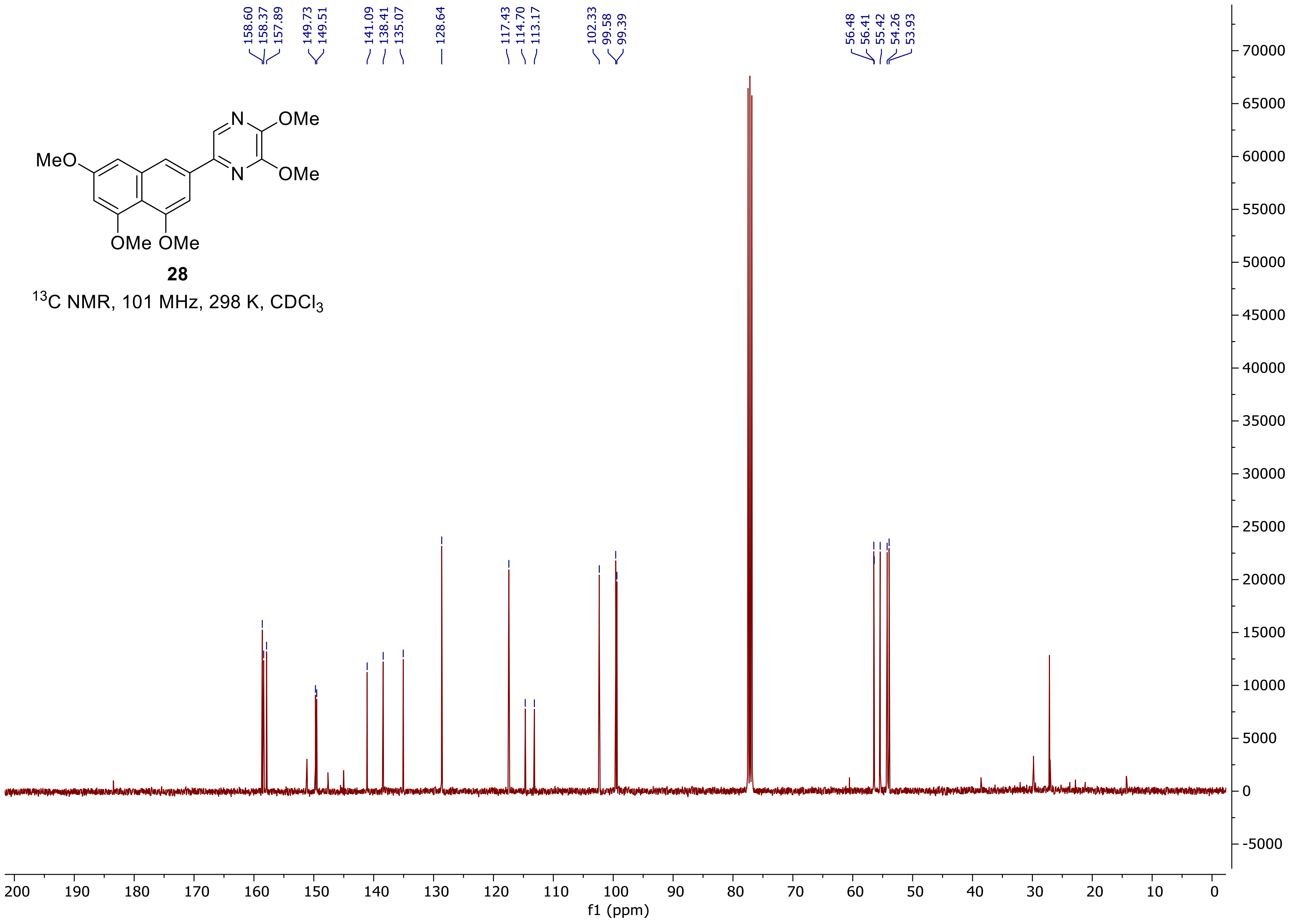


**28**

$^{13}$C NMR, 101 MHz, 298 K, $CDCl_3$

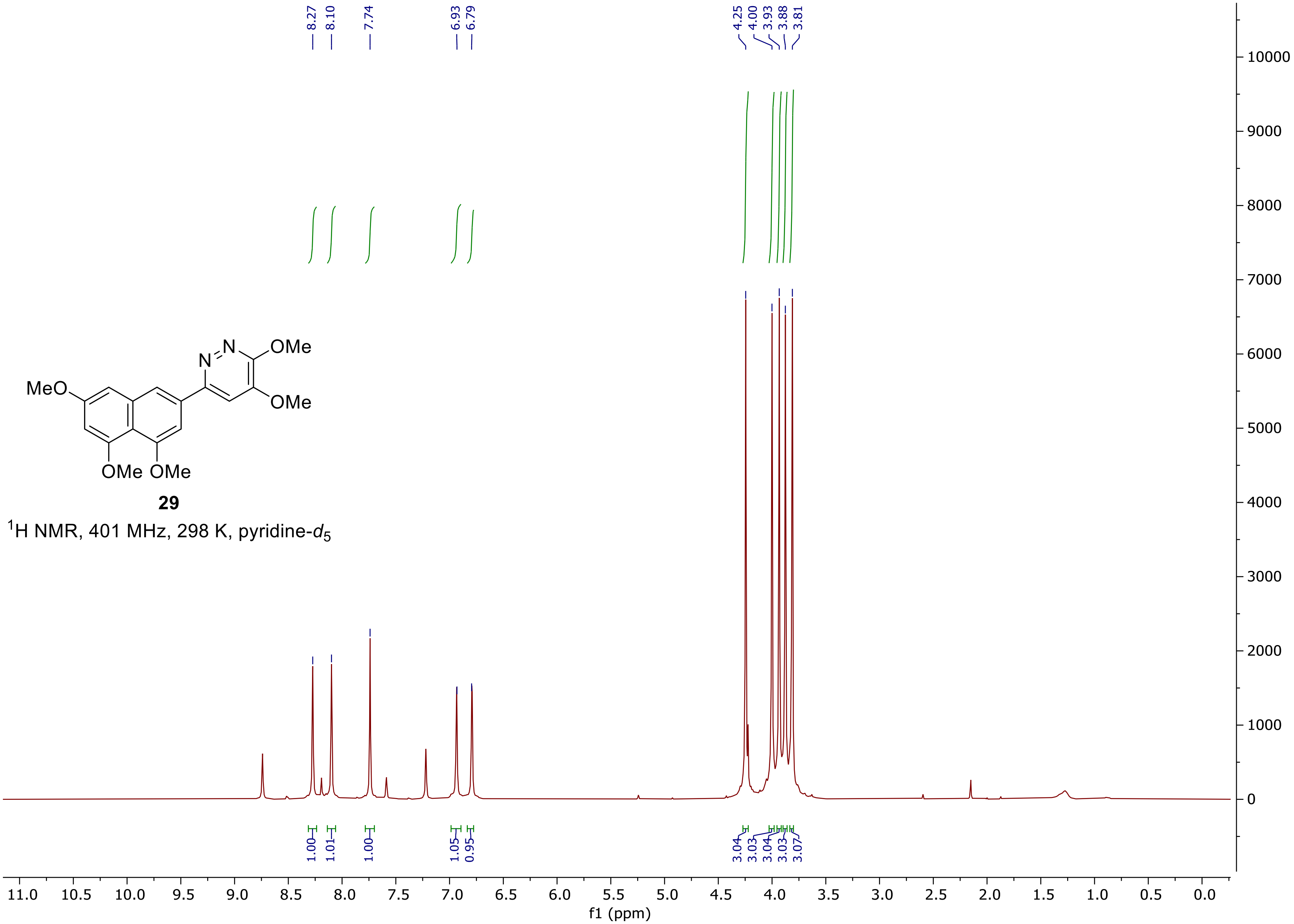


**29**

$^{1}H$ NMR, 401 MHz, 298 K, pyridine-$d_5$

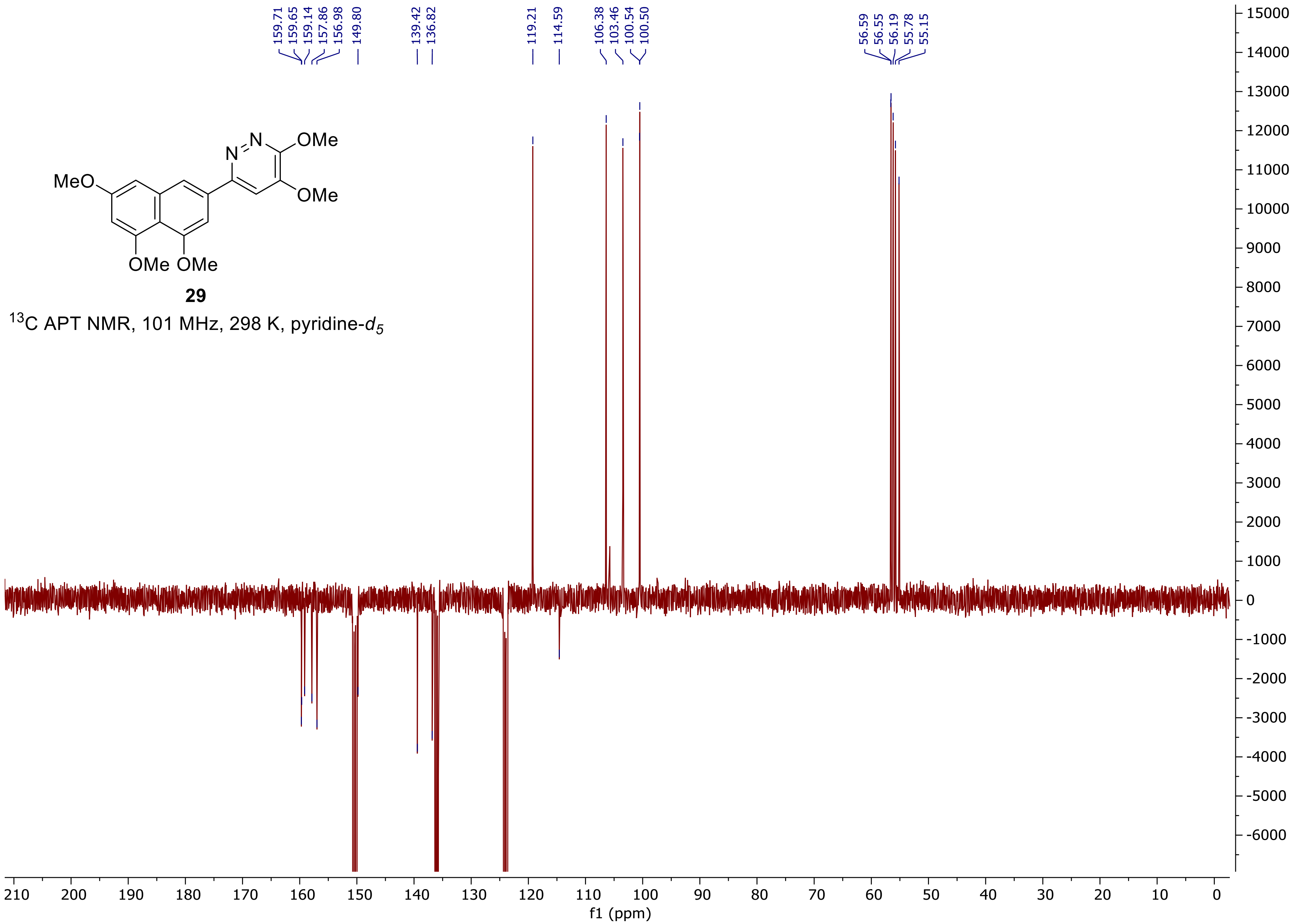


**29**

$^{13}C$ APT NMR, 101 MHz, 298 K, pyridine-$d_5$

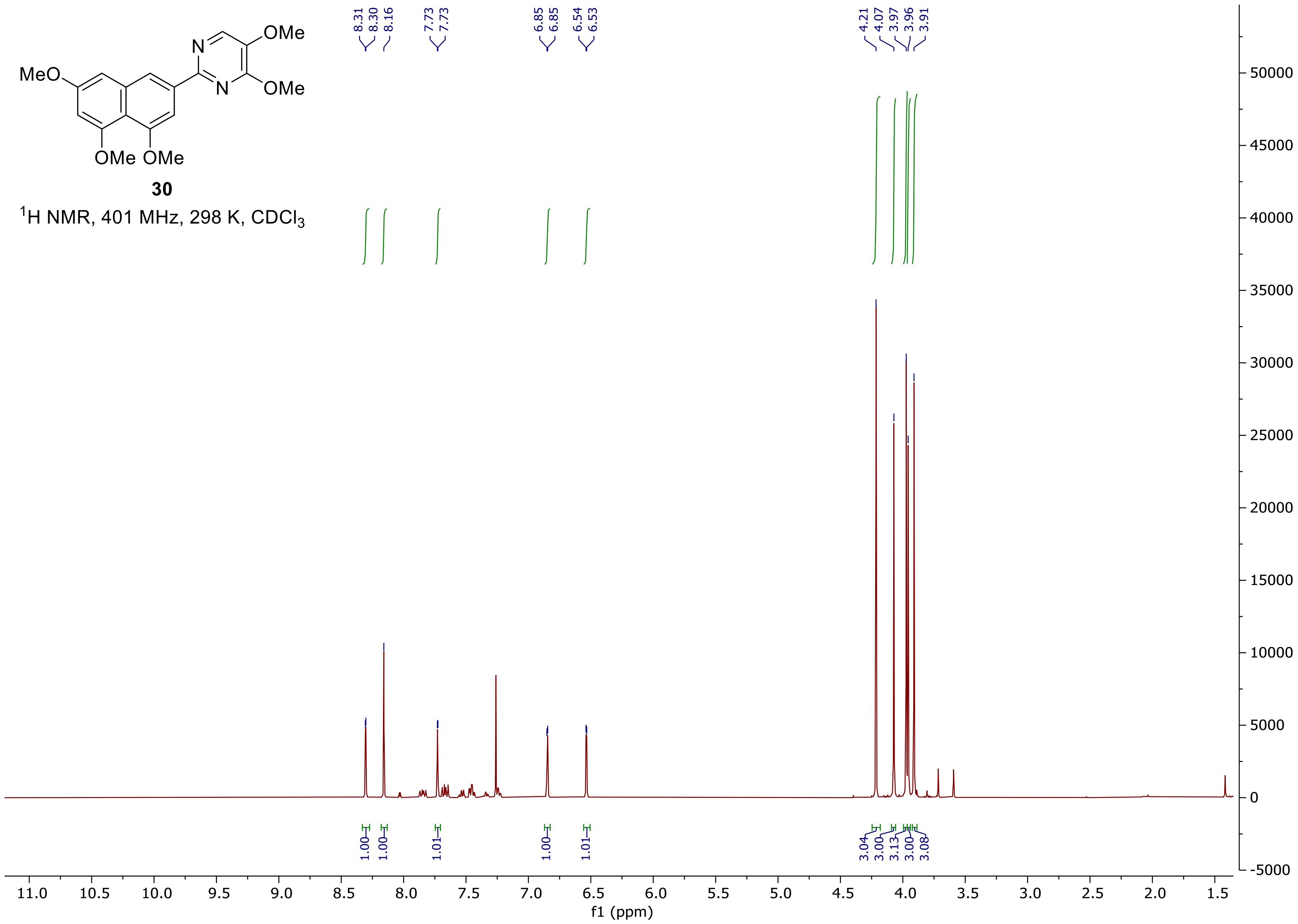

OMe
MeO
N
N
OMe
OMe OMe
30
$^1$H NMR, 401 MHz, 298 K, CDCl$_3$
8.31
8.30
8.16
7.73
7.73
6.85
6.85
6.54
6.53
4.21
4.07
3.97
3.96
3.91
1.00
1.00
1.01
1.00
1.01
3.04
3.00
3.13
3.00
3.08
11.0 10.5 10.0 9.5 9.0 8.5 8.0 7.5 7.0 6.5 6.0 5.5 5.0 4.5 4.0 3.5 3.0 2.5 2.0 1.5
f1 (ppm)
50000
45000
40000
35000
30000
25000
20000
15000
10000
5000
0
-5000

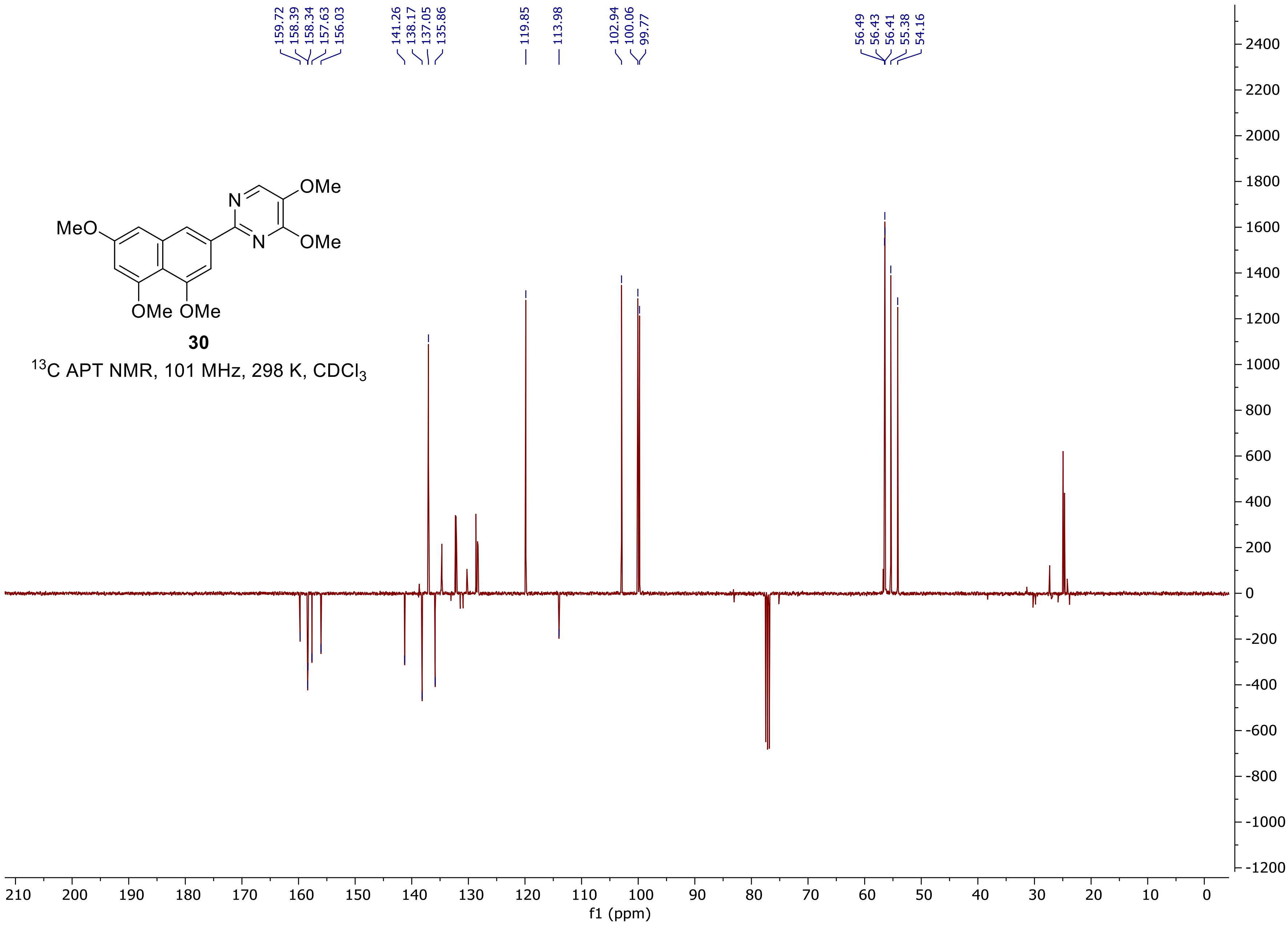


**30**

$^{13}C$ APT NMR, 101 MHz, 298 K, $CDCl_3$

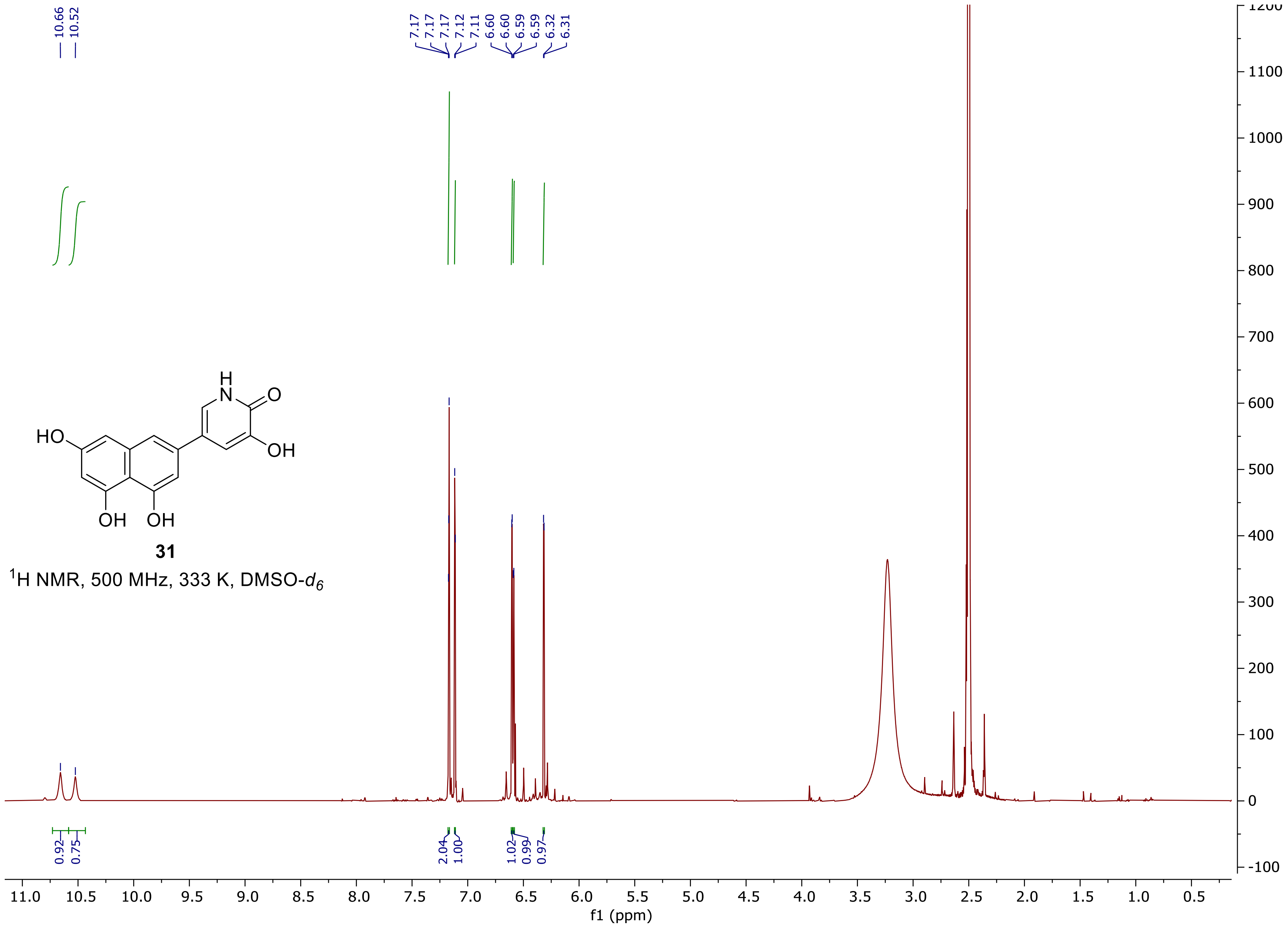


**31**

$^{1}$H NMR, 500 MHz, 333 K, DMSO-$d_6$

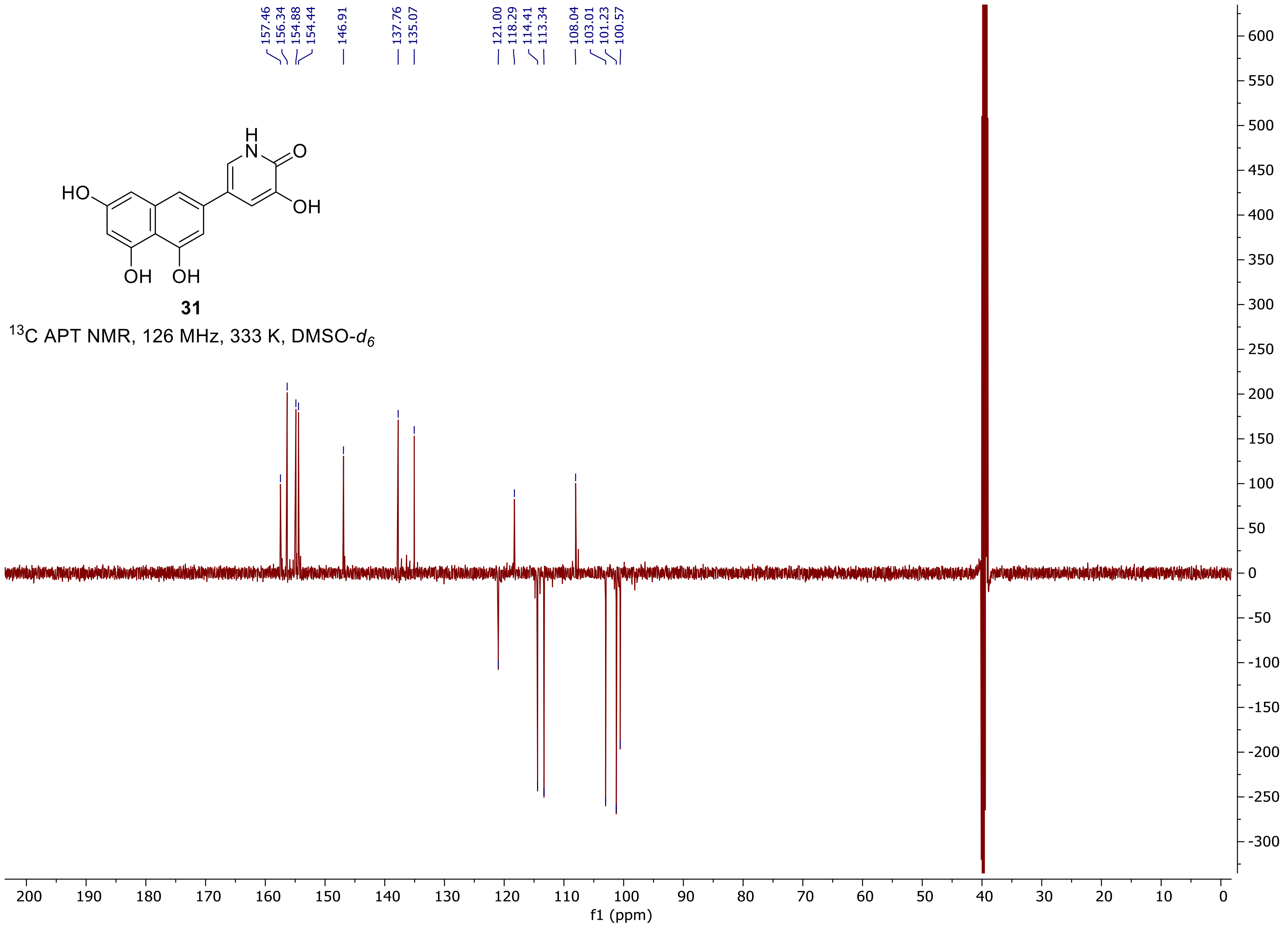
157.46
156.34
154.88
154.44
146.91
137.76
135.07
121.00
118.29
114.41
113.34
108.04
103.01
101.23
100.57
HO
H
N
O
OH
OH
OH
31
$^{13}$C APT NMR, 126 MHz, 333 K, DMSO-$d_6$
f1 (ppm)

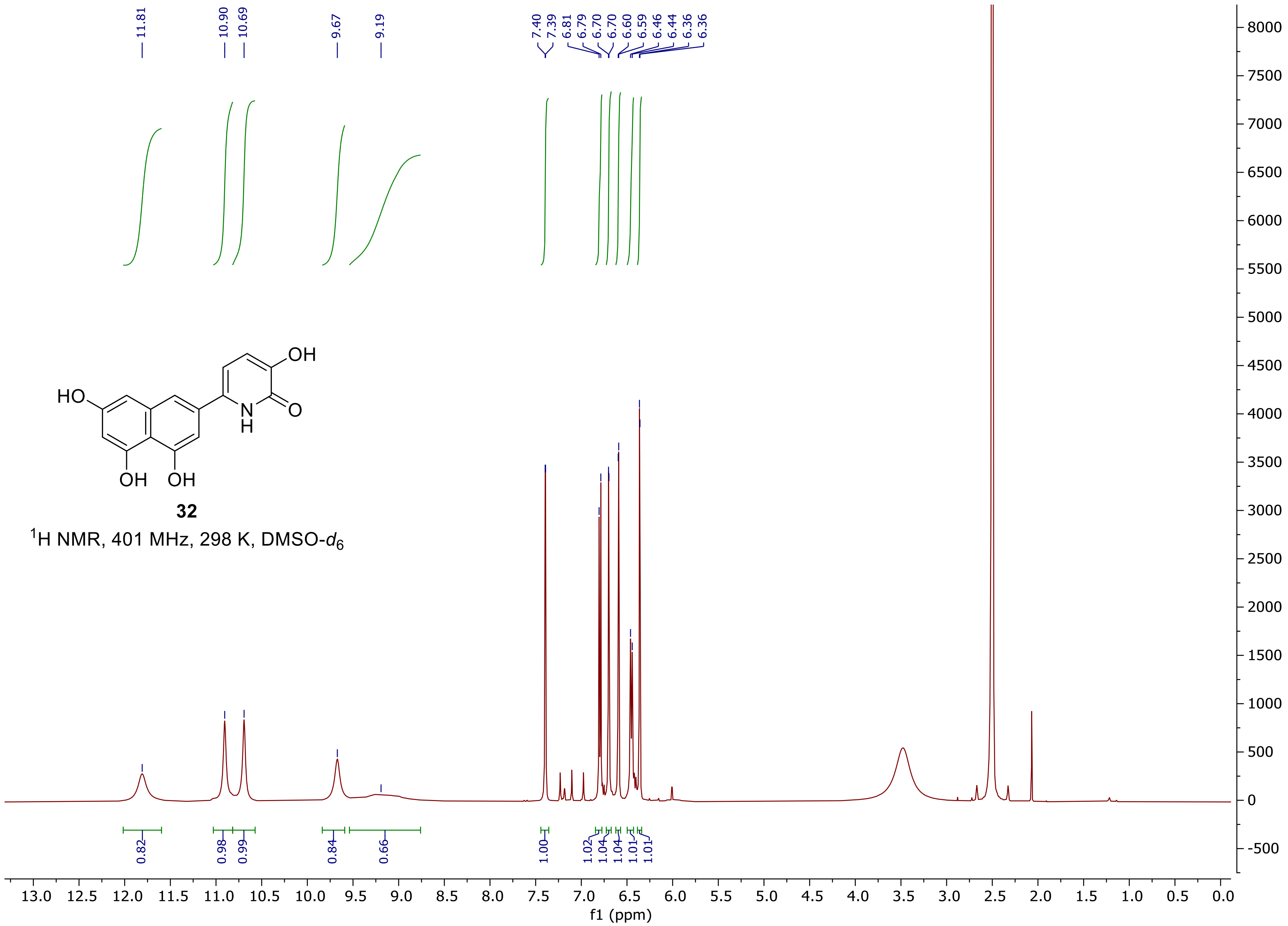

11.81
10.90
10.69
9.67
9.19
7.40
7.39
6.81
6.79
6.70
6.70
6.60
6.59
6.46
6.44
6.36
6.36
HO
OH
N
H
O
OH
OH
32
$^1$H NMR, 401 MHz, 298 K, DMSO-$d_6$
0.82
0.98
0.99
0.84
0.66
1.00
1.02
1.04
1.04
1.01
1.01
f1 (ppm)

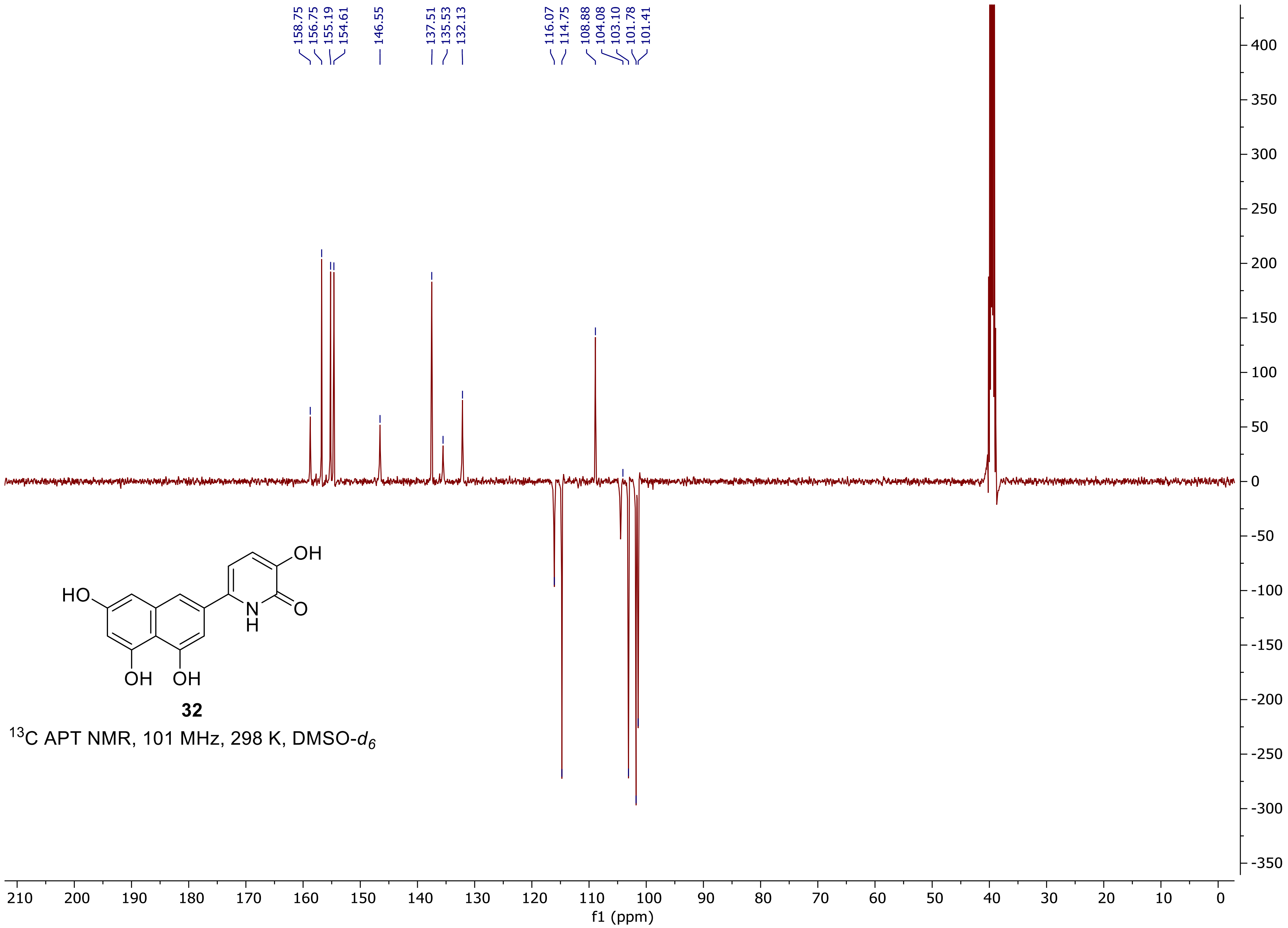


**32**

$^{13}$C APT NMR, 101 MHz, 298 K, DMSO-$d_6$

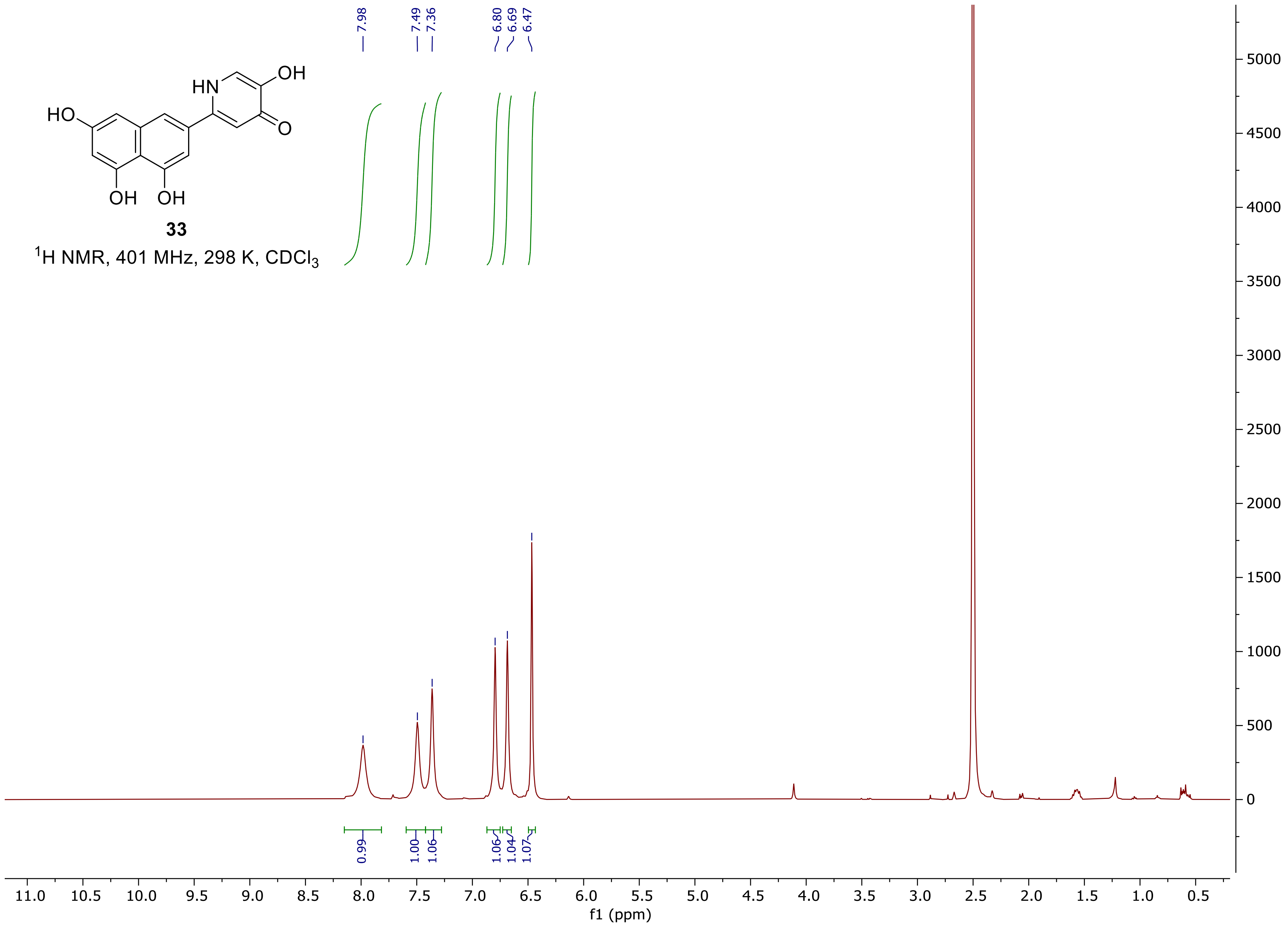

HO
HN
OH
O
OH OH
33
$^{1}$H NMR, 401 MHz, 298 K, $CDCl_3$
7.98
7.49
7.36
6.80
6.69
6.47
0.99
1.00
1.06
1.06
1.04
1.07
f1 (ppm)

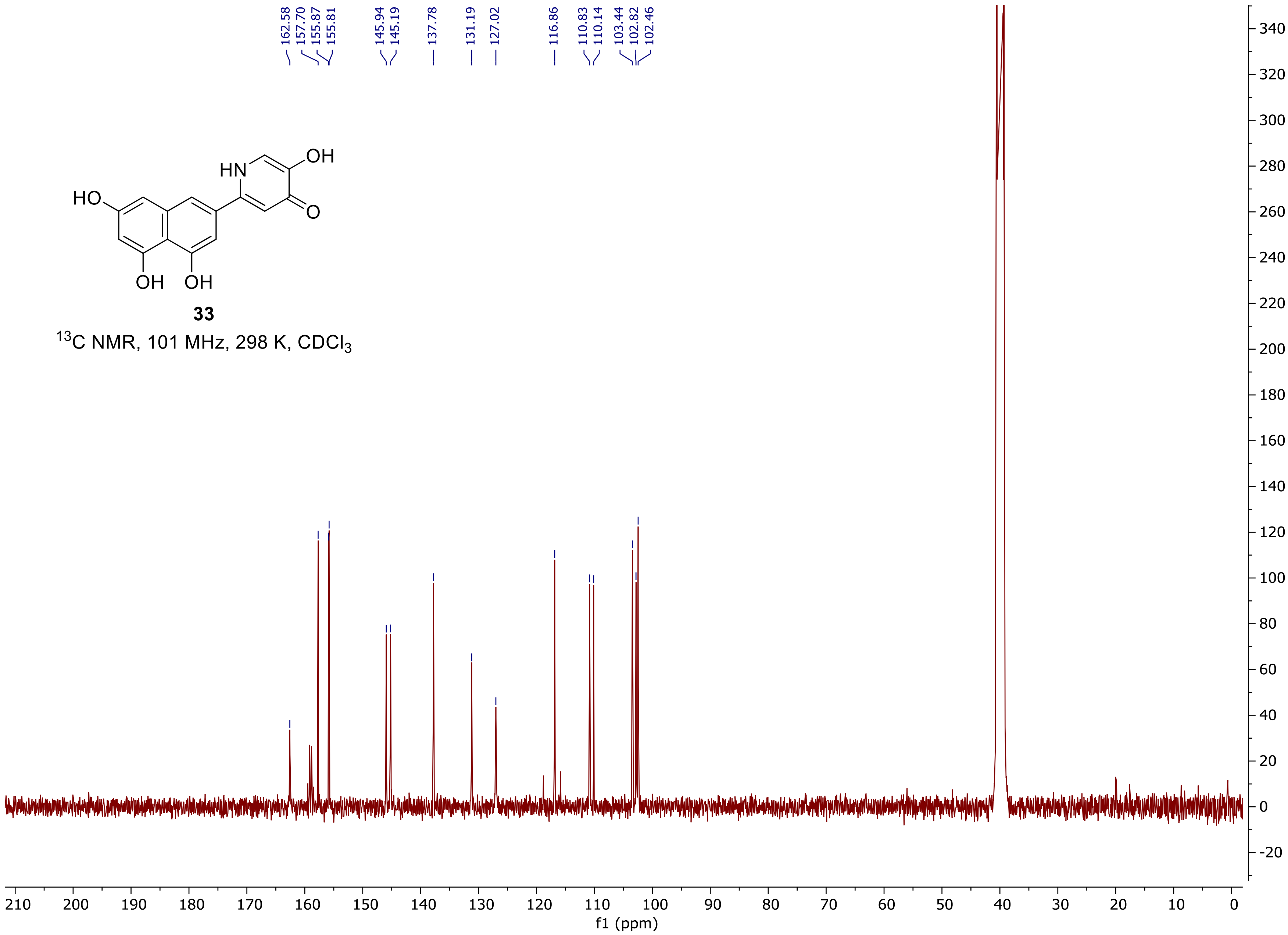


**33**

$^{13}$C NMR, 101 MHz, 298 K, $CDCl_3$

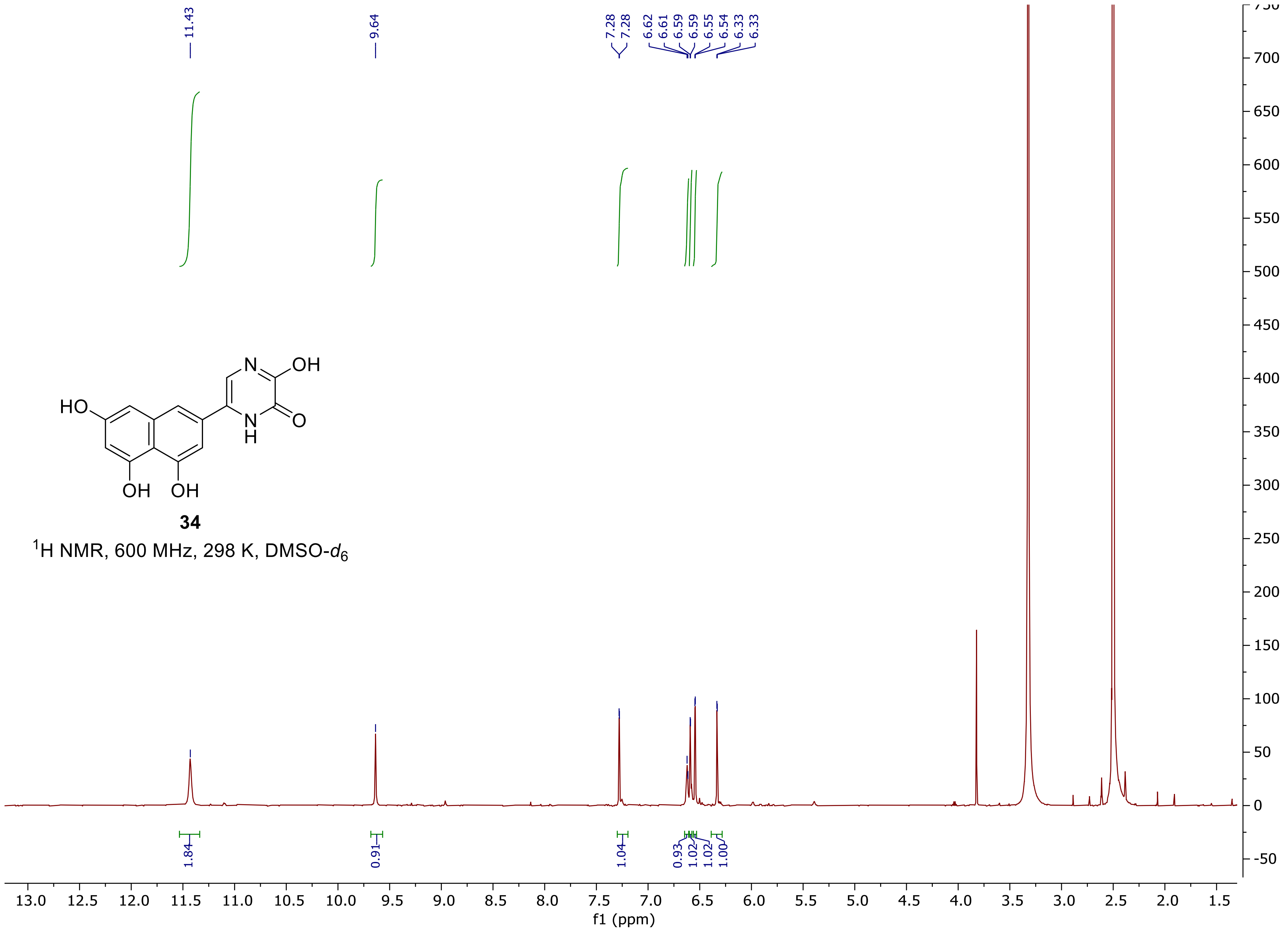


**34**

$^{1}$H NMR, 600 MHz, 298 K, DMSO-$d_6$

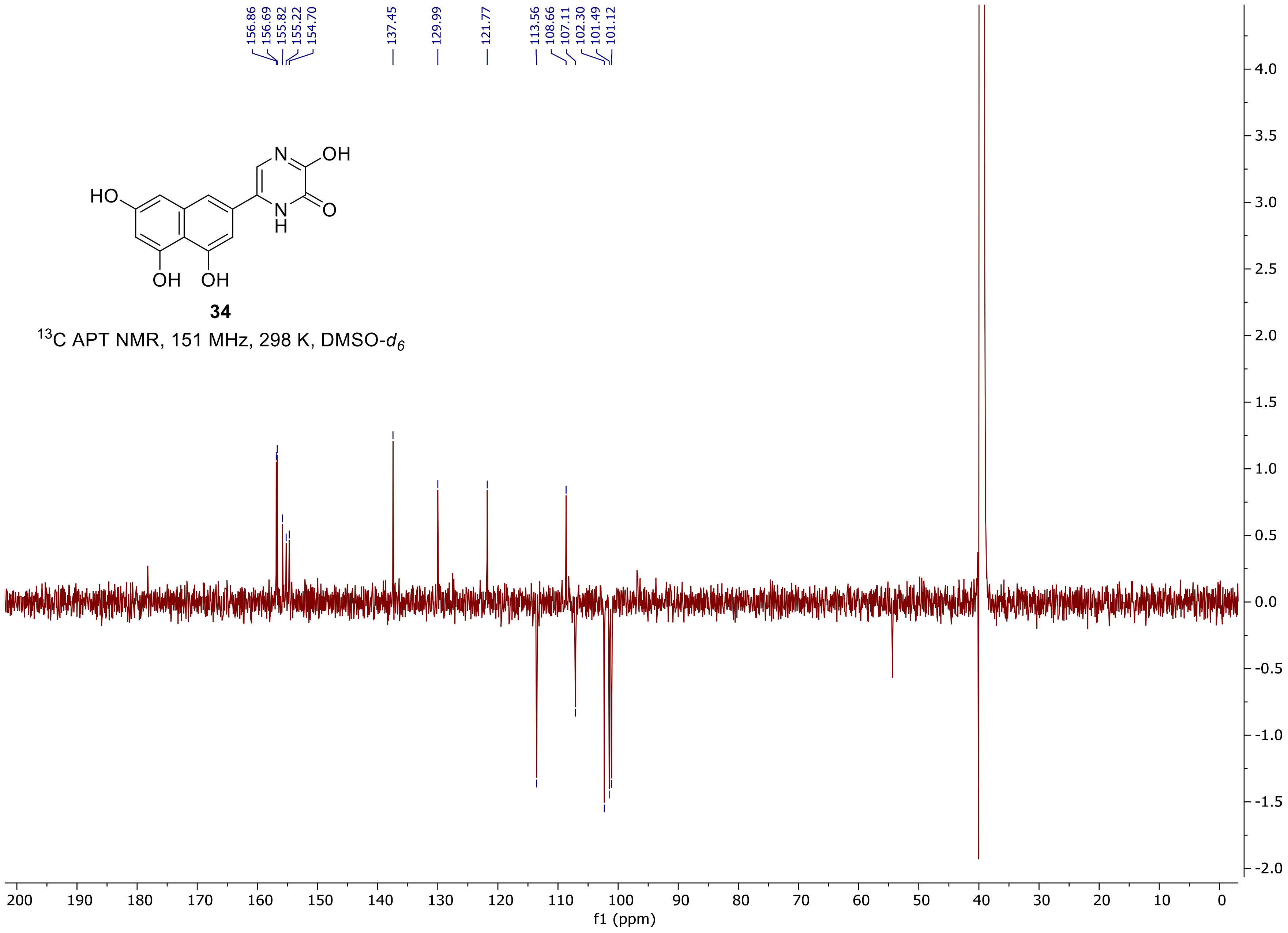


**34**

$^{13}$C APT NMR, 151 MHz, 298 K, DMSO-$d_6$

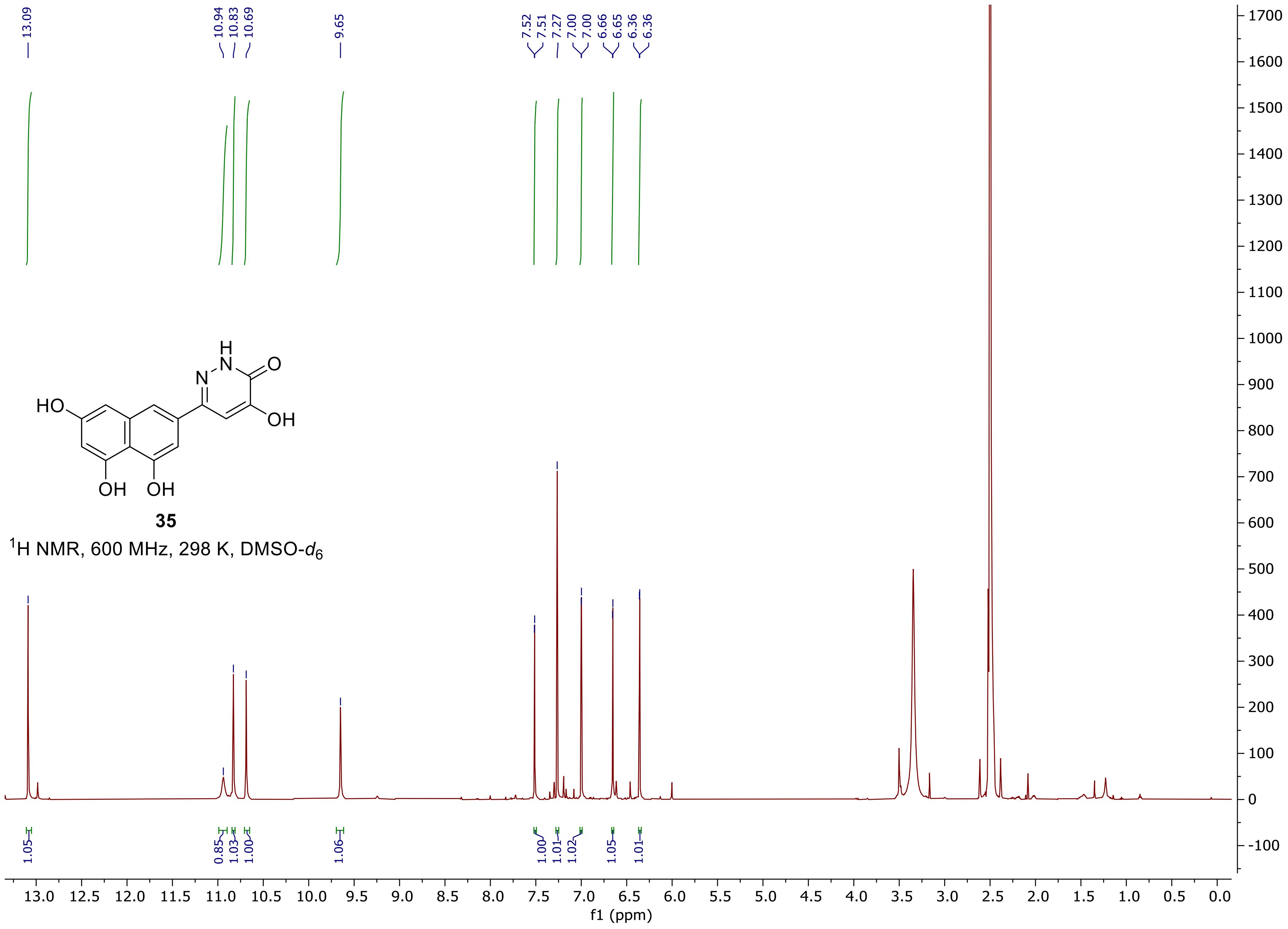
13.09
10.94
10.83
10.69
9.65
7.52
7.51
7.27
7.00
7.00
6.66
6.65
6.36
6.36
35
$^1$H NMR, 600 MHz, 298 K, DMSO-$d_6$
1.05
0.85
1.03
1.00
1.06
1.00
1.01
1.02
1.05
1.01
f1 (ppm)

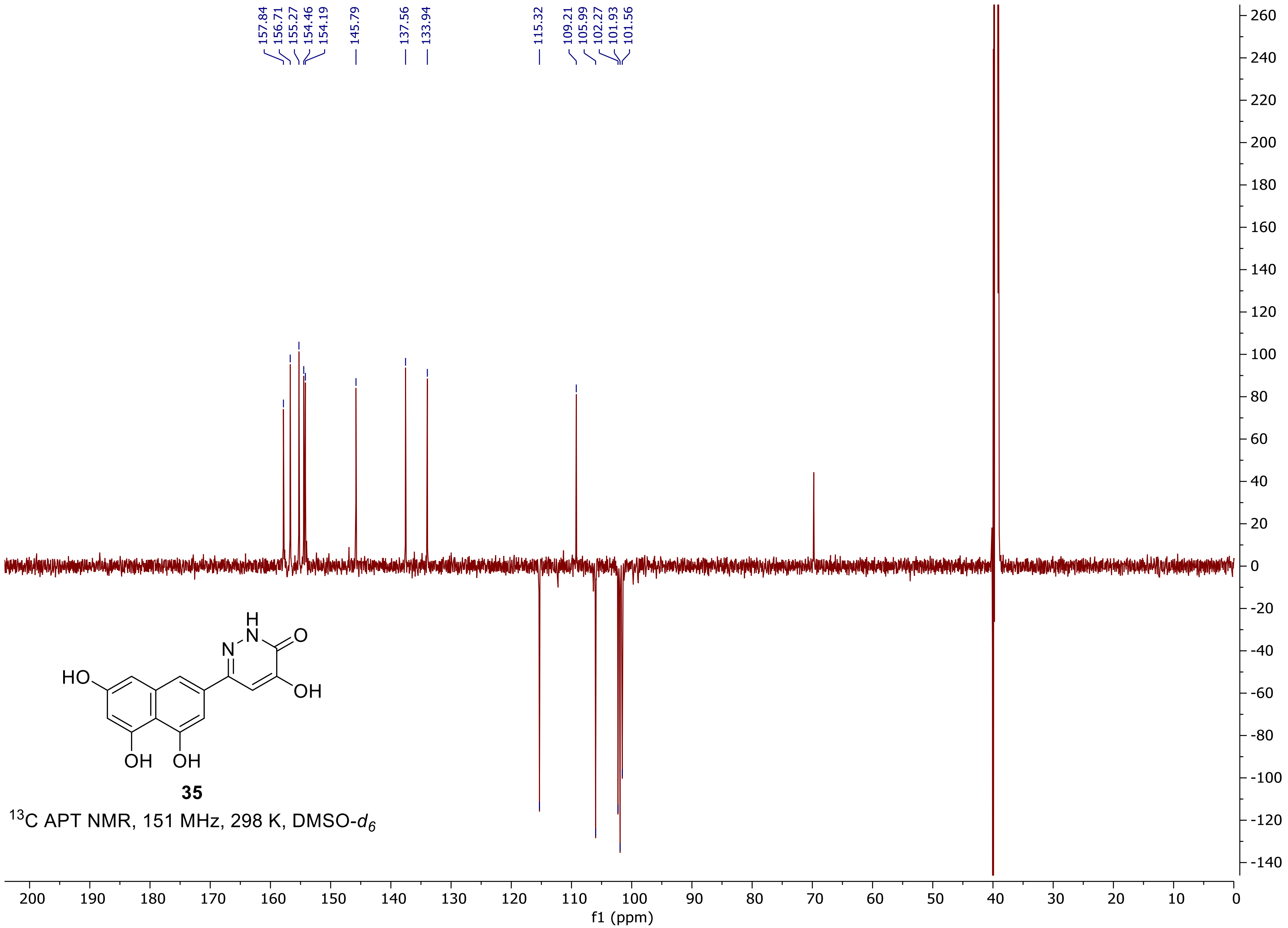


**35**

$^{13}$C APT NMR, 151 MHz, 298 K, DMSO-$d_6$

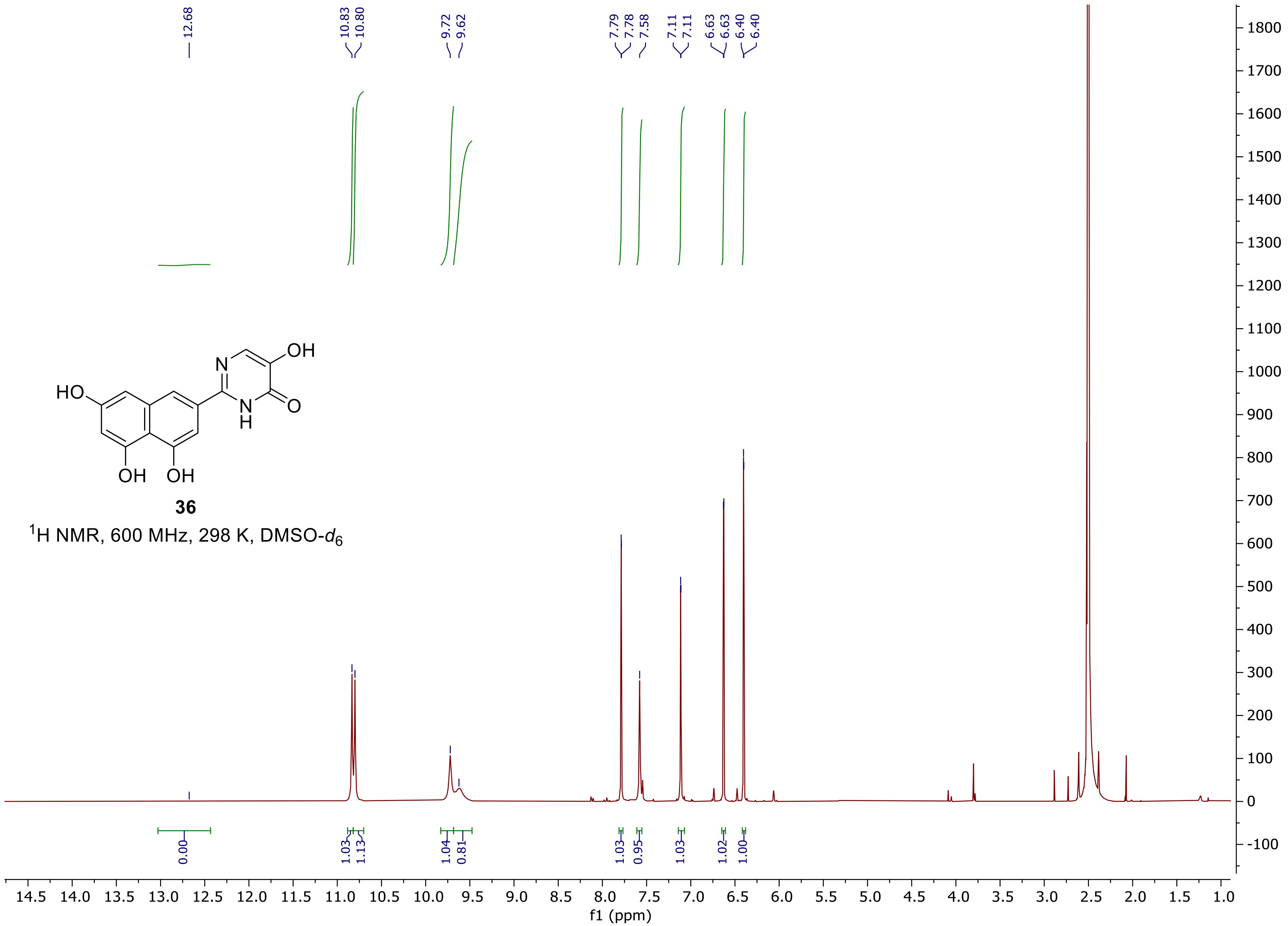

12.68
10.83
10.80
9.72
9.62
7.79
7.78
7.58
7.11
7.11
6.63
6.63
6.40
6.40
0.00
1.03
1.13
1.04
0.81
1.03
0.95
1.03
1.02
1.00
HO
OH
N
N
H
O
OH OH
36
$^{1}$H NMR, 600 MHz, 298 K, DMSO-$d_6$
f1 (ppm)

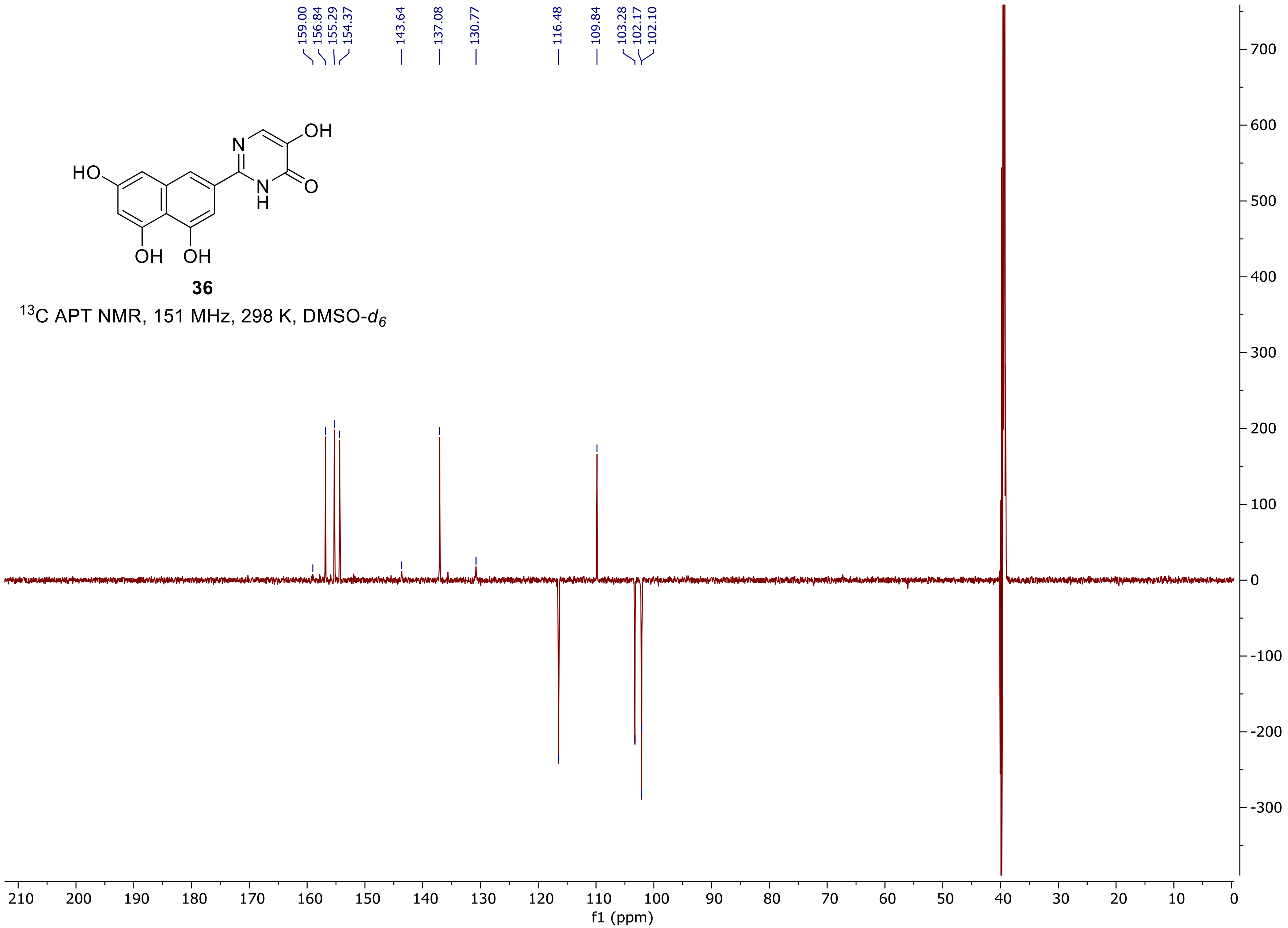


36

$^{13}C$ APT NMR, 151 MHz, 298 K, DMSO-$d_6$

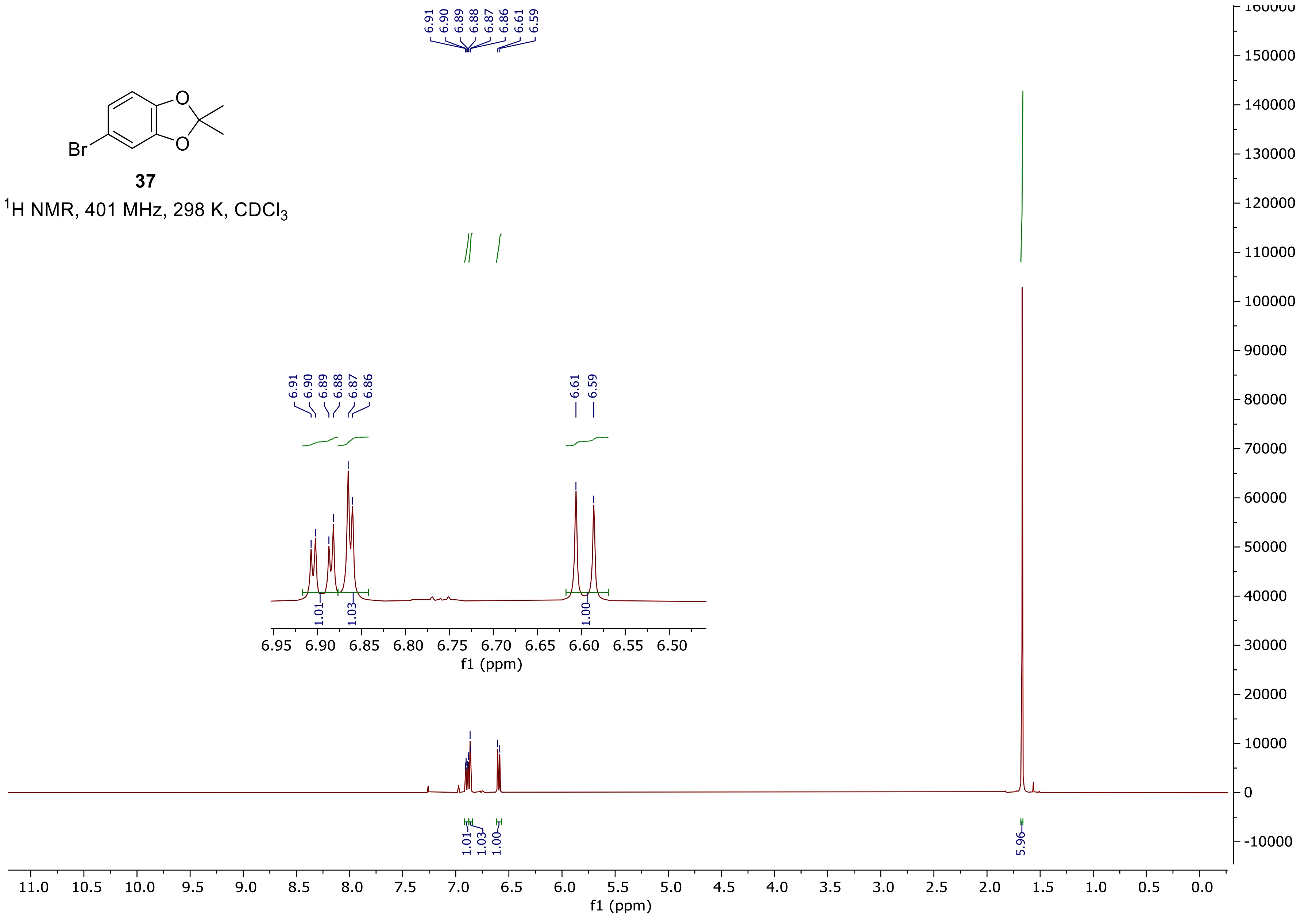

Br
O
O
37
$^1$H NMR, 401 MHz, 298 K, $CDCl_3$
6.91
6.90
6.89
6.88
6.87
6.86
6.61
6.59
1.01
1.03
1.00
5.96
f1 (ppm)

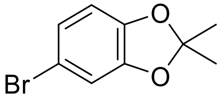


**37**

$^{13}$C NMR, 101 MHz, 298 K, $CDCl_3$

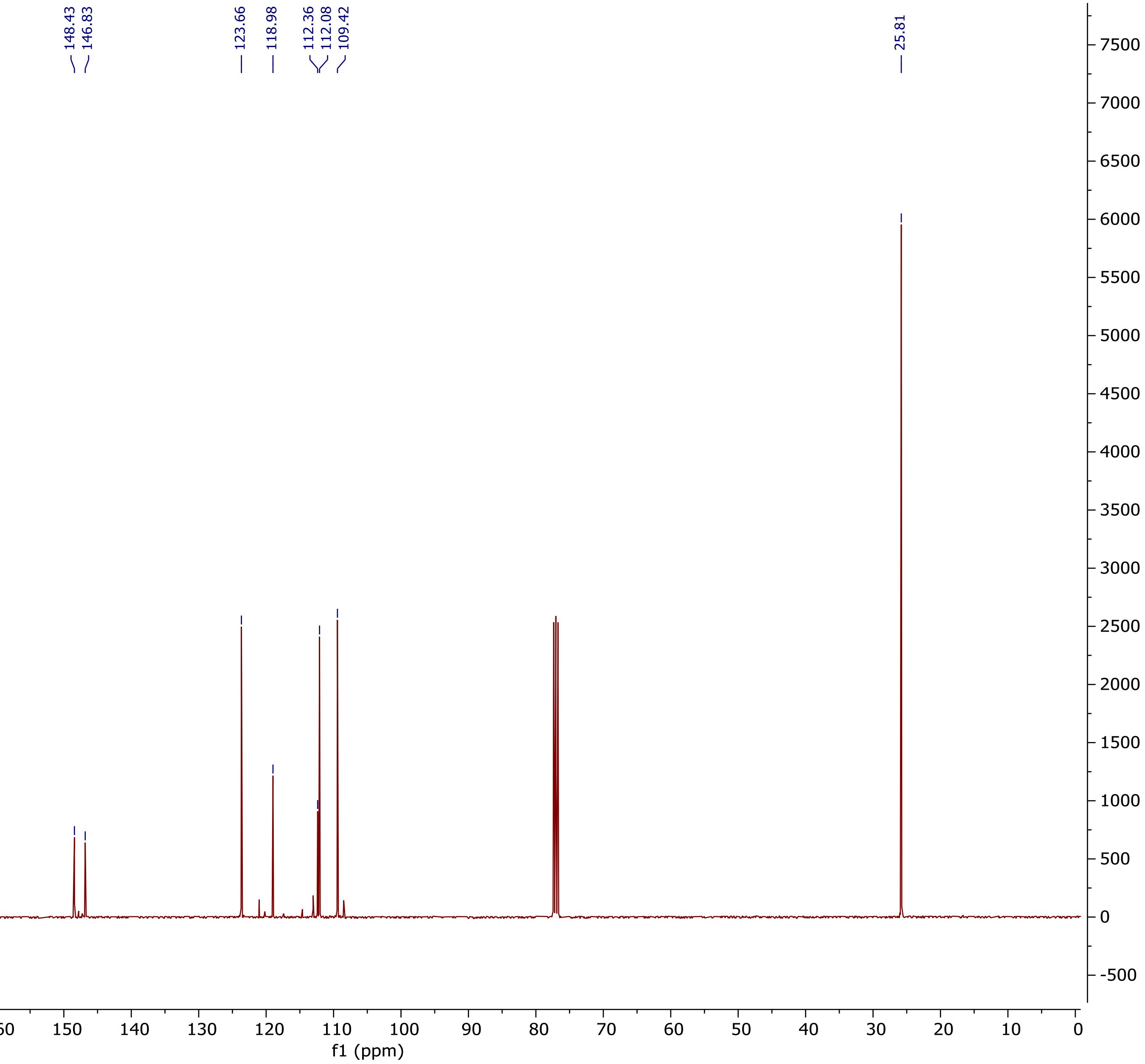

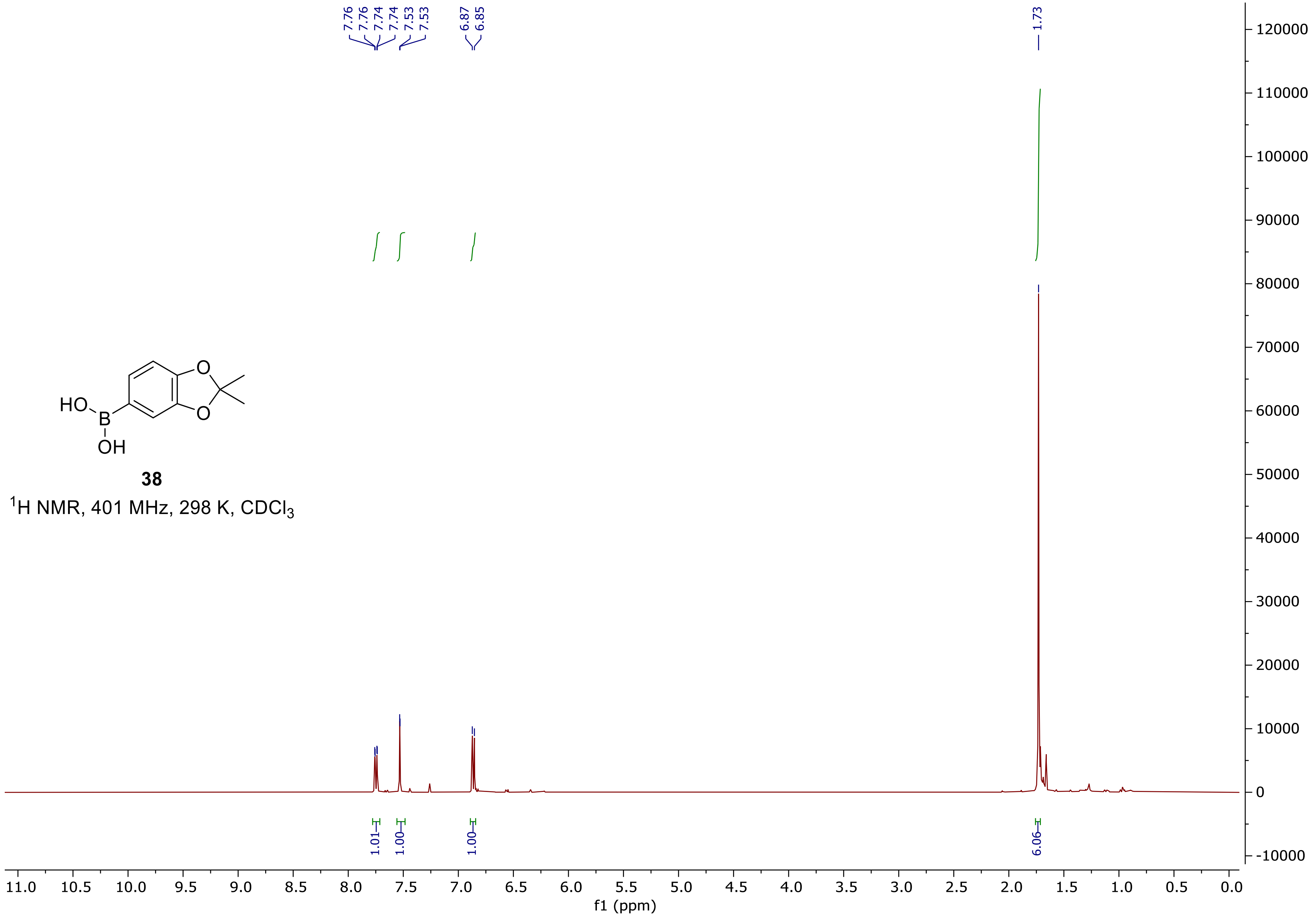


**38**

$^{1}H$ NMR, 401 MHz, 298 K, $CDCl_3$

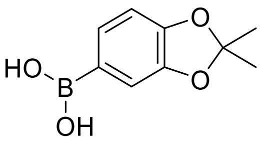

**38**

$^{13}C$ NMR, 101 MHz, 298 K, $CDCl_3$

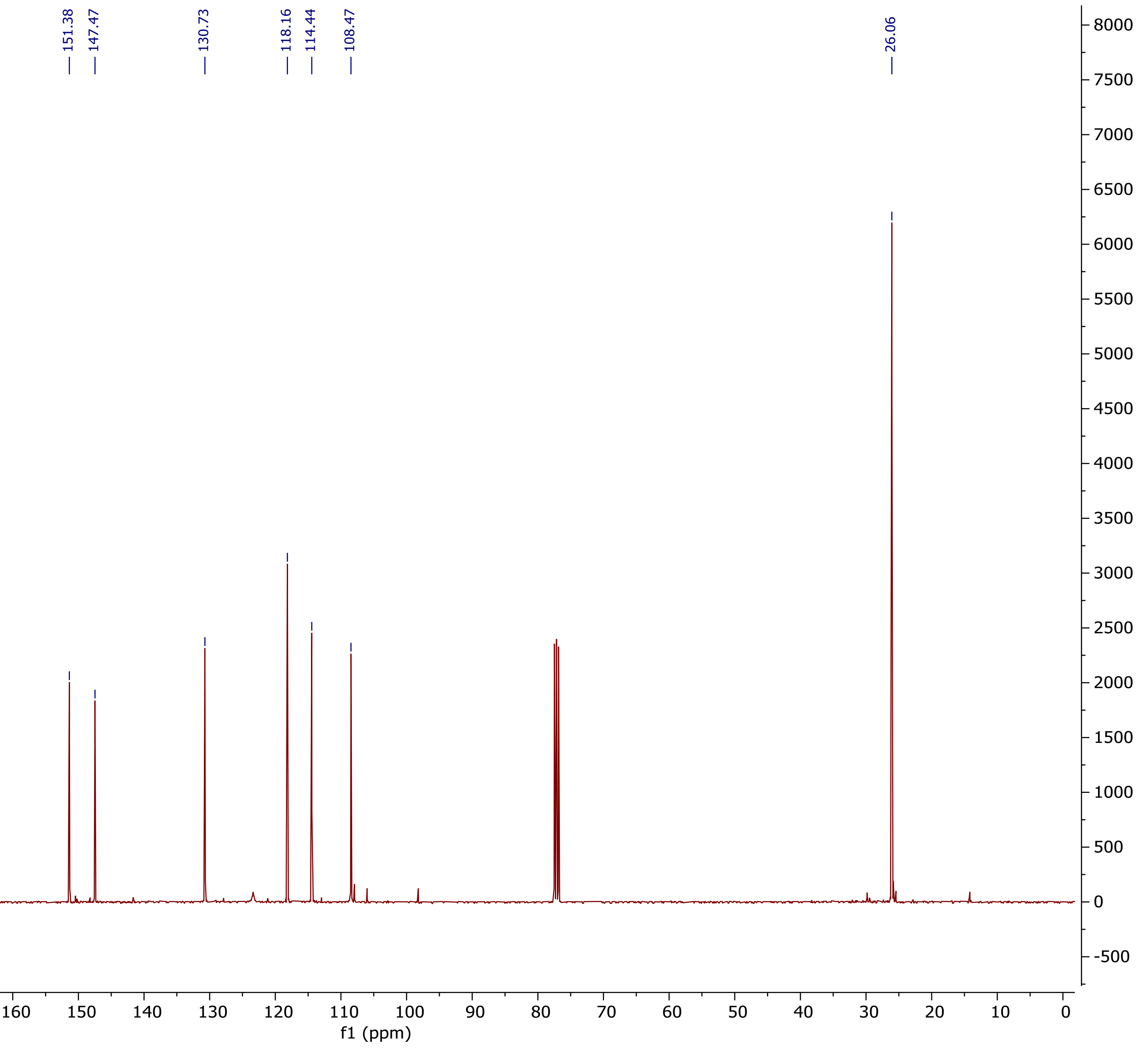

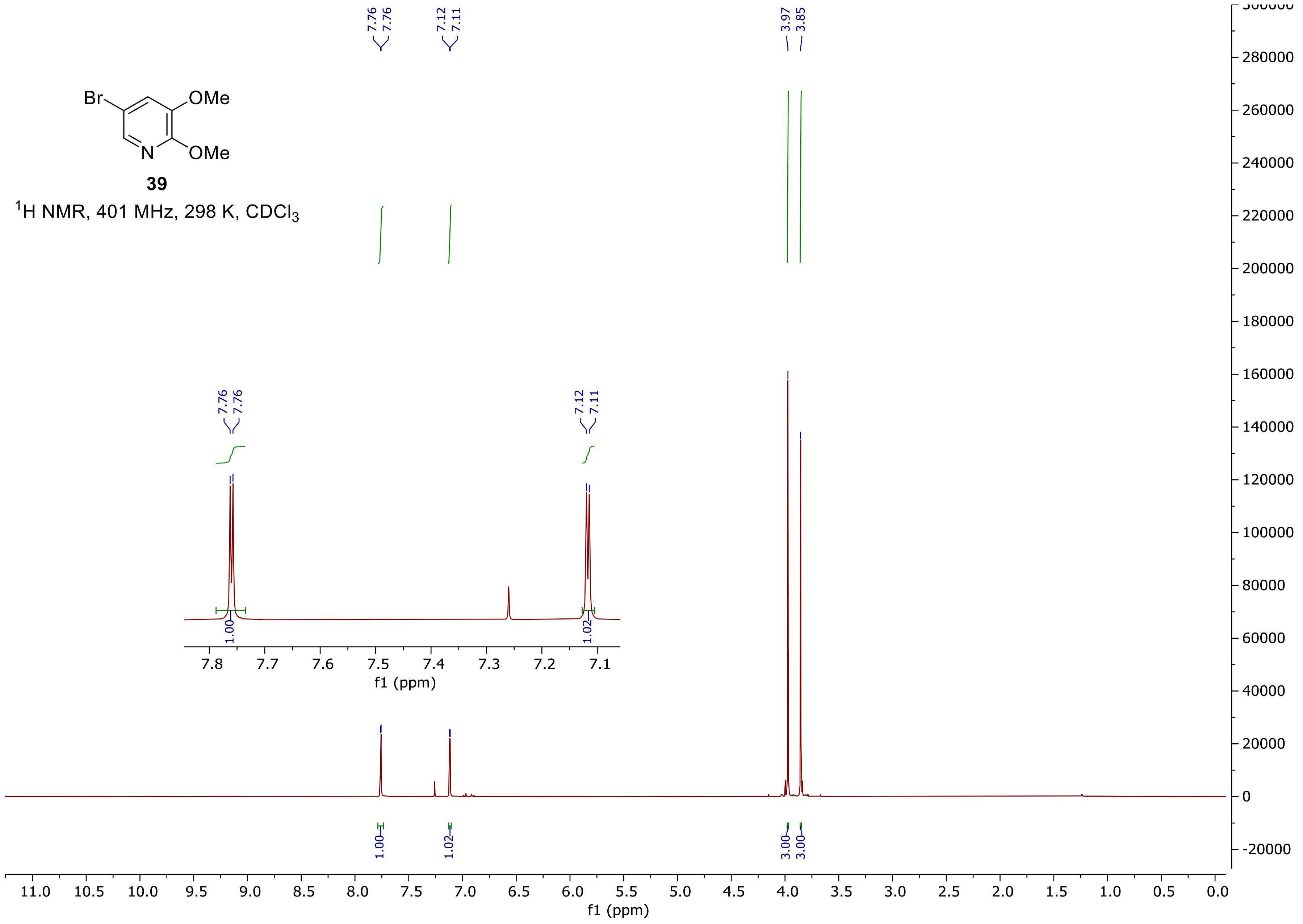
Br
OMe
N
OMe
39
$^{1}$H NMR, 401 MHz, 298 K, CDCl$_3$
7.76
7.76
7.12
7.11
3.97
3.85
1.00
1.02
3.00
3.00
f1 (ppm)

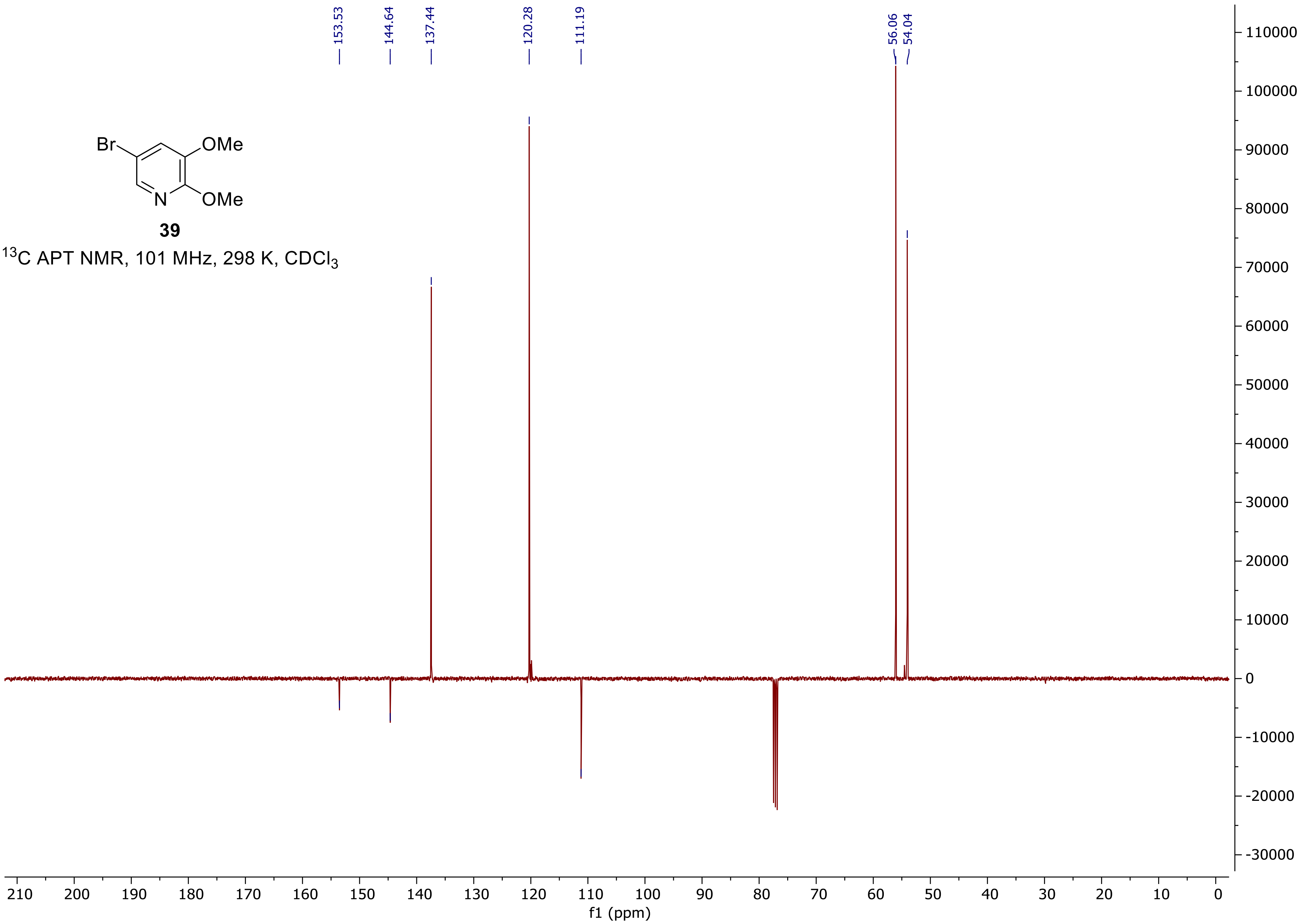


**39**

$^{13}$C APT NMR, 101 MHz, 298 K, $CDCl_3$

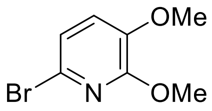


**40**

$^{1}$H NMR, 401 MHz, 298 K, $CDCl_3$

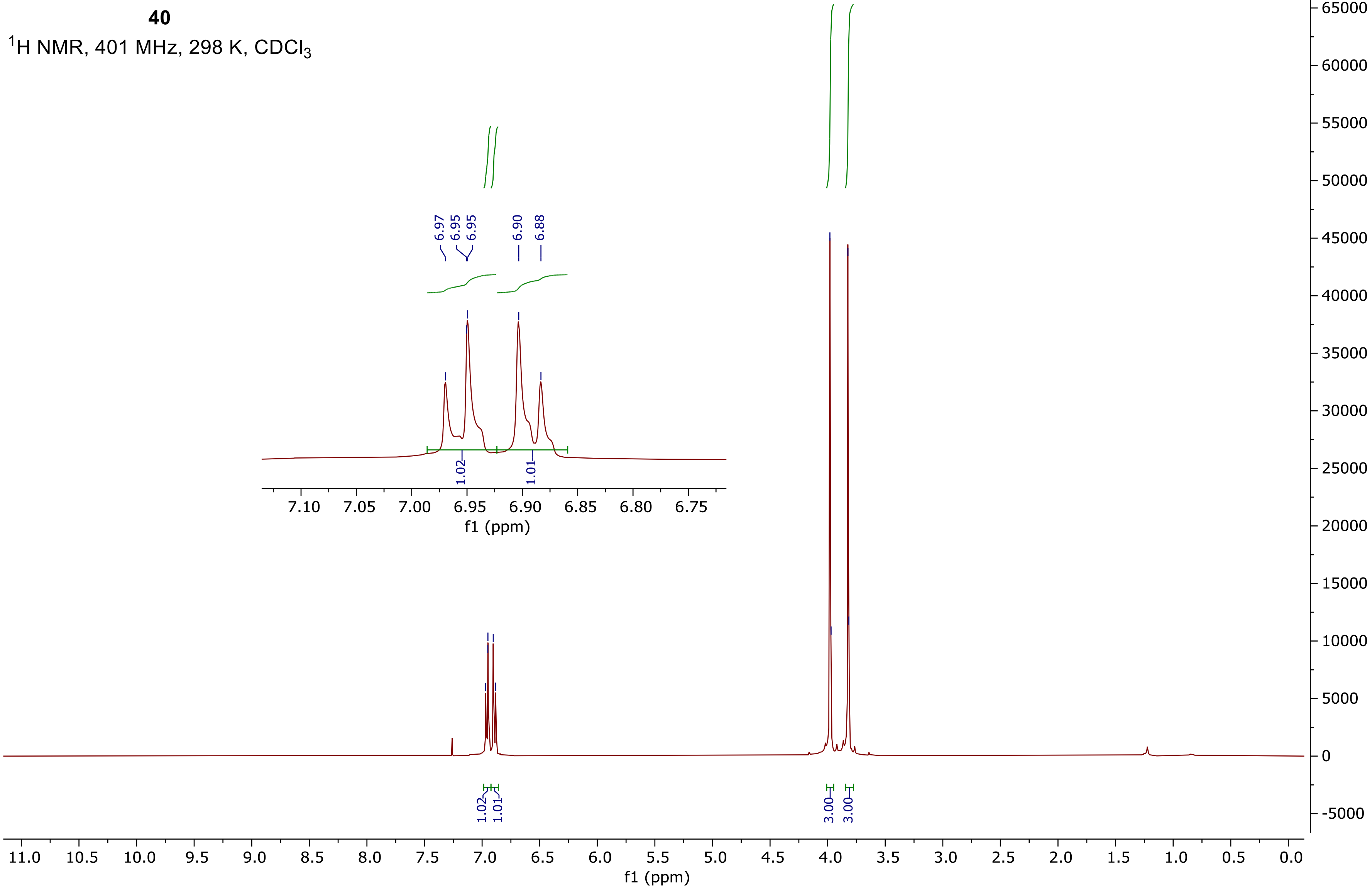

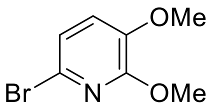

**40**

$^{13}$C APT NMR, 101 MHz, 298 K, CDCl3

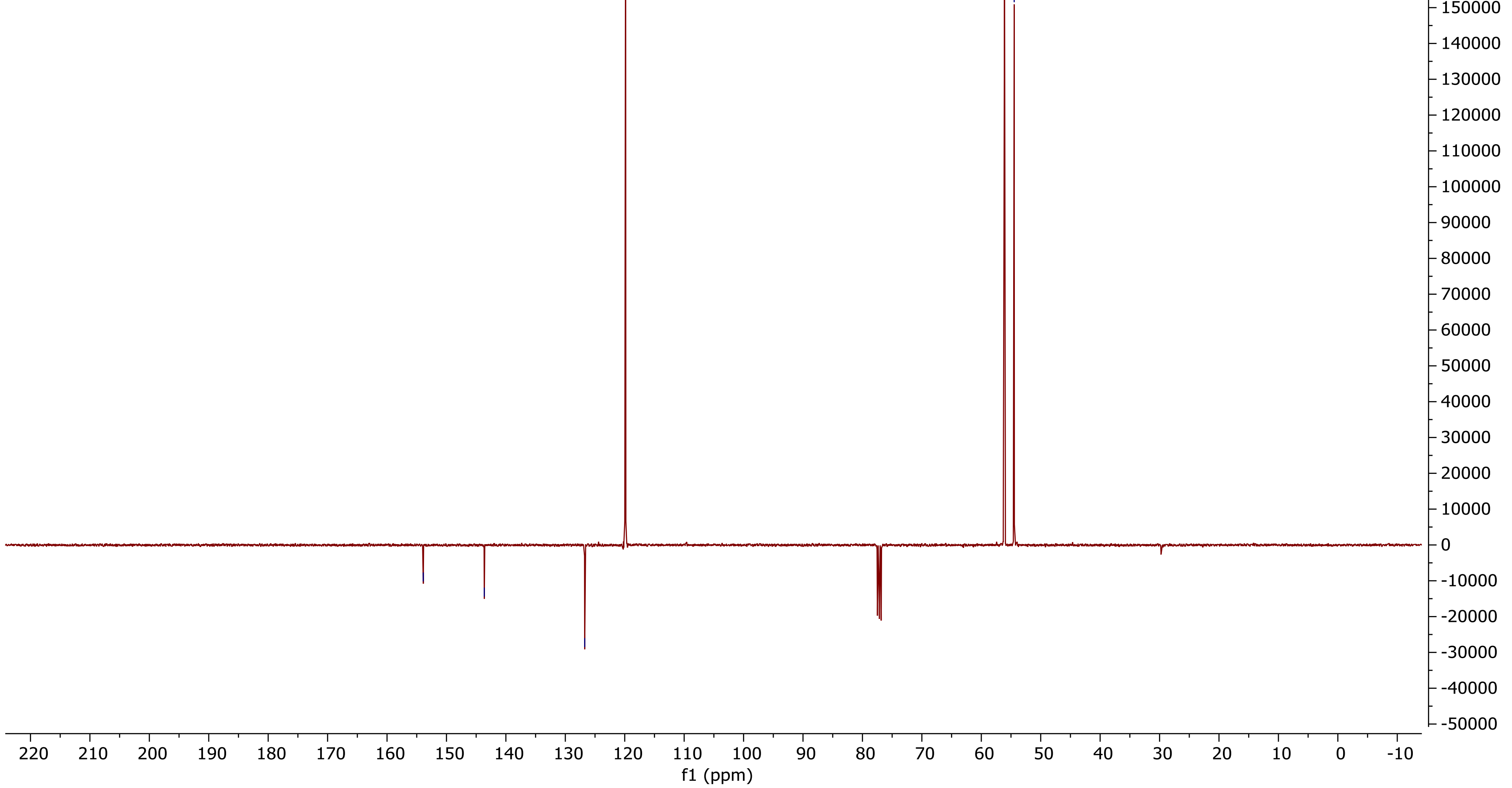

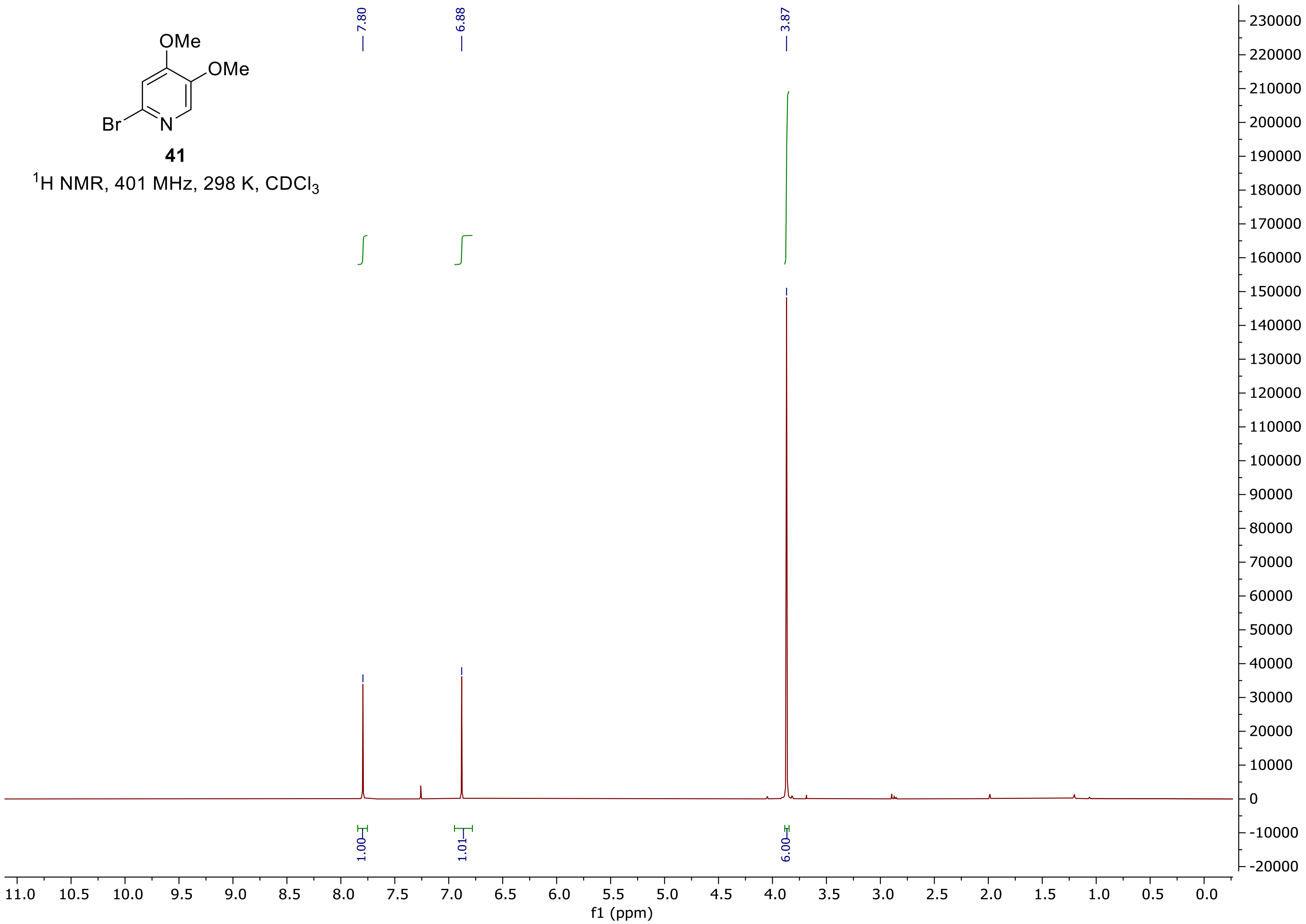

OMe
OMe
Br
N
41
¹H NMR, 401 MHz, 298 K, CDCl₃
7.80
6.88
3.87
1.00
1.01
6.00
f1 (ppm)

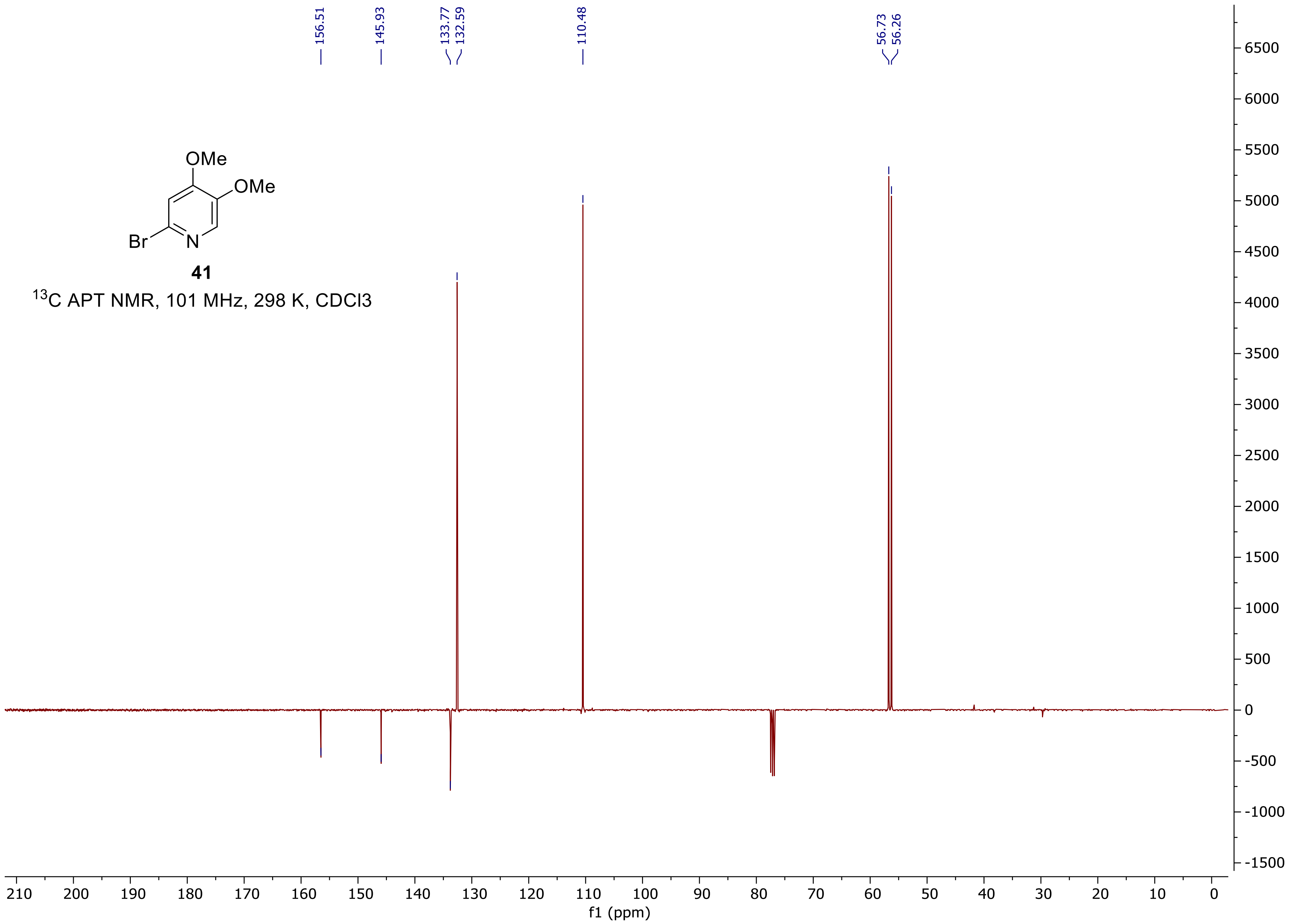


**41**

$^{13}$C APT NMR, 101 MHz, 298 K, CDCl3

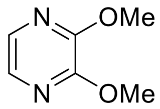


**42**

$^1$H NMR, 401 MHz, 298 K, $CDCl_3$

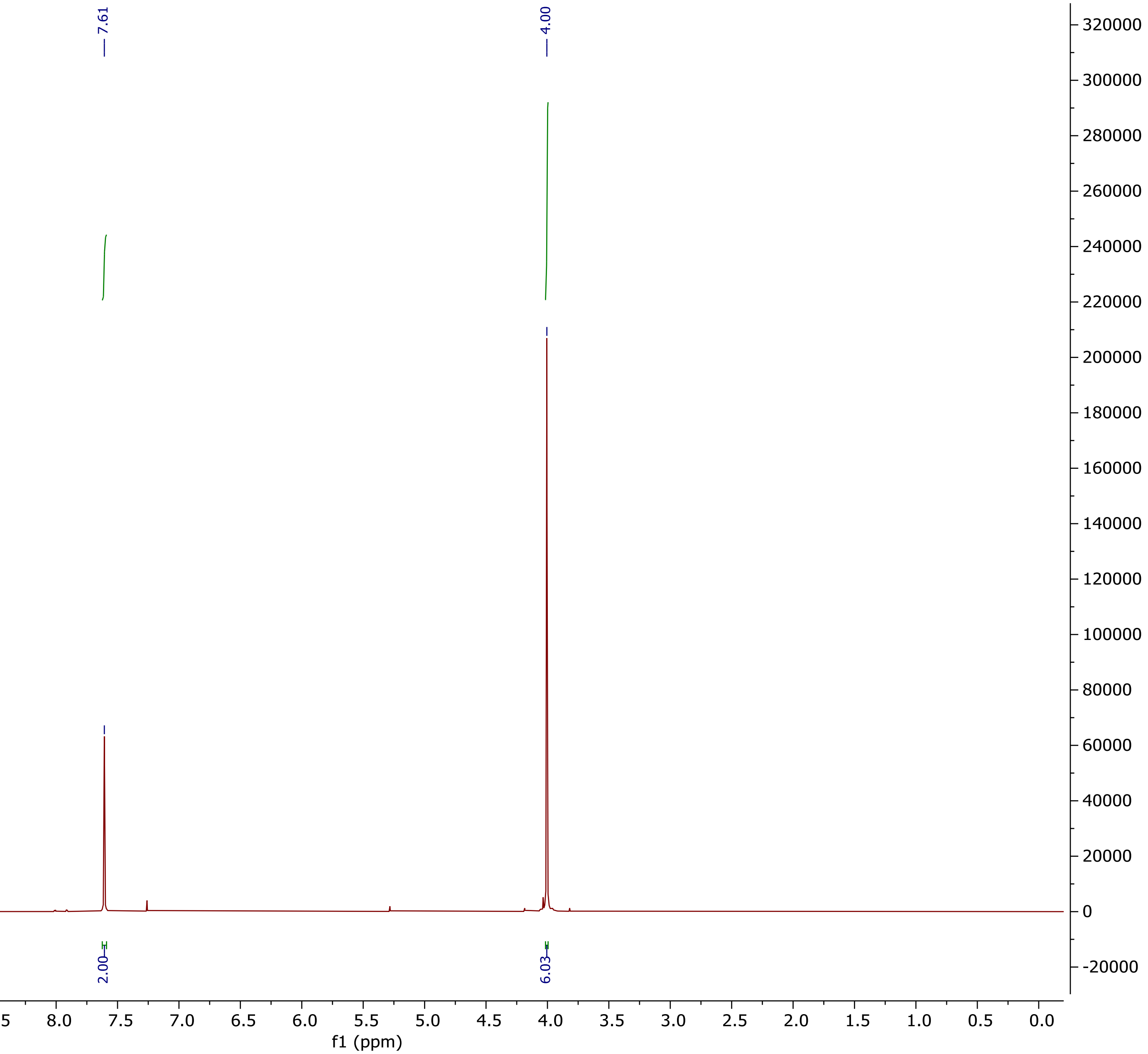

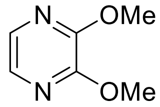

**42**

$^{13}$C NMR, 101 MHz, 298 K, $CDCl_3$

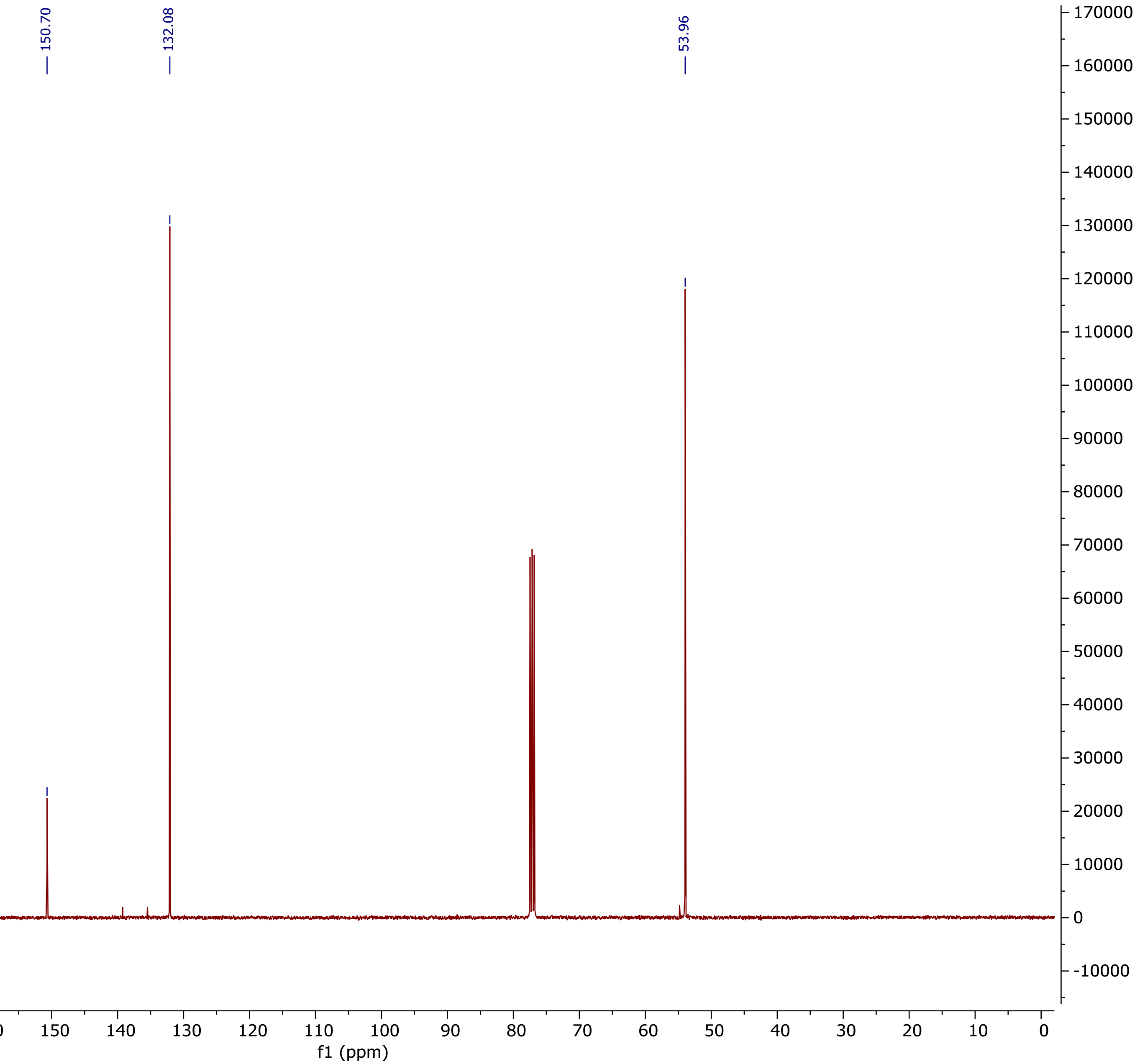

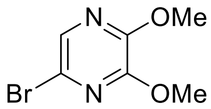

**43**

$^1H$ NMR, 401 MHz, 298 K, $CDCl_3$

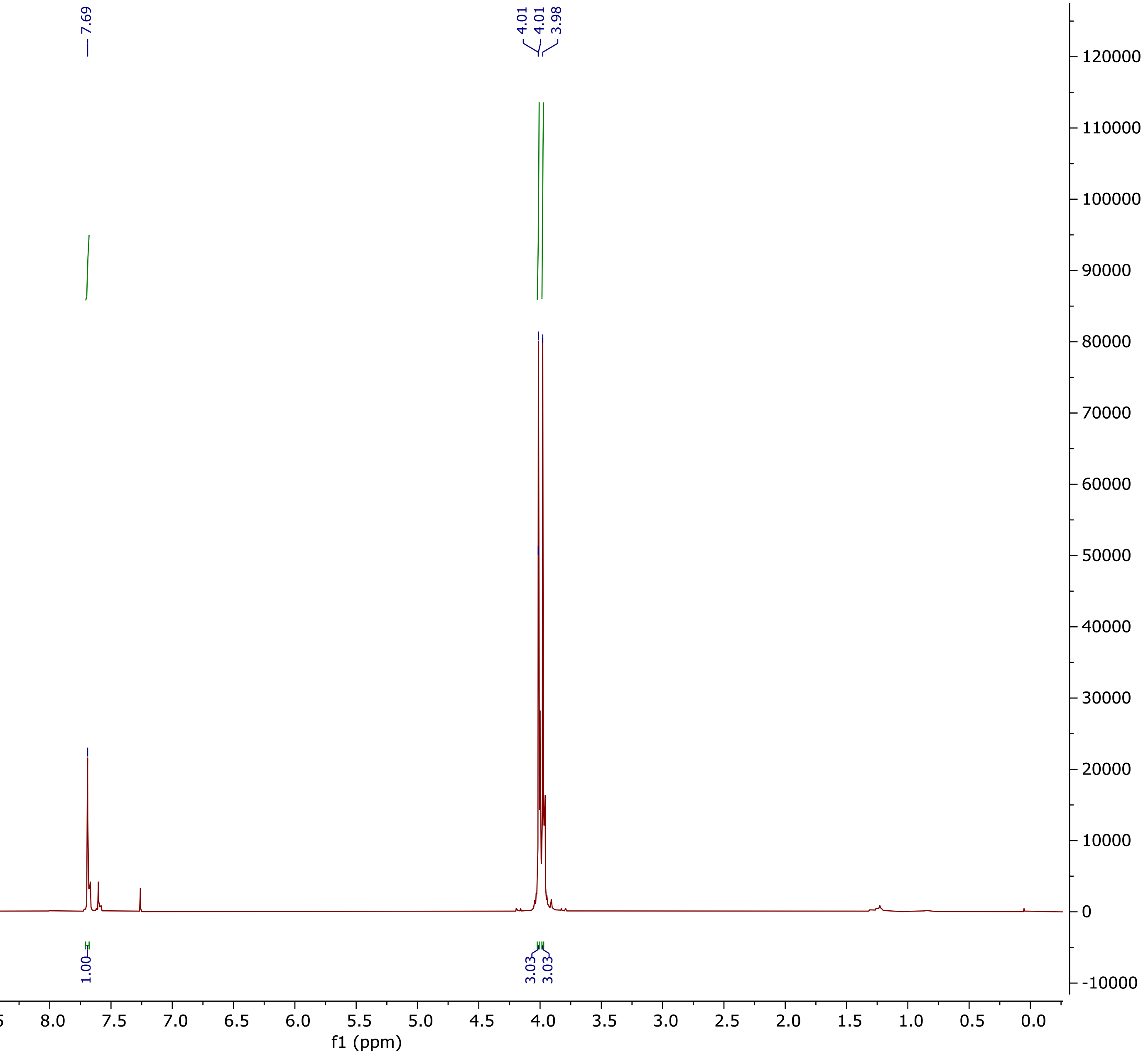

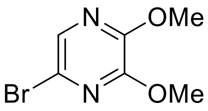


**43**

$^{13}C$ NMR, 101 MHz, 298 K, $CDCl_3$

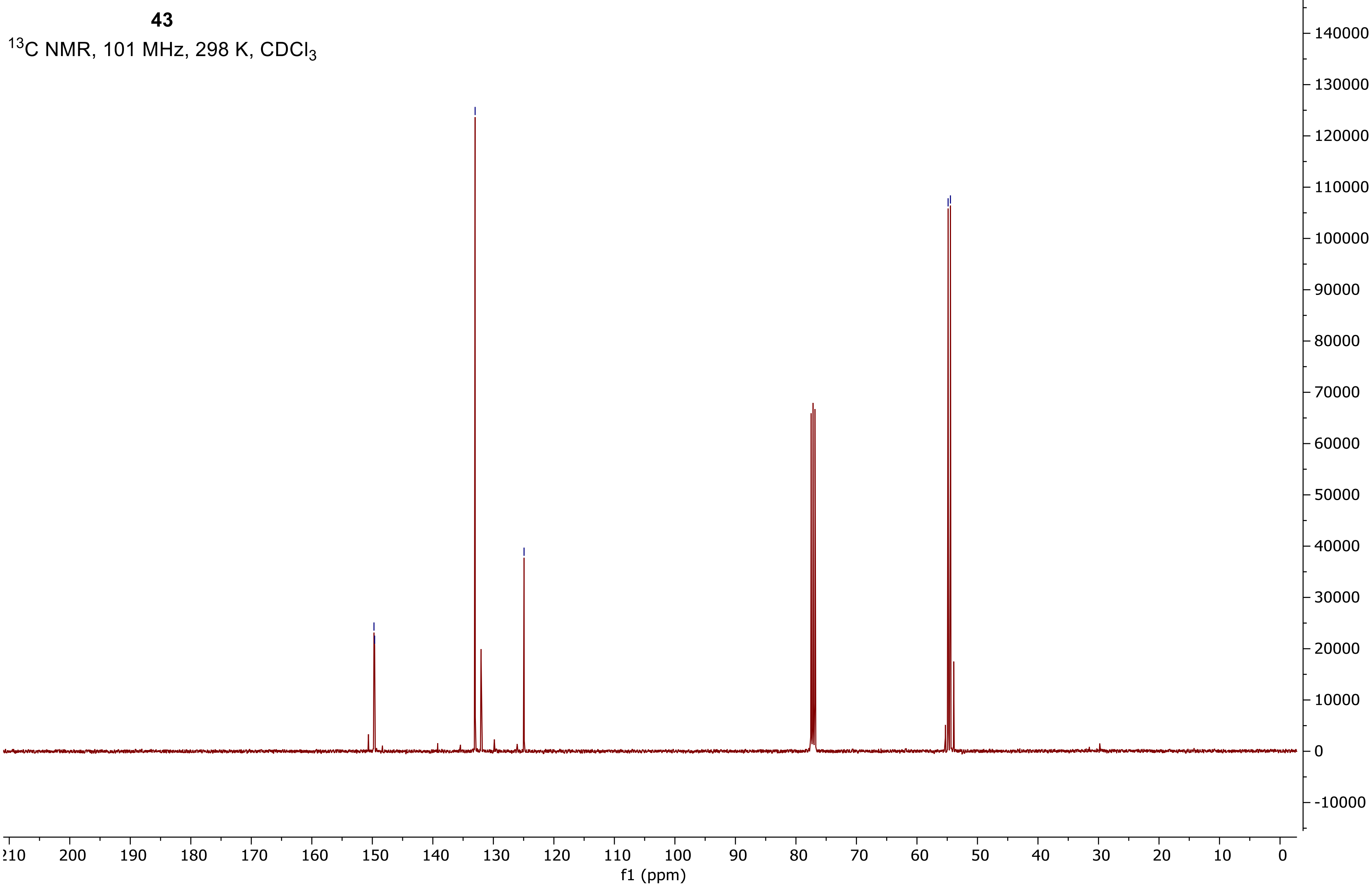

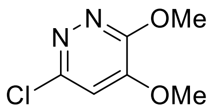

**44**

$^1H$ NMR, 401 MHz, 298 K, $CDCl_3$

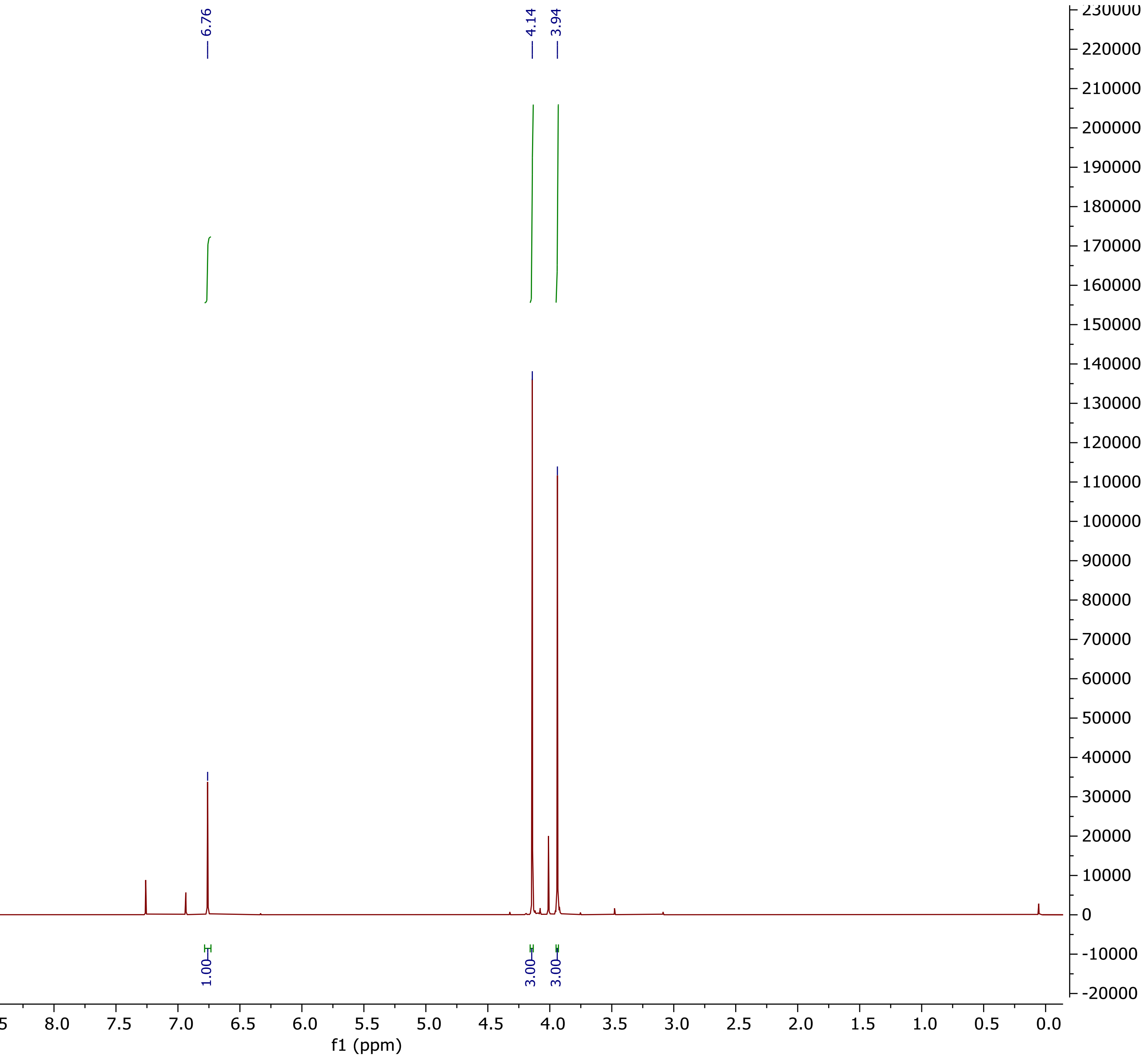

**44**

$^{13}C$ NMR, 101 MHz, 298 K, $CDCl_3$

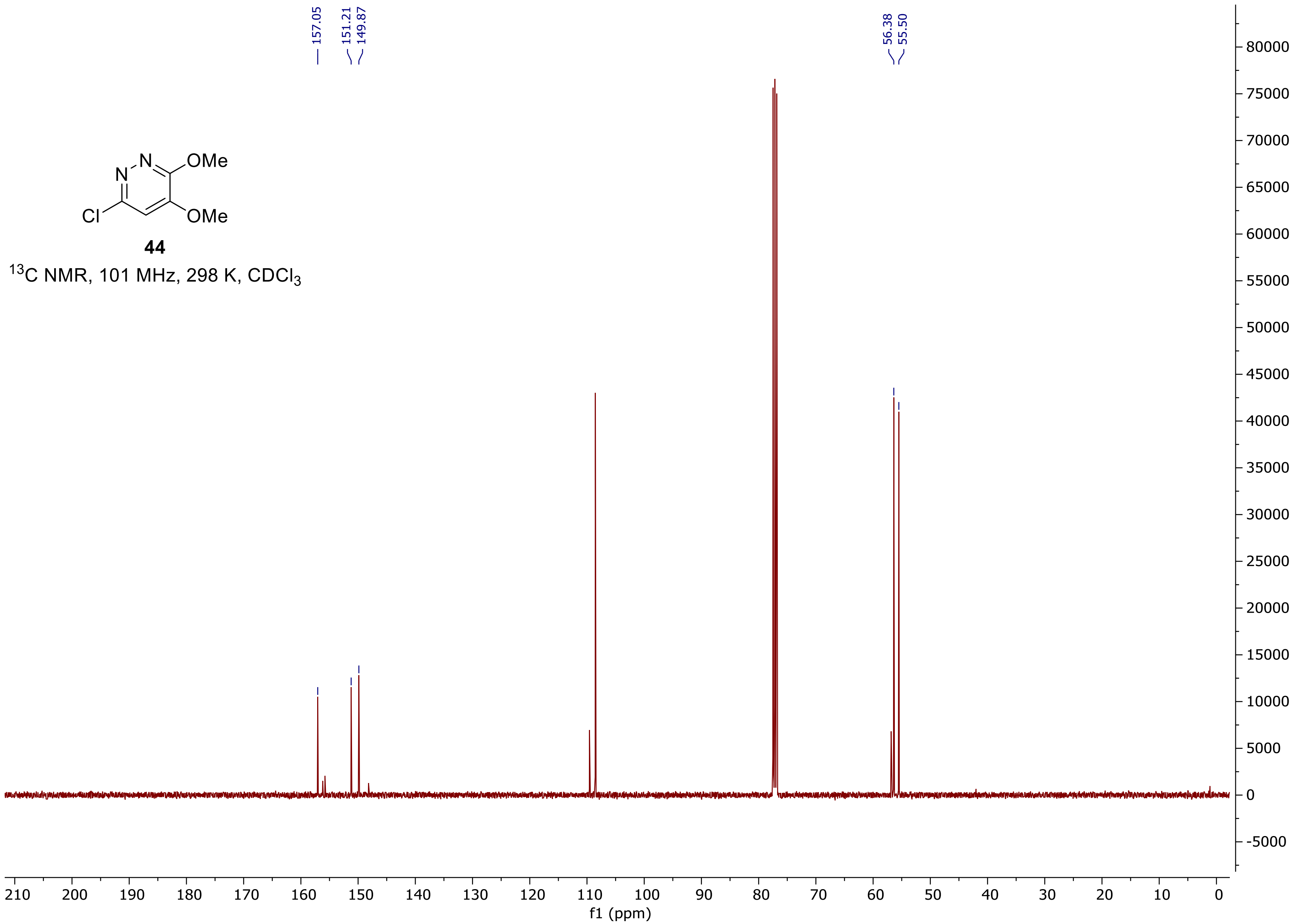

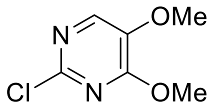

**45**

$^1$H NMR, 401 MHz, 298 K, $CDCl_3$

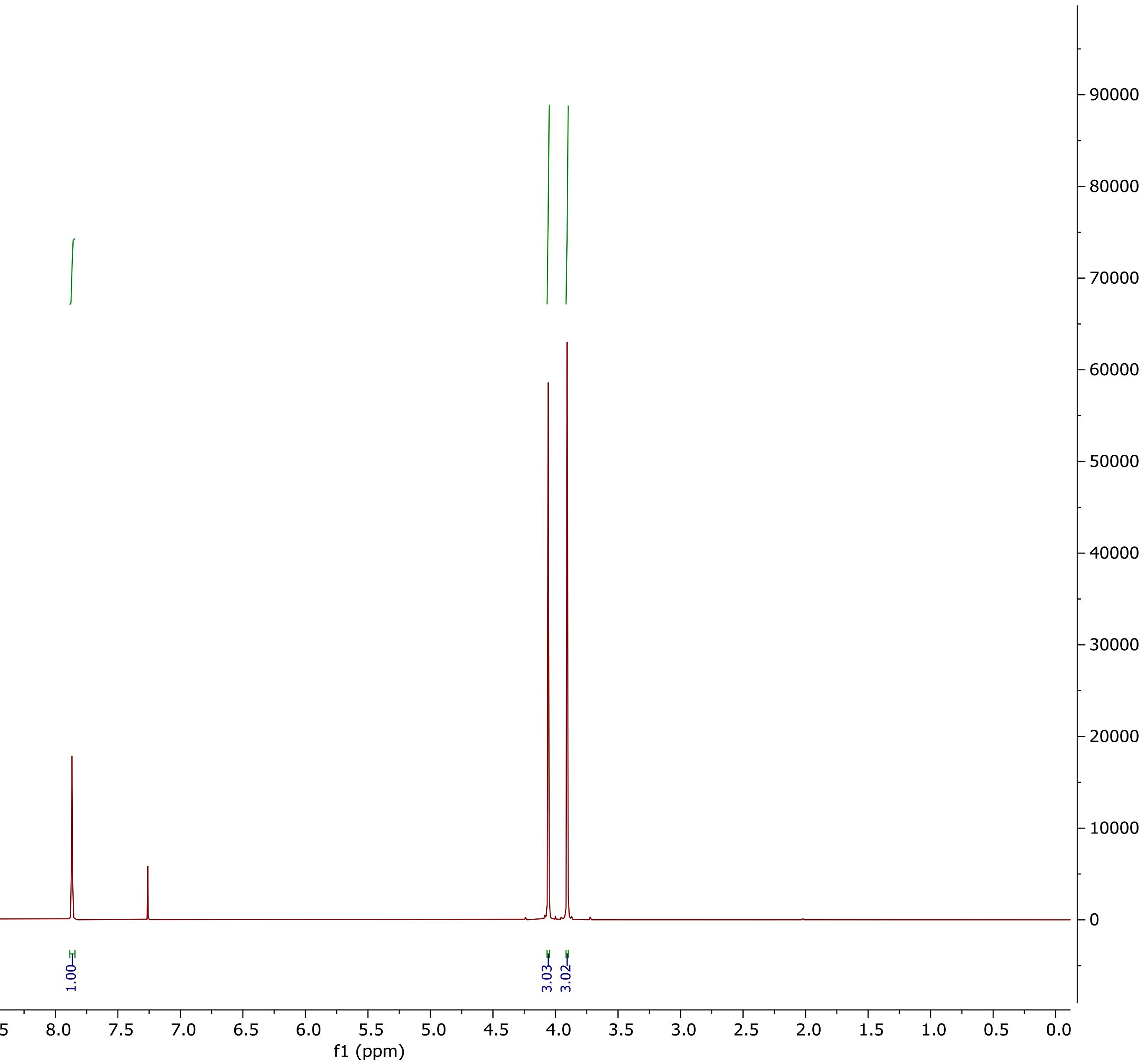

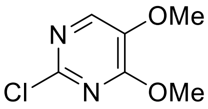

**45**

$^{13}C$ NMR, 101 MHz, 298 K, $CDCl_3$

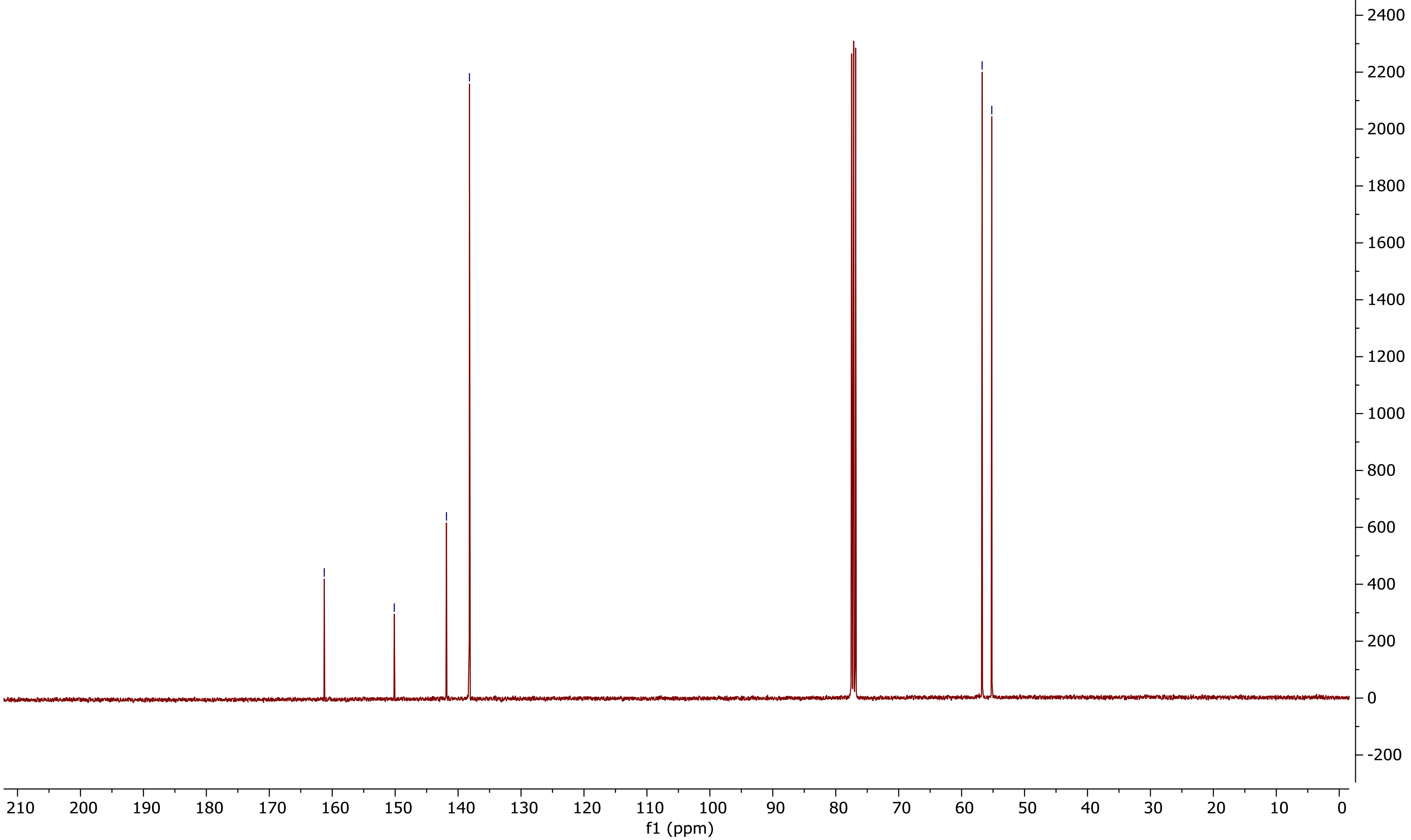

## IR spectra of pseudoflavonoids

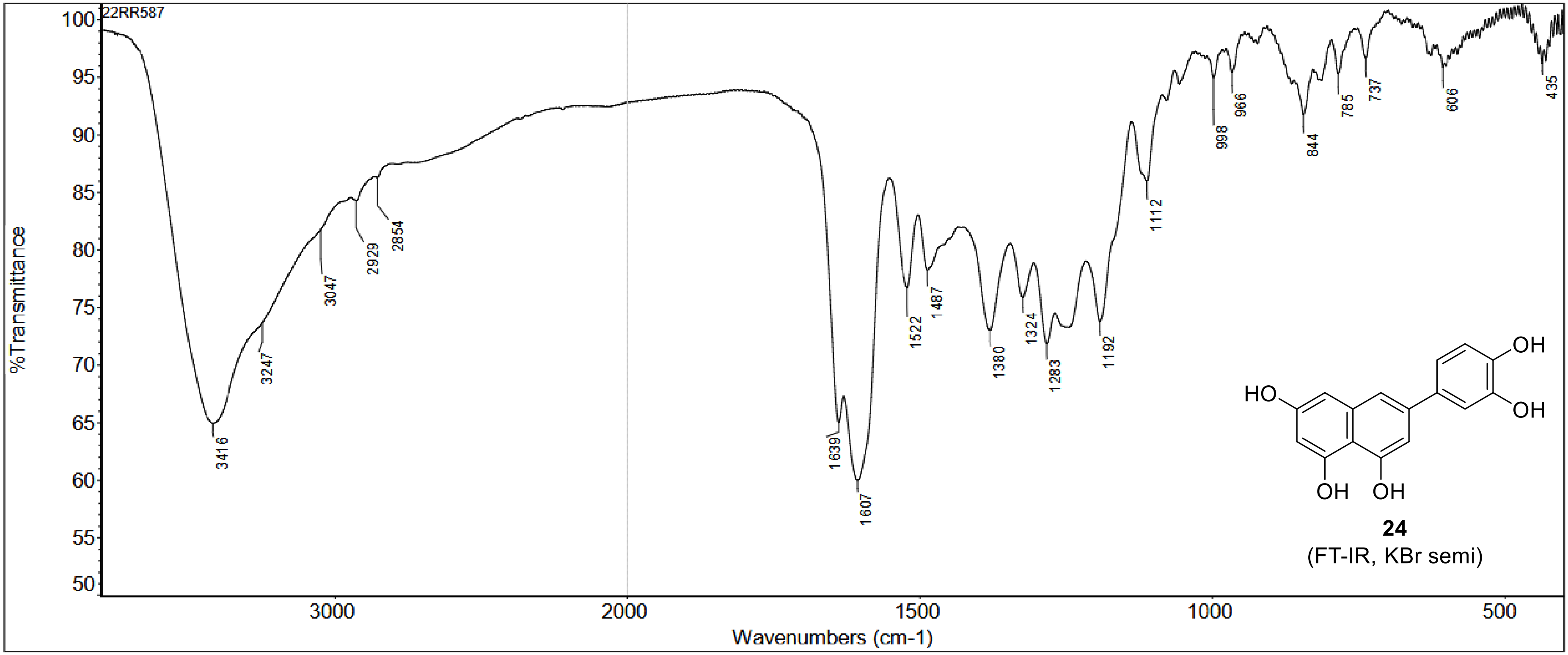

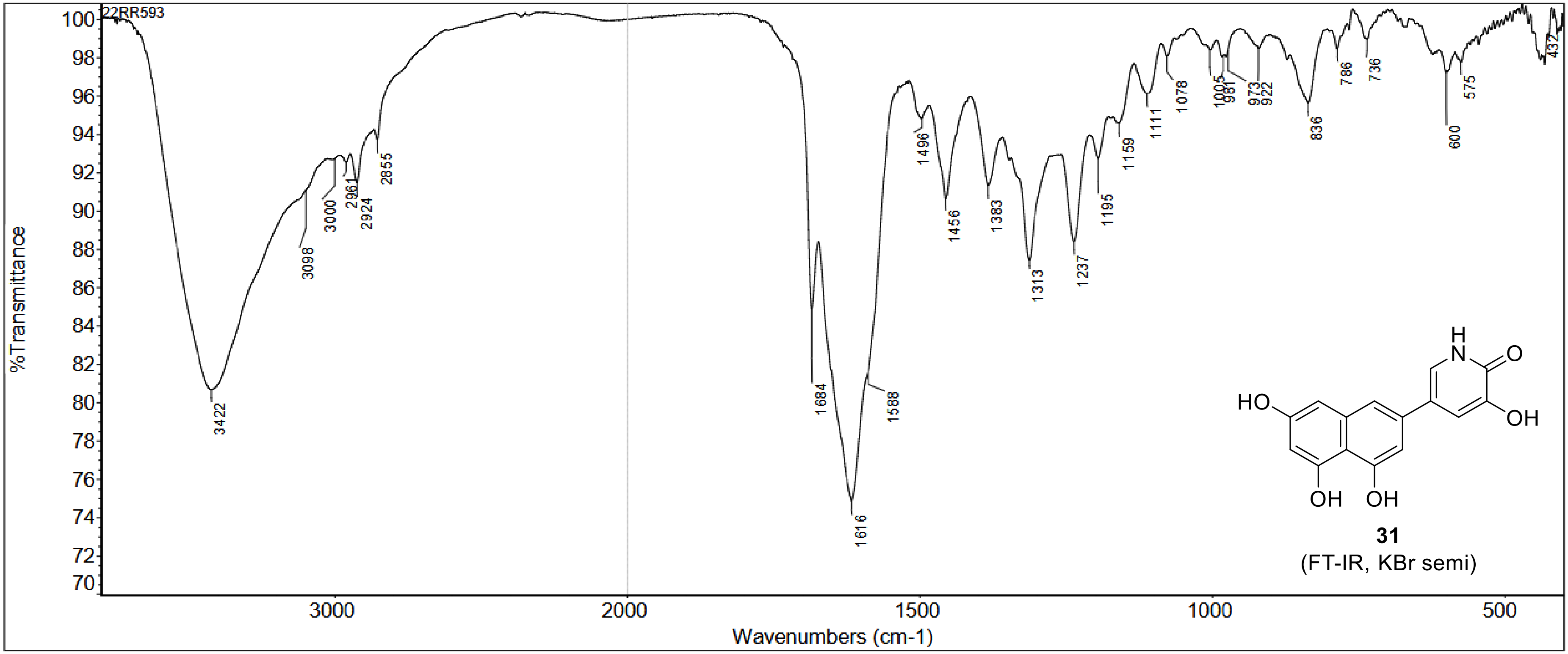


**31**

(FT-IR, KBr semi)

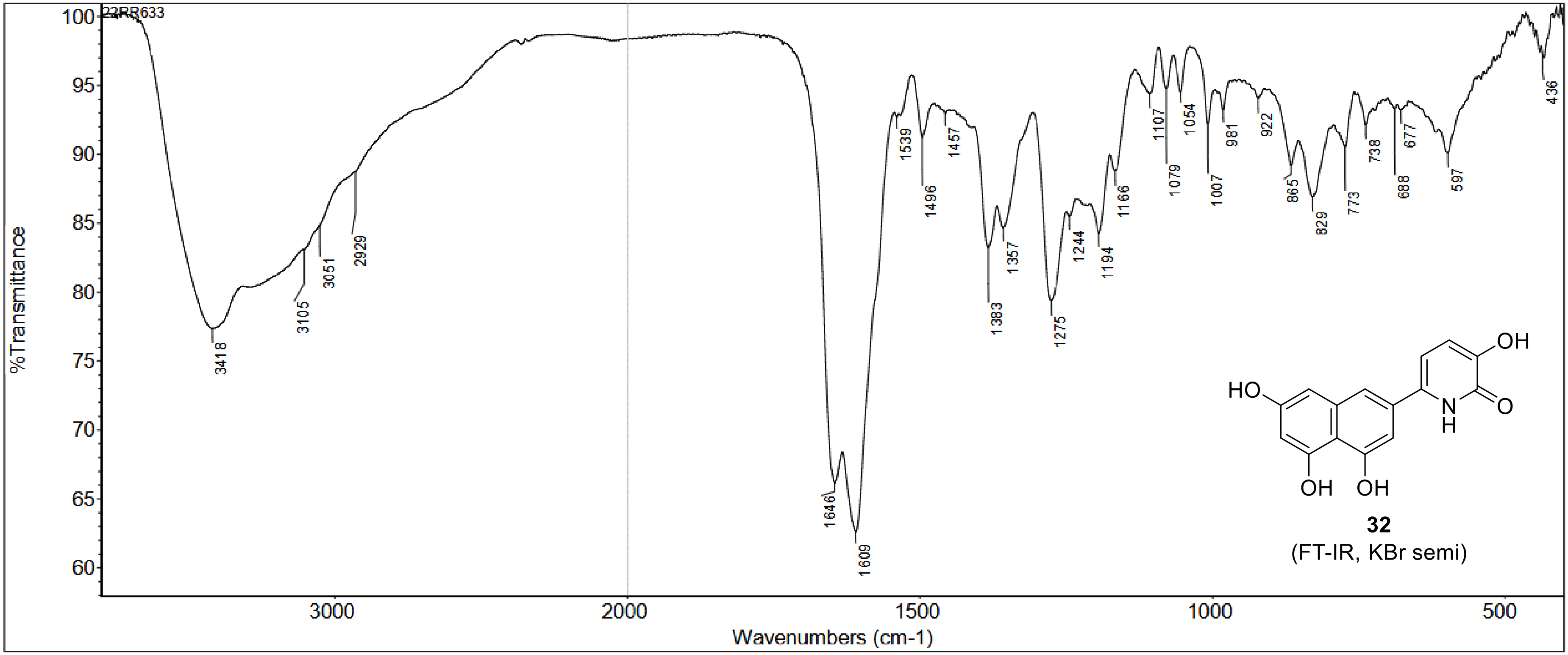
22RR633
%Transmittance
Wavenumbers (cm-1)
3418
3105
3051
2929
1646
1609
1539
1496
1457
1383
1357
1275
1244
1194
1166
1107
1079
1054
1007
981
922
865
829
773
738
688
677
597
436
HO
OH
OH
OH
N
H
O
32
(FT-IR, KBr semi)

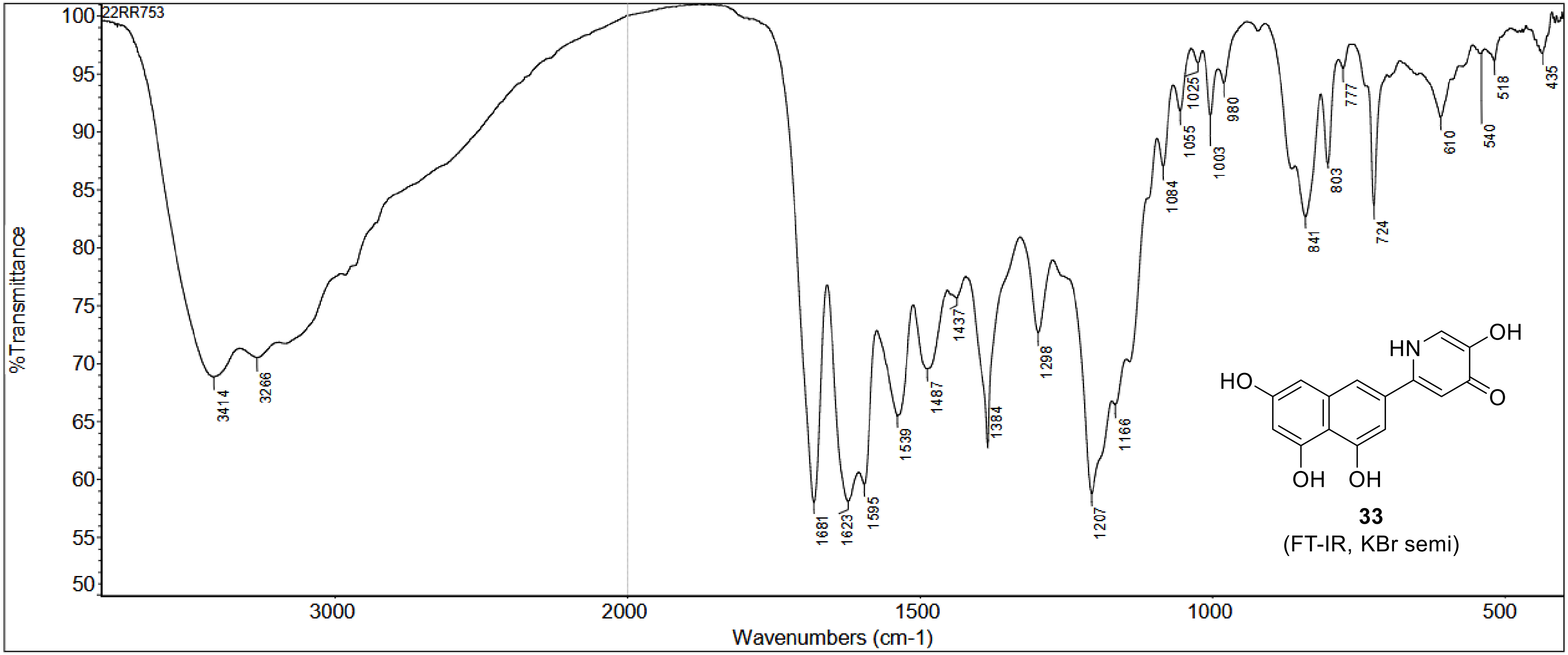
22RR753
%Transmittance
Wavenumbers (cm-1)
3414
3266
1681
1623
1595
1539
1487
1437
1384
1298
1207
1166
1084
1055
1025
1003
980
841
803
777
724
610
540
518
435
HN
OH
HO
O
OH
OH
33
(FT-IR, KBr semi)

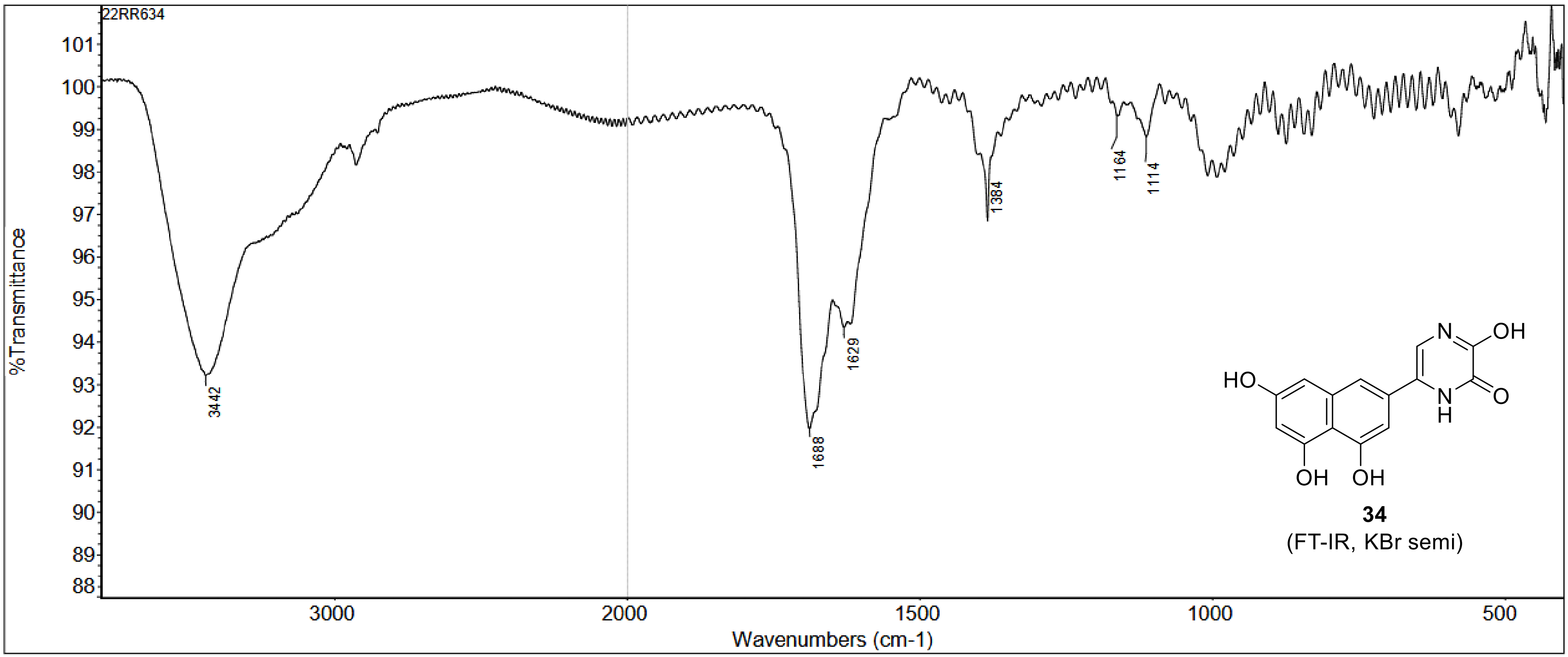
22RR634
%Transmittance
Wavenumbers (cm-1)
3442
1688
1629
1384
1164
1114
HO
OH
OH
N
OH
N
H
O
34
(FT-IR, KBr semi)

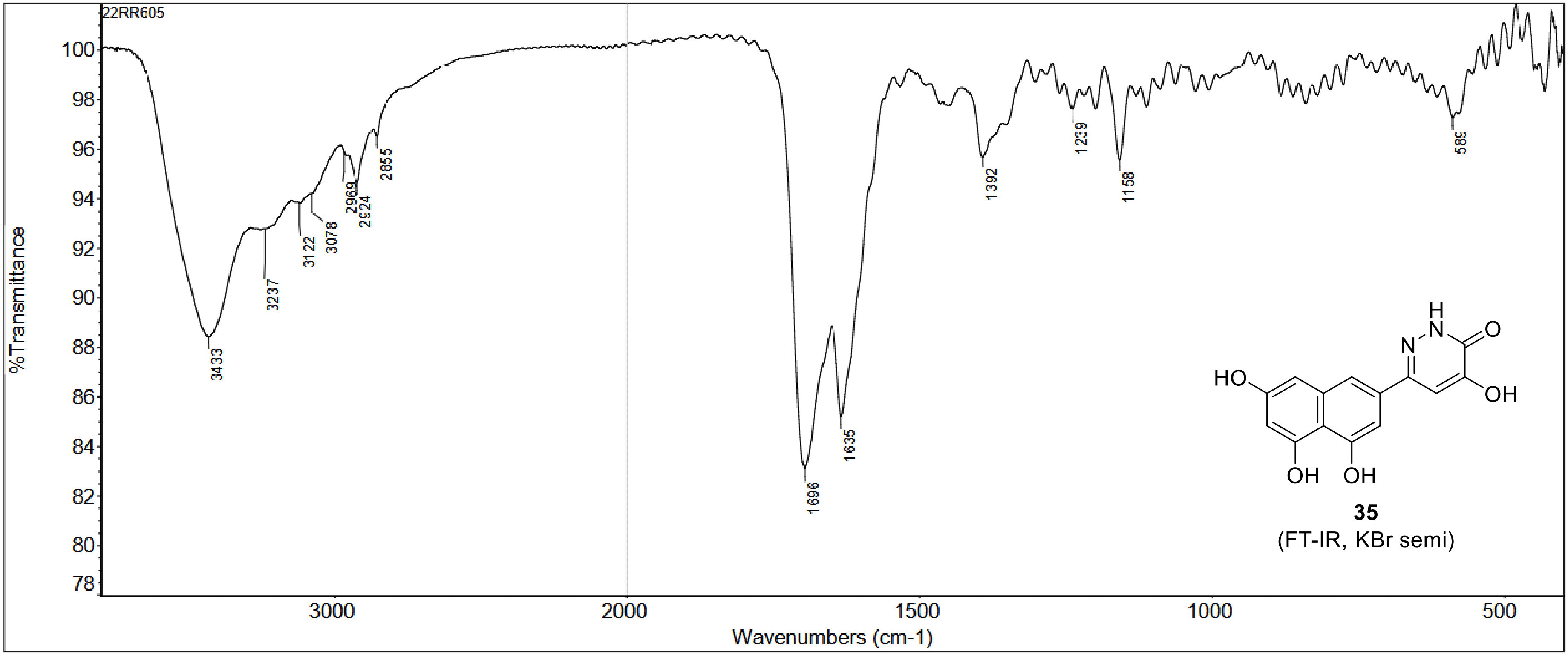
22RR605
%Transmittance
Wavenumbers (cm-1)
3433
3237
3122
3078
2969
2924
2855
1696
1635
1392
1239
1158
589
HO
OH
OH
OH
N
H
N
O
35
(FT-IR, KBr semi)

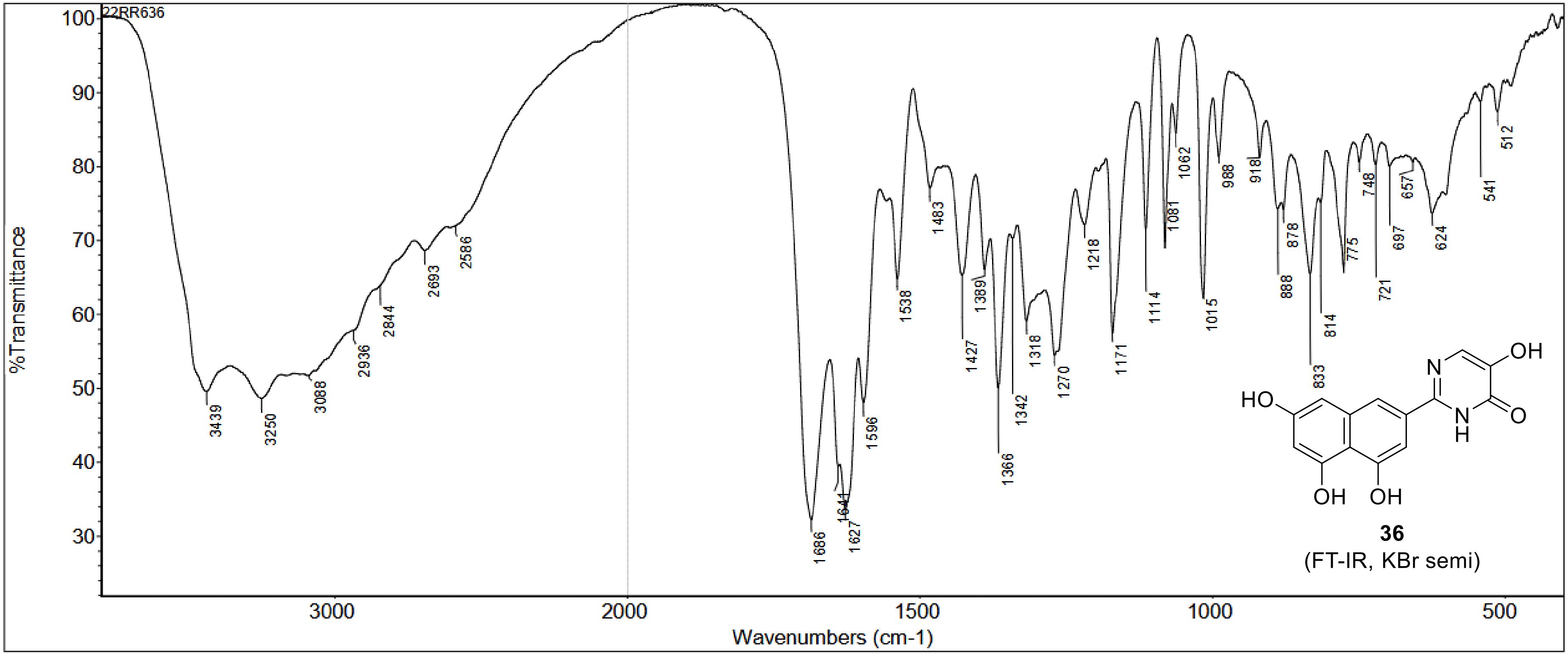
22RR636
%Transmittance
Wavenumbers (cm-1)
3439
3250
3088
2936
2844
2693
2586
1686
1641
1627
1596
1538
1483
1427
1389
1366
1342
1318
1270
1218
1171
1114
1081
1062
1015
988
918
888
878
833
814
775
748
721
697
657
624
541
512
HO
OH
OH
N
N
H
OH
O
36
(FT-IR, KBr semi)

# HRMS spectra

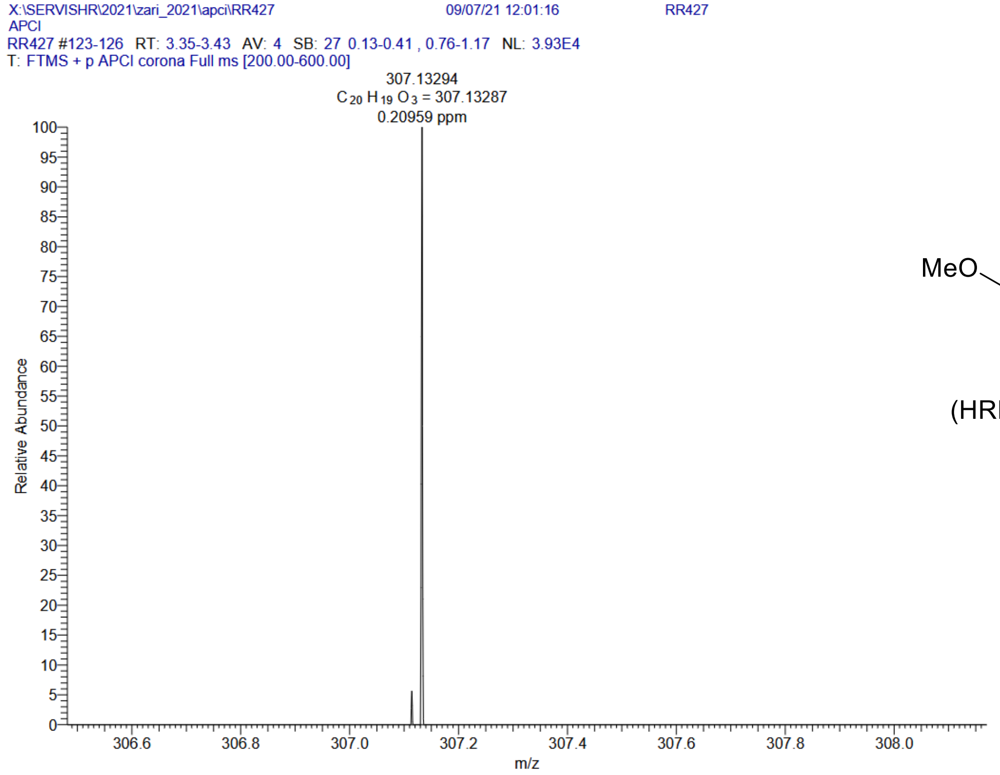


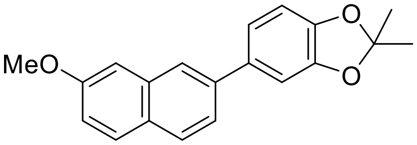


**3**
(HRMS, APCI, positive mode)

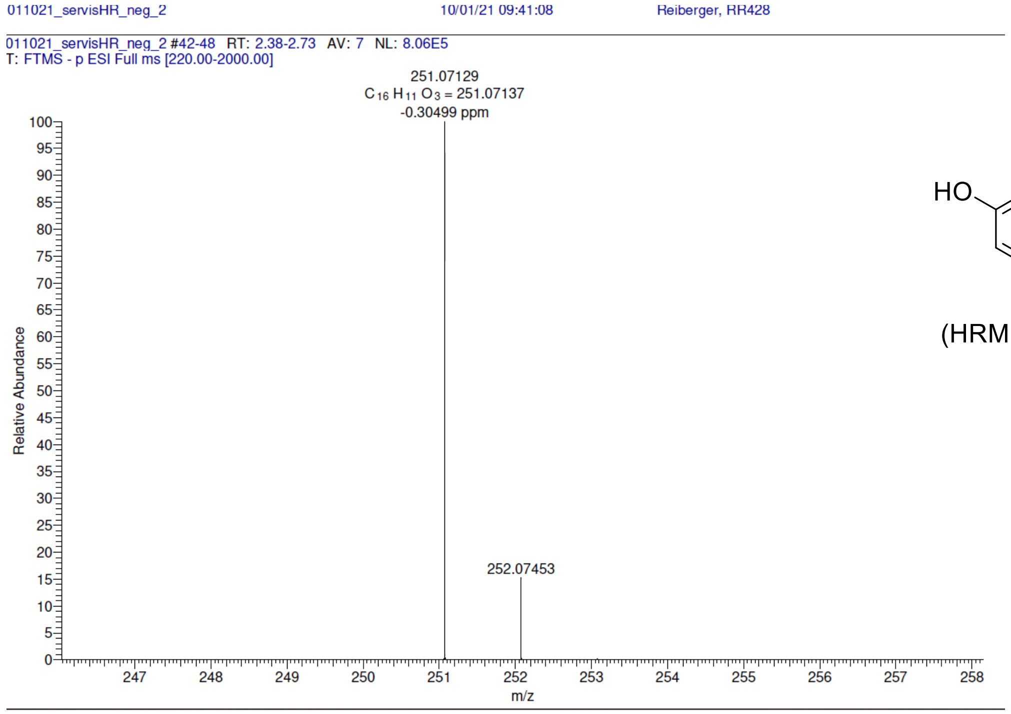


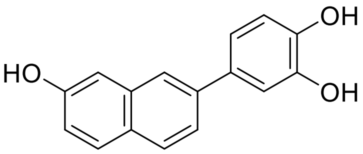


**4**
(HRMS, ESI, negative mode)

Z:\SERVISHR\...\020921_servisHR_2 09/02/21 10:15:00 Reiberger, RR418

020921_servisHR_2 #98-101 RT: 2.62-2.70 AV: 4 NL: 9.38E6
T: FTMS + p ESI Full ms [200.00-2000.00]

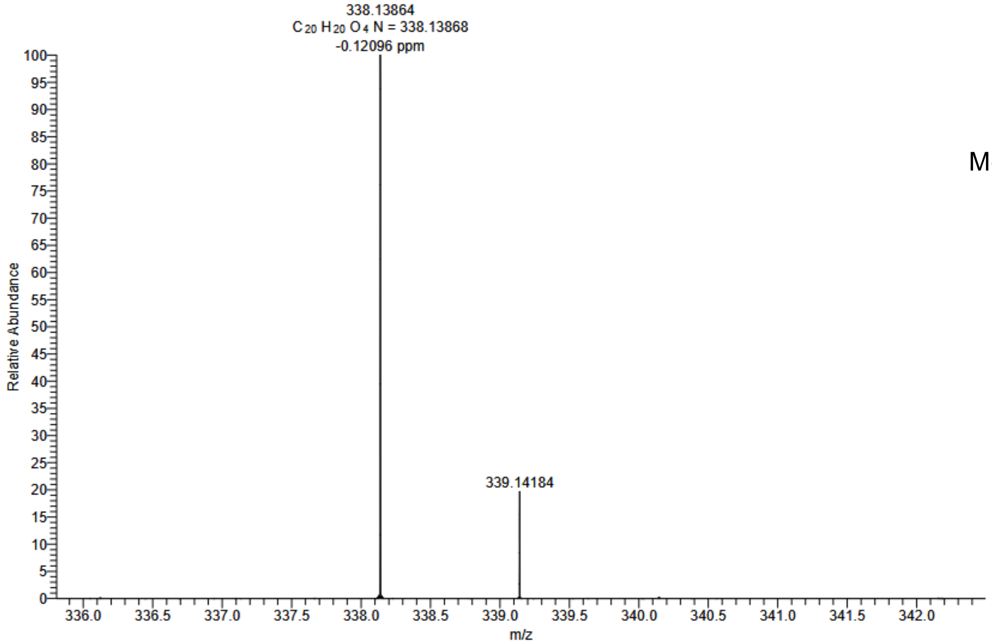


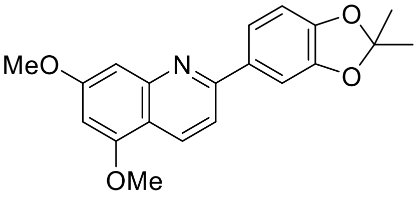


**6**
(HRMS, ESI, positive mode)

110921_servisHR_26 09/13/21 16:12:08 Reiberger RR 422

110921_servisHR_26 #87-95 RT: 2.32-2.54 AV: 9 NL: 9.33E6
T: FTMS + p ESI Full ms [200.00-2000.00]

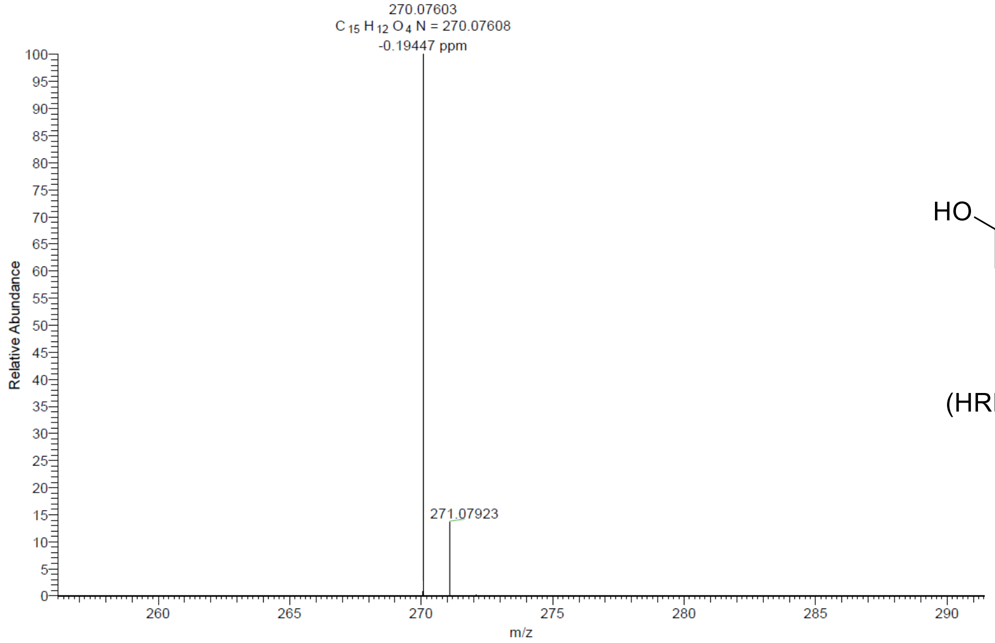


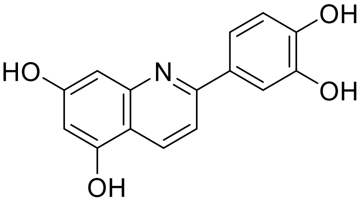


**7**
(HRMS, ESI, positive mode)

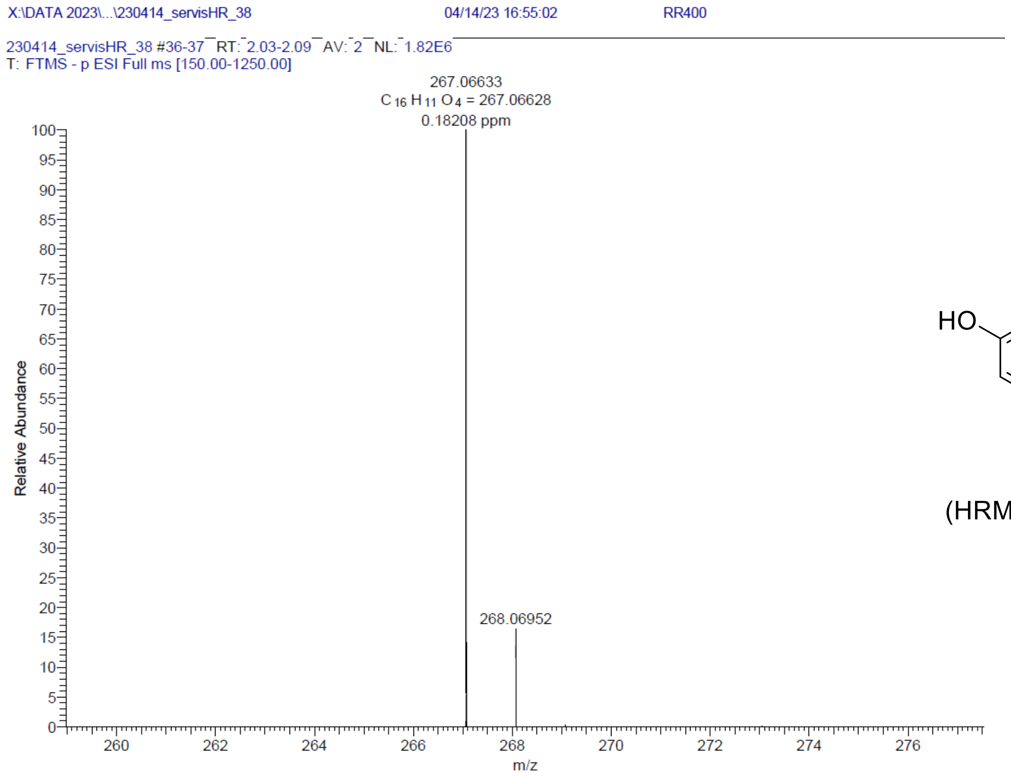


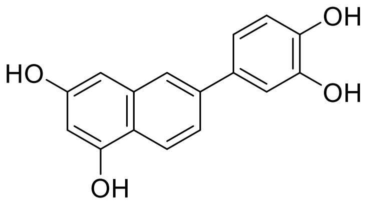


**12**
(HRMS, ESI, negative mode)

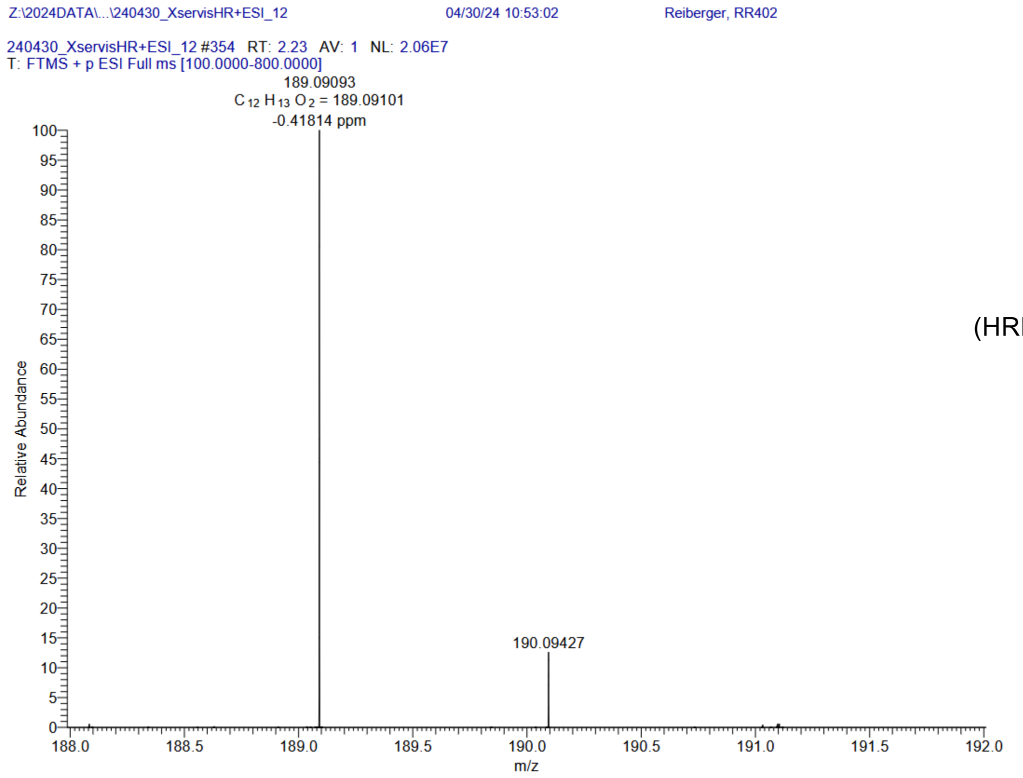


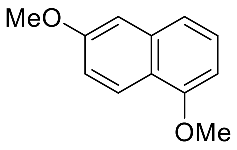


**14**
(HRMS, ESI, postive mode)

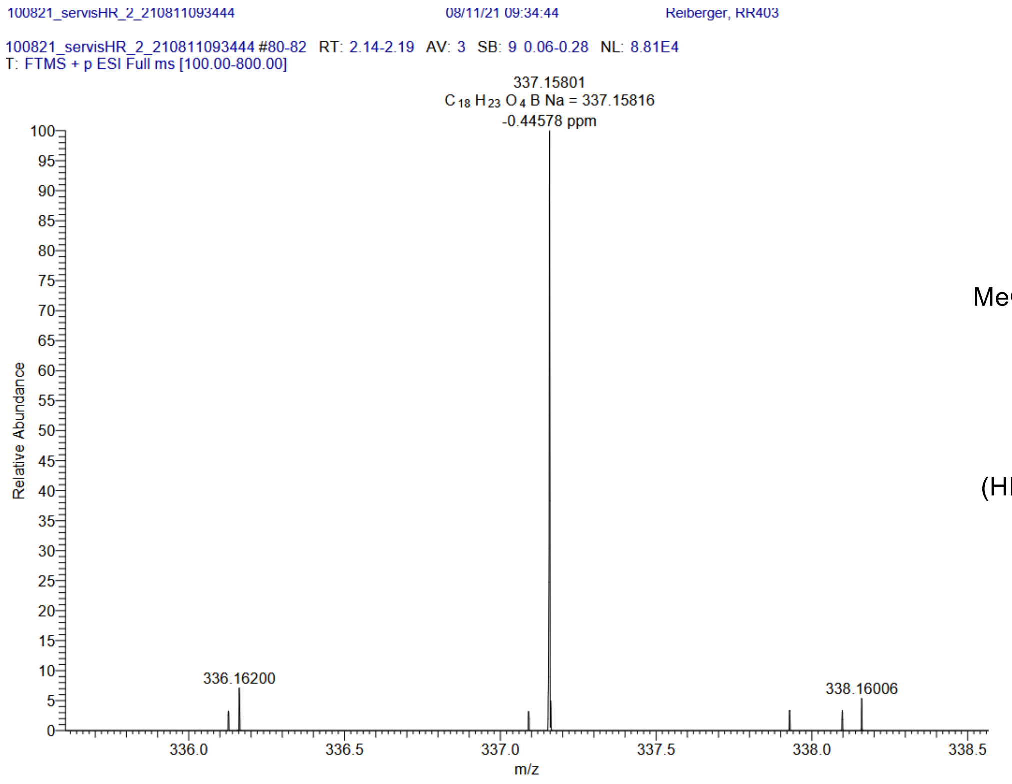


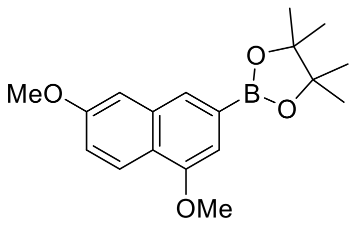


**15**
(HRMS, ESI, postive mode)

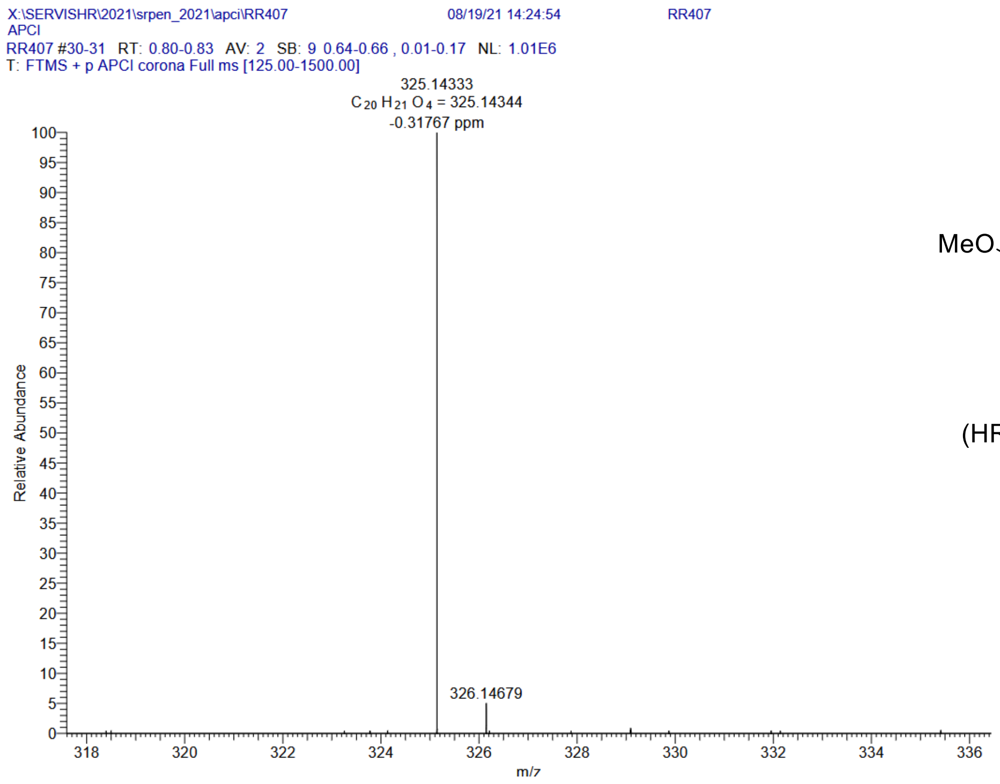


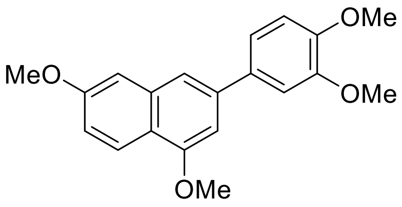


**16**
(HRMS, APCI, postive mode)

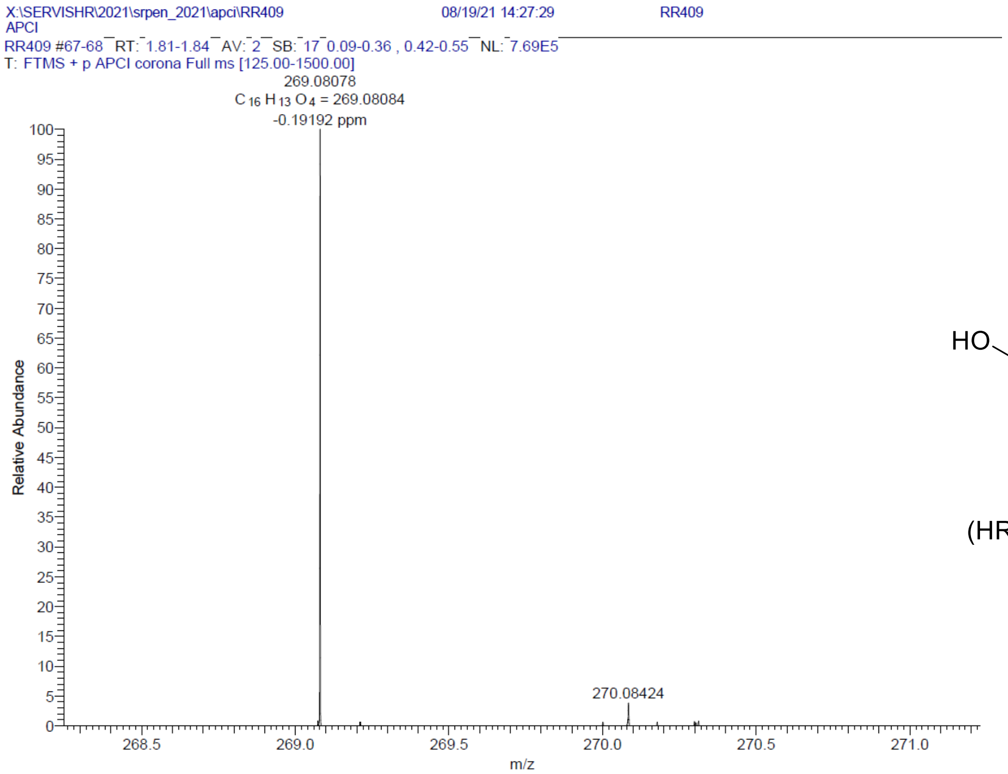


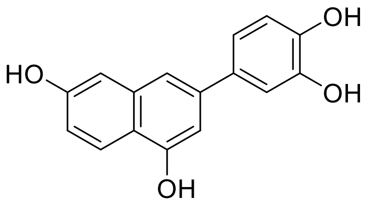


**17**

(HRMS, ESI, postive mode)

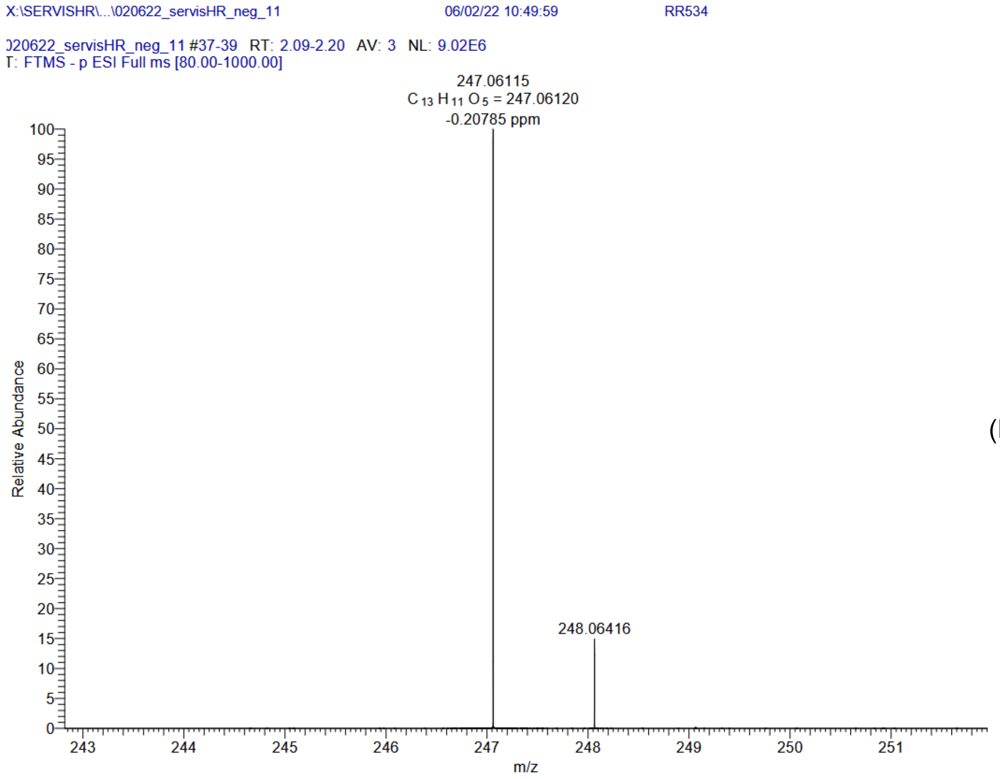


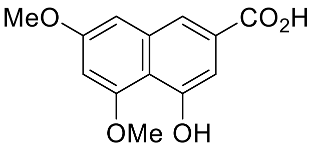


**20**

(HRMS, ESI, negative mode)

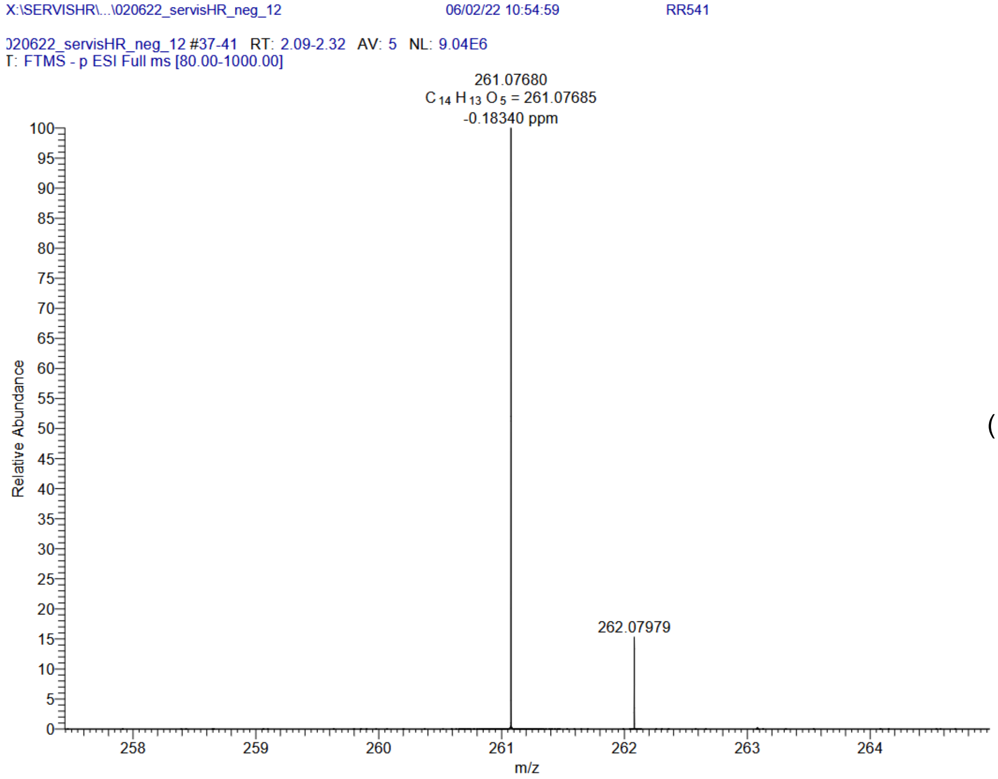


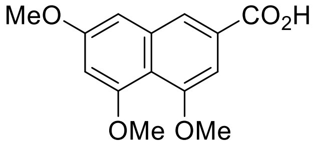


**21**
(HRMS, ESI, negative mode)

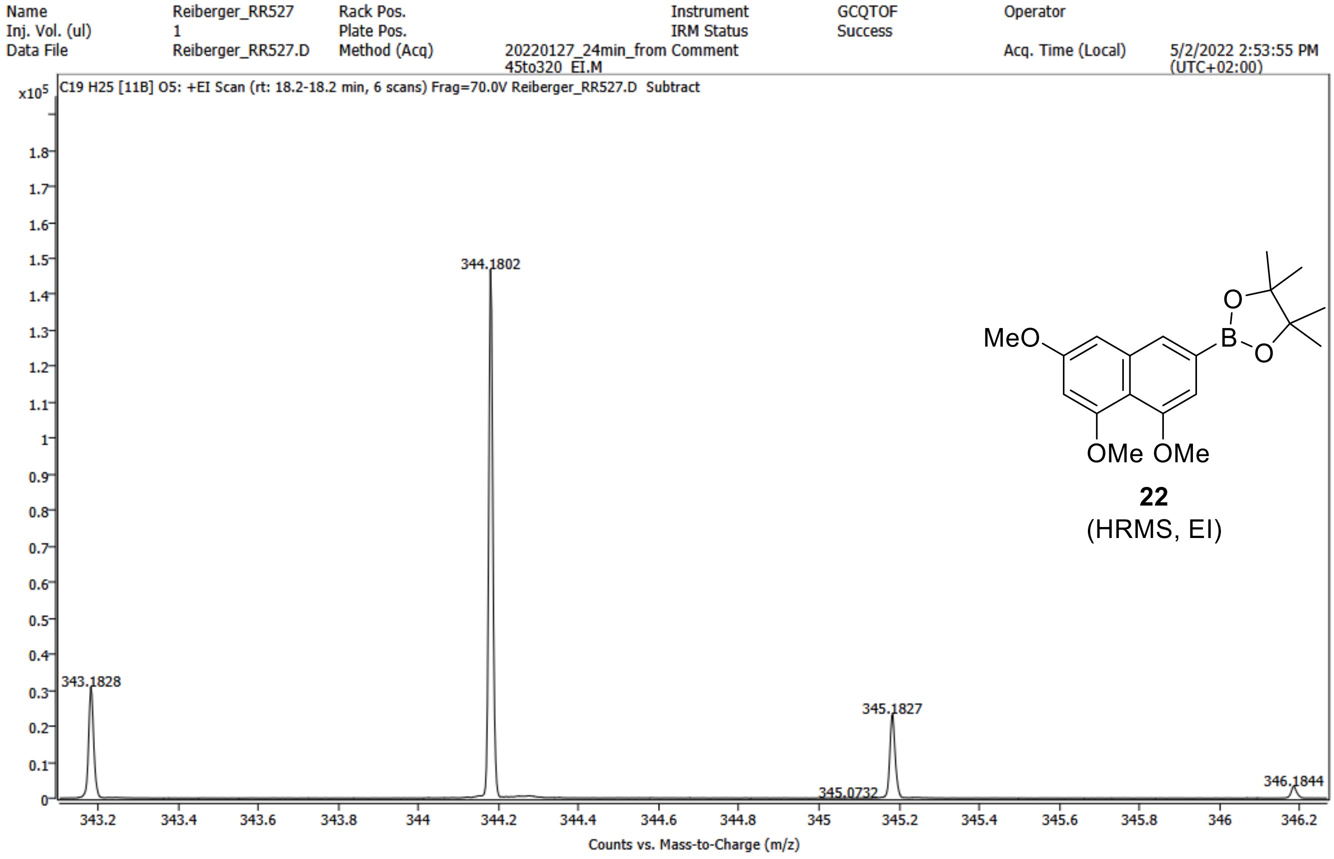


**22**
(HRMS, EI)

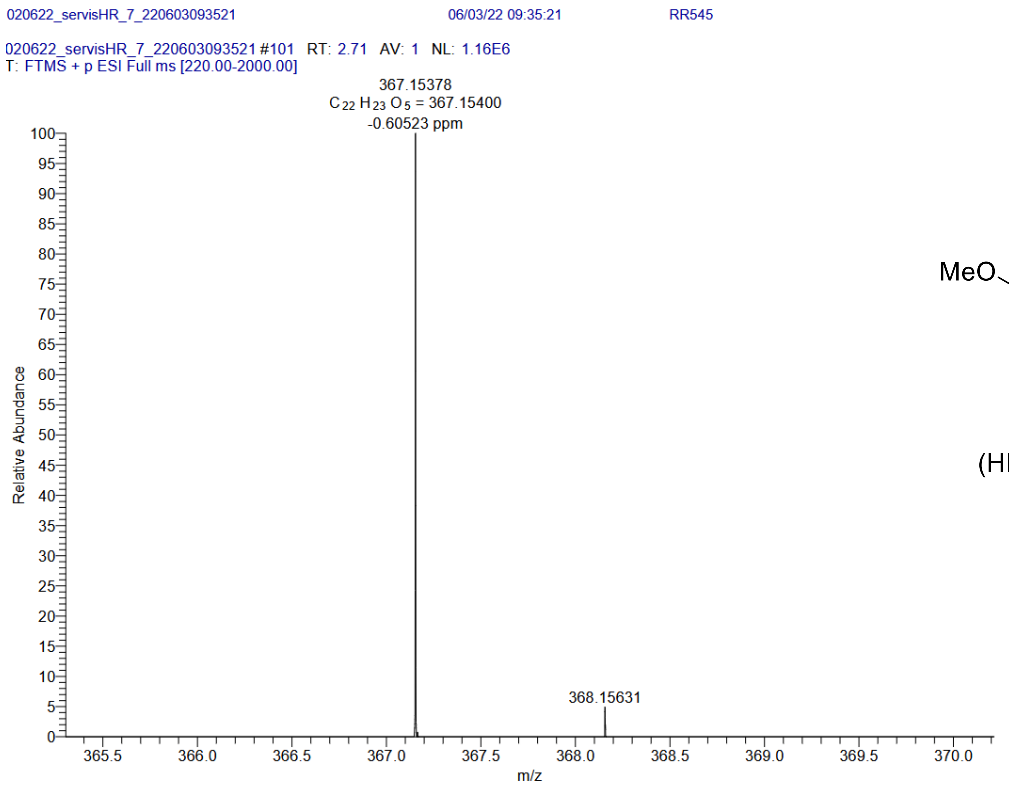


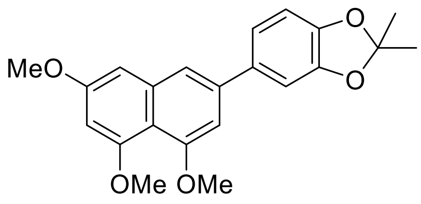


**23**

(HRMS, ESI, positive mode)

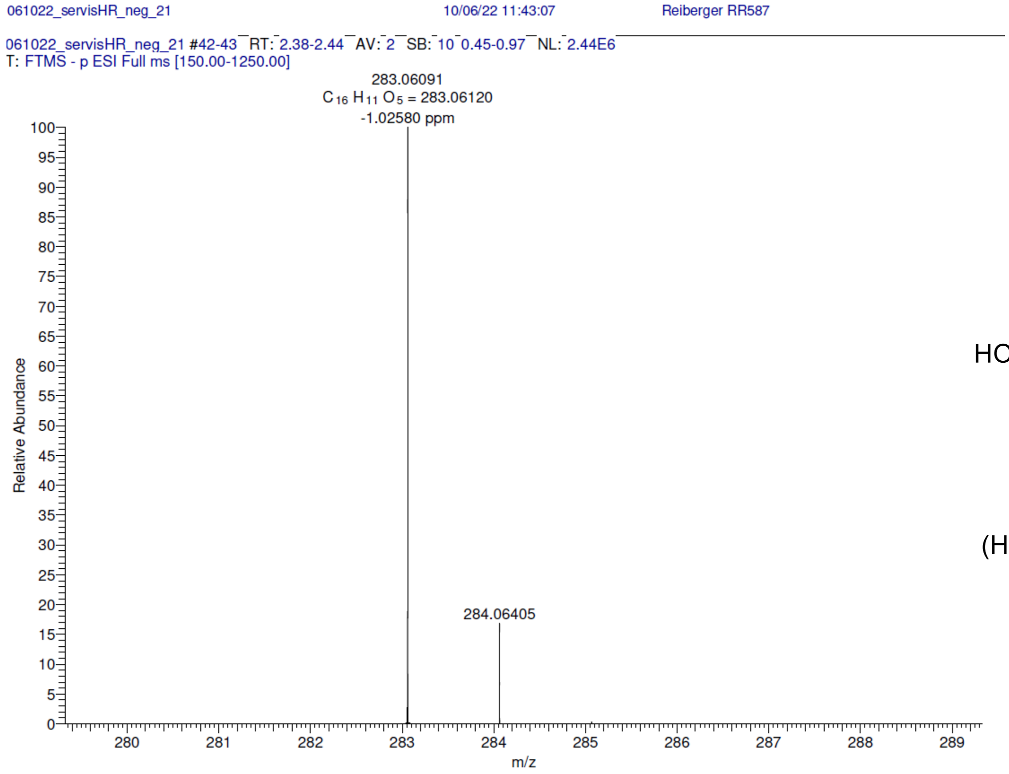


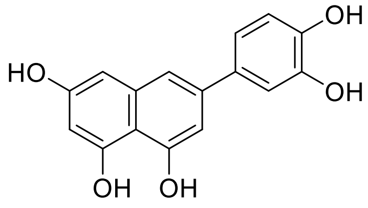


**24**

(HRMS, ESI, negative mode)

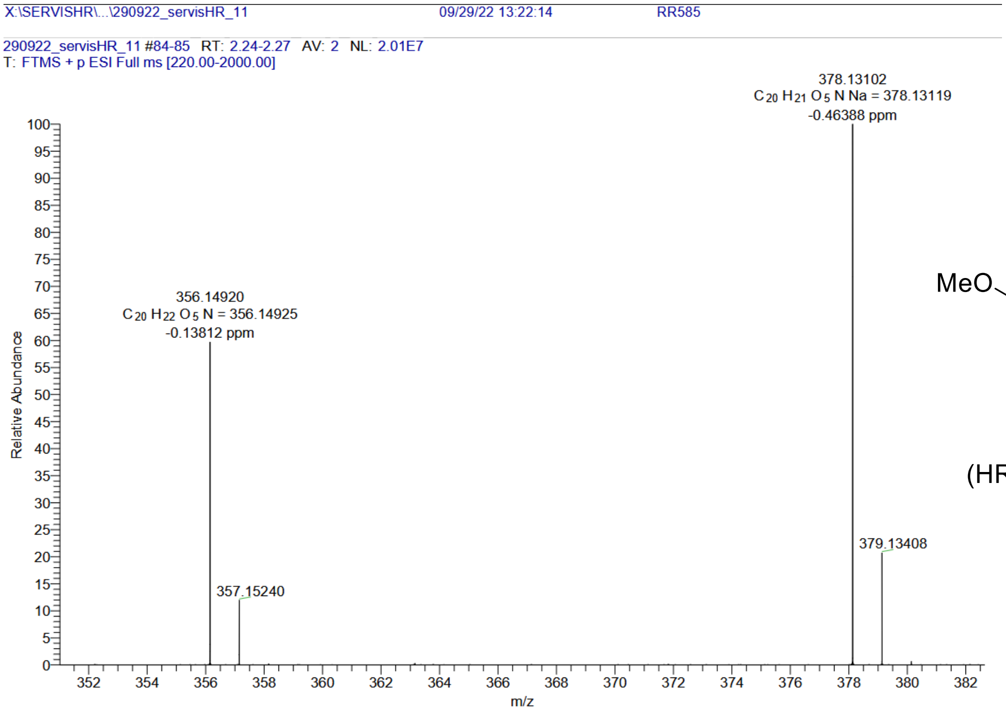


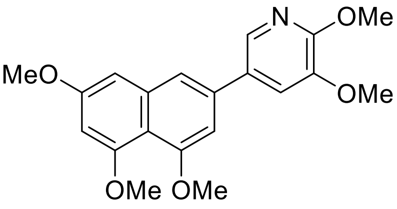


**25**
(HRMS, ESI, positive mode)

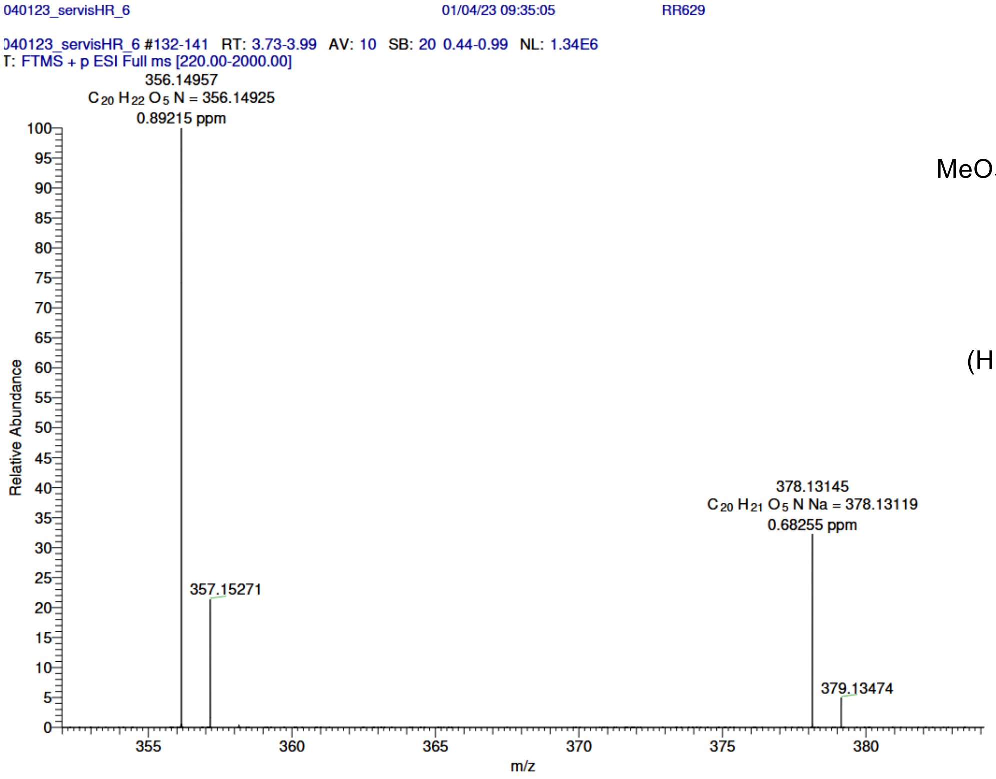


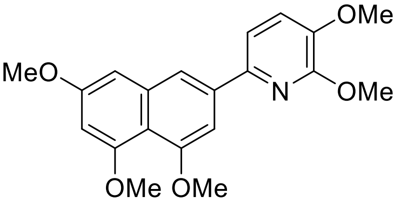


**26**
(HRMS, ESI, positive mode)

V:\2023DATA\...\230818_XservisHR+ESI_05 08/18/23 09:24:43 Reiberger RR751_1

230818_XservisHR+ESI_05 #542-566 RT: 3.23-3.37 AV: 25 SB: 91 0.45-1.00 NL: 8.43E7
T: FTMS + p ESI Full ms [200.0000-2000.0000]

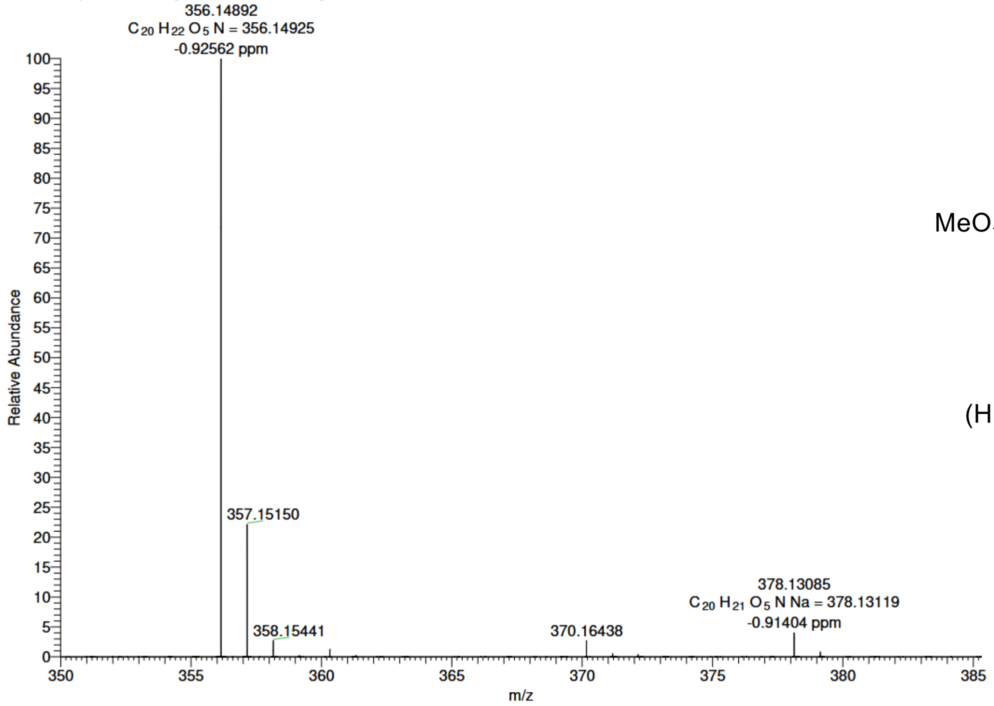


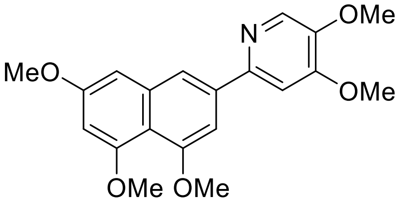


**27**

(HRMS, ESI, positive mode)

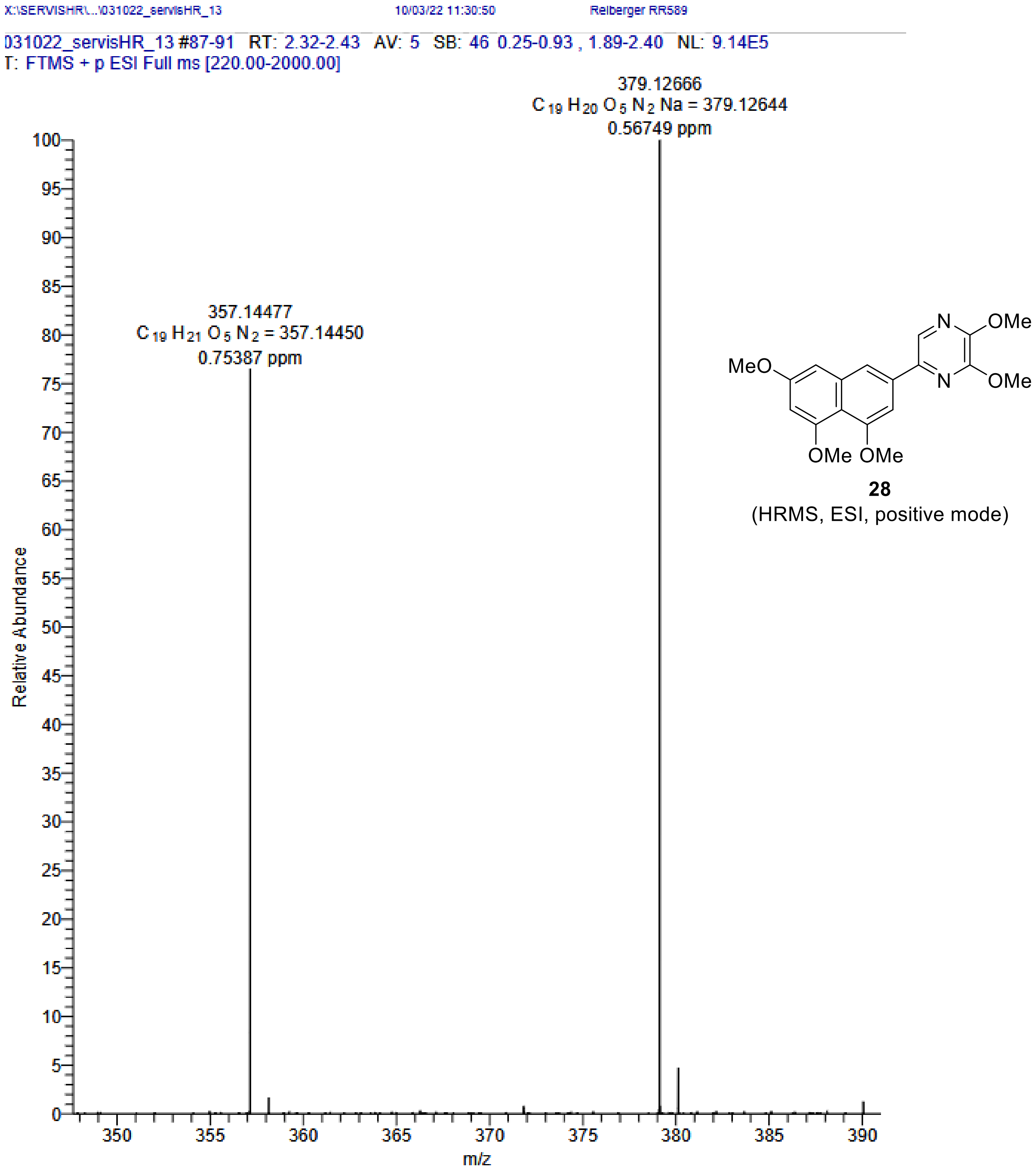


**28**

(HRMS, ESI, positive mode)

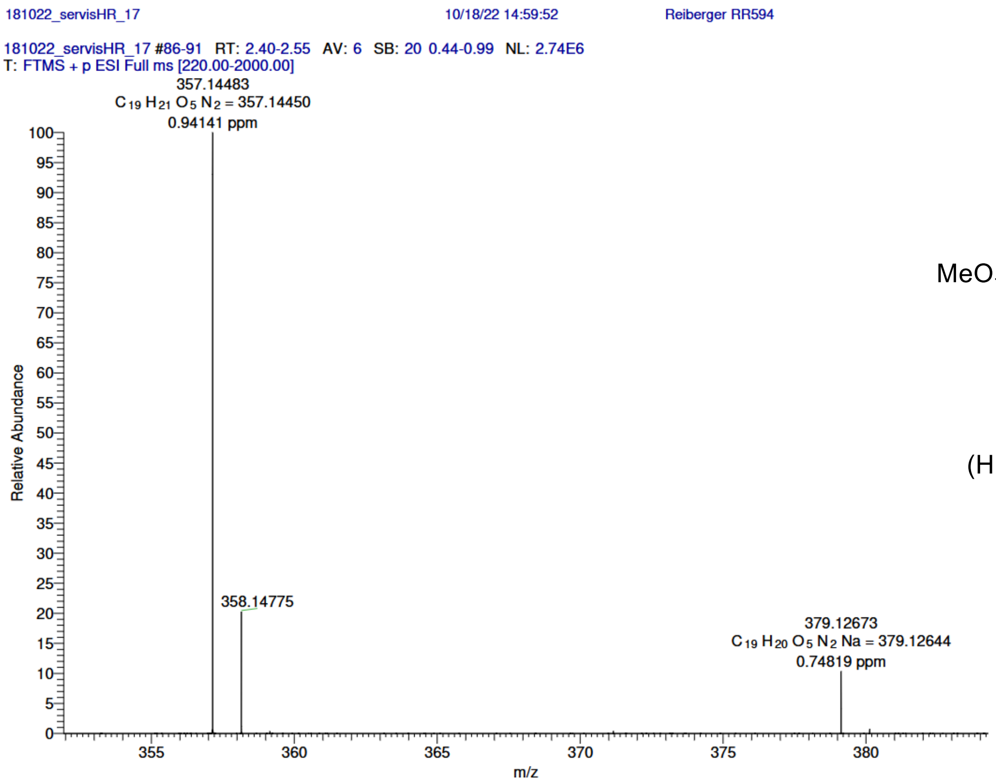


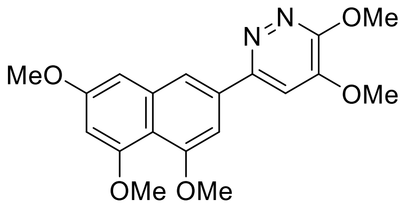


**29**

(HRMS, ESI, positive mode)

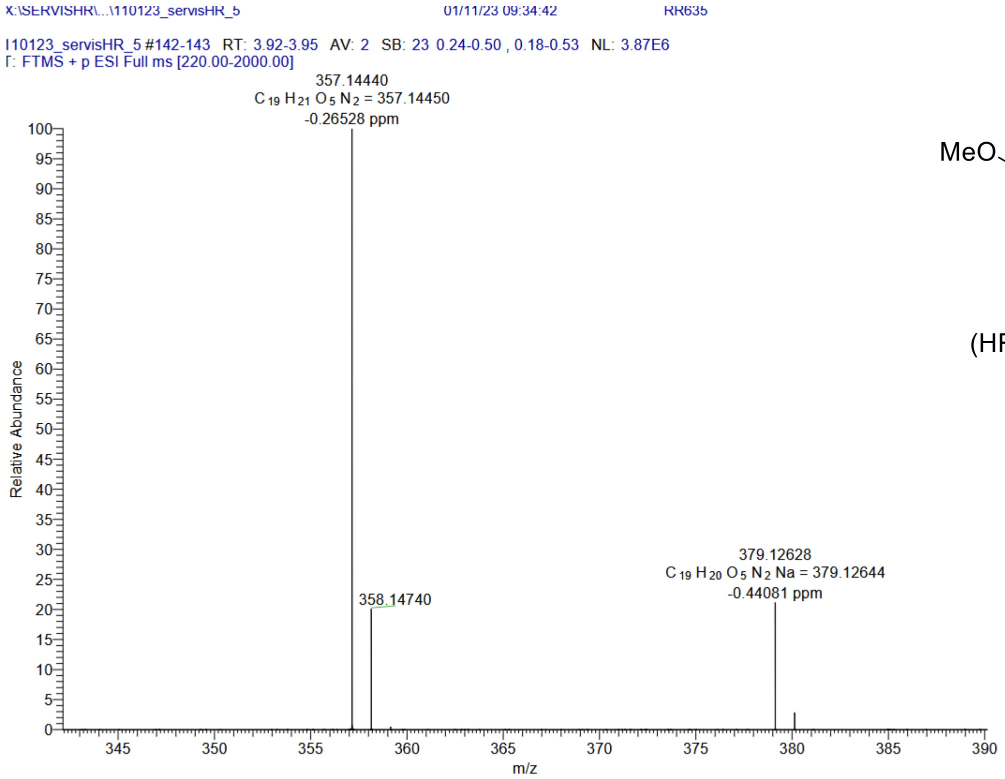


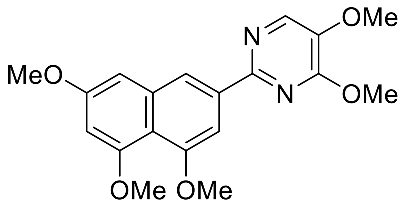


**30**

(HRMS, ESI, positive mode)

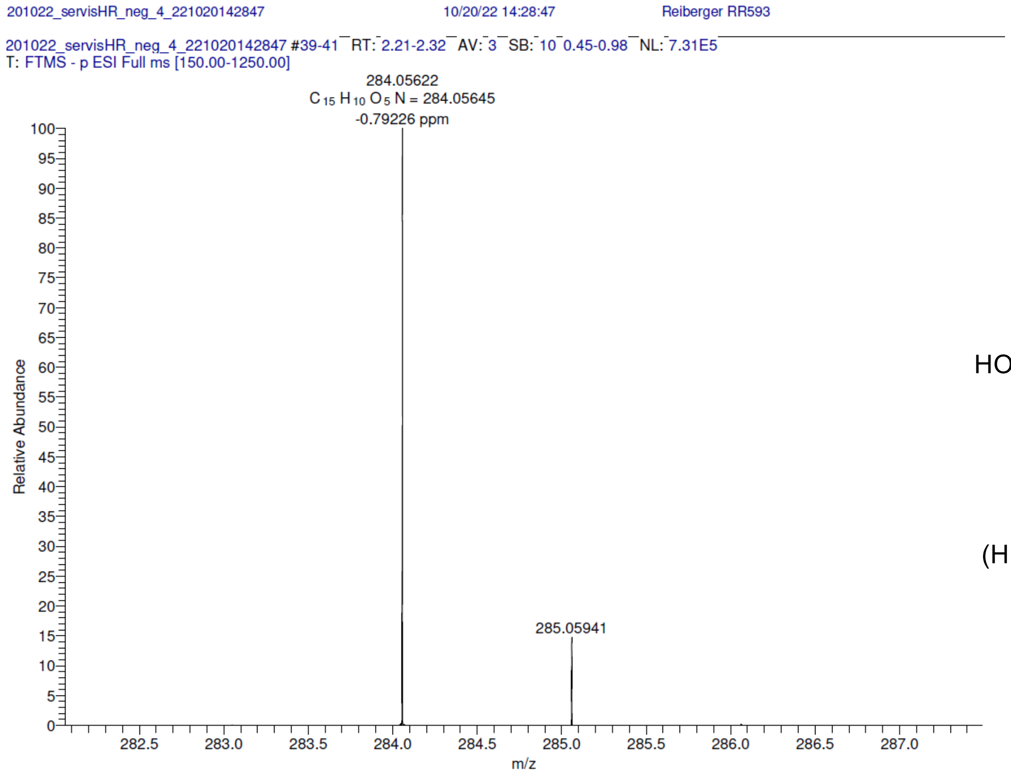


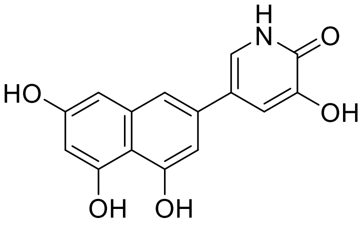

**31**
(HRMS, ESI, negative mode)

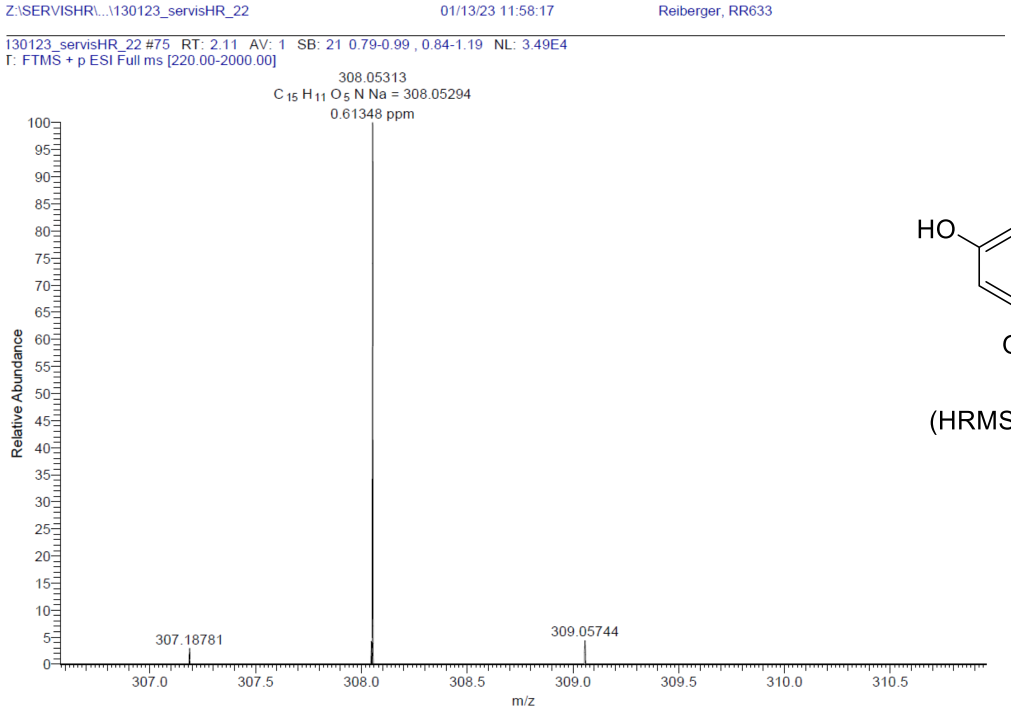


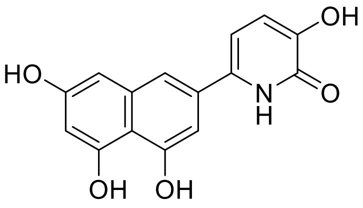

**32**
(HRMS, ESI, positive mode)

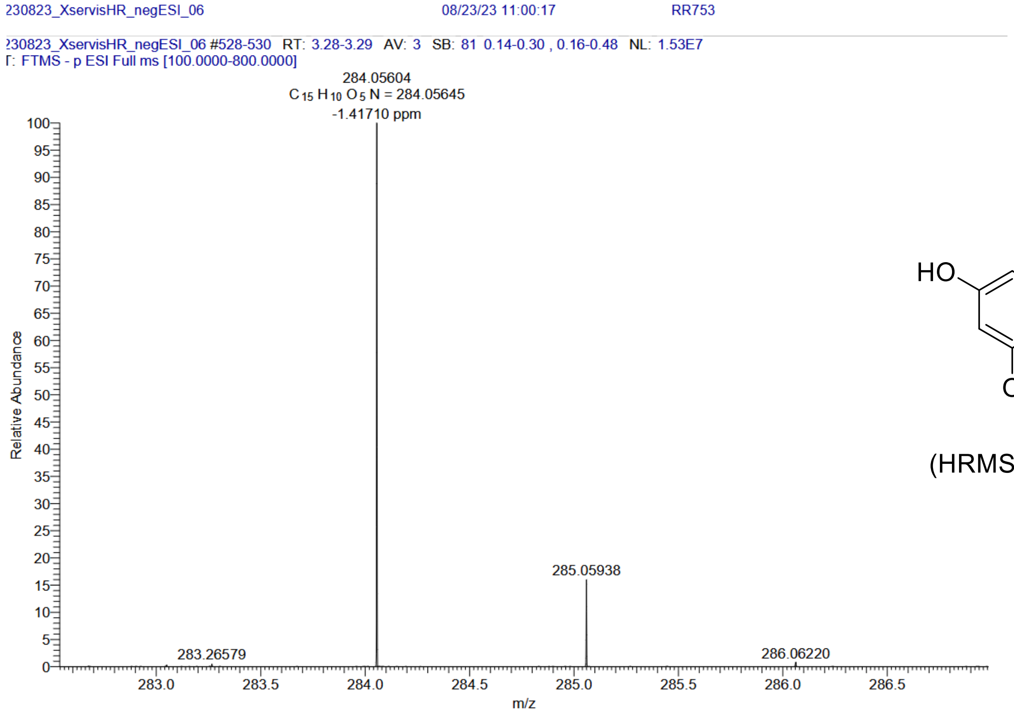


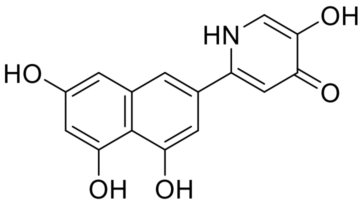


**33**

(HRMS, ESI, positive mode)

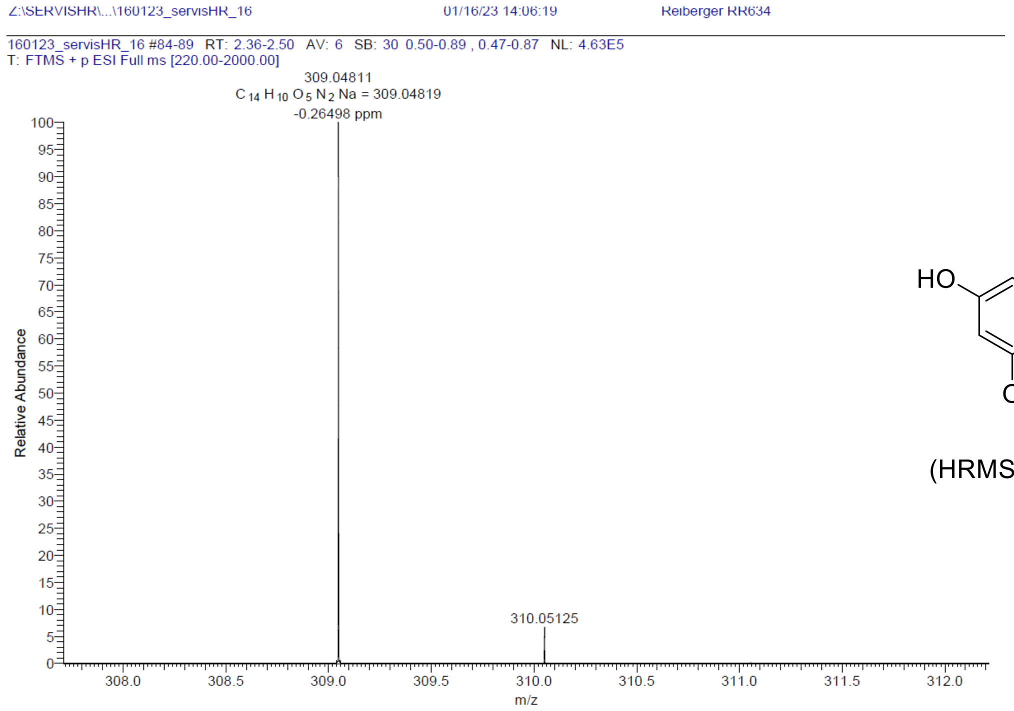


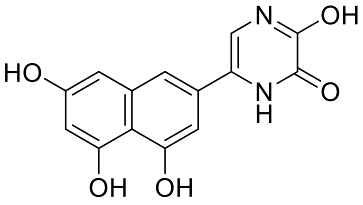


**34**

(HRMS, ESI, positive mode)

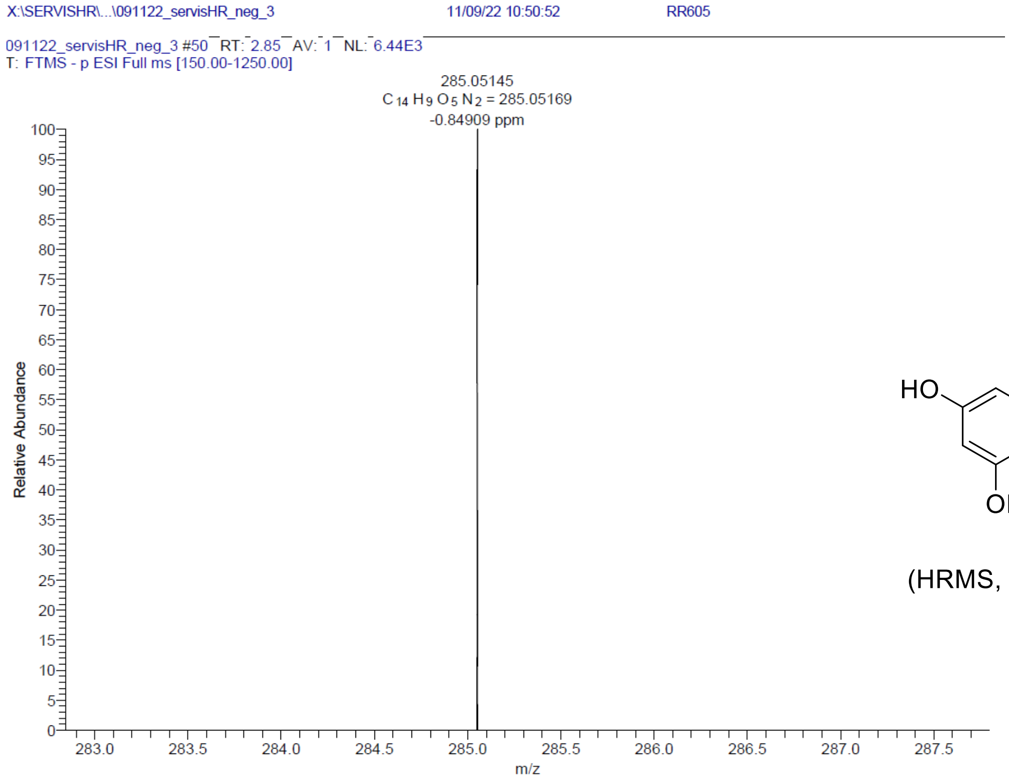


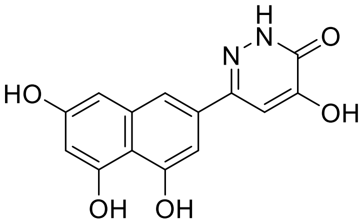


**35**
(HRMS, ESI, negative mode)

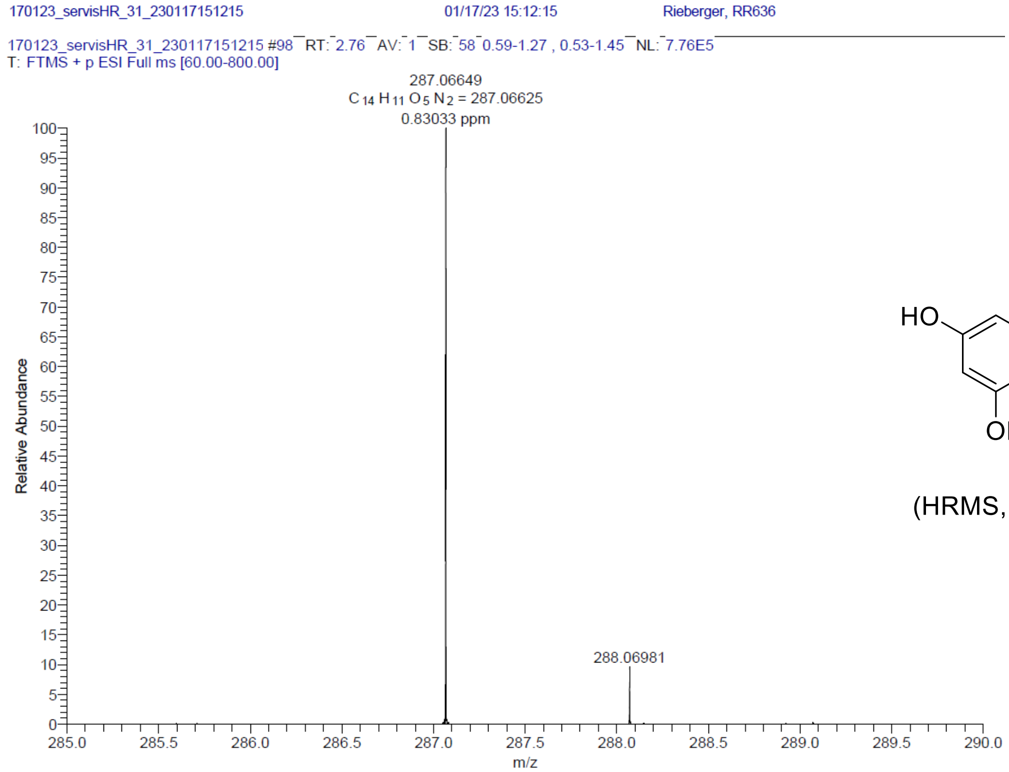


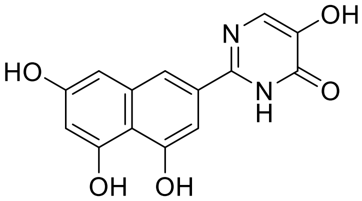


**36**
(HRMS, ESI, positive mode)
